# Pediatric Simulation-Based Prehospital Training Course in Botswana

**DOI:** 10.21980/J8306S

**Published:** 2021-07-15

**Authors:** Nicolaus W Glomb, Marideth C Rus, Adeola Adekunbi Kosoko, Sharmistha Saha, Kristen Murphy, Cara B Doughty, Cafen Galapi, Bushe Laba, Manish I Shah

**Affiliations:** * University of California, San Francisco, Department of Emergency Medicine, San Francisco, CA; ^ Baylor College of Medicine, Department of Pediatrics, Houston, TX; ‡ McGovern Medical School at the University of Texas Health Science Center at Houston, Department of Emergency Medicine, Houston, Tx; ** University of Texas Southwestern Medical Center, Department of Pediatrics, Dallas, TX; ^^ Botswana Ministry of Health and Wellness, Emergency Medical Services, Gaborone, Botswana

## Abstract

**Audience:**

This simulation-based training focuses on the most common and high risk pediatric prehospital scenarios in low- and middle-income countries (LMIC). The curriculum was developed based on a needs assessment to train Ministry of Health and Wellness (MOHW) prehospital providers in Botswana specifically for pediatric resuscitation and could be used for emergency medical services (EMS) providers in other LMIC. After participating in this curriculum, providers should enhance their assessment and interventions in acutely ill pediatric prehospital patients.

**Length of Curriculum:**

The entire course was designed to be presented over two days with 6–8 hours of instruction each day.

**Introduction:**

In recent years, prehospital medicine has shown continued growth in LMICs, specifically in Sub-Saharan Africa. As these programs develop focused training for the pediatric population, equipping the workforce with pediatric resuscitation skills is essential. A few years after its inception, the Botswana MOHW identified deficiencies in their current training program and sought external expertise and educational training. We partnered with the MOHW to create and implement a novel, prehospital simulation curriculum to teach pediatric resuscitation to prehospital providers. Our aim was to create a curriculum based on the needs of the community that could also be implemented in other similar resource-limited settings. This course included didactic sessions, five simulation scenarios using low fidelity mannequins and three pediatric-focused skill sessions. This program was found to be effective based on statistically significant improvement in written and simulation post-test scores.

**Educational Goals:**

The objective of this educational project was to design, implement, and evaluate a curriculum relevant to an EMS system based in a LMIC, so that it could be a basis for curricula for use in similar contexts. The educational goal is to improve prehospital providers performance in common pediatric resuscitations.

**Educational Methods:**

The educational methods used in this curriculum included simulation using rapid cycle deliberate practice (RCDP), didactic lectures, and hands on skills training for common pediatric scenarios. Outcomes were measured by comparing performance on written and simulation-based pre-and post-tests.

**Research Methods:**

Participants completed written and simulation-based pre- and post-tests covering the concepts taught in the curriculum. Continuous variables (written and simulation test scores) were compared between two dependent groups (pre- and post-trainings) using paired t-tests.

**Results:**

Mean written test scores increased by 11%, from 75% to 86% (p<0.0001), while mean simulated test scores increased by 22% (from 56% to 78 % (p<0.0001).

**Discussion:**

The curriculum we developed focused on high-yield pediatric skills based on the needs of the Botswana MOHW EMS program. We believe simulation training was an excellent and effective method for this type of training. We specifically designed RCDP scenarios for the training, due to the limited experience of the prehospital providers at that time. RCDP offers ample opportunities for feedback with immediate practice and improvement. Trainees demonstrated retention of knowledge and improved performance in simulation-based testing. The overall satisfaction level of the trainees was high and suggests additional training would be beneficial and desired. Additionally, as the results of our needs assessment mirrored common chief complaints in other LMIC countries in Sub-Saharan Africa^1^,^2^ we feel that this curriculum can be utilized and adopted with minor modifications in other LMIC settings, particularly where EMS programs are developing and in circumstances where few EMS providers have had extensive field experience.

**Topics:**

Respiratory distress, asthma, dehydration, hypovolemic shock, hypoglycemia, seizure, toxic ingestion, newborn resuscitation, precipitous delivery, traumatic injury, EMS, Botswana, global health, collaboration, rapid cycle deliberate practice (RCDP), medical simulation.

## USER GUIDE

[Table t1-jetem-6-3-c64]

**Table t1-jetem-6-3-c64:** 

List of Resources:	
Abstract	63
User Guide	66
Didactics and Hands-On Curriculum Chart	71
Appendix A: Prehospital Pediatric Written Test - Questions	84
Appendix B: Prehospital Pediatric Written Test - Answers	88
Appendix C: Simulation Testing Scenario Materials	92
Appendix D: Simulation Testing Scenario Assessment Tool	99
Appendix E: SKILLS STATION—Airway	107
Appendix F: SKILLS STATION—IV/IO/Length-Based Tape	112
Appendix G: SKILLS STATION—Cardiopulmonary Resuscitation	114
Appendix H: Pediatric Simulation Case: Respiratory Distress from Asthma	115
Appendix I: Pediatric Simulation Case: Diarrhea with Hypovolemic Shock	130
Appendix J: Pediatric Simulation Case: Newborn Delivery	142
Appendix K: Pediatric Simulation Case: Seizure	154
Appendix L: Pediatric Simulation Case: Trauma	167
Appendix M: Simulation Course for Prehospital Providers Course Evaluation	180
Appendix N: Equipment Setup for Simulation Scenarios	181
Appendix O: Debriefing Techniques	183
Appendix P: Commonly Used Abbreviations	184
Appendix Q: Lecture 1—Trauma Evaluation	185
Appendix R: Lecture 2—Introduction to Simulation	186
Appendix S: Lecture 3—Delivery and Neonatal Resuscitation	187
Appendix T: Lecture 4—EMS Pediatric Assessment	188
Appendix U: EMS Pediatric Training Course Schedule	189

**Learner Audience:**

Prehospital providers including emergency medical technicians, registered nurses, respiratory therapists, and physicians at any level of training.

**Recommended Number of Instructors:**

- 1 simulation instructor/debriefing facilitator and 1 confederate/assistant per group of three to six learners

**Length of Curriculum:**

The curriculum was designed to be taught over two days, six to eight hours per day. The curriculum consisted of five simulation sessions, lasting 50–60 minutes each, as well as skill sessions and didactic lectures. Each participant had up to 15 minutes to complete pre- and post-simulation testing. Most learners completed pre- and post-written testing in 15–30 minutes.

Day 1 included written and multiple-choice pre-testing (Appendix A, B), simulation pre-testing (Appendix C, D), skills stations (Appendix E–G), simulation scenarios (Appendix H–L), as time permitted, and didactic lectures (Appendix Q–T). Depending on the number of facilitators and simulation materials (see *Equipment & Environment)*, the learners were divided into groups of three to six people. Each scenario was repeated, using RCDP, for up to 40 minutes. The learners took turns and acted in different roles within each scenario. Day 2 involved completion of the scenarios (Appendix H–L), written multiple-choice post-testing (Appendix A, B) and simulation post-testing (Appendix C, D).

**Topics:**

Respiratory distress, asthma, dehydration, hypovolemic shock, hypoglycemia, seizure, toxic ingestion, newborn resuscitation, precipitous delivery, traumatic injury, EMS, Botswana, global health, collaboration, rapid cycle deliberate practice (RCDP), medical simulation.

**Objectives:**

Medical personnel will gain an understanding of and practice prehospital care with regard to some of the most common pediatric chief complaints.

By the end of this curriculum, the learners will:

1. Increase confidence in their rapid assessment and emergency interventions for common pediatric prehospital response calls.
2. Improve communication and teamwork skills when managing the acutely ill pediatric patient.
3. Develop basic competency in utilizing supplies/resources available in the prehospital setting.
4. Attain an increased proficiency in performing emergency procedures in the acutely ill pediatric prehospital patient.

### Brief introduction

Specialization in prehospital EMS is a relatively new practice in LMIC and having robust training curricula is necessary as these systems develop.^3^–^5^ It has been shown that clinical and hospital-interventions have reduced childhood mortality; however, many EMS systems are still in their infancy and simple prehospital interventions may save lives.^1^ The Botswana MOHW established their EMS program in 2012 in response to the high morbidity and mortality associated with road traffic accidents in the country.^6^ The Botswana MOHW specifically recognized pediatric training as a critical need. Lack of resources, insufficient training, and system deficiencies have shown to be related to poor patient outcomes.^7^ The present curriculum was designed to augment EMS provider training to in turn improve patient outcomes and save lives.

Simulation-based training programs in EMS offer an effective way to develop clinical knowledge, and procedural and communication skills. Simulation provides an opportunity to practice high-risk scenarios in a safe environment. Several studies have demonstrated that simulation-based training can improve prehospital providers’ assessment and clinical management skills, especially for low-frequency and high-risk situations.^8^,^9^,^10^ These simulations were designed based on the most common pediatric response calls. We developed this simulation curriculum based on a needs assessment in the Botswana MOHW EMS system in 2014. The epidemiologic review consisted of approximately 1,500 response calls over a 12-month period in Gaborone, Botswana.

With this information, we developed a pediatric curriculum to provide simulation-based training to the prehospital providers, including nurses, emergency medical technicians (EMTs), and paramedics who care for critically ill children. Particular focus was placed on the technical skills, communication skills, and knowledge required of prehospital providers. The curriculum consists of five pediatric response call scenarios including respiratory distress, hypovolemic shock, newborn delivery, seizures due to a toxic ingestion, and trauma. The scenarios are presented in rapid cycle deliberate practice (RCDP) format, using low-fidelity mannequins. A scoring rubric modified from a simulation team assessment tool was used to test for participant improvement.

The MOHW EMS program identified provider training as a top priority and invited our team to collaborate on the curriculum design and implementation. To design a curriculum relevant to the local context, it is essential to conduct a needs assessment consisting of stakeholders, an inventory of available equipment, a review of existing treatment guidelines, and an epidemiologic review of the population affected by the curriculum, so that it can be designed with the scope of practice and current skills of the EMS system in mind. We utilized these key steps in curriculum development to design a pediatric prehospital training program for EMS systems in LMIC. The objective of this educational project was to design, implement, and evaluate a curriculum relevant to an EMS system based in a LMIC, so that it could be a basis for curricula for use in similar contexts.

### Problem identification, general and targeted needs assessment

A recent study estimates that only 30% of African countries have active EMS programs, and that only 9% of the total population on the continent have access to EMS.^11^ Inadequate EMS systems limit the care that is available to critically ill children. Therefore, it is imperative that as EMS systems develop, they do so within the contexts of available resources and existing health care systems and focus on the local burden of acute diseases. This pediatric curriculum was developed to provide pertinent training on high-frequency response calls received by the Botswana MOHW EMS providers. The curriculum was developed for prehospital providers who have received general basic life support (BLS) training and minimal prehospital healthcare training. At the time of implementation of this program, the Botswana MOHW was primarily comprised of EMTs and nurses of varying healthcare backgrounds. We specifically designed RCDP scenarios for the training, due to the limited experience of the prehospital providers at that time. We utilized a simulation test scenario with an evaluation rubric and adapted pre- and post-test questions from the Pediatric Simulation Training for Emergency Prehospital Providers (PediSTEPPs) program for the written part of the evaluation process.^12^

The curriculum consists of a combination of lectures focused on core pediatric emergency principles, skills stations, and simulation scenarios. The course was given in three separate cities in Botswana (Gaborone, Mahalapye, and Francistown). Some of the didactic components are adapted from a lecture previously created for the PediSTEPPs program by several members of our team (Doughty, Shah),^12^ and the simulation scenarios were all authored by the main instructors.

Prior to commencement of the training, five MOHW EMT’s were trained to be instructors for the course. This training was intended to familiarize these individuals with material content and the specifics of simulation training. These instructors led future training sessions and are responsible for training other trainers in the future as the program grows. Similar models have demonstrated sustainability and successful collaborations between high-income and LMIC.^13^–^16^

Rapid cycle deliberate practice format was selected as the method for instruction because it incorporates multiple repetitions intermixed with feedback. This format is ideal for those with less experience with medical simulation because it provides immediate feedback while practicing high-risk scenarios in a safe learning environment. This method fosters clinical knowledge, procedural skills, confidence, teamwork, and communication, and has been shown to improve key performance measures during resuscitation.^17^

### Goals of the curriculum

Our aim was to create a curriculum based on the needs of the community that could also be implemented in other similar resource-limited settings. The curriculum we developed was focused on high-yield pediatric skills based on the needs of the Botswana MOHW EMS program. Because the results of our needs assessment mirrored common chief complaints in other LMIC countries in Sub-Saharan Africa,^1^,^2^ we feel that this curriculum can be utilized and adopted with minor modifications in other LMIC settings, especially where EMS programs are developing and in circumstances where few EMS providers have had extensive field experience.

### Objectives of the curriculum

Medical personnel will gain an understanding of and practice prehospital care with regard to some of the most common pediatric chief complaints.

By the end of this curriculum, learners will:

1. Increase confidence in their rapid assessment and emergency interventions for common pediatric prehospital response calls.
2. Improve communication and teamwork skills when managing the acutely ill pediatric patient.
3. Develop basic competency in utilizing supplies/resources available in the prehospital setting.
4. Attain an increased proficiency in performing emergency procedures in the acutely ill pediatric prehospital patient.

### Educational Strategies

Please see the separate document of linked objectives and educational goals.

### Equipment & Environment

- Large room with tables or ample floor space, or multiple rooms if available.
- Low fidelity simulation mannequins: with the exception of the neonatal resuscitation scenarios, the use of infant, toddler, or other pediatric mannequins can be adapted based on availability by modifying the age in the scenario. Recommend 3–6 learners per mannequin, 1–2 intravenous (IV) arm task trainers, 1–2 lower extremity intraosseous (IO) task trainers. We trained 12 learners per session and needed 3 mannequins.
- Standard equipment available in the EMS system’s medical bag including IV starter kits, IV fluids, IO needles, medical tape, bag-valve mask, and backboard, etc. (Appendix N)
- Video camera to record the simulation test sessions if planning to evaluate pre- and post-simulation test scoring.

### Results and tips for successful implementation

To start the general training, participants completed a written pre-test and a simulation-based pre-test. Then they began training with skills stations (Appendix A, C, E–G) and didactic lectures (Appendix Q–T). The participants then did the rapid-cycle simulation scenarios (Appendix H–L) which allowed for real-time debriefing and feedback cues in the scenarios. Critical actions and topics for debriefing are listed in each scenario (Appendix O). There was an approximate 3:1 participant to instructor ratio in each group, with each group consisting of no more than 6 people. Participants alternated roles frequently with the multiple cycles of each simulation. Completion of each simulation scenario required approximately 60 minutes per group. An example schedule is provided in Appendix U.

On the second day of training, the course concluded with a written post-test and a simulation post-test. Simulation testing scenarios were developed and used to assess the skills learned during the training. We video recorded these scenarios and later scored their performance.

### Demographic Characteristics

The curriculum was offered three times in three large Botswana cities in 2015. The learners represented convenience samples of off-duty public, prehospital care providers who were invited to participate by the Botswana MOHW. Collectively, 31 learners were involved in the three courses; 19 (61%) were men and 12 (39%) were women. The learners were roughly equally distributed between the three study sites: Francistown (10/41, 32.3%), Mahalapye (10/31 32.3%), and Gaborone (11/31, 35.4%).

### Test Scores

For this initial evaluation, we evaluated the 31 participants in the first 3 training sessions. A paired t-test was conducted to compare the pre- and post-test score differences. There was a statistically significant increase from the written pre-test score (mean=75.3, SD=9.6) to the post-test score (mean=85.9, SD=7.2), p<0.0001, and also from the simulation pretest score (mean= 56.2, SD=13.0) to the post-test score (mean=77.7, SD=8.7), p<0.0001. The mean score for the simulation increased by 22% from the pre- to post-test, while the mean score for the written test increased by 11%.

### Evaluation and Feedback

Feedback was overwhelmingly positive, with 100% of learners reporting the course was either very useful (30%) or extremely useful (70%) on a five-point Likert Scale (Appendix M). We believe simulation training was an excellent and effective method for this type of training, and the RCDP format of the scenarios provided opportunities for frequent feedback leading to immediate performance improvement. Trainees demonstrated retention of knowledge and improved performance in simulation-based testing immediately after the training. When asked what the best part of the course was, many reported that they most enjoyed the simulation portion of the curriculum.

- *“Simulation as they gave real life scenarios that we see every day.”*
- *“Simulation, scenario and giving feedback on how well we performed on scenarios.”*
- *“Assessment of pediatric and infant simulation was interesting, especially the topics on resuscitation of newborns.”*

In regard to feedback, participants primarily requested more time to enable a more extensive curriculum:

- *“The course should be longer (offered over a number of days) because there is a lot of material to cover.”*
- *“Increase duration of training.”*
- *“Increase the time frame to a week. “*
- *“I’d allocate more time to the course so that a lot of materials, including cardiac conditions, can be covered.”*

The overall satisfaction level of the trainees was high and suggests additional training would be beneficial and desired.

- *“The course was so helpful, informative, and I learned new things like managing/dealing with neonates and infants.”*
- *“Thank you for your time and teachings, I think I’m well equipped to manage the patient better than before.”*
- *“Enjoyed the one-on-one contact with the facilitators as we were a small group, we all had turns to practice. [Training of trainers] to do refresher courses yearly.”*

Overall, the results and feedback demonstrated improvement in knowledge and skills of the participants after the two-day course. Since the initial train the trainers course, this curriculum has been taught approximately 25 times throughout Botswana. Though this curriculum was developed based on the specific needs assessments of the Botswana EMS system, we believe that it can be adapted and implemented in other low resource, developing EMS systems in other LMICs. The observed change in test scores and qualitative feedback demonstrates the progress of this curriculum. Given that the course was offered over a two-day time period, with pre and post test scores only a day apart, future endeavors would include re-evaluating long-term retention of this curriculum, as well as application in actual clinical events. One limitation to our curriculum is we did not perform a six-month follow up retention test. Overall, our results show promising improvement in EMS provider skill, confidence, and intervention in various pediatric resuscitation scenarios.

### Associated Content

- Prehospital Pediatric Written Test – Questions
- Prehospital Pediatric Written Test – Answers
- Simulation Testing Scenario Materials
- Simulation Test Scenario Assessment Tool
- Pediatric Skills Station: Airway
- Pediatric Skills Station: Intravenous/Intraosseous/Length-Based Tape
- Pediatric Skills Station: Cardiopulmonary Resuscitation
- Pediatric Simulation Case: Respiratory Distress
- Pediatric Simulation Case: Diarrhea with Hypovolemic Shock
- Pediatric Simulation Case: Newborn Delivery
- Pediatric Simulation Case: Seizure
- Pediatric Simulation Case: Trauma
- Simulation Course for Prehospital Providers Course Evaluation
- Equipment Setup for Simulation Scenarios
- Debriefing Techniques
- Commonly Used Abbreviations
- Lecture 1—Trauma Evaluation
- Lecture 2—Introduction to Simulation
- Lecture 3—Delivery and Neonatal Resuscitation
- Lecture 4—EMS Pediatric Assessment
- EMS Pediatric Training Course Schedule

## Figures and Tables

**Table t2-jetem-6-3-c64:** 

Topic	Recommended Educational Strategy	Educational Content	Objectives	Learners	Timing, Resources Needed (Space, Instructors, Equipment, Citations of JETem pubs or other literature)	Recommended Assessment, Milestones Addressed
Respiratory Distress from Asthma	Rapid Cycle Deliberate Practice (RCDP) Simulation	Identification of a patient with respiratory distress secondary to asthma Initiate stabilization of a patient with respiratory distress	**Cognitive:** Verbalize recognition of respiratory distress in a pediatric patient and correlate exam findings consistent with asthma Know to manage respiratory distress with 100% O2 for hypoxic patients Know to initiate treatment for asthma with bronchodilator (salbutamol/ipratropium) Know to manage severe asthma exacerbation using early delivery of steroids Verbalize progression from respiratory distress to failure, including the need for bag-mask ventilation **Technical**: Be able to obtain IV or IO access Be able to initiate 100% O2 to patient Be able to deliver effective breaths by BVM ventilation **Behavioral:** Assign team roles Use closed-loop communication ○ All messages or orders addressed to specific individuals ○ Team members confirm each request and inform the team leader when a task begins or ends Respect each other with language and behavior ○ Use proper names with eye contact or touch ○ Share ideas and information as suggestions or constructive interventions, not as criticism Provide a shared mental model (summary of scenario and next steps given to maintain situational awareness)	Prehospital providers	Total Time: ~ 60 minutes for multiple rounds of RCDP Setup for All Rounds: Room Configuration: living room of a small home Equipment needed: ○ Standard equipment available in EMS truck and medical bag ○ Pediatric simulation mannequin, IV arm task trainer, lower extremity IO task trainer Personnel: ○ Simulation instructor/debriefer ○ Confederates: medical control (via telephone or radio), another EMS provider	Milestones: Emergency Stabilization, Task-switching, Vascular Access, Team Management Assessment: Critical actions of simulation scenario Evaluation: Real-time feedback during RCDP
Dehydration	Rapid Cycle Deliberate Practice (RCDP) Simulation	Identification of a patient with hypovolemic shock due to dehydration Initiate stabilization of a patient with hypovolemic shock	**Cognitive:** Be able to rapidly assess airway, breathing, and circulation in a child Be able to interpret abnormal vital signs and recognize signs of hypovolemic shock in the child with diarrhea Be able to initiate fluid resuscitation in the child with hypovolemic shock Know the importance of reassessing patients frequently. Continue to resuscitate with fluid boluses when the child continues to have signs of shock on reassessment Be able to recognize and treat hypoglycemia Be able to transport the patient to the hospital while continuing ongoing care **Technical:** Obtain IV/IO access **Behavioral:** Assign team roles Use closed-loop communication ○ All messages or orders addressed to specific individuals ○ Team members confirm each request is understood and inform the leader when a task begins or ends Respect each other with language and behavior ○ Use proper names with eye contact or touch ○ Share ideas and information as suggestions or constructive interventions, not as criticism Provide a shared mental model (summary of scenario and next steps given to maintain situational awareness)	Prehospital providers	Total Time: ~ 60 minutes for multiple rounds of RCDP Setup for All Rounds: Room Configuration: living room of a small home Equipment needed: ○ Standard equipment available in EMS truck and medical bag ○ Pediatric simulation mannequin, IV arm task trainer, lower extremity IO task trainer Personnel: ○ Simulation instructor/debriefer ○ Confederates: medical control (via telephone or radio), another EMS provider	Milestones: Emergency Stabilization, Task-switching, Vascular Access, Team Management Assessment: Critical actions of simulation scenario Evaluation: Real-time feedback during RCDP
Newborn Delivery	Rapid Cycle Deliberate Practice (RCDP) Medical Simulation	Rapid medical assessment to identify precipitous birth Deliver a baby in the field Initiate stabilization of a newborn infant	**Cognitive:** Know to immediately warm/dry/suction/stimulate all infants at deliveries Be able to rapidly identify a non-vigorous infant by looking for color, tone, and respiratory effort. Be able to manage an initially non-vigorous infant with immediate positive pressure ventilation Be able to manage initial bradycardia in a newborn and know when to initiate chest compressions **Technical:** Know the steps for correct umbilical cord cutting/clamping after delivery. Be able to perform adequate positive pressure ventilation (bag-mask ventilation) in a newborn. Be able to perform appropriate chest compressions in a newborn in a 3:1 ratio when bradycardia is not responsive to bag-valve mask ventilation alone **Behavioral:** Assign team roles Use closed-loop communication ○ All messages or orders addressed to specific individuals ○ Team members confirm each request and inform the team leader when a task begins or ends Respect each other with language and behavior ○ Use proper names with eye contact or touch ○ Share ideas and information as suggestions or constructive interventions, not as criticism Provide a shared mental model (summary of scenario and next steps given to maintain situational awareness)	Prehospital providers	Total Time: ~ 60 minutes for multiple rounds of RCDP Setup for All Rounds: Room Configuration: living room of a small home Equipment needed: ○ Standard equipment available in EMS truck and medical bag ○ Infant simulation mannequin, IV arm task trainer, lower extremity IO task trainer Personnel: ○ Simulation instructor/debriefer ○ Confederates: medical control (via telephone or radio), another EMS provider	Milestones: Emergency Stabilization, Task-switching, Vascular Access, Team Management Assessment: Critical actions of simulation scenario Evaluation: Real-time feedback during RCDP
Seizure	Rapid Cycle Deliberate Practice (RCDP) Medical Simulation	Identification of a pediatric patient presenting with a seizure Initiate stabilization of a patient with seizure Understand the various causes of seizure	**Cognitive:** Be able to perform initial ABC management of seizing patient Know to treat the actively seizing patient with benzodiazepine Recognize various causes of seizures including hypoglycemia and toxic ingestion Advanced learners should be able to treat the patient with organophosphate toxicity Anticipate airway compromise in a patient who has received benzodiazepines **Technical:** Be able to manage the airway in a seizing patient **Behavioral:** Assign team roles Use closed-loop communication ○ All messages or orders addressed to specific individuals ○ Team members confirm each request and inform the team leader when a task begins or ends Respect each other with language and behavior ○ Use proper names with eye contact or touch ○ Share ideas and information as suggestions or constructive interventions, not as criticism Provide a shared mental model (summary of scenario and next steps given to maintain situational awareness) Communicate with the receiving facility about an incoming seizure/toxic exposure case.	Prehospital providers	Total Time: ~ 60 minutes for multiple rounds of RCDP Setup for All Rounds: Room Configuration: living room of a small home Equipment needed: ○ Standard equipment available in EMS truck and medical bag ○ Pediatric simulation mannequin, IV arm task trainer, lower extremity IO task trainer Personnel: ○ Simulation instructor/debriefer ○ Confederates: medical control (via telephone or radio), another EMS provider	Milestones: Emergency Stabilization, Task-switching, Vascular Access, Team Management Assessment: Critical actions of simulation scenario Evaluation: Real-time feedback during RCDP
Trauma	Rapid Cycle Deliberate Practice (RCDP) Medical Simulation	Application of a rapid trauma survey Initiate stabilization of a patient with hemorrhagic shock Identify a patient with impaired mental status	**Cognitive:** Be able to perform ITLS primary survey Be able to initiate management of penetrating chest trauma Be able to recognize pediatric hemorrhagic shock and perform early fluid resuscitation Be able to initiate management of extremity trauma Be able to manage continuing assessment of a patient during transport Consider pain management in a trauma patient with IV narcotics **Technical:** Perform a rapid primary assessment Perform basic airway management Immobilize the cervical spine immediately and maintain immobilization throughout evaluation Manage a sucking chest wound by application of an occlusive dressing Administer supplemental oxygen Perform IV/IO line placement Perform a rapid secondary survey Apply a splint **Behavioral:** Assign team roles Use closed-loop communication ○ All messages or orders addressed to specific individuals ○ Team members confirm each request and inform the team leader when a task begins or ends Respect each other with language and behavior ○ Use proper names with eye contact or appropriate touch ○ Share ideas and information as suggestions or constructive interventions, not as criticism Provide a shared mental model (summary of scenario and next steps given to maintain situational awareness) Communicate with the receiving facility about transporting an incoming trauma patient	Prehospital providers	Total Time: ~ 60 minutes for multiple rounds of RCDP Setup for All Rounds: Room Configuration: living room of a small home Equipment needed: ○ Standard equipment available in EMS truck and medical bag ○ Pediatric simulation mannequin, IV arm task trainer, lower extremity IO task trainer Personnel: ○ Simulation instructor/debriefer ○ Confederates: medical control (via telephone or radio), another EMS provider	Milestones: Emergency Stabilization, Task-switching, Vascular Access, Team Management Assessment: Critical actions of simulation scenario Evaluation: Real-time feedback during RCDP
Managing Pediatric Airway	Pediatric Skills Station – Airway	Practice airway positioning, airway clearance, basic and advanced interventions, and delivering oxygen	**Cognitive:** Know the difference between pediatric and adult airways Know proper positioning of patient and how to open the airway via jaw thrust vs head tilt/chin lift Know that infants are obligate nose breathers Know the indications for oral airway placement and proper sizing Recognize need for supplemental oxygen and how to administer **Technical**: Demonstrate appropriate positioning technique Be able to suction with bulb, in-line, and rigid suction device for oral secretions Be able to initiate 100% O2 to patient via nasal canula, non-rebreather mask, and effective breaths by BVM ventilation **Behavioral:** Participants are to demonstrate the hands-on technique of each procedure	Prehospital providers	Total Time: ~ 30 minutes Setup: Room Configuration: living room of a small home Equipment needed: ○ Standard equipment available in EMS truck and medical bag ○ Pediatric simulation mannequin, bulb suction, in-line deep suction device, rigid suction device, oropharyngeal airway, nasopharyngeal airway, supra/extra-glottic device, nasal canula, simple face mask, non-rebreather face mask Personnel: ○ Simulation instructor/debriefer	Milestones: Airway positioning, suctioning, insertion of airway device, application of supplemental O2, proper BMV technique Assessment: Evaluation of hands-on technique Evaluation: Real-time feedback
Obtaining IV/IO access and using Length Based Tape	Pediatric Skills Station – IV/IO/Length-Based Tape	Practice obtaining IV/IO access and understanding how to utilize length-based tape	**Cognitive:** Know indication to obtain IV access Know different sizes of IV catheters in pediatric patients Know indication for rapid IO access **Technical**: Demonstrate appropriate IV access technique Demonstrate proper IO access technique Demonstrate how to use Length-Based tape to estimate weight **Behavioral:** Participants are to demonstrate the hands-on technique of each procedure.	Prehospital providers	Total Time: ~ 30 minutes Setup: Room Configuration: living room of a small home Equipment needed: ○ 20g, 22g, and 24g IV needle and catheter ○ IO needle ○ Length-Based tape Personnel: ○ Simulation instructor/debriefer	Milestones: IV placement, IO placement, proper use of Length-Based tape Assessment: Evaluation of hands-on technique Evaluation: Real-time feedback
Cardiopulmonary Resuscitation	Pediatric Skills Station – CPR	Practice CPR in infant and child	**Cognitive:** Understand the indication for CPR Know adequate depth and rate of chest compressions Know how to perform CPR with 1 vs 2 rescuers **Technical**: Demonstrate appropriate depth and rate of chest compressions technique Use 2 thumb encircling technique or 2 fingers on infant Use one hand technique on child **Behavioral:** Participants are to demonstrate proper CPR technique.	Prehospital providers	Total Time: ~ 30 minutes Setup: Room Configuration: living room of a small home Equipment needed: ○ Pediatric simulation mannequin, infant mannequin Personnel: ○ Simulation instructor/de briefer	Milestones: chest compressions Assessment: Evaluation of hands-on technique Evaluation: Real-time feedback

## DIDACTICS AND HANDS-ON CURRICULUM

[Table t2-jetem-6-3-c64]

## Appendix A Prehospital Pediatric Written Test - Questions

1. Which position best represents the “sniffing” position that allows the best opening of the pediatric airway?
  [Fig f1-jetem-6-3-c64]
2. You pick up a patient from a local school who is on a non-rebreather mask for respiratory distress. What should you do to deliver the highest concentration of oxygen?
  - Nasal cannula with 2 L/min oxygen flow
  - Simple face mask with 15 L/min oxygen flow
  - Non-rebreather face mask with 12 L/min oxygen flow
  - Bag-valve mask 1 cm from face with 15 L/min oxygen flow
3. You arrived at the scene of a home delivery of a newborn infant who is unresponsive. What is the most important initial action to perform for the infant?
  - Providing oxygen
  - Performing chest compressions
  - Ventilating the lungs with a bag-valve mask
  - Clamping and cutting the umbilical cord
4. A 4-year-old female suddenly collapsed at school. She has no pulse on your arrival. You and your partner begin CPR. The proper compression:ventilation ratio for this patient is:
  - 5:1
  - 10:2
  - 15:2
  - 30:2
5. You are called to the home of a 14-month-old male with 3 days of vomiting and diarrhea. His mother reports over 10 episodes of diarrhea daily, and that he has only urinated once in the past 24 hours.
  - Vitals: HR 200 RR 40 BP 68/32 O2 98% on RA Wt 10 kg
  - Exam: Cool extremities. Weak radial pulses.
  Your next intervention should be:
  - Give 500 mL NS IV bolus now
  - Start D5 ½ NS at 40 mL/hr
  - Give 200 mL NS bolus
  - Give 100 mL D5W bolus
6. A 6-month-old male has lethargy and poor feeding. He has had recent vomiting and diarrhea. Your glucose check is 2.1 mmol/L. The child weighs 8 kg. You have a peripheral IV (PIV) in the left hand. What is your best treatment option?
  - D5 NS at 32 mL/hour
  - D50 8 mL IV push
  - D10 40 mL IV push
  - D10 200 mL IV bolus
7. You are called to a delivery of a term infant. You arrive just as the baby is delivered. The baby is not breathing. After 30 seconds of bag-valve mask ventilation, the baby is still not breathing and has a heart rate of 50. You begin CPR at a compression:ventilation ratio of:
  - 30:2
  - 15:2
  - 5:1
  - 3:1
8. You arrive at the home of a 7-year-old child who is having difficulty breathing.
  - Vitals: HR 140 RR 40 BP 92/54 O2 95% on RA Wt 30 kg
  - Exam: Airway open. Lungs with expiratory wheezing bilaterally and subcostal retractions. No heart murmur. Capillary refill 2 seconds.
  Your next step is:
  - Begin bag-valve mask ventilation
  - Administer a normal saline bolus
  - Start oxygen by nasal cannula
  - Administer a nebulized treatment of salbutamol and ipratropium
9. You are called to the home of a 5-year-old male with a seizure disorder who is actively seizing. You have applied 100% oxygen via non-rebreather mask. The respiratory rate is 20 and the patient has a gag reflex. What should you do next?
  - Check a blood glucose
  - Ask about symptoms of infection
  - Assess for signs of trauma
  - Place an oropharyngeal airway
10. A 6-year-old male is found ambulatory at the scene after a road traffic accident and is complaining of neck pain. You note that he has mild midline cervical tenderness, but he has a normal neurologic exam. How should you transport this patient?
  - With a cervical collar and backboard on the stretcher
  - With a cervical collar without a backboard on the stretcher
  - Without a cervical collar and without backboard on the stretcher
  - Without a cervical collar and on a backboard on the stretcher
11. Which of the following is the best indication of successful placement of an intraosseous needle?
  - Fluids can be easily administered without local soft tissue swelling
  - The hub of the needle is touching the skin
  - You are able to aspirate blood from the needle
  - Pulsatile blood flow is present in the needle hub
12. A 5-year-old male is actively seizing when you arrive on the scene. A finger stick blood glucose is 4.0 mmol/L (72 g/dL). His airway is open. What should your next step be?
  - Give 25% dextrose, 4 mL/kg, IO
  - Give 10% dextrose, 5 mL/kg IV
  - Midazolam 0.2 mg/kg IM
  - Midazolam 2 mg/kg IV
13. You arrive at the home of a 9-year-old boy who fell from a second story window and has an obvious deformity to his left arm. The first step in your assessment of this child is to:
  - Splint his left arm
  - Check his blood glucose level
  - Assess airway, breathing, circulation, and disability
  - Assess his mental status
14. EMS is called to the scene of an unresponsive 2-year-old female. The child was previously healthy and the family witnessed the patient suddenly collapse. The first step in your assessment is to:
  - Reposition the patient’s airway
  - Check the patient’s pulse
  - Check for breathing
  - Check a blood glucose
15. You arrive at the scene of a road traffic accident and find a 10-year-old male in respiratory distress and an open wound on the right side of his chest. You notice air bubbles at the wound site as the patient exhales. Your management of this wound should be:
  - Leave the wound open
  - Pack the wound with gauze dressing
  - Using Vaseline gauze, cover the wound and tape on three sides
  - Initiate bag-mask ventilation

## Appendix B Prehospital Pediatric Written Test - Answers

1. Which position best represents the “sniffing” position that allows the best opening of the pediatric airway?
  [Fig f2-jetem-6-3-c64]
2. You pick up a patient from a local school who is on a non-rebreather mask for respiratory distress. What should you do to deliver the highest concentration of oxygen?
  - Nasal cannula with 2 L/min oxygen flow
  - Simple face mask with 15 L/min oxygen flow
  - **Non-rebreather face mask with 12 L/min oxygen flow**
  - Bag-valve mask 1 cm from face with 15 L/min oxygen flow
3. You arrived at the scene of a home delivery of a newborn infant who is unresponsive. What is the most important initial action to perform for the infant?
  - Providing oxygen
  - Performing chest compressions
  - **Ventilating the lungs with a bag-valve mask**
  - Clamping and cutting the umbilical cord
4. A 4-year-old female suddenly collapsed at school. She has no pulse on your arrival. You and your partner begin CPR. The proper compression:ventilation ratio for this patient is:
  - 5:1
  - 10:2
  - **15:2**
  - 30:2
5. You are called to the home of a 14-month-old male with 3 days of vomiting and diarrhea. His mother reports over 10 episodes of diarrhea daily, and that he has only urinated once in the past 24 hours.
  - Vitals: HR 200 RR 40 BP 68/32 O2 98% on RA Wt 10 kg
  - Exam: Cool extremities. Weak radial pulses.
  Your next intervention should be:
  - Give 500 mL NS IV bolus now
  - Start D5 ½ NS at 40 mL/hr
  - **Give 200 mL NS bolus**
  - Give 100 mL D5W bolus
6. A 6-month-old male has lethargy and poor feeding. He has had recent vomiting and diarrhea. Your glucose check is 2.1 mmol/L. The child weighs 8 kg. You have a peripheral IV (PIV) in the left hand. What is your best treatment option?
  - D5 NS at 32 mL/hour
  - D50 8 mL IV push
  - **D10 40 mL IV push**
  - D10 200 mL IV bolus
7. You are called to a delivery of a term infant. You arrive just as the baby is delivered. The baby is not breathing. After 30 seconds of bag-valve mask ventilation, the baby is still not breathing and has a heart rate of 50. You begin CPR at a compression:ventilation ratio of:
  - 30:2
  - 15:2
  - 5:1
  - **3:1**
8. You arrive at the home of a 7-year-old child who is having difficulty breathing.
  - Vitals: HR 140 RR 40 BP 92/54 O2 95% on RA Wt 30 kg
  - Exam: Airway open. Lungs with expiratory wheezing bilaterally and subcostal retractions. No heart murmur. Capillary refill 2 seconds.
  Your next step is:
  - Begin bag-valve mask ventilation
  - Administer a normal saline bolus
  - Start oxygen by nasal cannula
  - **Administer a nebulized treatment of salbutamol and ipratropium**
9. You are called to the home of a 5-year-old male with a seizure disorder who is actively seizing. You have applied 100% oxygen via non-rebreather mask. The respiratory rate is 20 and the patient has a gag reflex. What should you do next?
  - **Check a blood glucose**
  - Ask about symptoms of infection
  - Assess for signs of trauma
  - Place an oropharyngeal airway
10. A 6-year-old male is found ambulatory at the scene after a road traffic accident and is complaining of neck pain. You note that he has mild midline cervical tenderness, but he has a normal neurologic exam. How should you transport this patient?
  - **With a cervical collar and backboard on the stretcher**
  - With a cervical collar without a backboard on the stretcher
  - Without a cervical collar and without backboard on the stretcher
  - Without a cervical collar and on a backboard on the stretcher
11. Which of the following is the best indication of successful placement of an intraosseous needle?
  - **Fluids can be easily administered without local soft tissue swelling**
  - The hub of the needle is touching the skin
  - You are able to aspirate blood from the needle
  - Pulsatile blood flow is present in the needle hub
12. A 5-year-old male is actively seizing when you arrive on the scene. A finger stick blood glucose is 4.0 mmol/L (72 g/dL). His airway is open. What should your next step be?
  - Give 25% dextrose, 4 mL/kg, IO
  - Give 10% dextrose, 5 mL/kg IV
  - **Midazolam 0.2 mg/kg IM**
  - Midazolam 2 mg/kg IV
13. You arrive at the home of a 9-year-old boy who fell from a second story window and has an obvious deformity to his left arm. The first step in your assessment of this child is to:
  - Splint his left arm
  - Check his blood glucose level
  - **Assess airway, breathing, circulation, and disability**
  - Assess his mental status
14. EMS is called to the scene of an unresponsive 2-year-old female. The child was previously healthy and the family witnessed the patient suddenly collapse. The first step in your assessment is to:
  - Reposition the patient’s airway
  - **Check the patient’s pulse**
  - Check for breathing
  - Check a blood glucose
15. You arrive at the scene of a road traffic accident and find a 10-year-old male in respiratory distress and an open wound on the right side of his chest. You notice air bubbles at the wound site as the patient exhales. Your management of this wound should be:
  - Leave the wound open
  - Pack the wound with gauze dressing
  - **Using Vaseline gauze, cover the wound and tape on three sides**
  - Initiate bag-mask ventilation

### DIDACTICS AND HANDS-ON CURRICULUM INSTRUCTOR MATERIALS

*Simulation Testing Scenario*

## Appendix C Simulation Testing Scenario Materials

### Pediatric Simulation Pretest Case

#### Instructions for Candidates

During the next examination you will evaluate an emergency department patient. You will be the team leader. You will have additional team members to help you, but you will make all decisions related to the case.

The patient you encounter for this exercise is not a real patient but a SIMULATED PATIENT. For the purpose of this evaluation, the Training Instructors prepared a mannequin and a volunteer parent to act and respond to you and your care decisions as close to a real patient encounter as you might experience in the casualty ward. You can obtain all needed information from the simulated patient, as you would from a real patient.

Obtain any missing vital signs and demonstrate how you would perform them, ask for any additional information about the patient (signs and symptoms), and perform any necessary focused physical examinations based on the algorithms provided to you for the training.

Based on the patient’s chief complaint, vital signs, history, and physical exam findings, obtain any laboratory or perform medical interventions (including giving intravenous fluids and/or medications) based on the algorithm, by informing the Course Instructor of your request.

If you decide to administer medications, let the Course Instructor know what medication or treatment (eg, oxygen or medications) you would like to administer. Remember, you will also need to provide the course instructor with the correct dosing for any intravenous fluids and medications you give. You cannot administer any actual medications to the mannequin, but you can apply oxygen and perform procedures such as bag-mask ventilation, intubation, and CPR if you would perform these in real life. Your orders for medications or fluids should be reported to the examiner.

All information collected during this examination will be used for evaluation of the current training program. The specifics of your examination will be kept anonymous and not connected to your name. This information will not be given to anyone else but the course evaluation team. There will be no personal repercussions to you or anyone else if your performance is imperfect.

**Beginning of Case:**
You are called to the home of a 2-month-old female with difficulty breathing. The mother tells you that her daughter began to have some cough and fast breathing a few days ago. Today, the child has been breathing very fast and the mother tells you it looks like it is hard for her to breathe. She is no longer able to breastfeed today. The child has also felt hot at home. On your exam, the child has her eyes open but is very quiet aside from briefly crying when you approach her. She is breathing very fast at 70 breaths per minute, and has subcostal retractions, head bobbing, and nasal flaring.

#### Instructions for Examiner

Each candidate will be provided with the problem and chief complaint (difficulty breathing).

The candidate will also be provided with a short clinical vignette consisting of patient’s sex, age, and duration of problem. All information provided in the vignette will be presented clearly in order to directly lead the candidate to the simulated health problem or diagnosis.

All vital signs and exam findings aside from those in the vignette will need to be requested by the candidate in order to simulate the patient encounter.

The vignette for this case is below.

**Beginning of Case:**
You are called to the home of a 2-month-old female with difficulty breathing. The mother tells you that her daughter began to have some cough and fast breathing a few days ago. Today, the child has been breathing very fast and the mother tells you it looks like it is hard for her to breathe. She is no longer able to breastfeed today. The child has also felt hot at home. On your exam, the child has her eyes open but is very quiet aside from briefly crying when you approach her. She is breathing very fast at 70 breaths per minute and has subcostal retractions, head bobbing, and nasal flaring.

#### Additional Case Information for the Examiner

This patient presents with respiratory distress due to pneumonia.

AIRWAY/BREATHING: She is tachypneic and hypoxic with retractions. Her oxygen will briefly improve with administration of oxygen, but she will have worsening work of breathing and progress to respiratory failure due to the severity of her disease. She will eventually require ventilation with BVM.

CIRCULATION: The patient is tachycardic with dry mucous membranes and few tears; she has delayed capillary refill and weak pulses. She will require IV fluids to improve circulation.

#### Progression of Case for Examiners

**Initial Assessment**

**Vitals:** RR 70, HR 195, BP 66/32, O2 75% on RA, T 39.6°C, Weight 5 kg

**Exam:** Airway--open. Breathing with subcostal retractions, head-bobbing, nasal flaring. Lungs with crackles and decreased breath sounds to the entire right lung, with no breath sounds in the right base. Good breath sounds in other lung fields. Circulation with 1+ peripheral pulses, tachycardic, no murmur, cool extremities with CR 4 seconds. Mental status alert, briefly cries appropriately to painful stimuli, but overall quiet. Secondary survey--Dry mucous membranes, few tears, soft abdomen, no rashes, no bruising. If asked for an exam finding not above, the finding should be reported to the student as normal.

**Team will have a chance to complete general assessment, check vitals, initiate O2, obtain IV access. Glucose is immediately available if requested and is 91. After this time, team should ask for reassessment. If they do not, offer that child appears to be less alert, struggling to breathe, and her oxygen level is now 70%**.

**Reassessment #1**

**Vitals:** RR 40, HR 200, BP 66/32, O2 70% on O2

**Exam:** Airway--open. Breathing with subcostal retractions, head-bobbing, nasal flaring, grunting. Lungs with crackles and decreased breath sounds to the entire right lung, with no breath sounds in the right base. Good breath sounds in other lung fields. Circulation with 1+ peripheral pulses, tachycardic, no murmur, cool extremities, CR 4 seconds. Mental status lethargic, briefly opens eyes to pain only. Secondary survey- Dry mucous membranes, few tears, soft abdomen, no rashes, no bruising. If asked for an exam finding not above, the finding should be reported to the student as normal.

Team may initially try to increase O2 but saturations will increase to 83% and not higher. Work of breathing will not improve. Pt will require bag-valve-mask ventilation. Saturations will improve to 100% with this if correctly done. Saturations will not improve if correct technique is not used. If ventilation is stopped, patient will desaturate.

Patient is also hypotensive. NS bolus should be given to improve circulation. If one bolus is given, BP will improve to 72/43 but patient will remain tachycardic at 190 with CR 3–4 seconds. If second bolus is given, BP will improve to 80/50 with HR 180 and CR 2–3 seconds.

Team should then prepare to transport patient to hospital.

*Simulation Testing Scenario Assessment Tool*

## Appendix D Simulation Testing Scenario Assessment Tool

[Table t3-jetem-6-3-c64] [Table t4-jetem-6-3-c64] [Table t5-jetem-6-3-c64] [Table t6-jetem-6-3-c64] [Table t7-jetem-6-3-c64]

**Table t3-jetem-6-3-c64:**

**Modified Simulation Team Assessment Tool (STAT)**	
Date: __________________________	Participants:________________________
Time: __________________________	__________________________________
Scenario: _______________________	__________________________________

**Table t4-jetem-6-3-c64:**

Basics					
Task Group	Task	Complete & Timely	Incomplete or Untimely	Needed and Not Done	Not Required
**History & Physical**	Obtain SAMPLE history (sign/symptoms, allergies, meds, past illness, last meal, events preceding)	2	1	0	N/A
	Performs primary survey (ABCDE)	2	1	0	N/A
	Performs secondary survey (head to toe, including back)	2	1	0	N/A
**Patient Weight**	Estimates/obtains patient weight	2	1	0	N/A
**Monitors**	Ensures cardiorespiratory and O2 monitors placed/vitals taken	2	1	0	N/A
**Access**	Obtains or confirms vascular access	2	1	0	N/A
	Attempts IO access	2	1	0	N/A
**Labs**	Orders appropriate lab testing	2	1	0	N/A
	Responds to lab results appropriately	2	1	0	N/A
					
**Recognition**	Recognizes urgent/emergent situation (either at beginning of scenario or with decompensation)	2	1	0	N/A
**Universal Precautions**	Team uses appropriate universal precautions	2	1	0	N/A
**Consults**	Contacts other EMS team for appropriate support	2	1	0	N/A
**Family**	Directs updates to family	2	1	0	N/A
					

**Table t5-jetem-6-3-c64:**

Airway & Breathing					
Task Group	Task	Complete & Timely	Incomplete or Untimely	Needed and Not Done	Not Required
					
**Assessment**	Assesses airway	2	1	0	N/A
	Assesses breathing	2	1	0	N/A
**Basic Intervention**	Performs airway maneuvers	2	1	0	N/A
	Provides supplemental oxygen	2	1	0	N/A
	Uses appropriate adjunct airway	2	1	0	N/A
**Bag-mask ventilation**	Initiated BVM	2	1	0	N/A
	Bag at appropriate rate	2	1	0	N/A
	Assesses chest rise	2	1	0	N/A
	Uses proper BVM technique and positioning	2	1	0	N/A

**Table t6-jetem-6-3-c64:**

Circulation					
Task Group	Task	Complete & Timely	Incomplete or Untimely	Needed and Not Done	Not Required
**Basics**	Assesses heart rate	2	1	0	N/A
	Assesses pulses	2	1	0	N/A
	Assessesz blood pressure	2	1	0	N/A
	Assesses distal perfusion (cap refill)	2	1	0	N/A
**Management**	Initiates volume resuscitation	2	1	0	N/A
	Selects isotonic fluid	2	1	0	N/A
	Initiates appropriate IV fluid dose	2	1	0	N/A
	Ongoing fluid resuscitation as needed	2	1	0	N/A
**CPR**	Correct hand placement	2	1	0	N/A
	Correct rate of compressions	2	1	0	N/A
	Use appropriate surface (backboard, floor)	2	1	0	N/A
	Uses appropriate ratio of ventilations: compressions	2	1	0	N/A
	Assess quality of CPR (pulse check during compressions)	2	1	0	N/A
	Minimize interruptions in CPR	2	1	0	N/A
	Medications (gives adrenaline appropriately)	2	1	0	N/A
	Pulse/rhythm check after 2 minutes	2	1	0	N/A
**Airway adjuncts**	Appropriate assessment of need for airway adjunct	2	1	0	N/A
	Able to select correctly the size of airway adjunct needed	2	1	0	N/
	Correct placement of nasal trumpet	2	1	0	N/A
	Correct placement of oral airway	2	1	0	N/A

**Table t7-jetem-6-3-c64:**

Team Management					
Task Group	Task	Complete & Timely	Incomplete or Untimely	Needed and Not Done	Not Required
**Team**	All team members exhibit professional attitude and interactions	2	1	0	N/A
**Leadership (Team Leader)**	There is a clearly identified team leader	2	1	0	N/A
	Assigns roles to team members	2	1	0	N/A
	Maximizes skill set of personnel in assigned roles	2	1	0	N/A
	Directs/redirects team members appropriately	2	1	0	N/A
	Monitors actions of team members	2	1	0	N/A
	Addresses specific persons when requesting info/assigning tasks	2	1	0	N/A
	Uses closed-loop communication (orders directed & confirmed)	2	1	0	N/A
	Resolves conflicts	2	1	0	N/A
	Engages team members in decision-making	2	1	0	N/A
	Recruits additional personnel when appropriate	2	1	0	N/A
	Maintains global view (does not get sidetracked by procedures, details)	2	1	0	N/A
	Performs tasks in appropriate sequence/prioritizes well	2	1	0	N/A
	Reprioritizes for urgent/emergent events	2	1	0	N/A
	Avoids fixation errors (considers full differential for problems encountered)	2	1	0	N/A
	Performs interim summary/assessment for team coordination	2	1	0	N/A
	Summarizes case for transfer of care	2	1	0	N/A
	Work-load balancing	2	1	0	N/A
**Management (Team Members)**	Carry out tasks in appropriate sequence	2	1	0	N/A
	Stay in roles, appropriately	2	1	0	N/A
	Adjust roles to address urgent events, appropriately	2	1	0	N/A
	Verbalize questions/info to team leader	2	1	0	N/A
	Use closed loop communication (confirm order, task completion)	2	1	0	N/A
	Ask for assistance if unable to complete task/balance workload	2	1	0	N/A
	Engage in decision making	2	1	0	N/A
	Suggest additional resources (personnel, etc. appropriately	2	1	0	N/A

## Appendix E SKILLS STATION—Airway

### PRACTICE

- **Airway positioning (chin lift, jaw thrust)**
- **Airway clearance (suctioning with bulb, in-line,** **rigid suction device for oral secretions)**
- **Basic airway interventions (oropharyngeal, nasopharyngeal airways)**
- **Oxygen delivery (nasal cannula, simple face mask, non-rebreather mask)**
- **Basic intervention for respiratory failure (bag-mask ventilation)**
- **Advanced intervention for respiratory failure (blind insertion of supra/extra-glottic device)**

**When teaching skills stations, it is important to provide a** **simple case scenario**, **have** **participants demonstrate** **the hands-on technique of how they would do each procedure, and for the** **instructor to correct inappropriate techniques**.

**Case scenario:** You arrive on scene for a 6-month-old infant (or can replace with a 30-year-old man) in respiratory distress. The patient has a normal pulse for age. Airway assessment shows secretions in the nose and mouth. The patient is slightly tachypneic for age with an oxygen saturation of 80% on room air. (As the case progresses, the patient becomes apneic requiring bag-mask ventilation).

- Airway maneuvers
  ○ **Position:** The ideal position for ventilation is the “sniffing position” (neither hyperextended or hyperflexed). The pediatric airway can become obstructed when supine due to the large occiput, the floppy neck, and small airway size relative to the head. It is vital to remember airway alignment when ventilating or immobilizing an infant or child.
  ○ **Padding:** Pad the shoulders to place children in the sniffing position.
  ○ **Open the airway:** This can be accomplished with a few simple maneuvers. The **jaw thrust** is done by placing a hand to each side of the head (as if you were doing inline stabilization of the C-spine) and then pressing upward at the angle of the mandible thus sliding the jaw forward. The **head tilt/chin lift**: This is done by tilting the head posteriorly and placing the fingers under the chin and lifting upwards. Both maneuvers will lift the tongue off the back of the airway. The head tilt/chin lift should not be done when there has been potential neck trauma.
- Airway clearance
  ○ **Bulb Suction:** Use your bulb suction device to clear the nose in infants. Normal saline can be used by applying several drops in each nare and then attempting suctioning. The purpose of the saline is to break up the mucus to enable easier and more effective suctioning.
  ○ **Inline deep suctioning**: Use saline drops as discussed above and then choose a suction catheter that is small enough to fit in the nostril. Feed the catheter straight back towards the occiput, posterior to the tongue. This can go deep enough to clear any secretions in the posterior pharynx near the glottic opening. Be prepared for gagging and vomiting. You may need to quickly turn the head to the side for vomiting. Be gentle with the catheter, as this may cause bleeding. Suction on the way out.
    ▪ Bronchiolitis (lower airway) infection--Small children and infants cannot cough up phlegm or blow their nose like adults. Breathing may be artificially disordered due to a plugged-up airway and suctioning alone may improve the respiratory work of breathing. Bronchiolitis is usually seen in children less than 2 years old.
    ▪ O2 saturation may drop initially after suctioning. Use supplemental oxygen as needed.
    ▪ To help avoid bleeding, a lubricant can be used.
  ○ **Rigid suction device****:** This is used for vomitus vs soft flexible in line suctioning device may help with chunks of food particles.
- Discuss the differences in pediatric vs. adult airways
  ○ **Large occiput and floppy neck:** The back part of the skull is relatively large and protrudes (sticks out) in children. In conjunction with a short and floppy neck, the airway is easily occluded. Think of a 5cm piece of oxygen tubing…place a coconut on one end and a 10lb sandbag on the other. The tube is like a pediatric airway.
  ○ **Nose breathers:** Infants are obligate nose breathers. When their nose is occluded (by secretions, etc.) they will have difficulty breathing.
  ○ **Large tongue**
- Oral airway placement
  ○ **Indications:** An oral airway should be placed in a person who has altered mental status and no gag reflex, and who needs airway maneuvers to open the airway. The oral airway is a useful adjunct to bag-mask ventilation.
  ○ **Contraindications:** If there is a gag reflex upon placement, remove the oral airway immediately because this may induce vomiting and potentially cause aspiration into the lungs. Awake patients will not tolerate oral airway placement.
  ○ **Sizing:** Measure from the **lip to earlobe** to find the appropriate oral airway size for your patient. Oral airways that are not properly sized can actually cause more airway obstruction.
  ○ **Method of insertion:** The airway can be placed by using a scissoring motion of the right hand while placing with the left. It should go in upside down and then be rotated into place. Take care not to scrape the roof of the mouth to cause bleeding. If the mouth cannot be opened wide, you can place it by just following the curve of the tongue. With this method, you must be careful to ensure that the tip of the oral airway reaches the base of the tongue and does not cause obstruction.
- Nasopharyngeal airway placement
  ○ **Indications:** A nasopharyngeal airway may be placed in a person with signs of nasopharyngeal airway obstruction, such as snoring. The nasopharyngeal airway can also be a useful adjunct to bag-mask ventilation. It may be placed whether or not a gag reflex is present.
  ○ **Contraindications:** A nasopharyngeal airway should not be placed in a patient with a suspected midface or basilar skull fracture; signs of this may be bloody or clear drainage from the nose or periorbital or mastoid bruising.
  ○ **Sizing:** Measure from the **nostril to earlobe** to find the appropriate nasopharyngeal airway size for your patient.
  ○ **Method of insertion:** The airway can be placed by inserting it into one nostril and slightly twisting it from side to side, as you push it directly posteriorly. Do not point it inferiorly or superiorly. If secretions are absent in the nostril, using a lubricant gel may be necessary to easily pass it.
- Oxygen delivery
  ○ The fraction of inspired oxygen (FiO2) for room air is 21%. Supplemental oxygen adds approximately 3% FiO2 per liter to the 21% in room air.
  ○ **Nasal Cannula:** Nasal cannulas work well for infants and small children since they are nose breathers. Flow should be 0.5–4 liters/minute and deliver a maximum of approximately 30–40% FiO2.
  ○ **Simple Face Mask:** Does not have an oxygen reservoir and allows rebreathing of exhaled carbon dioxide. Flow should be 5–10 liters/minute and delivers a maximum of approximately 50–60% FiO2.
  ○ **Non-Rebreather:** Does have an oxygen reservoir and minimizes rebreathing of exhaled carbon dioxide. Flow should be 10–15 liters/minute and delivers a maximum of approximately 80–90% FiO2.
- Bag-valve mask ventilation
  ○ **Indications:** Respiratory failure (slow or absent breathing). Fast breathing is not an indication to provide bag-mask ventilation unless there are other signs of respiratory failure that have not resolved with the above-noted interventions, such as hypoxia or altered mental status.
  ○ **Appropriate size of mask:** The correct size mask covers the mouth and nose and does not interfere with the eyes. Correct sizing of the mask will make it much easier to get a good seal, without air leak. Incorrect sizing may lead to trauma to the eyes, and inability to adequately bag-valve mask the patient.
  ○ **Do NOT press down into the face, as the airway will obstruct. Instead, draw the face into the mask**. Essentially you are performing a chin lift to the mask. Use the C-E grip with the left hand with left fingers (middle/ring/pinky) placed on the jaw, with the thumb and index finger on top of the mask. In infants and small children, be careful that your fingers do not press on the neck or soft tissue, because this can cause airway obstruction. It may be necessary to have one person hold the mask, while another provides bag-mask ventilation in order to ensure a good seal.
  ○ **Small puffs:** Over-ventilation will cause the stomach to inflate and then induce vomiting. Only ventilate enough to see chest rise and auscultate air entry in the lungs. The chest should not be heaving and it should be a smooth motion.
  ○ **Respiratory rate:** Children breathe faster than adults and should receive more ventilation than adults. However, we all have a tendency to bag too fast in emergency situations, which can be harmful.
- Blind insertion of supra/extra-glottic device
  ○ **What are these devices?** These are airways that can be inserted into the mouth with direct visualization of the vocal cords, in order to provide ventilation to the patient. These are not definitive airways, so patient still have a risk for aspiration if they were to vomit when one is in place. Some of the supra/extra-glottic devices do not require inflation; other devices have balloons that need to be inflated after insertion.
  ○ **Indications:** Basic and advanced emergency medical technicians should consider placing a supra/extra-glottic device in the setting of respiratory failure when bag-mask ventilation does not seem to be providing effective oxygenation and/or ventilation for the patient, or if the transport time is long and making ongoing bagmask ventilation difficult.
  ○ **Contraindications:** The presence of a gag reflex.
  ○ **Sizing:** Neonates through adults have supra/extra-glottic devices sizes available. The size of the device is based on weight, which can be estimated with the length-based tape to estimate weight and is available on the side of the package and device itself.
  ○ **Method of Insertion:** After opening the mouth, place the soft fell or balloon (uninflated) portion of the supra/extra-glottic devices past the tongue and push the device into the pharynx until it cannot be pushed further. If the supra/extra-glottic device has a balloon, inflate it once it is inserted. Then attach a ventilation bag to provide breaths. In case the oral cavity is dry, the use of a gel lubricant may be necessary.

## Appendix F SKILLS STATION—IV/IO/Length-Based Tape

### PRACTICE

- **Obtaining intravenous (IV) access**
- **Obtaining intraosseous (IO)access**
- **Estimating weight using length-based tape**

**When teaching skills stations, it is important to provide a** **simple case scenario**, **have** **participants demonstrate** **the hands-on technique of how they would do each procedure, and for the** **instructor to correct inappropriate techniques**.

**Case scenario:** You arrive on scene for a 2-year-old child in shock. Airway/breathing are intact. What will you do about the signs of poor perfusion?

- IV access
  ○ **Sizes:** Children: 20g, 22g, 24g--The easiest site in children may be the hand. It is helpful in infants especially to hold the hand and wrist in an extreme flexed position demonstrating this position. This is a different technique than what is used in adults, where the antecubital fossa is often best. Other sites for IV access include the foot and ankle.
  ○ **Unable to place IV:** If IV placement is unsuccessful, think about other routes for medications (intranasal, intramuscular, or intraosseous).
- IO access
  ○ **Use:** Rapid IO access is essential if an IV cannot be placed in the setting of cardiac arrest, shock, hypoglycemia, respiratory failure, unresponsiveness, or seizures not responding to rectal (PR) diazepam. If an actual IO needle is not available, use a large bore needle (14g, 16g, 18g) instead.
  ○ **Site:** The easiest insertion site is in the tibia, 1–2 fingers inferior/medial to the tibial tuberosity (below the patella) on the flattest portion of the tibia. Do not place through a broken bone or infected skin. DO NOT place in the joint itself. Other sites that can be used are the distal/medial tibia, the anterior/distal femur and the proximal/lateral humerus.
  ○ 90-degree insertion to the bone
  ○ Do not continue with fluids or medications if there is swelling
  ○ Should not be loose when properly placed in the bone
  ○ IO should be secured, so it does not become dislodged en route
  ○ Aspiration of blood or bone marrow is not always possible
- Length-based tapes
  ○ **Use:** If available, it is best to use a length-based tape rather than parental report to estimate the weight of a child to determine medication dosing and equipment size
  ○ **Technique:** Unfold the tape starting with the arrow at the patient’s head and extending it to the feet, with the patient supine with legs outstretched.
  ○ **Caution:** The weight may be overestimated in malnourished (wasted or edematous) children, so fluid and medications might also be overestimated.

## Appendix G SKILLS STATION—Cardiopulmonary Resuscitation

### HIGH QUALITY CPR

- Adequate depth is at least 1/3 the anterior posterior diameter of the chest
- Compression rate is at least 100 per minute
- Allow for adequate recoil of the chest
- Place patient on a hard surface
- Position self directly above patient-shoulder should be directly over hand

### RATIOS AND POSITIONING

- Infant 2-person rescuer ratio: 15 compressions: 2 breaths. Use 2 thumbs encircling or 2 fingers.
- Child 2-person rescuer ratio: 15 compressions: 2 breaths. Use 1 hand to compress.
- Newborn does not need to be covered in this station, but ratio is 3 compressions: 1 breath

### PRACTICE

- Cardiopulmonary resuscitation in the infant (non-neonate)
- CPR in the child, 1 or 2 rescuers
- CPR in the adult, 1 or 2 rescuers

**When teaching skills stations, it is important to provide a** **simple case scenario**, **have** **participants demonstrate** **the hands-on technique of how they would do each procedure, and for the** **instructor to correct inappropriate techniques****. Have participants practice initiating CPR and coordinating pulse check and switches**.

*Pediatric Simulation Case: Respiratory Distress from Asthma*

## Appendix H Pediatric Simulation Case: Respiratory Distress from Asthma

**Learner Audience:**
Prehospital providers including emergency medical technicians, registered nurses, respiratory therapists, and physicians at any level of training.
**Time Required for Implementation:**
~ 60 minutes for multiple rounds of RCDP
**Recommended Number of Instructors:**

- 1 simulation instructor/debriefing facilitator per group of three to six learners
- 1 confederate/assistant per group of three to six learners

**Objectives:**
By the end of the session, learners should be able to: Cognitive:

1. Verbalize recognition of respiratory distress in a pediatric patient and correlate exam findings consistent with asthma
2. Know to manage respiratory distress with 100% O2 for hypoxic patients
3. Know to initiate treatment for asthma with bronchodilator (salbutamol/ipratropium)
4. Know to manage severe asthma exacerbation using early delivery of steroids
5. Verbalize progression from respiratory distress to failure, including the need for bag-mask ventilation

Technical:

1. Be able to obtain IV or IO access
2. Be able to initiate 100% O2 to patient
3. Be able to deliver effective breaths by BVM ventilation

Behavioral:

1. Assign team roles
2. Use closed-loop communication
  ○ All messages or orders addressed to specific individuals
  ○ Team members confirm each request and inform the team leader when a task begins or ends
3. Respect each other with language and behavior
  ○ Use proper names with eye contact or touch
  ○ Share ideas and information as suggestions or constructive interventions, not as criticism
4. Provide a shared mental model (summary of scenario and next steps given to maintain situational awareness)

**Case Title:** Respiratory Distress from Asthma

**Equipment or Props Needed:**

Setup for All Rounds:

- Pediatric mannequin
- Equipment to be brought into the scenario by the prehospital provider team. In our trials, this included:
  ○ Monitors including blood pressure cuff and oxygen saturation monitor
  ○ Airway equipment including suction catheter, stethoscope, oral pharyngeal airway, nasopharyngeal airway, BVM with different sizes of masks, nasal cannula, simple face mask, non-rebreather face mask
  ○ Intravenous (IV) access equipment including various sizes of pediatric IV cannulas, 18g needle or intraosseous (IO) needle, IV tubing
  ○ IV fluids including normal saline or Ringer’s lactate, dextrose containing fluid (D50, D25, D10, D5 if available)
  ○ Glucometer
  ○ Length-based tape to estimate weight
  ○ Splinting material
  ○ Cervical collar (C-collar) and backboard

**Common verbal prompt to start all rounds:** “EMS response called to a patient’s home. The patient is a 3-year-old female in respiratory distress for 3 days. The mother found her with worsening breathing and cough for the past 3 days.”

**Other history (if asked):** Patient has had 2–3 wheezing episodes over the last 2–3 months. Strong family history of atopy and asthma. **Initial vital signs for all rounds:** T 37.0°C, HR 160, RR 50, BP 87/43, O2 70% on room air Weight per Broselow tape

Initial physical exam for all rounds:

AIRWAY**:** airway open
BREATHING: subcostal retractions, nasal flaring, diffuse wheezing heard on auscultation of bilateral lung fields.
CIRCULATION**:** strong pulses, capillary refill (CR) 2–3 seconds
DISABILITY**:** alert
OTHER: child has cyanosis to the mouth and is in distress. Otherwise assume other exam findings are normal.

### Round 1: Initial assessment of respiratory distress

**Objectives introduced this round:**

1. Verbalize recognition of respiratory distress in a pediatric patient and correlate exam findings consistent with asthma
2. Know to manage respiratory distress with 100% O2 for hypoxic patients
3. Be able to obtain IV or IO access

**Verbal prompt:** “EMS response call to a patient’s home. The patient is a 3-year-old female who presents with respiratory distress for 3 days. The mom found her with worsening breathing and cough for the past 3 days.”

**Other history (if asked):** Patient has had 2–3 wheezing episodes over the last 2–3 months. Strong family history of atopy and asthma.

**Initial vital signs:** T 37.0 °C, HR 160, RR 50, BP 87/43, O2 70% on room air Weight per Broselow tape

**Initial physical exam**

AIRWAY: airway open
BREATHING: subcostal retractions, nasal flaring, diffuse wheezing heard on auscultation of bilateral lung fields
CIRCULATION: strong pulses, CR 2–3 seconds
DISABILITY: alert
OTHER: child has cyanosis to the mouth and is in distress. Otherwise assume other exam findings are normal

**Expected actions after verbal prompt**

- Rapid assessment of ABC (= Airway, Breathing, Circulation)
- Team member should check complete set of vital signs
- Team should recognize signs of respiratory distress and verbalize them
- Team member should immediately place O2 for the hypoxic patient
- Team member should obtain PIV
- Team member should take basic history from mother

**Scenario progression:** *If airway positioned and 100% O2 initially applied, patient will have improvement of saturations to 95% but continued retractions and wheezing*

### END Round 1

Review initial assessment, recognition of respiratory distress, importance of initiating supplemental oxygen in a patient in respiratory distress, and importance of getting accurate history if available.

Review teaching points pertaining to expected actions as below:[Table t8-jetem-6-3-c64]

**Table t8-jetem-6-3-c64:**

Expected Action	Teaching Point
Rapid assessment of ABC	Assess airway, breathing and circulation. If the patient is unresponsive, the order of assessment would change to circulation, airway, breathing.
Check vital signs	Interpret and document initial vitals
Recognize signs of respiratory distress	Tachypnea, hypoxia, retractions, head-bobbing, tripoding
Immediately place O2 for the hypoxic patient	For the patient with distress + hypoxia, place non-rebreather face mask at 10–15 liters per minute to deliver 95–100% O2
Obtain IV	Place IV early to ensure access is available during resuscitation in case medications need to be administered.
Take basic history from mother	History is important to management. Try to obtain basic details from caregiver while you are performing other tasks, because the history will guide management
Recognize asthma as likely diagnosis based on history and physical	Expiratory wheezing and respiratory distress in a child, particularly with a past diagnosis of wheezing, should raise suspicion for asthma

### Round 2: Management of severe asthma exacerbation

**Objectives introduced this round:**

1. Know to initiate treatment for asthma with bronchodilator (salbutamol/ipratropium)
2. Know to manage severe asthma exacerbation using early delivery of steroids

**Restart from the beginning of the case**.

**Verbal prompt:** “EMS response call to a patient’s home. The patient is a 3-year-old female who presents with respiratory distress for 3 days. The mom found her with worsening breathing and cough for the past 3 days.”

**Other history (if asked):** Patient has had 2–3 wheezing episodes over the last 2–3 months. Strong family history of atopy and asthma.

**Initial vital signs:** T 37.0° C, HR 160, RR 50, BP 87/43, O2 70% on room air Weight per Broselow tape

**Initial physical exam**

AIRWAY: airway open
BREATHING: subcostal retractions, nasal flaring, diffuse wheezing heard on auscultation of bilateral lung fields
CIRCULATION: strong pulses, CR 2–3 seconds
DISABILITY: alert
OTHER: child has cyanosis to the mouth and is in distress. Otherwise assume other exam findings are normal

**Expected actions after verbal prompt**

- Repeat expected actions from previous round
  ○ Rapid assessment of ABC
  ○ Check vital signs
  ○ Start O2 for the hypoxic patient
  ○ Obtain PIV
  ○ Take basic history from mother

**Scenario progression #1**: *After airway positioned and 100% O2 initially applied, the patient will have improvement of saturations to 95% but continued retractions and wheezing*

**Expected actions after scenario progression**

- Team should recognize asthma exacerbation and initiate bronchodilators (salbutamol, ipratropium) immediately
- Begin treatment for asthma with salbutamol (or other short acting beta agonist) nebulization
- Initiate hydrocortisone (or other available oral [PO], intramuscular [IM], or IV glucocorticoid) due to severe respiratory distress
- Reassess vital signs and patient after treatment

**Scenario Progression #2:** *If bronchodilator, ipratropium, and steroid given, will note the following changes in vital signs and physical exam:*

*HR 170, RR 45, O2 saturation 95%*

*Breathing: improved but continues with scattered wheezing bilaterally*

### END Round 2

Discuss the recognition of asthma and reinforce the importance of rapid intervention and aggressive management in severe cases.

Review expected actions and discuss teaching points as below:[Table t9-jetem-6-3-c64]

**Table t9-jetem-6-3-c64:**

Expected Action	Teaching Point
Recognize asthma exacerbation and initiate bronchodilators immediately	Wheezing on exam suggests asthma. In patients with severe asthma, one may hear decreased air movement and only mild wheezing initially. As one treats the patient with medication, the wheezing may become louder. This means that treatment is working, and more air is moving.
Begin treatment for asthma with salbutamol nebulization	Salbutamol is a short acting beta-2 agonist. The dose is 5 mg. Ipratropium is an anticholinergic drug used early in the treatment of asthma; the dose is 500 mcg and can be mixed with salbutamol for nebulization.
Initiate hydrocortisone	Early administration of steroids is important in patients with asthma, and if available should be provided by EMS particularly for those patients in severe distress. The hydrocortisone dose is 2 mg/kg IV/IO/IM/PO.
Reassess vital signs and patient after treatment	It is always important to reassess vital signs and the patient after administration of any treatment.

### Round 3: Bag-mask Ventilation

**Objectives introduced this round:**

1. Be able to recognize progression to respiratory failure and need for bag-mask ventilation
2. Be able to deliver effective breaths by bag-valve mask ventilation

**Restart from the beginning. If initial actions performed well, can restart where needed**.

**Verbal prompt:** “EMS response call to a patient’s home. The patient is a 3-year-old female who presents with respiratory distress for 3 days. The mom found her with worsening breathing and cough for the past 3 days.”

**Other history (if asked):** Patient has had 2–3 wheezing episodes over the last 2–3 months. Strong family history of atopy and asthma.

**Initial vital signs:** T 37.0° C, HR 160, RR 50, BP 87/43, O2 70% on room air Weight per Broselow tape

**Initial physical exam**

AIRWAY: airway open
BREATHING: subcostal retractions, nasal flaring, diffuse wheezing heard on auscultation of bilateral lung fields
CIRCULATION: strong pulses, CR 2–3 seconds
DISABILITY: alert
OTHER: child has cyanosis to the mouth and is in distress. Otherwise assume other exam findings are normal

**Expected actions after verbal prompt**

- Repeat expected actions from previous rounds:
- Rapid assessment of ABC
- Check vital signs
- Start O2 in the hypoxic patient
- Obtain PIV
- Take basic history from mother
- Begin treatment for asthma with salbutamol nebulization
- Initiate hydrocortisone
- Reassess vital signs and patient after treatment

**Scenario Progression #1:** *After initiation of O2 and bronchodilators, the patient will have brief improvement of saturations*. The *patient will then drop oxygen saturation to 70s, with poor air movement; respiratory rate falls to 15*.

**Expected actions after round progression**

- Team to recognize deterioration in patient with respiratory distress
- Team to understand indications to begin bag-mask ventilation
- Team member to initiate proper bag-mask ventilation
- Team member to place oral airway if difficult to bag
- Team to consider supra/extra-glottic airway placement, if available, and needs ongoing BVM
- Team member to call to hospital department for report

**Scenario Progression #2**: *After brief BVM, patient’s saturations improve to 90% and air movement improves*.

### END Round 3

Discuss respiratory failure and decompensation in a patient with acute asthma exacerbation.

Review expected actions and teaching points as below:[Table t10-jetem-6-3-c64]

**Table t10-jetem-6-3-c64:**

Expected Action	Teaching Point
Recognize deterioration in patient with respiratory distress	Rapidly decreasing respiratory rate, drop in saturations, decreased air movement
Understand indications to begin BVM	Persistent hypoxia, apnea, ineffective respirations (shallow with poor air movement and unable to maintain saturations)
Proper technique in administering BVM	1 breath every 3–5 seconds, attach to O2, C–E technique to hold mask
Place oral airway if difficult to administer BVM	Oral airways will help to prevent the tongue from obstructing the airway.
Consider supra/extra-glottic airway placement if available and needs ongoing BVM	A more definitive airway in transport may help a single team member to bag effectively; consider placement prior to transport
Call to hospital department for report	Accurate and concise report of patient’s presentation, treatments and current condition

### Round 4: Full scenario without interruption

**Depending on time available, repeat the entire scenario from the beginning without interruption. If initial actions performed well, can restart where needed**.

### END of RCDP Session

**Final debriefing and feedback**

- Praise learners
- Provide areas for continued improvement

**Additional teaching points for asthma case**

Definition of asthma exacerbation:

- Episode of progressive increase in shortness of breath, wheezing, coughing, or chest tightness, or some combination of these symptoms.
- Many patients will present in respiratory distress.
- Patients typically have a decrease in airflow in their lungs.

Pathophysiology of asthma:

During an asthma exacerbation the following will happen to patients:

- Bronchoconstriction
- Airway hyper-responsiveness
- Airway inflammation resulting in airway edema and mucous plugs

Differential Diagnosis for respiratory distress:

- Croup
- Foreign body
- Reflux (GERD)
- Vocal cord dysfunction
- Heart failure

Important questions for EMS provider to ask family:

- Severity and duration of symptoms
- Any medications given prior to arrival? Any response to these medications?
- Any history of sleep disturbances?
- Any difficulty with exercise?
- Time of onset of symptoms?
- Any prior hospitalizations for asthma or breathing problems?
- Any family history of asthma?
- Any identifiable triggers (seasonal changes, exposure to smoke, recent illness, allergies)?

Physical exam for respiratory distress:

- Need to evaluate ability of patient to speak in full sentences
- Auscultate for decreased breath sounds and/or wheezing
- Vitals including respiratory rate, heart rate, oxygen saturation
- Any retractions or accessory muscle use? Any cyanosis?
- Any signs of a respiratory infection such as rhinorrhea, mucous swelling?

### Medications

Salbutamol: Is a short acting beta-2 adrenergic agonist causing smooth muscle relaxation--bronchodilation. Fast onset of action within 5–15 minutes

Ipratropium: It is an anticholinergic that causes bronchodilation by blocking acetylcholine receptors of bronchial smooth muscle. It may be given in three repeat does with Salbutamol. Its onset of action is within 15 minutes

Hydrocortisone, Methylprednisolone, Prednisone, Prednisolone, Dexamethasone: Systemic anti-inflammatory with a delayed onset of action so should be given as soon as possible.

## Appendix I Pediatric Simulation Case: Diarrhea with Hypovolemic Shock

**Learner Audience:**
Prehospital providers including emergency medical technicians, registered nurses, respiratory therapists, and physicians at any level of training.
**Time Required for Implementation:**
~ 60 minutes for multiple rounds of RCDP
**Recommended Number of Instructors:**

- 1 simulation instructor/debriefing facilitator per group of three to six learners
- 1 confederate/assistant per group of three to six learners

**Objectives:**
By the end of the session, learners should be able to: Cognitive:

1. Be able to rapidly assess airway, breathing, and circulation in a child
2. Be able to interpret abnormal vital signs and recognize signs of hypovolemic shock in the child with diarrhea
3. Be able to initiate fluid resuscitation in the child with hypovolemic shock
4. Know the importance of reassessing patients frequently. Continue to resuscitate with fluid boluses when the child continues to have signs of shock on reassessment
5. Be able to recognize and treat hypoglycemia
6. Be able to transport the patient to the hospital while continuing ongoing care

Technical:

1. Assign team roles
2. Use closed-loop communication
  ○ All messages or orders addressed to specific individuals
  ○ Team members confirm each request is understood and inform the leader when a task begins or ends
3. Respect each other with language and behavior
  ○ Use proper names with eye contact or touch
  ○ Share ideas and information as suggestions or constructive interventions, not as criticism
4. Provide a shared mental model (summary of scenario and next steps given to maintain situational awareness)

**Case Title:** Diarrhea with Hypovolemic Shock

**Equipment or Props Needed:**

Setup for All Rounds:

- Pediatric mannequin
- Equipment to be brought into the scenario by the prehospital provider team. In our trials, this included:
  ○ Monitors including blood pressure cuff and oxygen saturation monitor
  ○ Airway equipment including suction catheter, stethoscope, oral pharyngeal airway, nasopharyngeal airway, BVM with different size masks, supplemental oxygen, nasal cannula, simple face mask, non-rebreather facemask
  ○ IV equipment including various size pediatric IV cannulas, 18g needle or intraosseous needle, IV tubing
  ○ IV fluids including normal saline or Ringer’s lactate, dextrose containing fluid (D50, D25, D10, D5 if available)
  ○ Glucometer
  ○ Length-based tape to estimate weight
  ○ Splinting material
  ○ Cervical collar (C-collar) and backboard

**Common verbal prompt to start all rounds:** “You have been called to the home of a sick infant. The patient is a 6-month-old male with 2 days vomiting and diarrhea. His mother reports he is very sleepy today.”

**Additional history (if asked):** Emesis is nonbloody, nonbilious. Diarrhea is nonbloody. No fevers. Previously healthy.

**Initial Vital signs for all rounds (if asked):** T 36.5°C, HR 200, RR 40, BP 50/34, O2 98% on RA

Weight per Broselow tape (approx. 6 kg). If asked for a weight, parents to answer they don’t know

**Initial Physical exam findings for all rounds** (if asked):

AIRWAY: Airway open
BREATHING: Lungs with shallow respirations, clear bilaterally
CIRCULATION: No murmur, weak central and peripheral pulses. CR 4–5 seconds, extremities cool
OTHER: Poor skin turgor, dry mucous membranes (MM), eyes sunken. Abdomen soft, bowel sounds active. Pt lethargic, brief cry to interventions but minimally active on exam

### Round 1: Initial assessment

**Objectives introduced this round:**

1. Rapidly assess airway, breathing, and circulation in a child
2. Place IV or IO
3. Be able to recognize abnormal vital signs

**Restart scenario from beginning**.

**Verbal Prompt:** “You have been called to the home of a sick infant. The patient is a 6-month-old male with 2 days vomiting and diarrhea. His mother reports he is very sleepy today.”

**Additional history (if asked):** Emesis is nonbloody, nonbilious. Diarrhea is nonbloody. No fevers. Previously healthy.

**Vital signs (if asked):** T 36.5°C, HR 200 RR 40 BP 50/34 O2 98% on RA

Weight per Broselow tape (approx. 6 kg). If asked for a weight, parents to answer they don’t know

**Initial physical exam** (if asked):

AIRWAY: Airway open
BREATHING: Lungs with shallow respirations, clear bilaterally
CIRCULATION: No murmur, weak central and peripheral pulses. CR 4–5 seconds, extremities cool
OTHER: Poor skin turgor, dry MM, eyes sunken. Abdomen soft, bowel sounds active. Pt lethargic, brief cry to interventions but minimally active on exam.

**Expected actions after verbal prompt:**

- Team should perform rapid assessment of ABC
- Team member should check vital signs, and team should recognize abnormal vital signs
- Team member should obtain IV/IO access
- Team member should take basic history from mother

### END Round 1

Review the initial exam and reinforce the importance of rapid initial assessment.

Review expected actions and discuss teaching points as below:[Table t11-jetem-6-3-c64]

**Table t11-jetem-6-3-c64:**

Expected Action	Teaching Point
Rapid assessment of ABC	When presented with an ill patient, assess airway, breathing, & circulation and immediately intervene if an emergent situation is found. Remember, this applies to a RESPONSIVE child. If the child is unresponsive, the order changes to CAB.
Check vital signs	Document initial vitals: HR, RR, BP, O2 sat. Temp can be taken after other unstable vitals addressed.
Obtain IV/IO access	Try PIV first, particularly if patient awake. IO if critically ill, particularly if altered mental status / coma.
Recognize abnormal vitals	HR for age: over 180 abnormal BP: SBP should be > (age in years ×2) + 70. In infants over 1 month, SBP should be at least 70.
Take basic history from mother	History is important to management. Try to obtain basic details from caregiver while you are performing other tasks because the history will guide management.

### Round 2: Recognition and Management of shock

**Objectives introduced this round:**

1. Recognize signs of hypovolemic shock in the child with diarrhea, including weak pulses, tachycardia, and hypotension
2. Initiate fluid resuscitation in the child with hypovolemic shock
3. Know the importance of reassessing the patient after each fluid bolus

**Restart scenario from beginning**.

**Verbal Prompt:** “You have been called to the home of a sick infant. The patient is a 6-month-old male with 2 days vomiting and diarrhea. His mother reports he is very sleepy today.”

**Additional history (if asked):** Emesis is nonbloody, nonbilious. Diarrhea is nonbloody. No fevers. Previously healthy.

**Vital signs (if asked):** T 36.5 °C, HR 200, RR 40, BP 50/34, O2 98% on RA.

Weight per Broselow tape (approx. 6 kg). If asked for a weight, parents to answer they don’t know

**Initial Physical exam** (if asked):

AIRWAY: Airway open
BREATHING: Lungs with shallow respirations, clear bilaterally
CIRCULATION: No murmur, weak central and peripheral pulses. CR 4–5 seconds, extremities cool
OTHER: Poor skin turgor, dry MM, eyes sunken. Abdomen soft, bowel sounds active. Pt lethargic, brief cry to interventions but minimally active on exam

**Expected actions after verbal prompt:**

- Team should repeat expected actions from prior round
  ○ Rapidly assess ABC
  ○ Check vital signs and recognize abnormal vital signs
  ○ Obtain IV/IO access
  ○ Take basic history from mother
- Team should recognize hypovolemic shock and verbalize
- Team should estimate patient’s weight using Broselow tape and initiate IV bolus of fluids
- Team should reassess patient after first IV fluid bolus
- Clear communication with teammates

**Scenario progression:** *After first bolus, HR 195, BP 60/39, RR 40, sat 98 on RA. Physical exam** s**hows capillary refill improved to 4, extremities are still cool, there is no murmur, and there is** n**o hepatomegaly*.

### END Round 2

Review expected actions and teaching points as below:[Table t12-jetem-6-3-c64]

**Table t12-jetem-6-3-c64:**

Expected Action	Teaching Point
Recognize shock (& etiology)	In a hypotensive patient with diarrhea, most likely hypovolemic shock from fluid losses.
Estimate weight	Broselow tape (or other weight-based tape)
Give IV fluids	The treatment of hypovolemic shock is aggressive fluid resuscitation. Rapid bolus, 20 mL/kg push over 5 min or less. Only isotonic fluids should be given as a bolus.
Reassess after bolus	Always recheck vitals, perfusion, and respiratory status after giving a bolus. Also check for signs of over-resuscitation including new murmur, crackles on pulmonary exam, or hepatomegaly.
Clear communication	Assign one person to be the “team lead.” Closed-loop communication (leader gives a clear request, and 2^nd^ team member reports back when the request is complete). Mental modeling: team leader shares thought process (ex: “I think this patient has hypovolemic shock, so we will treat with IV fluid boluses.”)

### Round 3: Continued fluid management and hypoglycemia

**Objectives introduced this round:**

1. Be able to recognize and treat hypoglycemia
2. Know the importance of reassessing patients frequently. Continue to resuscitate with fluid boluses when the child continues to have signs of shock on reassessment
3. Be able to transport the patient to the hospital while continuing ongoing care

**Restart case from beginning. If initial actions performed well, can restart where needed**.

**Verbal Prompt:** “You have been called to the home of a sick infant. The patient is a 6-month-old male with 2 days vomiting and diarrhea. His mother reports he is very sleepy today.”

**Additional history (if asked):** Emesis is nonbloody, nonbilious. Diarrhea is nonbloody. No fevers. Previously healthy.

**Initial Vital signs (if asked):** T 36.5°C, HR 200, RR 40, BP 50/34, O2 98% on RA

Weight per Broselow tape (approx. 6 kg). If asked for a weight, parents to answer they don’t know

**Initial Physical exam** (if asked):

AIRWAY: Airway open.
BREATHING: Lungs with shallow respirations, clear bilaterally
CIRCULATION: No murmur, weak central and peripheral pulses. CR 4–5 seconds, extremities cool
OTHER: Poor skin turgor, dry MM, eyes sunken. Abdomen soft, bowel sounds active. Pt lethargic, brief cry to interventions but minimally active on exam

**Expected actions after verbal prompt:**

- Team should repeat expected actions from prior rounds
  ○ Rapidly assess ABC
  ○ Check vital signs
  ○ Obtain IV/IO access
  ○ Recognize abnormal vital signs
  ○ Take basic history from mother
  ○ Recognize shock and etiology and verbalize
  ○ Initiate first IV bolus of fluids
  ○ Reassess patient after first IV bolus of fluids

**Scenario progression #1:** *After first bolus, HR 195, BP 60/39, RR 40, sat 98 on RA. Physical** e**xam shows capillary refill improved to 4, extremities are still cool, there is no murmur, and** t**here is no hepatomegaly*.

**Expected actions after scenario progression:**

- Recognize continued hypotension
- Continue to give repeat IV fluid boluses, reassessing after each bolus
- Check blood glucose and give IV dextrose bolus if hypoglycemic
- Repeat blood glucose check after dextrose bolus
- Initiate transportation to hospital while continuing ongoing care.

**Scenario progression #2:** *If the team checks glucose, the result is 1.8 mmol/L (32 mg/dL). If an** a**ppropriate dextrose bolus is given and it is rechecked, repeat will be 4.2 (75 mg/dL)*. *Provide improvements in patient’s heart rate, blood pressure, and capillary refill after each** f**luid bolus until they have normalized*.

### END Round 3

Ensure trainees are transporting to the hospital during this ongoing care.

Review expected actions and teaching points as below:[Table t13-jetem-6-3-c64]

**Table t13-jetem-6-3-c64:**

Expected Action	Teaching Point
Recognize continued hypotension	Recheck vitals after every intervention
Repeat fluids	Continue to bolus 20 mL/kg, reassess after each bolus. In hypovolemic shock, patients may require well over 60 mL/kg of NS.
Check glucose	Hypoglycemia is common in patients with diarrhea and may be a cause of altered mental status in these patients. For reference: <3.3 mmol/L (60mg/dl) – 3.9 mmol/L (70 mg/dl) is considered low but usually asymptomatic <2.8 mmol/L (50 mg/dl) – 3 mmol/L (54 mg/dl) is low and patients are usually symptomatic
Administer dextrose bolus	Dextrose can be given by PIV in any concentration up to 12.5%. If you have an IO, you can give any concentration, including D50. Use the “rule of 50”: 1 mL/kg of D50 (IO only!), 5 mL/kg of D10.

### Round 4: Full scenario without interruptions

**Repeat the scenario from start to end without interruption. If initial actions performed**** w****ell, can restart where needed**.

### END OF RCDP Session

**Final debriefing and feedback**

- Praise learners
- Provide areas for continued improvement

**Additional teaching points for diarrhea with hypovolemic shock case**

- Management of hypoglycemia: “Rule of 50”
  - **50 ÷ percent dextrose = mL/ kg needed for dose**
  - Examples:
    ○ 50 ÷ 50% dextrose = 1 mL/kg dose
    ○ 50 ÷ 25% dextrose = 2 mL/kg dose
    ○ 50 ÷ 10% dextrose = 5 mL/kg dose
    ○ 50 ÷ 5% dextrose = 10 mL/kg dose
- Can give D50 through IO.
- Should not give more than D10 via PIV.
- Can mix D10 from D50 by mixing 4 parts NS or sterile water with 1-part D50.

## Appendix J Pediatric Simulation Case: Newborn Delivery

**Learner Audience:**
Prehospital providers including emergency medical technicians, registered nurses, respiratory therapists, and physicians at any level of training.
**Time Required for Implementation:**
~ 60 minutes for multiple rounds of RCDP
**Recommended Number of Instructors:**

- 1 simulation instructor/debriefing facilitator per group of three to six learners
- 1 confederate/assistant per group of three to six learners

**Objectives:**
By the end of the session, learners should be able to: Cognitive:

1. Know to immediately warm/dry/suction/stimulate all infants at deliveries
2. Be able to rapidly identify a non-vigorous infant by looking for color, tone, and respiratory effort
3. Be able to manage an initially non-vigorous infant with immediate positive pressure ventilation
4. Be able to manage initial bradycardia in a newborn and know when to initiate chest compressions

Technical:

1. Know the steps for correct umbilical cord cutting/clamping after delivery
2. Be able to perform adequate positive pressure ventilation (bag-mask ventilation) in a newborn
3. Be able to perform appropriate chest compressions in a newborn in a 3:1 ratio when bradycardia is not responsive to bag-valve mask ventilation alone

Behavioral:

1. Assign team roles
2. Use closed-loop communication
  ○ All messages or orders addressed to specific individuals
  ○ Team members confirm each request and inform the team leader when a task begins or ends
3. Respect each other with language and behavior
  ○ Use proper names with eye contact or touch
  ○ Share ideas and information as suggestions or constructive interventions, not as criticism
4. Provide a shared mental model (summary of scenario and next steps given to maintain situational awareness)

**Case Title:** Pediatric Simulation Case: Newborn Delivery

**Equipment or Props Needed:**

Setup for All Rounds:

- Infant mannequin
- Equipment to be brought into the scenario by the prehospital provider team. In our trials, this included:
  ○ Monitors including blood pressure cuff and oxygen saturation monitor
  ○ Airway equipment including suction catheter, stethoscope, oral pharyngeal airway, nasopharyngeal airway, bag-valve mask (BVM) with different sizes of masks, nasal cannula, simple face mask, non-rebreather face mask
  ○ Intravenous (IV) access equipment including various sizes of pediatric IV cannulas, 18g needle or intraosseous (IO) needle, IV tubing
  ○ IV fluids including normal saline or Ringer’s lactate, dextrose containing fluid (D50, D25, D10, D5 if available)
  ○ Glucometer
  ○ Length-based tape to estimate weight
  ○ Splinting material
  ○ Cervical collar (c-collar) and backboard

**Common verbal prompt to start all rounds:** “You have just been called to a home as a mother delivers a newborn baby. The mother at this time is awake and alert, and another team is caring for her. However, you notice as the baby is delivered that he is blue in color and has no cry.”

**Additional case information for the instructor:**

This newborn is blue, not breathing, and is not moving.
AIRWAY/BREATHING: No spontaneous respirations. Unable to measure oxygen saturation
CIRCULATION: Initial HR 80, strong umbilical and brachial pulses (will drop to 40s)
OTHER: Patient cyanotic, limp, no cry. No response to stimulation
Teams will need to focus on initial interventions for the non-vigorous infant, recognition of respiratory arrest, and need to initiate chest compressions for persistent bradycardia when heart rate does not increase after 30–60 seconds of positive-pressure ventilation

### Round 1: Warm/Dry/Stimulate

**Objectives introduced this round:**

1. Know to immediately warm/dry/suction/stimulate all infants at deliveries
2. Know the steps for correctly cutting and clamping the umbilical cord after delivery.

**Verbal Prompt:** “You have just been called to a home as a mother delivers a newborn baby. The mother at this time is awake and alert, and another team is caring for her. However, you notice as the baby is delivered that he is blue in color and has no cry.”

**Vital Signs/Physical exam:** Not yet applicable.

**Expected actions after verbal prompt:**

- Warm, dry, suction, stimulate, and cut/clamp the cord on the baby immediately

**Scenario progression:** *No case progression yet. ****If the team asks for vital signs or physical exam, stop and start over after giving partial feedback***.

### END of Round 1

Reinforce the importance of rapid initial stimulation of ALL babies regardless of appearance to prevent apnea. As opposed to all other non-neonatal resuscitations, initial warming/drying/stimulation should occur prior to any assessment of airway/breathing/circulation.

Review the expected action and discuss teaching point below:[Table t14-jetem-6-3-c64]

**Table t14-jetem-6-3-c64:**

Warm, dry, suction, stimulate, and cut/clamp the cord on the baby	NO MATTER WHAT the baby looks like upon delivery, every infant needs to be warmed, dried, suctioned, stimulated, and have its cord cut/clamped. Bulb suction the nares and mouth at the perineum. Cord should be cut immediately after (or during, if more than 1 provider) stimulation and airway clearance. Hold the baby at the level of the mother, and clamp both ends of the cord before cutting.

### Round 2: Initial assessment of a newborn

**Objectives introduced this round:**

1. Be able to rapidly identify a non-vigorous infant by looking for color, tone, and respiratory effort
2. Be able to manage an initially non-vigorous infant with immediate positive pressure ventilation

**Restart from the beginning of the case**.

**Verbal prompt:** “You have just been called to a home as a mother delivers a newborn baby. The mother at this time is awake and alert, and another team is caring for her. However, you notice as the baby is delivered that he is blue in color and has no cry.”

**Vital Signs (if asked):** RR 0, O2 not yet available. HR not yet to be checked. Temperature and blood pressure measurement should not be attempted until the patient is stabilized.

**Physical Exam (if asked):** Patient is limp, cyanotic, has no cry or respiratory effort, no response to stimulation.

**Expected actions after verbal prompt:**

**Within the first 60 seconds after delivery:**

- Team should immediately warm, dry, suction, stimulate, and clamp/cut the cord
- Team member should perform rapid assessment of the newborn (color, respiratory effort and tone) and recognize this infant is non-vigorous
- Team member should initiate positive pressure ventilation for a non-vigorous infant

*No progression of scenario. If the team asks for full physical exam findings, pause briefly** a**nd give feedback that this will not be relevant until the patent is fully stabilized*.

### END Round 2

Review the importance of rapid assessment for vigorousness and prompt intervention with bag-mask ventilation if not vigorous. **The non-vigorous newborn with respiratory arrest**** s****hould have airway cleared, stimulation, cord clamped/cut, and bag-mask ventilation**** s****tarted WITHIN ONE MINUTE** of delivery.

Review expected actions and teaching points as below:[Table t15-jetem-6-3-c64]

**Table t15-jetem-6-3-c64:**

Expected Action	Teaching Point
Rapid assessment of the newborn infant for vigorousness, followed by positive pressure ventilation if non-vigorous	An infant is considered non-vigorous if it is: blue (centrally), limp, OR not breathing. If any of these are present, the ONLY intervention required at this point is bag-mask ventilation with C-E technique, proper mask size, and rate of respirations 40–60 breaths/ min. Reposition the airway if difficult. Use an infant bag or only squeeze a larger bag enough to cause chest rise to prevent excessive airway pressure. Further assessment is NOT needed (yet). Chest compressions are NOT indicated (yet)

### Round 3: Assess heart rate in response to BVM

**Objectives introduced this round:**

1. Be able to manage initial bradycardia in a newborn and know when to initiate chest compressions

**Restart from the beginning of the case**.

**Verbal Prompt:** “You have just been called to a home as a mother delivers a newborn baby. The mother at this time is awake and alert, and another team is caring for her. However, you notice as the baby is delivered that he is blue in color and has no cry.”

**Vital Signs:** RR 0, O2 not yet available. HR not yet to be checked. Temperature and blood pressure measurement should not be attempted until the patient is stabilized.

**Physical Exam (if asked):** Cyanotic, no cry, no response to stimulation. Limp.

**Expected actions after verbal prompt:**

**Within 60 seconds of delivery:**

- Team should immediately warm, dry, suction, stimulate, and clamp/cut the cord
- Team member should perform rapid assessment of the newborn (color, respiratory effort, and tone) and recognize this infant is non-vigorous
- Team member should initiate positive pressure ventilation for a non-vigorous infant
- **Assess the heart rate (by chest auscultation or palpation of umbilical stump)** **a** **fter PPV is given for 30 seconds**

**Scenario progression #1:** *Vital signs: The heart rate is 80 after 30 seconds of PPV. SpO2 (if** a**vailable) is undetectable*.

**Expected action after scenario progression:**

- Team member should continue positive pressure ventilation (PPV), and reassess heart rate after 30 additional seconds
- Do not initiate chest compressions

### END Round 3

Reinforce how to treat a non-vigorous newborn. Review importance of BVM at a rate of 40–60 breaths per minute × 30 seconds. If the HR is <100 after 30 seconds of bag-mask ventilation, then ventilation should be done for 30 more seconds before reassessing the heart rate.

Review expected actions and teaching points as below:[Table t16-jetem-6-3-c64]

**Table t16-jetem-6-3-c64:**

Expected Action	Teaching Point
Assessment of heart rate after positive pressure ventilation given for 30 seconds; If HR is <100 continue PPV (bag-mask ventilation) and recheck HR after 30 additional seconds.	Bradycardia is usually due to apnea in the neonate. Therefore, chest compressions are not indicated unless bag-mask ventilation × 60 seconds is ineffective.
If HR is <100 on reassessment, continue PPV	Bag-mask ventilation is required if HR remains <100. Chest compressions are not indicated if HR remains above 60 after 1 minute of BVM.

### Round 4: Effective CPR for ongoing bradycardia

**Objectives introduced this round:**

1. Initiate appropriate chest compressions: ventilations in a 3:1 ratio when bradycardia is not responsive to 60 seconds of bag-valve mask ventilation alone

**Restart from the beginning of the case**.

**Verbal Prompt:** “You have just been called to a home as a mother delivers a newborn baby. The mother at this time is awake and alert, and another team is caring for her. However, you notice as the baby is delivered that he is blue in color and has no cry.”

**Vital Signs:** RR 0, O2 not yet available. HR not yet to be checked. Temperature and blood pressure measurement should not be attempted until the patient is stabilized.

**Physical Exam (if asked):** Cyanotic, no cry, no response to stimulation. Limp.

**Expected actions:**

- Immediately warm, dry, suction, and clamp/cut the cord
- Perform rapid assessment of the newborn and recognize this infant is non-vigorous
- Initiate positive pressure ventilation, attach SpO2 monitors, and EKG leads for a non-vigorous infant with no respiratory effort within 60 seconds of delivery
- **Assess the heart rate (by chest auscultation or palpation of umbilical stump)** **a** **fter PPV is given for 30 seconds**

**Scenario progression #1:** *HR is 80. SpO2 undetectable*.

**Expected actions after scenario progression #1:**

- **Continue PPV. Recheck HR after 30 additional seconds**

**Scenario progression #2:** *HR has dropped to 40*.

**Expected actions after scenario progression #2:**

- Initiate proper chest compressions while continuing PPV in 3:1 compression: breath ratio.
- Pause compressions to allow reassessment of heart rate every 2 minutes.

### END of Round 4

Review expected actions and teaching points as below:[Table t17-jetem-6-3-c64]

**Table t17-jetem-6-3-c64:**

Expected Action	Teaching Point
Assessment of heart rate after positive pressure ventilation given for 30 seconds, followed by additional positive pressure ventilation for 30 seconds if HR<100	Bradycardia is usually due to apnea in the neonate. Therefore, chest compressions are not indicated unless HR is <60 after 60 seconds of bag-mask ventilation
Give proper chest compressions	Ratio of compressions to breaths in neonatal resuscitation is 3:1. Use encircling thumbs technique. Pause and reassess heart rate and respiratory effort every 2 minutes

- Review proper CPR technique and need for reassessment.
- Review the need for adrenaline if bradycardia is still ongoing after 2 minutes of CPR and within the scope of practice of the provider.

Note: adrenaline may only be available in 1mL ampules of 1:1,000 concentrations. If planning to administer adrenaline via IV/IO, the 1:1,000 concentrations must be diluted to 1:10,000 concentrations. This can be done by mixing the 1mL of 1:1,000 with 9mL of NS or water for injection. The dose will be 1 mL/kg IV/IO. The 1:1,000 dose of 1mL/kg may only be administered via an endotracheal tube.

### Round 5

**Repeat the entire scenario without interruption. If initial actions performed well, can start**** w****here needed**.

### END OF RCDP SESSION

**Final debriefing and feedback**

- Praise learners
- Provide areas for continued improvement

**Additional teaching points for newborn delivery case**

**Review newborn resuscitation flow chart below**.

**Review APGAR (Appearance, Pulse, Grimace, Activity, and Respiration)**** s****cores below**.[Fig f3-jetem-6-3-c64]

**APGAR scores:** [Table t18-jetem-6-3-c64]

**Table t18-jetem-6-3-c64:**

	0	1	2
Appearance: Color	Blue or pale	Acrocyanosis	Completely pink
Pulse: Heart rate	Absent	<100 bpm	>100 bpm
Grimace: Reflex irritability	No response	Grimace	Cry or active withdrawal
Activity: Muscle tone	Limp	Some flexion	Active motion
Respiration	Absent	Weak cry	Good, crying

APGAR is utilized as an assessment of newborn babies at 1 minute, 5 minutes, and 10 minutes.

## Appendix K Pediatric Simulation Case: Seizure

**Learner Audience:**
Prehospital providers including emergency medical technicians, registered nurses, respiratory therapists, and physicians at any level of training.
**Time Required for Implementation:**
~ 60 minutes for multiple rounds of RCDP
**Recommended Number of Instructors:**

- 1 simulation instructor/debriefing facilitator per group of three to six learners
- 1 confederate/assistant per group of three to six learners

**Objectives:**
By the end of the session, learners should be able to:: Cognitive:

1. Be able to perform initial ABC management of seizing patient
2. Know to treat the actively seizing patient with benzodiazepine
3. Recognize various causes of seizures including hypoglycemia and toxic ingestion
4. Advanced learners should be able to treat the patient with organophosphate toxicity
5. Anticipate airway compromise in a patient who has received benzodiazepines

Technical:

1. Be able to manage the airway in a seizing patient

Behavioral:

1. Assign team roles
2. Use closed-loop communication
  ○ All messages or orders addressed to specific individuals
  ○ Team members confirm each request and inform the team leader when a task begins or ends
3. Respect each other with language and behavior
  ○ Use proper names with eye contact or touch
  ○ Share ideas and information as suggestions or constructive interventions, not as criticism
4. Provide a shared mental model (summary of scenario and next steps given to maintain situational awareness)
5. Communicate with receiving facility about an incoming seizure/toxic exposure case

**Case Title:** Pediatric Simulation Case: Seizure

**Equipment or Props Needed:**

Setup for All Rounds:

- Pediatric mannequin
- Equipment to be brought into the scenario by the prehospital provider team. In our trials, this included:
  ○ Monitors including blood pressure cuff and oxygen saturation monitor
  ○ Airway equipment including suction catheter, stethoscope, oral pharyngeal airway, nasopharyngeal airway, O2, BVM with different size masks, supplemental oxygen, nasal cannula, simple face mask, non-rebreather facemask
  ○ IV equipment including various size pediatric IV cannulas, 18g needle or intraosseous (IO) needle, IV tubing
  ○ IV fluids including normal saline or Ringer’s lactate, dextrose containing fluid (D50, D25, D10, D5 if available)
  ○ Glucometer
  ○ Pediatric length-based tape for estimating weight
  ○ Splinting material
  ○ Cervical collar (c-collar) and backboard
- Empty bottle of pesticide as a prop, or empty container labelled as such

**Common verbal prompt to start all rounds:** “EMS response call to a private home in Block 3 for chief complaint of an unresponsive child who is ‘shaking.’ The patient is a 7-year-old male, previously healthy, who was last seen playing outside his home. EMS team finds the child unresponsive and shaking movements in all extremities.”

**Additional History (if asked):** If mother is questioned, she will state that patient was playing near some chemicals the last time he was seen healthy and will point to the empty bottle of pesticide.

**Additional information for the instructor:** This patient has been exposed to organophosphates and presents with seizure. Learner will need to **manage the airway**, treat a seizure, recognize the reason for seizure is toxic ingestion, and decontaminate the patient.

**Initial Vital Signs for all rounds:** Unable to obtain because patient is shaking

Weight per Broselow tape

**Physical Exam for all rounds:**

AIRWAY: a lot of secretions
BREATHING: shallow gasping respirations
CIRCULATION: capillary refill < 3 seconds, cyanosis to mouth
DISABILITY: patient not responsive and shaking of bilateral upper and lower extremities

### Round 1: Initial assessment of seizing patient

**Objectives introduced this round:**

1. Be able to perform initial ABC management of seizing patient

**Verbal prompt:** “EMS response call to a private home in Block 3 for chief complaint of an unresponsive child who is ‘shaking.’ The patient is a 7-year-old male, previously healthy, who was last seen playing outside his home. EMS team finds the child unresponsive and shaking movements in all extremities.”

**Additional History (if asked):** If mother is questioned, she will state that patient was playing near some chemicals the last time he was seen healthy and will point to the empty bottle of pesticide.

**Initial Vital Signs (if asked):** Unable to obtain because patient is shaking

Weight per Broselow tape. If O2 sat checked, initially 82%

**Initial Physical Exam (if asked):**

AIRWAY: a lot of secretions
BREATHING: shallow gasping respirations
CIRCULATION: capillary refill < 3 seconds, cyanosis to mouth
DISABILITY: patient not responsive and shaking of bilateral upper and lower extremities

**Expected actions after verbal prompt:**

- Rapid assessment of Airway, Breathing, Circulation.
- Protect the airway by placing on his side. Do not place ANYTHING in patient’s mouth.
- Administer oxygen to the patient.
- Take basic history from mother.

**Scenario progression:** *If airway is positioned and O2 administered, saturations will improve to 90%. Seizure will continue*.

### END Round 1

Review initial exam, reinforce importance of rapid initial assessment, recognizing airway compromise in a seizing patient and importance of administering oxygen while preparing for further management.

Review expected actions and teaching points below:[Table t19-jetem-6-3-c64]

**Table t19-jetem-6-3-c64:**

Expected Action	Teaching Point
Rapid assessment of ABC	When presented with an ill patient, assess airway, breathing, and circulation and immediately intervene if an emergent situation is found. Remember, in an unresponsive and **not moving (not seizing) patient**, order changes to CAB.
Protect the airway	Roll patient on his side. Do not place anything in patient’s mouth--do not place oral airway or fingers because this can cause damage to the patient’s teeth/jaw or injury to the caregiver. Rolling the patient on his side will prevent aspiration should the patient vomit.
Administer oxygen	Administer oxygen NRB 10–15 liters per min to a patient who is actively seizing and is hypoxic.
Take basic history from mother	History is important to management. Try to obtain basic details from caregiver while you are performing other tasks because history will guide management

### Round 2: Seizure management

**Objectives introduced this round:**

1. Place PIV
2. Treat the actively seizing patient with a benzodiazepine

**Start case from beginning**. *Note: if no EMT-A or nurse on simulation team, can adjust** s**cenario to make the patient hypoglycemic with glucose of 1.6 mmol/L--(treatment for** s**eizure will be dextrose containing fluid bolus 5 mL/kg of D10 fluid)*

**Verbal prompt:** “EMS response call to a private home in Block 3 for the chief complaint of an unresponsive child who is ‘shaking’. The patient is a 7-year-old male, previously healthy who was last seen playing outside his home. The EMS team finds the child unresponsive and shaking movements in all extremities.”

**Additional History (if asked):** If mother is questioned, she will state that patient was playing near some chemicals the last time he was seen healthy and will point to an empty bottle of pesticide.

**Initial Vital Signs (if asked):** Unable to obtain because the patient is shaking

Weight per Broselow tape. If O2 sat checked, initially 82%

**Initial Physical Exam (if asked):**

AIRWAY: a lot of secretions
BREATHING: shallow, gasping respirations
CIRCULATION: capillary refill < 3 seconds, cyanosis to mouth
DISABILITY: patient not responsive with generalized tonic-clonic movements of bilateral upper and lower extremities

**Expected actions after verbal prompt:**

- Repeat expected actions from prior round
  ○ Rapid assessment of ABC
  ○ Place patient on his side to protect the airway
  ○ Administer oxygen
  ○ Take basic history from mother
- Place PIV
- Manage the seizure with intramuscular (IM) or intranasal (IN) diazepam 0.2 mg/kg (maximum 10 mg), or intravenous (IV) if readily obtainable; or rectal diazepam 0.5 mg/kg up to 10 mg if no IV access.
- Check blood glucose. Treat with dextrose IV or intraosseous (IO) if hypoglycemic.

**Scenario progression:** *If airway is positioned and O2 administered, saturations will improve to 90%. Seizure will continue. If benzodiazepine is given, seizure will stop; otherwise, patient will** c**ontinue seizing*.

***Glucose*** *(if asked) is 4.4 mmol/L (80 g/dL). No dextrose is needed.*

*Provide Secondary physical exam findings (if asked):*

**Pupils**: pinpoint
**Eyes**: significant tearing from eyes
**Mouth**: significant salivation and secretions
**Lungs**: shallow respirations, coarse breath sounds throughout with scant wheezing

*New vital signs (if asked): HR 65, RR 9, BP 78/55, SpO2 90%*

### END Round 2

Review expected actions and teaching points as below:[Table t20-jetem-6-3-c64]

**Table t20-jetem-6-3-c64:**

Expected Action	Teaching Point
Manage seizure	If IV is readily obtained, give diazepam 0.2mg/kg IV or 0.5mg/kg per rectum (up to 10 mg). Other possible medications include midazolam 0.1mg/kg IV or 0.2mg/kg IM.
Obtain access	PIV generally sufficient in a patient with seizure. However, if there is a need to immediately administer fluids, dextrose, or medications other than benzodiazepines, may consider placing IO.
Check glucose	Glucose should always be checked in a patient actively seizing. If low, treat with dextrose IV or IO. Up to 12.5% dextrose can be given via IV, any concentration via IO. Use the “rule of 50” (see “Additional Teaching Points” below): % dextrose × mL/kg = 50. <3.3 mmol (60)–3.9 mmol (70) considered low but usually symptomatic <2.8 mmol (50)–3 mmol (54).

### Round 3: Post seizure care, identifying cause of seizure

**Objectives introduced this round:** Anticipate airway compromise in a patient who has received benzodiazepines

1. Recognize various causes of seizures including toxic ingestion
2. Advanced learners should be able to recognize and treat organophosphate toxicity

**Start scenario from beginning**.

**Verbal prompt:** “EMS response call to a private home in Block 3 for chief complaint of an unresponsive child who is ‘shaking.’ The patient is a 7-year-old male, previously healthy who was last seen playing outside his home. The EMS team finds the child unresponsive and shaking movements in all extremities.

**Additional History (if asked):** If mother is questioned, she will state that patient was playing near some chemicals the last time he was seen healthy, and will point to an empty bottle of pesticide

**Initial Vital Signs (if asked):** Unable to obtain because the patient is shaking

Weight per Broselow. If O2 sat checked, initially 82%

**Initial Physical Exam (if asked):**

AIRWAY: a lot of secretions
BREATHING: shallow, gasping respirations
CIRCULATION: capillary refill < 3 seconds, cyanosis to mouth
DISABILITY: patient not responsive with generalized tonic-clonic movements of bilateral upper and lower extremities

**Expected actions:**

- Repeat expected actions from prior round:
  ○ Rapid assessment of ABC
  ○ Place patient on his side to protect the airway
  ○ Administer oxygen
  ○ Take basic history from mother
  ○ Place PIV
  ○ Manage the seizure with benzodiazepine
  ○ Check blood glucose. Treat with dextrose IV or IO if hypoglycemic

**Scenario progression #1:** *If airway is positioned and O2 administered, saturations will improve (briefly) to 90%. Seizure will stop after benzodiazepine is given*.

***Glucose*** *(if asked) is 4.4 mmol/L (80 mmol/dL). No dextrose is needed*

*Provide Secondary physical exam findings (if asked):*

**Pupils**: pinpoint
**Eyes**: significant tearing from eyes
**Mouth**: significant salivation and secretions
**Lungs**: shallow respirations, coarse breath sounds throughout with scant wheezing

*New vital signs (if asked):* **HR 65 RR 9 BP 78/55 *****SpO2 dropped to 70% after benzodiazepine****** g******iven***

**Expected action after scenario progression #1:**

- Provide post-seizure care by suctioning secretions, positioning the airway, placing an oropharyngeal or nasopharyngeal airway, and administering oxygen.

**Scenario progression #2:** *If jaw thrust, suctioning, O2 applied, saturations will improve to 95%. You have the option of dropping saturations further and requiring bag-mask ventilation if** n**eeded*.

**Expected action after scenario progression #2:**

- Create a differential diagnosis for possible causes of seizures
- For advanced learners:
  ○ Recognize symptoms of cholinergic toxicity (including lacrimation, salivation, bronchoconstriction, pinpoint pupils, hypotension, and bradycardia) in the setting of pesticide exposure
  ○ Decontaminate patient by removing all clothing and rinsing if possible
  ○ Give atropine to manage organophosphate toxicity

### END Round 3

Review post-seizure care and differential diagnosis for seizures. For advanced learners, discuss signs, symptoms and treatment of organophosphate poisoning leading to cholinergic toxicity.

Review expected actions and teaching points below:[Table t21-jetem-6-3-c64]

**Table t21-jetem-6-3-c64:**

Expected Action	Teaching Point
Provide care after seizure is stopped	Monitor airway and breathing. Patient may have shallow respirations, particularly if several doses of benzodiazepine were needed. First line interventions include positioning the airway, placing OPA or NPA, administering oxygen. If these measures are not effective, consider BVM.
Create a differential of possible causes of seizures	EMS team should be able to discuss other possible causes of seizures including but not limited to, hypoglycemia, other electrolyte abnormalities (Na+, Ca2+), trauma, brain mass or brain bleed, epilepsy.
Recognize symptoms of cholinergic toxicity (**FOR ADVANCED LEARNERS)**	Use the mnemonic DUMBBELLSS (see info below regarding teaching points in patients with cholinergic toxicity). With severe toxicity, patients may seize. This patient has lacrimation (tearing of eyes), salivation, bronchoconstriction (difficulty breathing), pinpoint pupils, hypotension, bradycardia, and seizures all due to cholinergic toxicity (pesticide exposure of patient).
Decontaminate patient (**FOR ADVANCED LEARNERS)**	When toxic exposure is recognized and there is concern of toxic substance on patient’s body, decontaminate by removing contaminated clothing, rinsing if possible. Atropine may be given (IV/IM 0.05–0.1mg/kg/dose; may repeat q 5–10 minutes as needed)

### Round 4: Full scenario without interruption

**If time allows, repeat Round 3 from beginning to end without interruption**.

### END of RCDP session

**Final debriefing and feedback**

- Praise learners
- Provide areas for continued improvement

**Additional Teaching Points for seizure case**

### IV Treatment of Hypoglycemia

- Rule of 50
- % dextrose × mL/kg = 50
- ie, 10% dextrose × _? _ml/kg = 50 (answer: give 5 ml/kg of 10% dextrose)
  25% dextrose × _? _ml/kg = 50 (answer: give 2 ml/kg of 25% dextrose)
  50% dextrose × _?_ ml/kg = 50 (answer: give 1 ml/kg of 50% dextrose)

### Cholinergic Toxicity

- Caused by drugs that function to enhance the effects mediated by acetylcholine
- Examples: Pesticides, organophosphates (cholinesterase inhibitors), carbamates, nerve agents, sarin, physostigmine, mushrooms
- Presentation:
  ○ VS: ↑ ↓ HR, ↑ ↓ BP, Normal or ↓ temp
  ○ Mental status: confusion, lethargy, seizures
  ○ Pupils: miosis or mydriasis
  ○ Skin: wet
  ○ CNS: Agitation, confusion, seizures, restlessness, coma
- Look for muscarinic symptoms (DUMBBELLSS)
  ○ -Diarrhea
  ○ -Urination
  ○ -Miosis
  ○ -Bradycardia
  ○ -Bronchorrhea
  ○ -Emesis
  ○ -Lacrimation (tears)
  ○ -Lethargy
  ○ -Salivation
  ○ -Sweating
- May also see nicotinic symptoms (MTWHF)
  ○ -Mydriasis
  ○ -Tachycardia
  ○ -Weakness
  ○ -Hypertension
  ○ -Fasciculation
- Predominance of central CNS manifestations in childhood insecticide poisoning^1^
- Difficult to recognize muscarinic effects in children (crying, rhinorrhea at baseline^1^
- Organophosphate intoxication in children associated with
  ○ seizures (30%)
  ○ coma (31%)
  ○ respiratory failure (35%)^2^
- **Treatment** **:**
  ○ Protect yourself
  ○ Decontaminate patient^3^,^4^
  ○ ABC’s!: Protect the airway
  ○ **Atropine, Atropine, Atropine:** Muscarinic antagonist, dose 0.05–0.1 mg/kg IV/IM every 5–10 minutes; may need to give > 2 × the recommended dose; titrate until decreased bronchial secretions^(5^
  ○ Expect LARGE, repeat doses
  ○ Note that atropine treats muscarinic but not nicotinic symptoms
  ○ Diazepam for seizures (0.2 mg/kg IV or 0.5 mg/kg per rectum; up to 10 mg/dose max)^6^,^7^

## Appendix L Pediatric Simulation Case: Trauma

**Learner Audience:**
Prehospital providers including emergency medical technicians, registered nurses, respiratory therapists, and physicians at any level of training.
**Time Required for Implementation:**
~ 60 minutes for multiple rounds of RCDP
**Recommended Number of Instructors:**

- 1 simulation instructor/debriefing facilitator per group of three to six learners
- 1 confederate/assistant per group of three to six learners

**Objectives:**
By the end of the session, learners should be able to: Cognitive:

1. Be able to perform International Trauma Life Support (ITLS) primary survey
2. Be able to initiate management of penetrating chest trauma
3. Be able to recognize pediatric hemorrhagic shock and perform early fluid resuscitation
4. Be able to initiate management of extremity trauma
5. Be able to manage continuing assessment of a patient during transport
6. Consider pain management in a trauma patient with IV narcotics

Technical:

1. Perform a rapid primary assessment
2. Perform basic airway management
3. Immobilize the cervical spine immediately and maintain immobilization throughout evaluation
4. Manage a sucking chest wound by application of an occlusive dressing
5. Administer supplemental oxygen
6. Perform IV/IO line placement
7. Perform a rapid secondary survey
8. Apply a splint

Behavioral:

1. Assign team roles
2. Use closed-loop communication
  ○ All messages or orders addressed to specific individuals
  ○ Team members confirm each request and inform the team leader when a task begins or ends
3. Respect each other with language and behavior
  ○ Use proper names with eye contact or appropriate touch
  ○ Share ideas and information as suggestions or constructive interventions, not as criticism
4. Provide a shared mental model (summary of scenario and next steps given to maintain situational awareness)
5. Communicate with receiving facility about transporting an incoming trauma patient

**Case Title:** Pediatric Simulation Case: Trauma

**Equipment or Props Needed:**

Setup for All Rounds:

- Pediatric mannequin
- Equipment to be brought into the scenario by local pre-hospital provider team. In our trials, this included:
  ○ Cardiovascular monitors including blood pressure cuff and oxygen saturation monitor
  ○ Airway management equipment including suction catheter, stethoscope, oropharyngeal airway, nasopharyngeal airway, bag-valve mask (BVM) with various mask sizes, nasal cannula, simple face mask, non-rebreather facemask
  ○ Intravenous (IV) catheter equipment including various size pediatric IV cannulas, 18g needle (or larger) or intraosseous needle, IV tubing
  ○ IV fluid bags labeled for: normal saline (NS) or Ringer’s lactate (LR), dextrose containing fluids (D50, D25, D10, D5)
  ○ Glucometer
  ○ Pediatric length-based tape for estimating weight
  ○ Splinting materials (eg, elastic bandages, planks of hard material)
  ○ Cervical collar (c-collar) and backboard

**Common verbal prompt to start all rounds:** “An 8-year-old male was in a minibus today when it rolled over on the road. He was thrown from the vehicle. He was initially talking at the scene, but as you arrive his mother reports he has become sleepier.”

**Initial Vital Signs for all rounds:** RR 40, HR 140, BP 75/35, O2 85% on RA, T 36.7°C

Weight per Broselow tape

**Initial Physical Exam for all rounds:**

AIRWAY: Airway patent. (Patient mumbles answers to questions, not speaking in full sentences)
BREATHING: Lungs with shallow respirations, clear on left, no breath sounds on right. 2×3 cm open wound to right chest, oozing blood and bubbling with each inspiration
CIRCULATION: No murmur, weak central and peripheral pulses, capillary refill (CR) 4–5 seconds, extremities are cool.
DISABILITY: Opens eyes to voice and answers questions, localizes stimuli. Pupils equal and reactive to light (PERRL) 4→2 mm
OTHER: Bruises to chest and abdomen. Abdomen distended and patient moans with palpation. Deformity to right forearm with palpable pulses (fracture is closed, not open)

### Round 1: Primary survey

**Objectives introduced this round:**

1. Be able to perform ITLS primary survey
2. Perform a rapid primary assessment
3. Immobilize the cervical spine immediately and maintain immobilization throughout evaluation
4. Be able to recognize pediatric hemorrhagic shock

**Verbal Prompt:** “An 8-year-old male was in a minibus today when it rolled over on the road. He was thrown from the vehicle. He was initially talking at the scene, but as you arrive his mother reports he has become sleepier.”

**Vital Signs (if asked):** RR 40, HR 140, BP 75/35, O2 85% on RA, T 36.7°C

Weight per Broselow tape

**Physical exam (if asked):**

AIRWAY: Airway patent. (Patient mumbles answers to questions, not speaking in full sentences)
BREATHING: Lungs with shallow respirations, clear on left, no breath sounds on right. 2×3 cm open wound to right chest, oozing blood and bubbling with each inspiration
CIRCULATION: No murmur, weak central and peripheral pulses, capillary refill (CR) 4–5 seconds, extremities are cool
DISABILITY: Opens eyes to voice and answers questions, localizes stimuli. Pupils equal and reactive to light (PERRL) 4 →2 mm
OTHER: Bruises to chest and abdomen. Abdomen distended and patient moans with palpation. Deformity to right forearm with palpable pulses (fracture is closed, not open)

**Expected actions after verbal prompt:**

- Ensure the scene is safe.
- General impression scan to look for immediate life-threatening problems
- Immediate immobilization of cervical spine.
- Begin primary survey: assessment of airway, breathing, circulation, disability, and exposure of the patient for full evaluation.
- Address each problem encountered in the primary survey sequentially before advancing to the next portion of the survey.
- Verbalize patient is in pediatric hemorrhagic shock based upon vital signs and physical exam.

**Scenario progression:**

- *If oxygen is given, chest wound is covered, and patient is reassessed, patient’s** s**aturation improves to 95%*.
- *If NS or LR bolus is ordered and patient is reassessed after administration of full** b**olus, patient’s BP increases to 92/50 and HR decreases to 110*.

### END Round 1

Review the importance of rapid primary survey. Some actions to stabilize patient may be performed prematurely (ie, objectives of round 2 may be completed in round 1). This is to be expected and facilitators can debrief appropriately, reinforcing the appropriate timing of interventions. If critical actions are NOT addressed in round 1, reinforce that these are critical actions that need to be addressed early in the patient’s presentation.

Review expected actions and teaching points as below:[Table t22-jetem-6-3-c64]

**Table t22-jetem-6-3-c64:**

Expected Action	Teaching Point
Ensure the scene is safe	Off the road, away from fires, etc. Move the patient to a safer area if necessary.
General impression scan for immediate life-threatening situation	If there is an obvious life-threatening problem when scanning the patient (for example, an amputated leg with profuse bleeding), treat immediately before moving into full primary.
Immediate immobilization of cervical spine	This should be the first step in a trauma patient as you begin your assessment. One provider at the head of the patient, one hand on each side of the head, avoid flexion/ extension as well as side-to-side movement. If available, place cervical collar, towel rolls, and/or immobilizing blocks. Scan for neck wounds that would be hidden from view before placing the collar.
Primary survey	Primary survey should be completed in the “ABCDE” order: **A**irway, **B**reathing, **C**irculation, **D**isability, **E**xposure of patient. *Remember, if the patient is ****unresponsive and not moving****, **o**rder changes to C-A-B-D-E*.
Assessment of airway	Ask the patient a question (eg, “what is your name?”). If the answer is clear, the airway is open. Otherwise, listen for air movement.
Assessment of breathing	Listen to breath sounds and watch for chest rise. Are breath sounds symmetric? Are they absent on one side?
Assessment of circulation	Check central and peripheral pulses and perfusion (Capillary refill, warmth of extremities).
Assessment of disability	Do a brief assessment of mental status. Have the patient try to move extremities (ie, “wiggle your fingers/toes).
Exposure of patient for full evaluation	Remove clothing to assess for other injuries.
Begin to address each problem sequentially before moving to next portion of survey	In primary survey, if an emergency with A-B-C is found, begin treatment BEFORE moving along to the remainder of the survey.

### Round 2: Initial stabilization

**Objectives introduced this round:**

1. Perform basic airway management
2. Administer supplemental oxygen
3. Manage a sucking chest wound by application of an occlusive dressing
4. Perform IV/IO line placement

**Restart scenario from beginning**.

**Verbal Prompt:** “An 8-year-old male was in a minibus today when it rolled over on the road. He was thrown from the vehicle. He was initially talking at the scene, but as you arrive his mother reports he has become sleepier.”

**Vital Signs (if asked):** RR 40, HR 140, BP 75/35, O2 85% on RA, T 36.7°C

Weight per Broselow tape

**Physical exam (if asked):**

AIRWAY: Airway patent. (Patient mumbles answers to questions, not speaking in full sentences)
BREATHING: Lungs with shallow respirations, clear on left, no breath sounds on right. 2×3 cm open wound to right chest, oozing blood and bubbling with each inspiration
CIRCULATION: No murmur, weak central and peripheral pulses, capillary refill (CR) 4–5 seconds, extremities are cool
DISABILITY: Opens eyes to voice and answers questions, localizes stimuli. Pupils equal and reactive to light (PERRL) 4→2 mm
OTHER: Bruises to chest and abdomen. Abdomen distended and patient moans with palpation. Deformity to right forearm with palpable pulses (fracture is closed, not open)

**Expected actions after verbal prompt:**

- Ensure the scene is safe.
- General impression scan for immediate life-threatening situation.
- Immediate immobilization of cervical spine.
- Assessment of airway, breathing, circulation, disability, and exposure of patient Address each problem encountered in the primary survey sequentially before advancing to the next portion of the survey.
  ○ Airway:
    ▪ Jaw thrust to maintain c-spine alignment and open airway
  • Breathing
    ▪ Apply oxygen immediately after recognizing problem with airway or breathing
    ▪ Dress chest wound with an occlusive dressing, taped on 3 sides, to prevent tension pneumothorax and allow adequate pulmonary expansion
  • Circulation
    ▪ Obtain IV access
    ▪ If unable to obtain PIV, try IO access
    ▪ Administer 20 ml/kg rapid bolus of isotonic fluid
    ▪ Control active bleeding by applying pressure

**Scenario progression:** *If oxygen is given, chest wound is covered, and patient is reassessed, patient’s saturation improves to 95%*.

*If NS or LR bolus is ordered and patient is reassessed after administration of full bolus, patient’s BP increases to 92/50 and HR decreases to 110*.

### END Round 2

Review the initial exam. Reinforce the importance of immediate intervention when primary survey reveals a problem.

Review expected actions and teaching points as below:[Table t23-jetem-6-3-c64]

**Table t23-jetem-6-3-c64:**

Expected Action	Teaching Point
Jaw thrust	Do not use head tilt/chin lift in a trauma patient, use jaw thrust to maintain c-spine alignment (**demonstrate** on mannequin if this skill is not performed correctly).
Apply oxygen	If airway/breathing problem, apply O2 immediately. If possible, start 100% O2 with NRB facemask, 10–15 Lpm of flow.
Dress chest wound	3 side tape for occlusive dressing. Leave one side open. This allows air to escape, but not enter the chest. This is important, because the risk of a fully occluded chest wound is that air will enter the chest cavity with each breath, leading to a tension pneumothorax, but an open wound will lead to poor lung expansion.
Obtain IV access	Attempt PIV first. In a critically ill trauma patient, if unable to get IV access, try IO access.
Administer bolus Control active bleeding	If poor circulation/ hypotension, give 20 mL/kg bolus of isotonic fluid such as NS or LR. If there is active bleeding, apply manual pressure.

### Round 3: Rapid transport and secondary survey

**Objectives introduced this round:**

1. Manage continuing assessment of a patient during transport
2. Be able to initiate management of extremity trauma by applying a splint
3. Consider pain management in a trauma patient.

**Restart scenario from beginning**.

**Verbal Prompt:** “An 8-year-old male was in a minibus today when it rolled over on the road. He was thrown from the vehicle. He was initially talking at the scene, but as you arrive his mother reports he has become sleepier.”

**Vital Signs (if asked):** RR 40, HR 140, BP 75/35, O2 85% on RA, T 36.7°C

Weight per Broselow tape

**Physical Exam (if asked):**

AIRWAY: Airway patent. (Patient mumbles answers to questions, not speaking in full sentences)
BREATHING: Lungs with shallow respirations, clear on left, no breath sounds on right. 2×3 cm open wound to right chest, oozing blood and bubbling with each inspiration
CIRCULATION: No murmur, weak central and peripheral pulses, capillary refill (CR) 4–5 seconds, extremities are cool
DISABILITY: Opens eyes to voice and answers questions, localizes stimuli. Pupils equal and reactive to light (PERRL) 4→2 mm
OTHER: Bruises to chest and abdomen. Abdomen distended and patient moans with palpation. Deformity to right forearm with palpable pulses (fracture is closed, not open)

**Expected actions after verbal prompt:**

- Ensure the scene is safe.
- General impression scan for immediate life-threatening situation.
- Immediate immobilization of cervical spine.
- Assessment of airway, breathing, circulation, disability, and exposure of patient. Address each problem encountered in the primary survey sequentially before advancing to the next portion of the survey.
  ○ Airway:
    ▪ Jaw thrust to maintain c-spine alignment
    ▪ Apply oxygen immediately
  • Breathing
    ▪ Dress chest wound to prevent tension pneumothorax
  • Circulation
    ▪ Obtain IV access
    ▪ If unable to obtain PIV, try IO access.
    ▪ Administer 20 ml/kg bolus of isotonic fluid
    ▪ Control active bleeding by applying pressure

Consider treatment of the patient’s pain with IV narcotic

**Scenario progression:** *If oxygen is given, chest wound is covered, and patient is reassessed, patient’s saturation improves to 95%*.

*If NS or LR bolus is ordered and patient is reassessed after administration of full bolus, patient’s BP increases to 92/50 and HR decreases to 110*.

**Expected actions after scenario progression:**

- Transport to hospital *where surgical services are available* as soon as immediate life-threatening problems are addressed (ABCs).
- Complete secondary survey (examine head to toe) while in ambulance.
- Splint forearm.
  ○ Check pulses distal to fracture before and after splinting.
- Start IV analgesic medication to treat pain.
- Call receiving facility during transport to notify them about a critical trauma patient on the way.

### END Round 3

Review the importance of minimizing scene time and transporting to hospital. Further evaluation and interventions can continue in the ambulance.

Review expected actions and teaching points as below:[Table t24-jetem-6-3-c64]

**Table t24-jetem-6-3-c64:**

Expected Action	Teaching Point
Transport to hospital as soon as immediate life-threatening problems are addressed (ABCs)	The most critical time for trauma patients is the first hour, particularly for those with internal bleeding. Secondary survey and splinting of fractures can occur in the ambulance (the exception to this are femur and pelvic fractures since they can be associated with a large amount of internal bleeding). Transport to a hospital where you know surgical services are available.
Complete secondary survey	Perform a quick screen of the rest of the body, head to toe, while in ambulance (if possible) while en route to receiving hospital.
Splint forearm	Use materials available, splint in current position, avoid taping directly over the area with deformity. Check pulses distal to fracture before and after splinting.
Pain management	It is important to treat pain in patients who have undergone trauma. Treat with IV formulation of analgesic medication.
Call ahead with critical trauma patient	Call receiving facility when you are transporting a critical trauma patient to notify the care team that the patient is on the way.

### Round 4: Full scenario without interruption

**Depending on time available, repeat round 3 from the beginning without interruption. If**** i****nitial actions are performed well, restart where needed**.

*Note: The scenario can be altered so that instead of a sucking chest wound, the patient has a** r**ight sided pneumothorax that requires needle decompression. The patient will not have an** o**pen chest wound and will instead have decreased breath sounds on the right side noted on** a**uscultation. A large bore needle can be inserted between the second and third intercostal** s**pace (above the third rib) at the midclavicular line or into the fourth intercostal space at the** a**nterior axillary line. The patient’s breathing should improve and breath sounds should** i**mprove on auscultation after needle decompression*.

### END OF RCDP Session

**Final debriefing and feedback**

- Praise learners
- Provide areas for continued improvement

## Appendix M Simulation Course for Prehospital Providers Course Evaluation

1. Please rate the course on a scale of 1–5 (please select one):[Table t25-jetem-6-3-c64]
  **Table t25-jetem-6-3-c64:**
  Not useful	Somewhat Useful	Useful	Very Useful	Extremely Useful
  1	2	3	4	5
2. What was the best part of the course?
3. What changes would you make to the course?
4. Please comment about the specific instructors:
  [Name]:
  [Name]:
  [Name]:
  [Name]:
5. Any other comments?

## Appendix N Equipment Setup for Simulation Scenarios

Based on Botswana EMS Response Bag

**Fluids/Access:**

□ Intravenous (IV) cannulas of various sizes (18, 20, and 22 gauge)
□ 1 × 45 mm intraosseous needle (14 gauge or 16 gauge regular can be used instead)
□ 1 × 25 mm intraosseous needle (14 gauge or 16 gauge regular can be used instead)
□ 5–10 × syringes of various volumes (3, 5, 10, 20, 50 mL)
□ 1–2 rubber tourniquets
□ 1 × roll of cloth tape
□ 1 × razor blade or 1 pair of scissors
□ IV fluids:
□ 1 × adult IV infusion set
□ 1 × pediatric IV infusion set
□ 1-liter bag of Ringer’s lactate
□ 1-liter bag of saline
□ 1 × 500 mL bag of dextrose, 10% saline
□ 1 × 500 mL bag of dextrose, 5% saline
□ 1 × ampule of dextrose 50
□ 2 × 10 mL sterile water for injection

**Airway equipment:**

□ 1 × adult-size, pediatric-size, infant-size simple face mask
□ 1 × adult-size, pediatric-size, infant-size non-rebreather face mask
□ 1 × adult-size, pediatric-size, infant-size nebulizer mask
□ 1 × of each adult-size, pediatric-size, infant-size nasal cannula
□ 1 × of each adult-size, pediatric-size, infant-size bag-valve and mask
□ 1 × non-flexible suction tip
□ 2 × flexible suction catheters of various sizes
□ 1 set of oropharyngeal airways (infant to adult sizes)
□ 1 set of nasopharyngeal airways (infant to adult sizes)
□ Multiple endotracheal tubes of various sizes (3.0–7.5, including sizes)
□ 1 × pulse oximeter
□ 1 × size 1, 2, 3 straight laryngoscope blades
□ 1 × size 1, 2, 3, 4, 5 curved laryngoscope blades
□ 1 × size 1, 3, 4 laryngeal mask airways

**Medications:**

□ 1 × albuterol
□ 1 × paracetamol
□ 1 × diazepam

**Trauma equipment:**

□ 1 × spine immobilization board with stretcher straps
□ 1 × head blocks and securing straps

**Orthopedic splinting material (**Can be cardboard, padded boards, or other polymer malleable splinting materials)

□ 1 × trauma tourniquet
□ 1 × each adjustable cervical collars (infant, pediatric, and adult)

**Miscellaneous equipment:**

□ 1 × pediatric length-based tape to estimate weight
□ Disposable gloves of various sizes
□ 10 × gauze pads of various sizes
□ 5 × plastic bandage wraps of various widths
□ 1 × container of glucose test strips
□ 1 × glucometer
□ 1 × adult, pediatric, infant blood pressure cuff
□ 1 × stethoscope
□ 1 × thermometer
□ 3 × wooden tongue blades
□ 1 × small flashlight
□ 5 towels of various sizes
□ 1 × umbilical cord clamp

## Appendix O Debriefing Techniques

[Table t26-jetem-6-3-c64]

**Table t26-jetem-6-3-c64:**

Directive Feedback	Learner Self-Assessment (plus/delta)	Advocacy-Inquiry
Immediate, instructor-driven feedback on performance gaps and learning objectives	Exploration of learner-centric performance gaps and learning objectives	Learner-focused facilitation related to instructor-driven performance gaps and learning objectives
*“I noticed you did (**actual action**)*. *Next time you may want to try **d**oing (**desired action**) because* (*rationale**).”* *“I liked how you did (**praise point**)*. *Next time continue to do that **b**ecause (**rationale**).”*	*“What aspects of (* *performance* *) * *d* *o you think you’d like to do* * d* *ifferently?”* *What aspects of (* *performance* *) do * *y* *ou think you managed well?”*	*“I noticed (**actual action**). I was **c**oncerned because (**rationale**)*. *How do you see it?”* *I saw how you (**desired action**). I **l**iked that because (**appreciation**)*. *What was your take on it?”*

## Appendix P Commonly Used Abbreviations

[Table t27-jetem-6-3-c64]

**Table t27-jetem-6-3-c64:**

ABC	Airway, Breathing, Circulation
A&E	Accident and emergency
AVPU.	Alert, Voice, Pain, Unresponsive
BMV	Bag-Mask Ventilation
BP	Blood Pressure
BVM	Bag-Valve Mask
C-collar	Cervical collar
CPR	Cardiopulmonary Resuscitation
CR	Capillary refill
D10	Dextrose 10%
D25	Dextrose 25%
D5	Dextrose 5%
D50	Dextrose 50%
D5W	Dextrose 5% Water
EKG	Electrocardiogram
EMS	Emergency Medical Services
EMT	Emergency Medical Technician
HR	Heart Rate
IM	Intramuscular
IN	Intranasal
IO	Intraosseous
IV	Intravenous
LMIC	Low Middle Income Country
LR	Lactated Ringer’s
MOH	Ministry of Health
MOHW	Ministry of Health and Wellness
NS	Normal Saline
O2	Oxygen
PERRL	Pupils Equally Round and Reactive to Light
PO	By mouth (per os)
PPV	Positive Pressure Ventilation
RA	Room Air
RCDP	Rapid Cycle Deliberate Practice
RR	Respiratory Rate
T	Temperature
Wt	Weight

## Appendix Q Lecture 1—Trauma Evaluation

Please see associated PowerPoint file

## Appendix R Lecture 2—Introduction to Simulation

Please see associated PowerPoint file

## Appendix S Lecture 3—Delivery and Neonatal Resuscitation

Please see associated PowerPoint file

## Appendix T Lecture 4—EMS Pediatric Assessment

Please see associated PowerPoint file

## Appendix U EMS Pediatric Training Course Schedule

[Table t28-jetem-6-3-c64] [Table t29-jetem-6-3-c64]

**Table t28-jetem-6-3-c64:**

Day 1 Schedule	
**08:00–09:00**	Introduction to simulation didactic
**09:00–11:00**	Pretest-written test and simulation test, with tea available during testing breaks (rotating schedule created depending on # of participants)
**11:00–12:00**	Introduction EMS Pediatric Assessment didactic
**12:00–1:00**	Lunch break
**13:00–13:30**	Basic airway skills station
**13:30–14:00**	IV/ IO access skills station
**14:00–14:30**	Chest compressions/ CPR skills station
**14:30–14:45**	Break
**14:45–15:45**	Group 1: Simulation 1 (peds respiratory: asthma) Group 2: Simulation 2 (peds shock: diarrhea/hypovolemic shock)
**15:45–16:45**	Group 1: Simulation 2 (peds shock: diarrhea/hypovolemic shock) Group 2: Simulation 1 (peds respiratory: asthma)

**Table t29-jetem-6-3-c64:**

Day 2 Schedule	
**08:00–09:00**	Newborn delivery and resuscitation didactic
**09:00–10:00**	Group 1: Newborn resuscitation simulation Group 2: Newborn resuscitation simulation
**10:00–10:15**	Break
**10:15–10:45**	Trauma didactic
**10:45–11:45**	Group 1: Simulation 1 (pediatric trauma) Group 2: Simulation 2 (peds tox/sz)
**11:45–12:45**	Lunch break
**12:45–13:45**	Group 1: Simulation 2 (peds tox/sz) Group 2: Simulation 1 (pediatric trauma)
**13:45–14:00**	Break
**14:00–16:00**	Post-test-written and simulation, with tea available during testing breaks (rotating schedule created depending on # of participants)
