# Supplementary material for: Pediatric Simulation-Based Prehospital Training Course in Botswana
Source: J Educ Teach Emerg Med. 2021 Jul 15;6(3):C64–C189. doi: 10.21980/J8306S (PMC10332686; doi:10.21980/J8306S)
Supplement: Supplementary file 1 — Please see associated PowerPoint file [file jetem-6-3-c64-AppendixQ.pptx]

## Slide 1
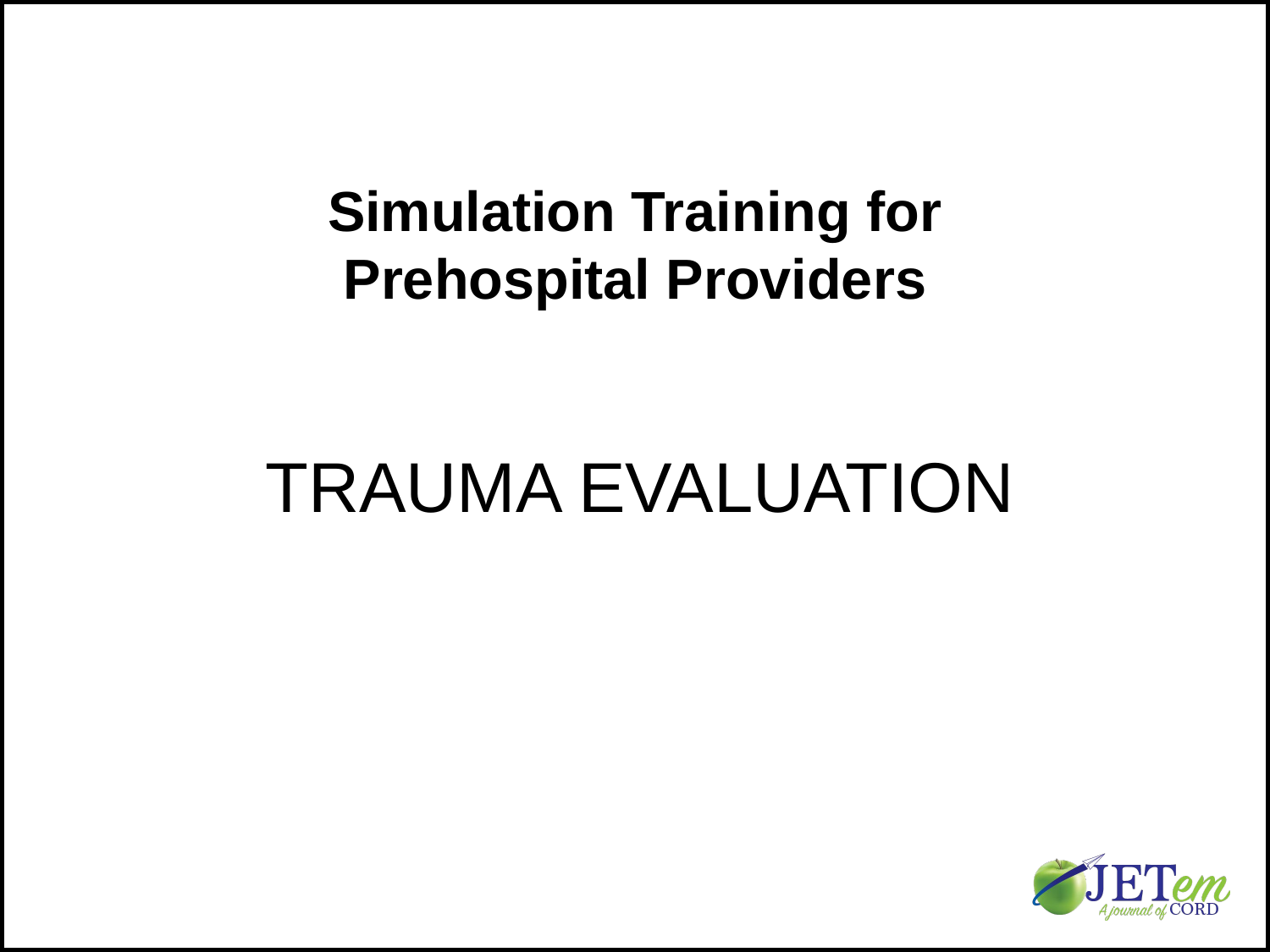

# Simulation Training for Prehospital Providers
TRAUMA EVALUATION

## Slide 2
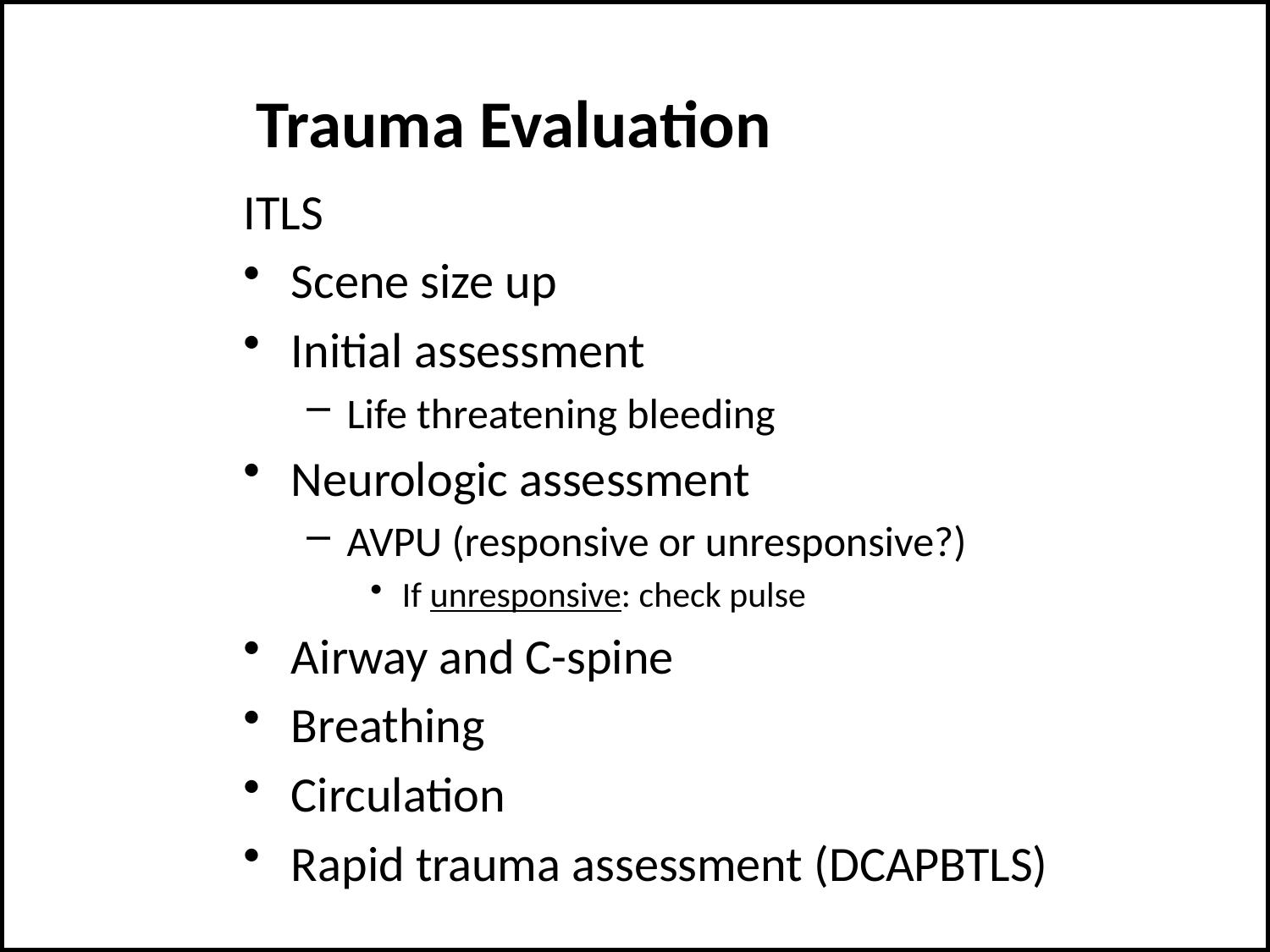

# Trauma Evaluation
ITLS
Scene size up
Initial assessment
Life threatening bleeding
Neurologic assessment
AVPU (responsive or unresponsive?)
If unresponsive: check pulse
Airway and C-spine
Breathing
Circulation
Rapid trauma assessment (DCAPBTLS)

## Slide 3
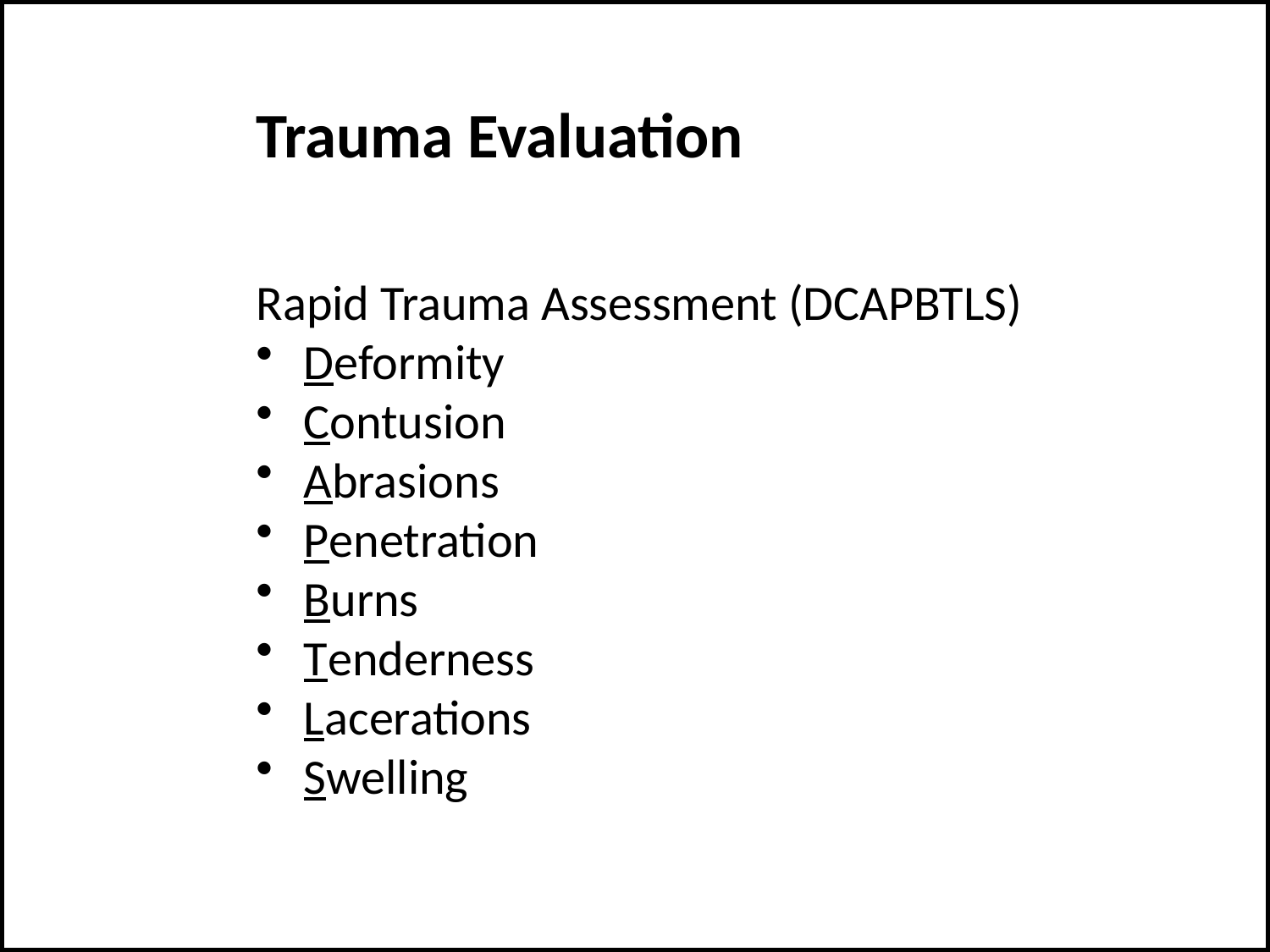

# Trauma Evaluation
Rapid Trauma Assessment (DCAPBTLS)
Deformity
Contusion
Abrasions
Penetration
Burns
Tenderness
Lacerations
Swelling
