# Supplementary material for: Pediatric Simulation-Based Prehospital Training Course in Botswana
Source: J Educ Teach Emerg Med. 2021 Jul 15;6(3):C64–C189. doi: 10.21980/J8306S (PMC10332686; doi:10.21980/J8306S)
Supplement: Supplementary file 2 — Please see associated PowerPoint file [file jetem-6-3-c64-appendixR.pptx]

## Slide 1
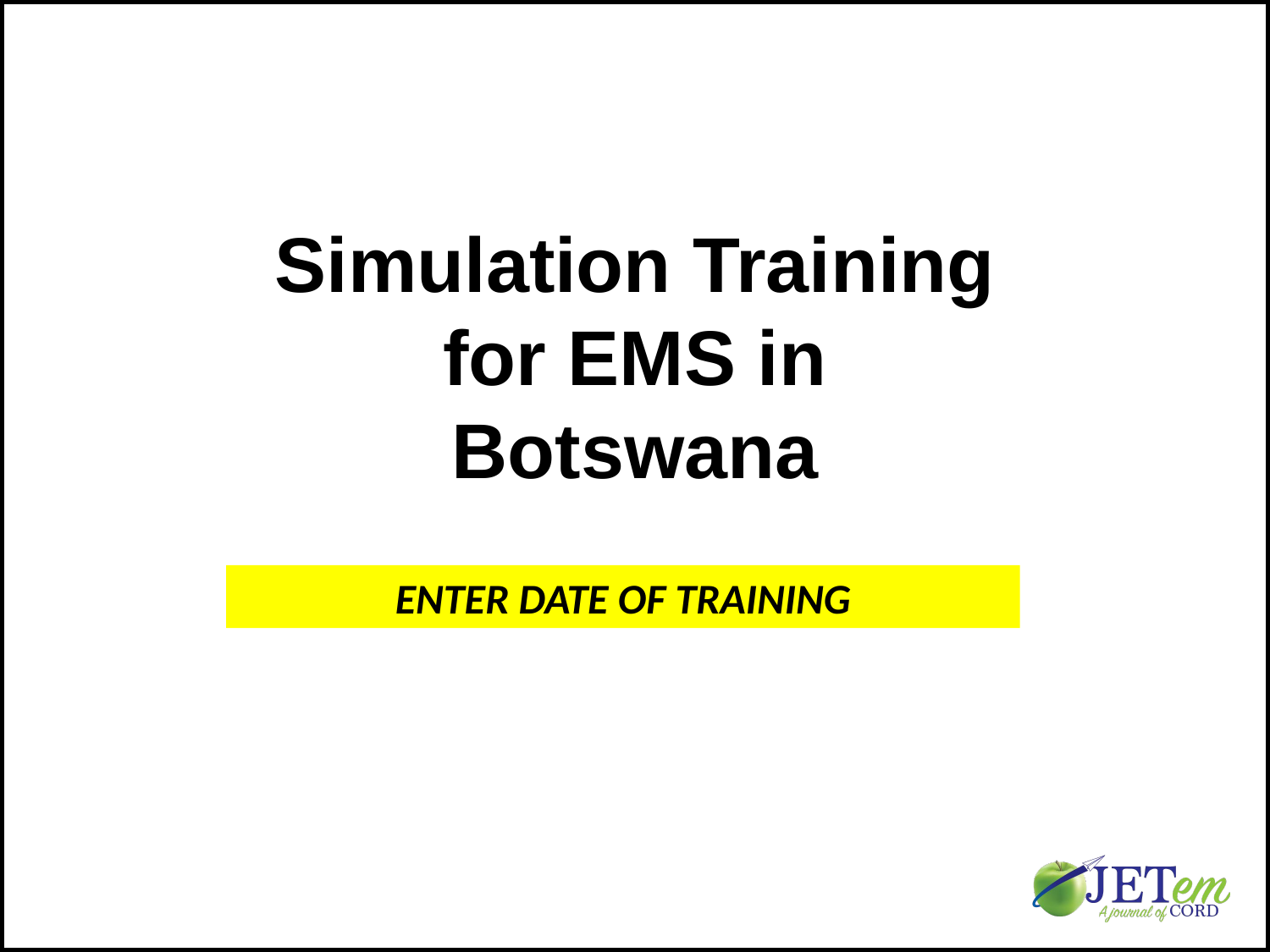

# Simulation Training for EMS in Botswana
ENTER DATE OF TRAINING

## Slide 2
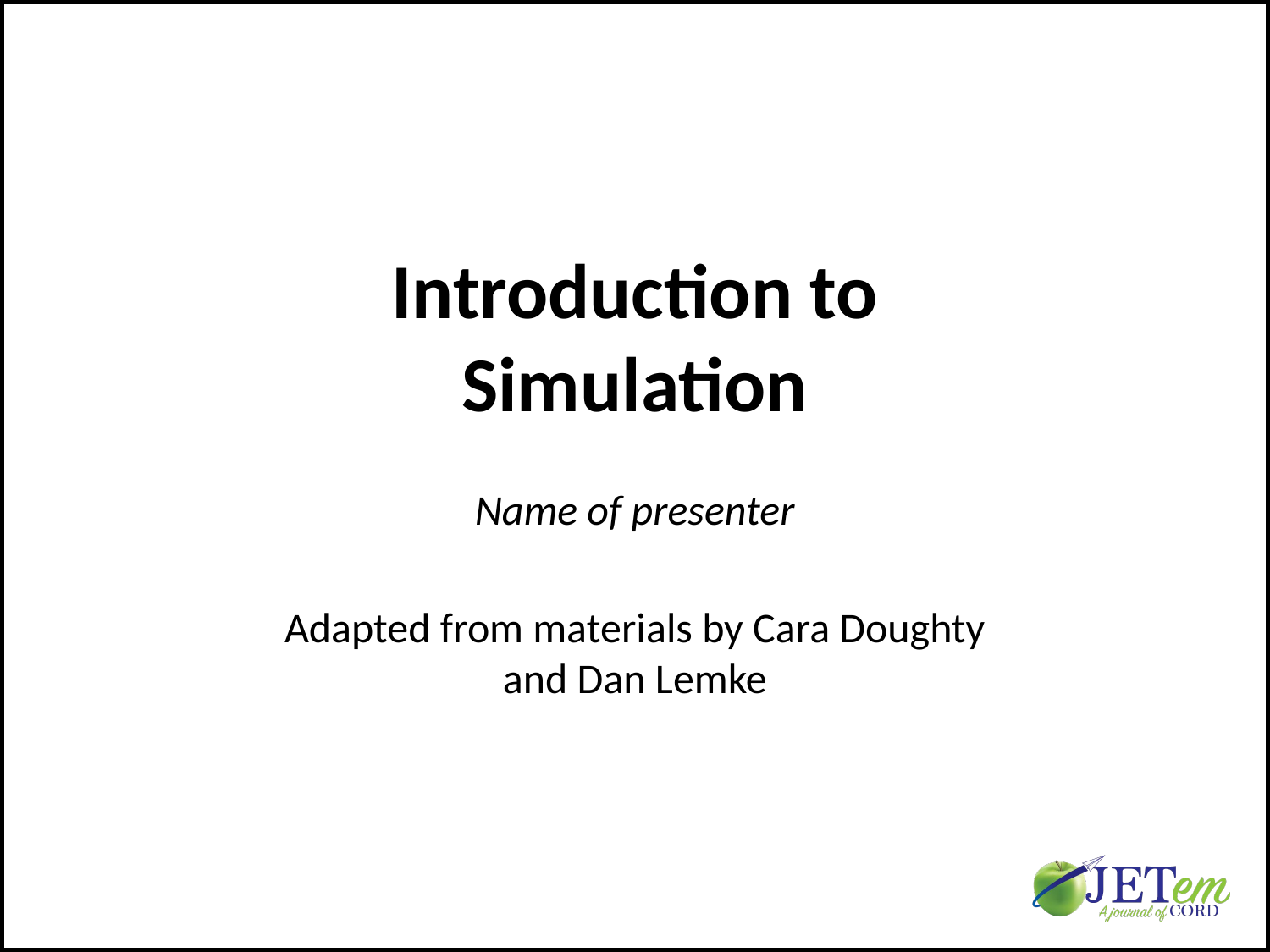

# Introduction to Simulation
Name of presenter
Adapted from materials by Cara Doughty and Dan Lemke

## Slide 3
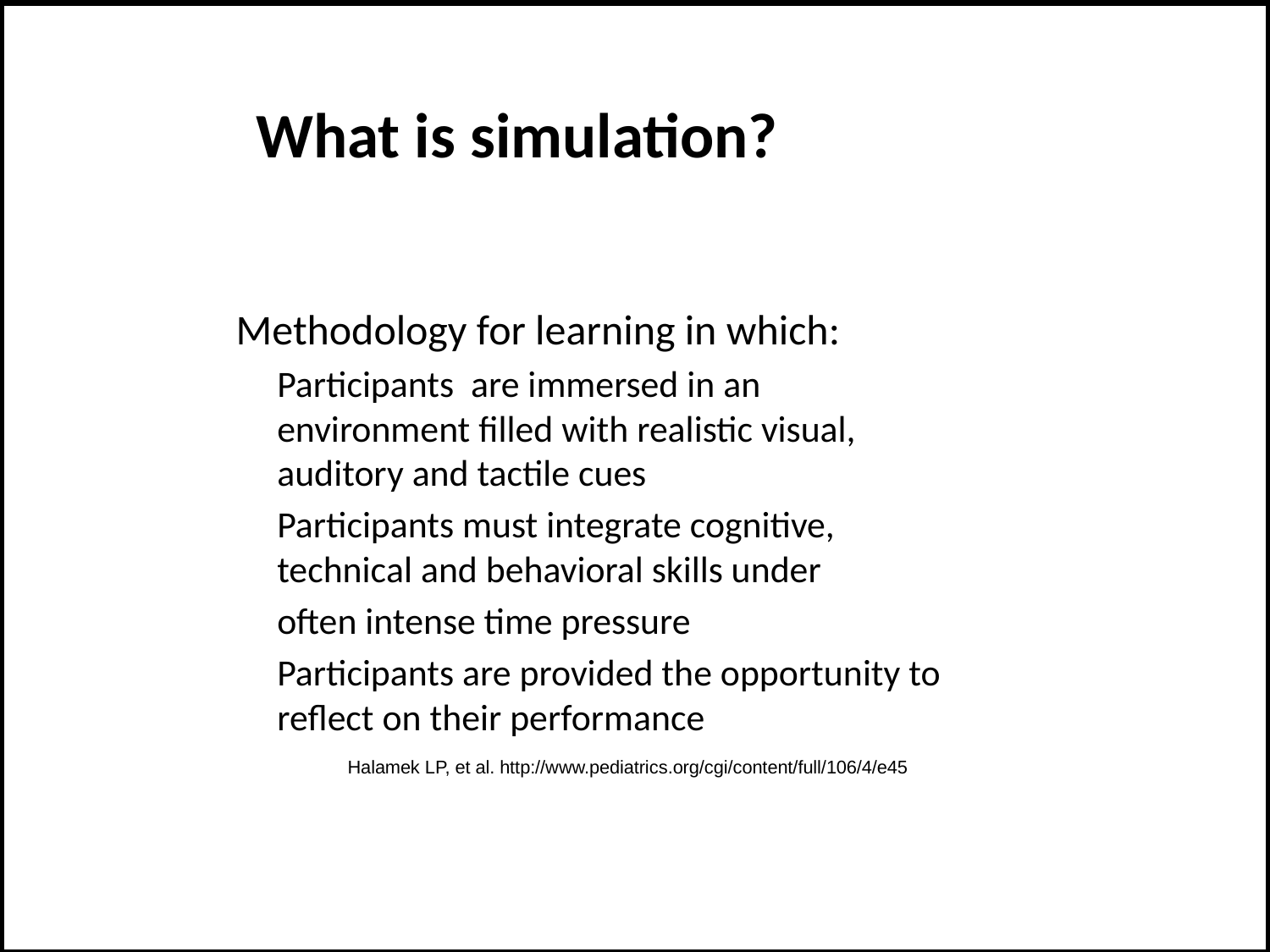

# What is simulation?
Methodology for learning in which:
Participants are immersed in an environment filled with realistic visual, auditory and tactile cues
Participants must integrate cognitive, technical and behavioral skills under
often intense time pressure
Participants are provided the opportunity to reflect on their performance
Halamek LP, et al. http://www.pediatrics.org/cgi/content/full/106/4/e45

## Slide 4
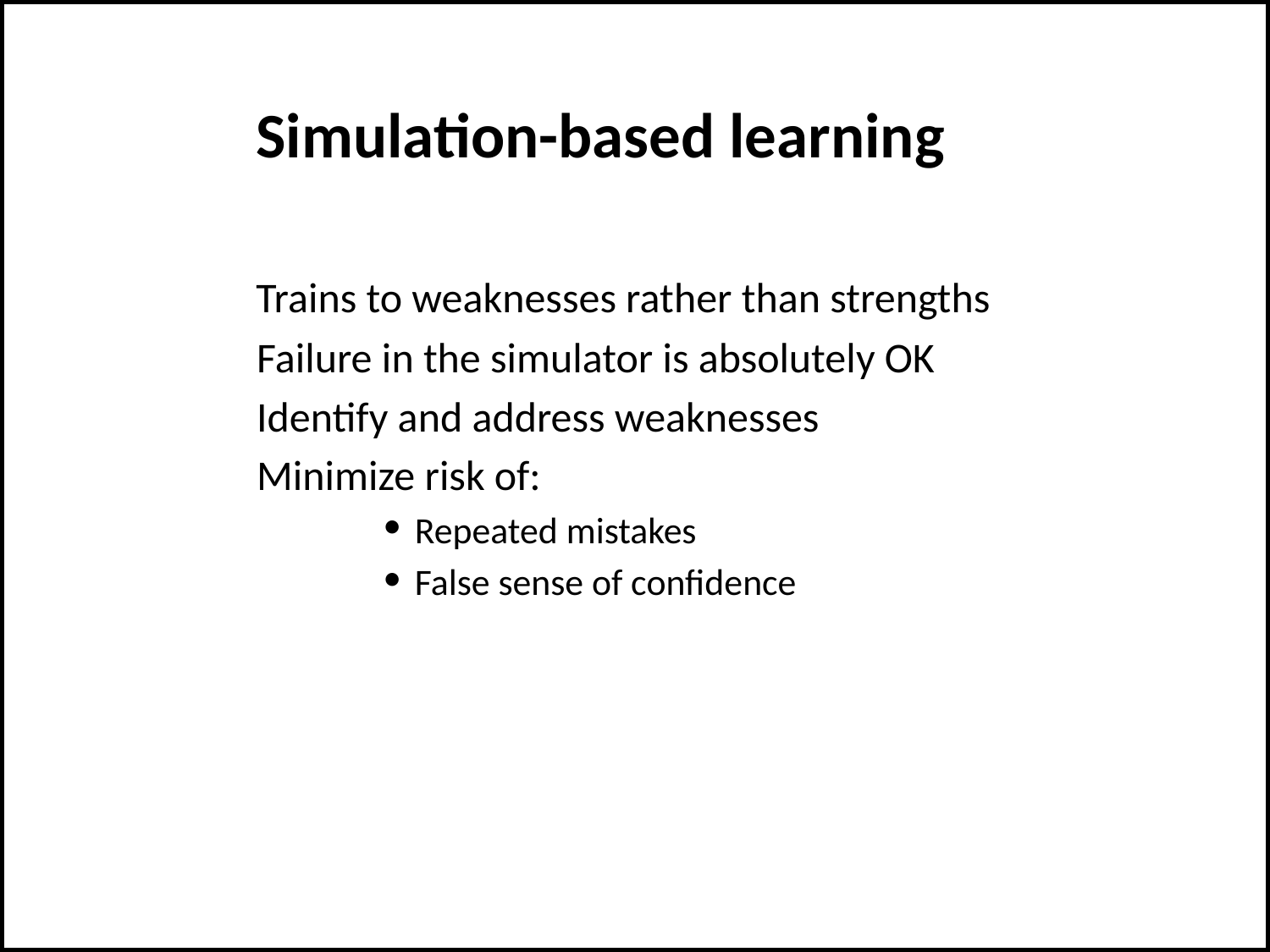

# Simulation-based learning
Trains to weaknesses rather than strengths
Failure in the simulator is absolutely OK
Identify and address weaknesses
Minimize risk of:
Repeated mistakes
False sense of confidence

## Slide 5
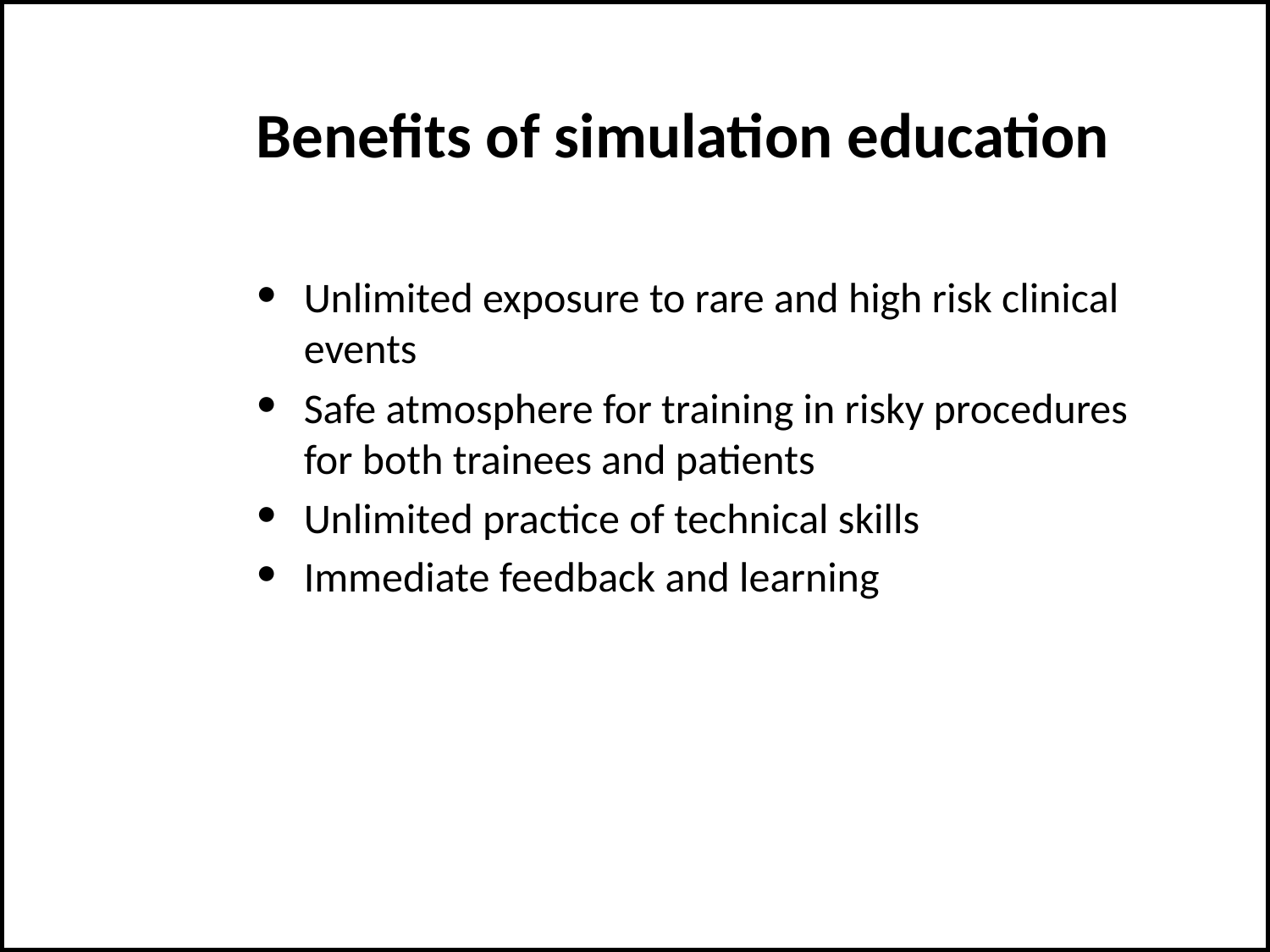

# Benefits of simulation education
Unlimited exposure to rare and high risk clinical events
Safe atmosphere for training in risky procedures for both trainees and patients
Unlimited practice of technical skills
Immediate feedback and learning

## Slide 6
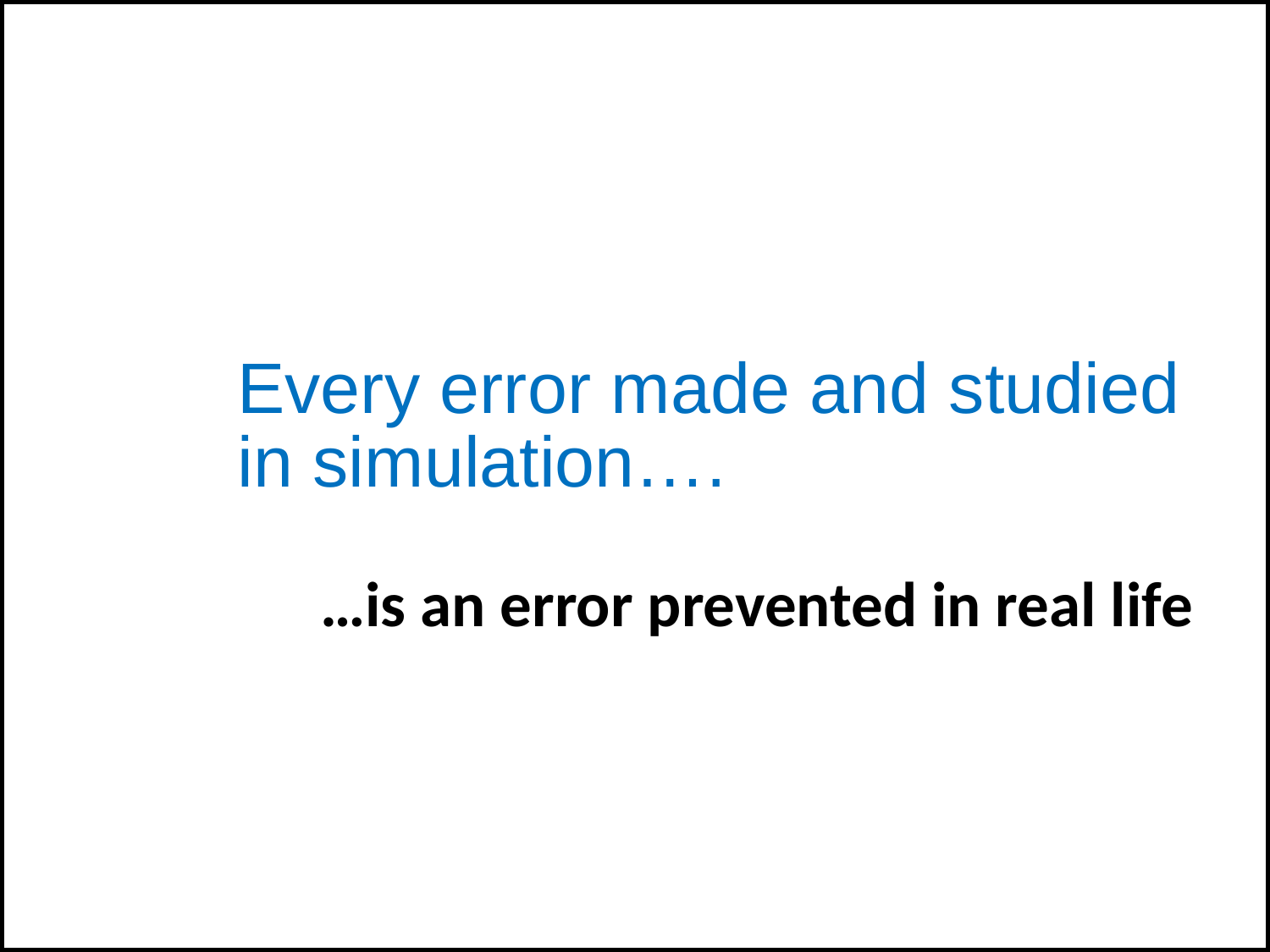

Every error made and studied in simulation….
# …is an error prevented in real life

## Slide 7
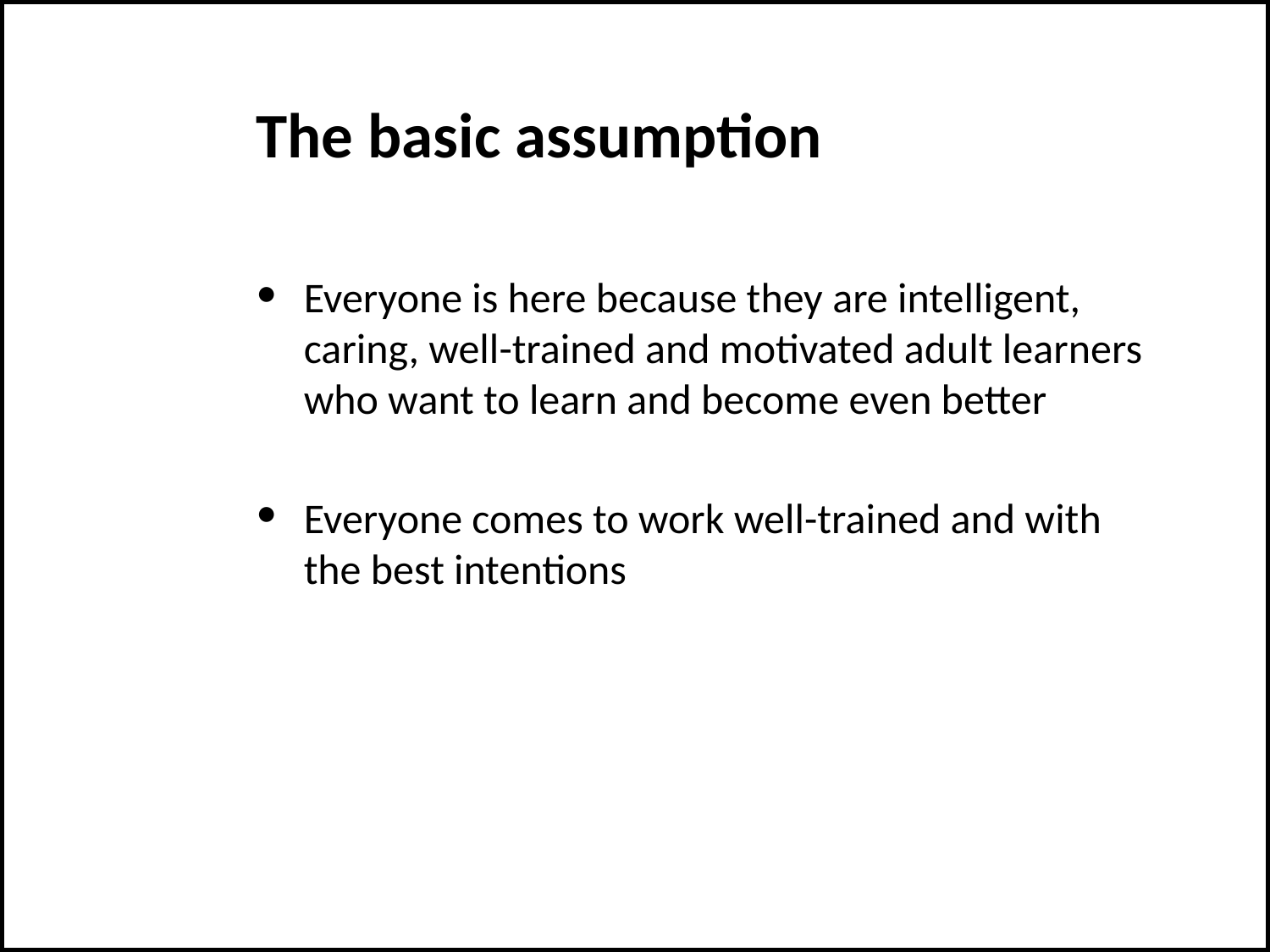

# The basic assumption
Everyone is here because they are intelligent, caring, well-trained and motivated adult learners who want to learn and become even better
Everyone comes to work well-trained and with the best intentions

## Slide 8
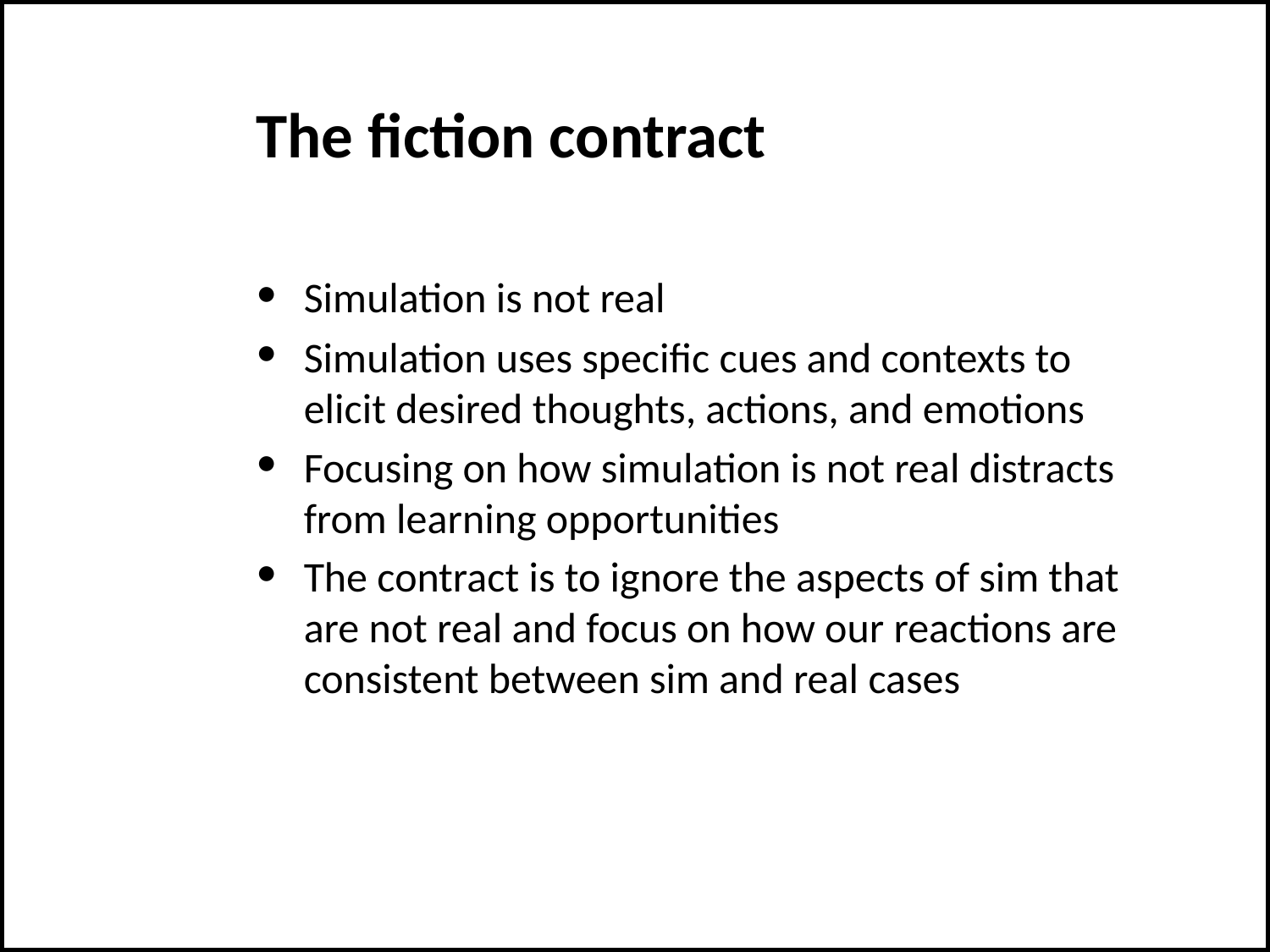

# The fiction contract
Simulation is not real
Simulation uses specific cues and contexts to elicit desired thoughts, actions, and emotions
Focusing on how simulation is not real distracts from learning opportunities
The contract is to ignore the aspects of sim that are not real and focus on how our reactions are consistent between sim and real cases

## Slide 9
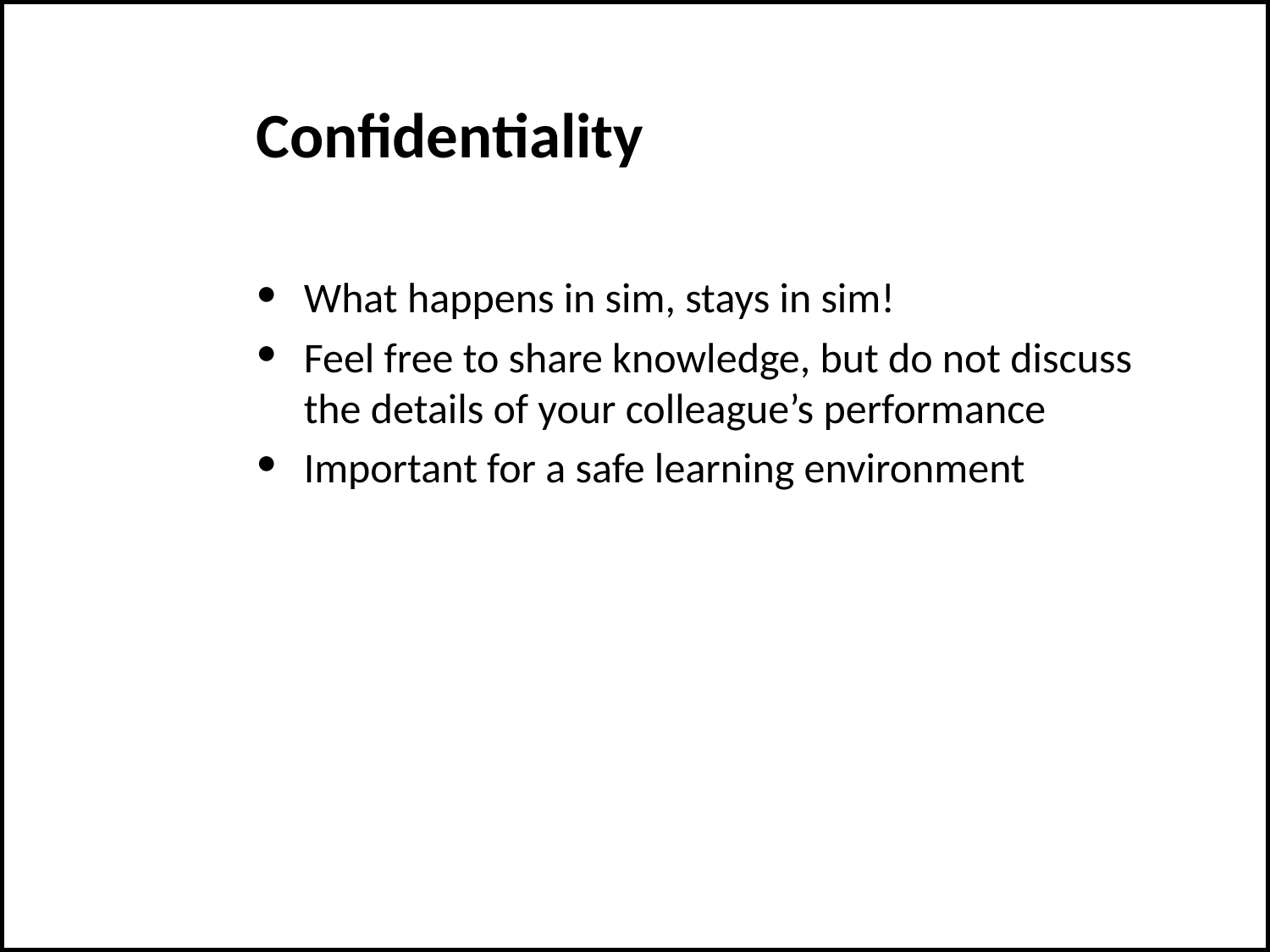

# Confidentiality
What happens in sim, stays in sim!
Feel free to share knowledge, but do not discuss the details of your colleague’s performance
Important for a safe learning environment

## Slide 10
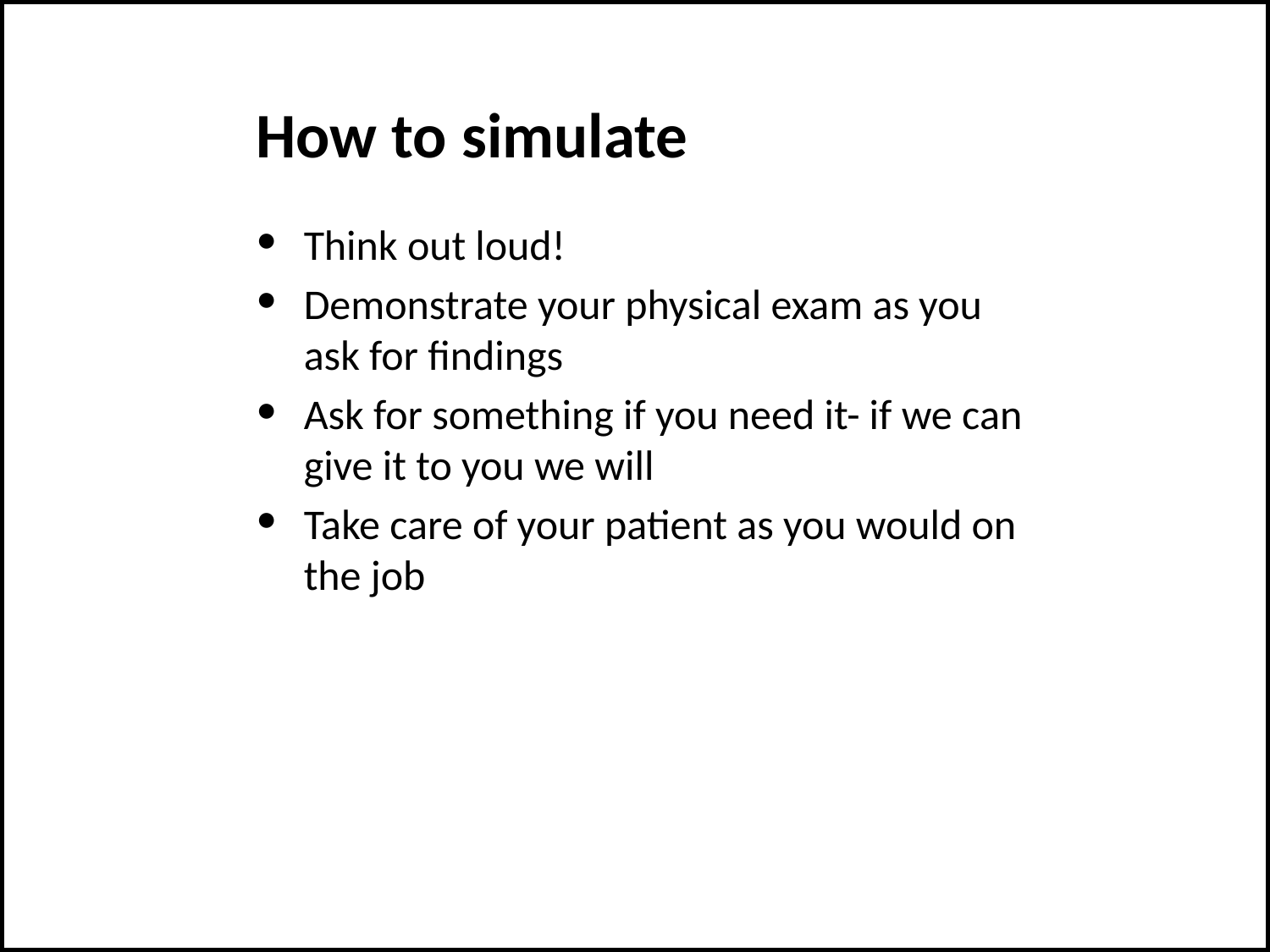

# How to simulate
Think out loud!
Demonstrate your physical exam as you ask for findings
Ask for something if you need it- if we can give it to you we will
Take care of your patient as you would on the job

## Slide 11
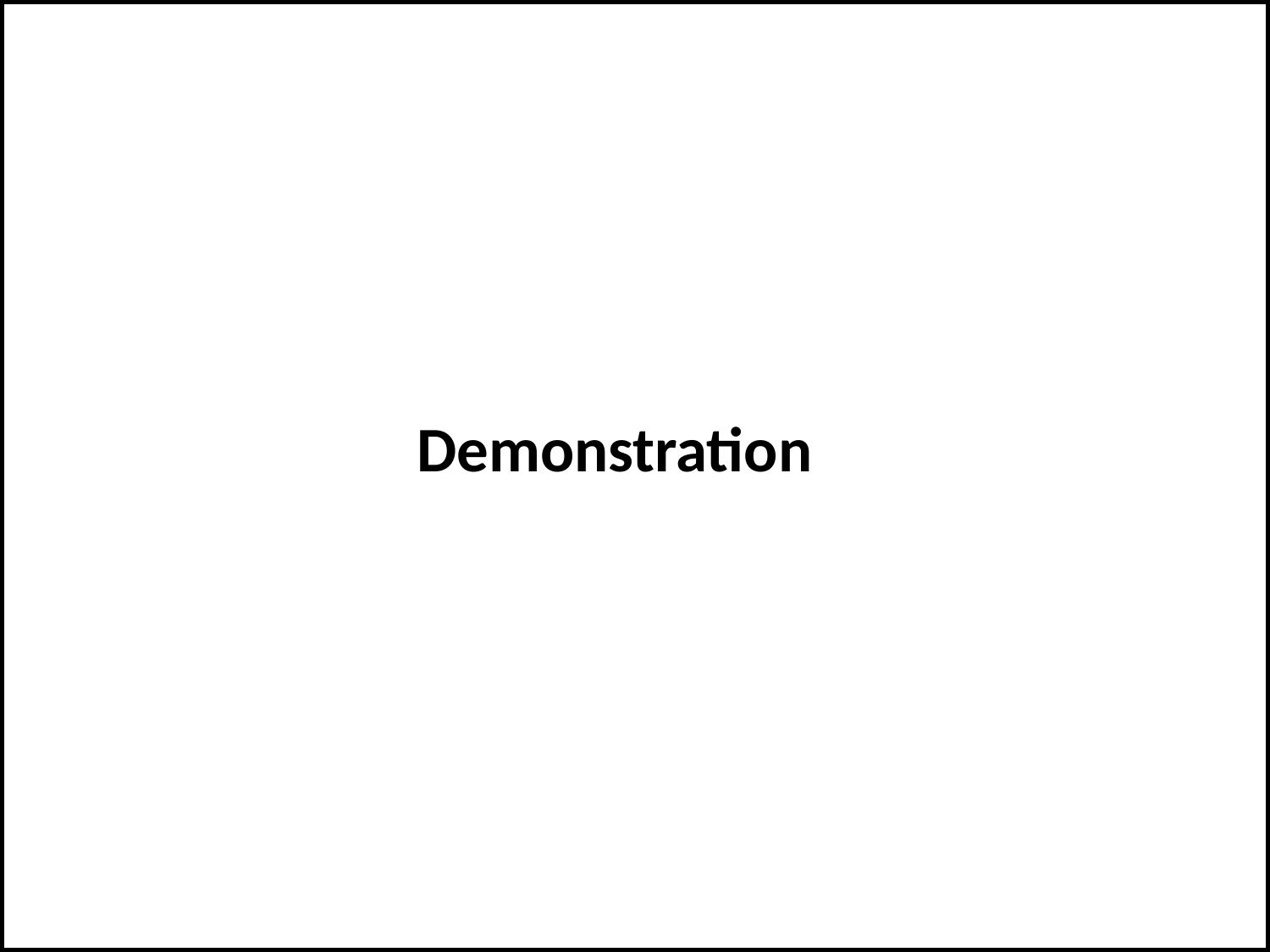

# Demonstration
