# Supplementary material for: Pediatric Simulation-Based Prehospital Training Course in Botswana
Source: J Educ Teach Emerg Med. 2021 Jul 15;6(3):C64–C189. doi: 10.21980/J8306S (PMC10332686; doi:10.21980/J8306S)
Supplement: Supplementary file 3 — Please see associated PowerPoint file [file jetem-6-3-c64-appendixS.pptx]

## Slide 1
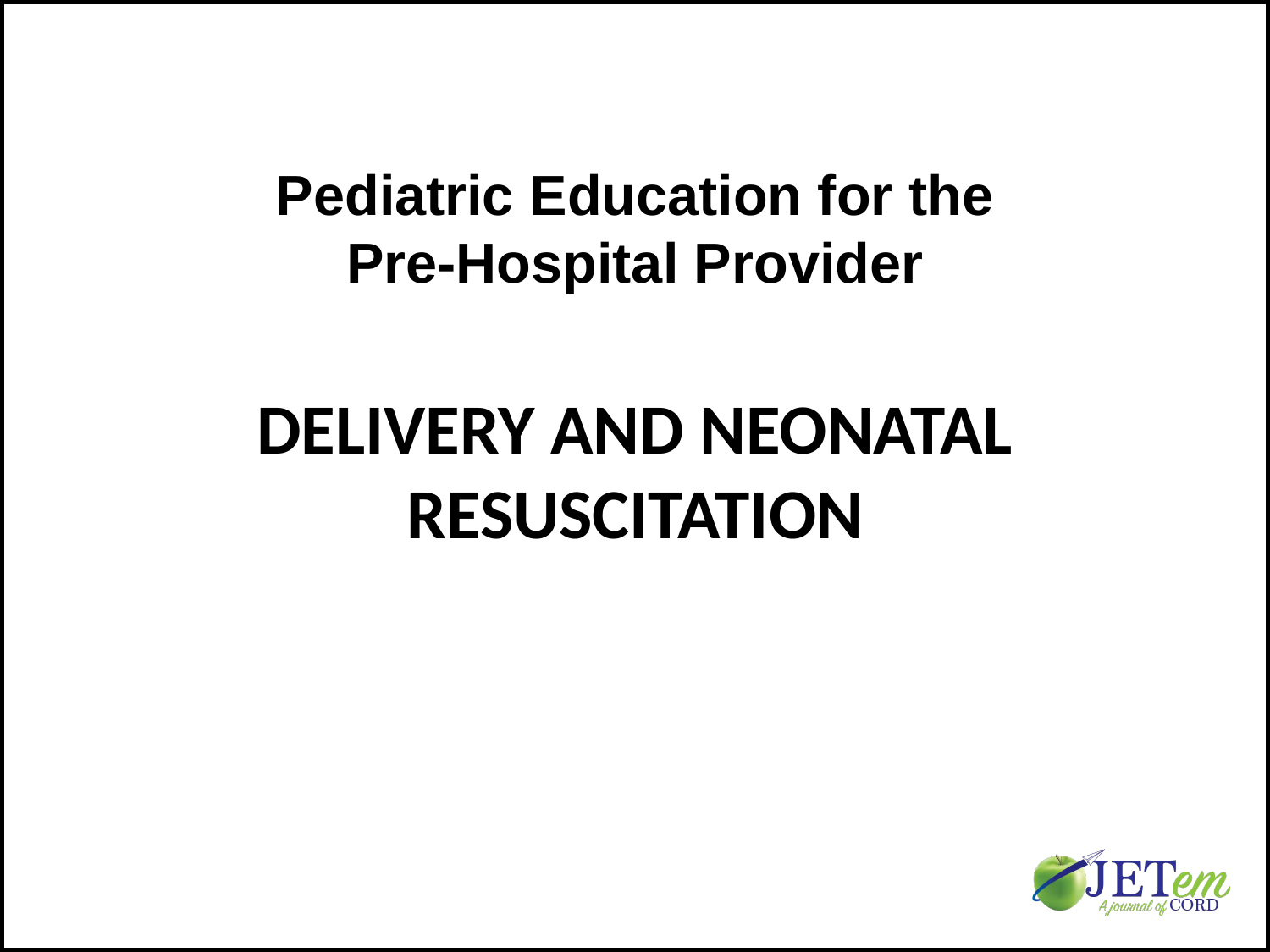

# Pediatric Education for the Pre-Hospital Provider
DELIVERY AND NEONATAL RESUSCITATION

## Slide 2
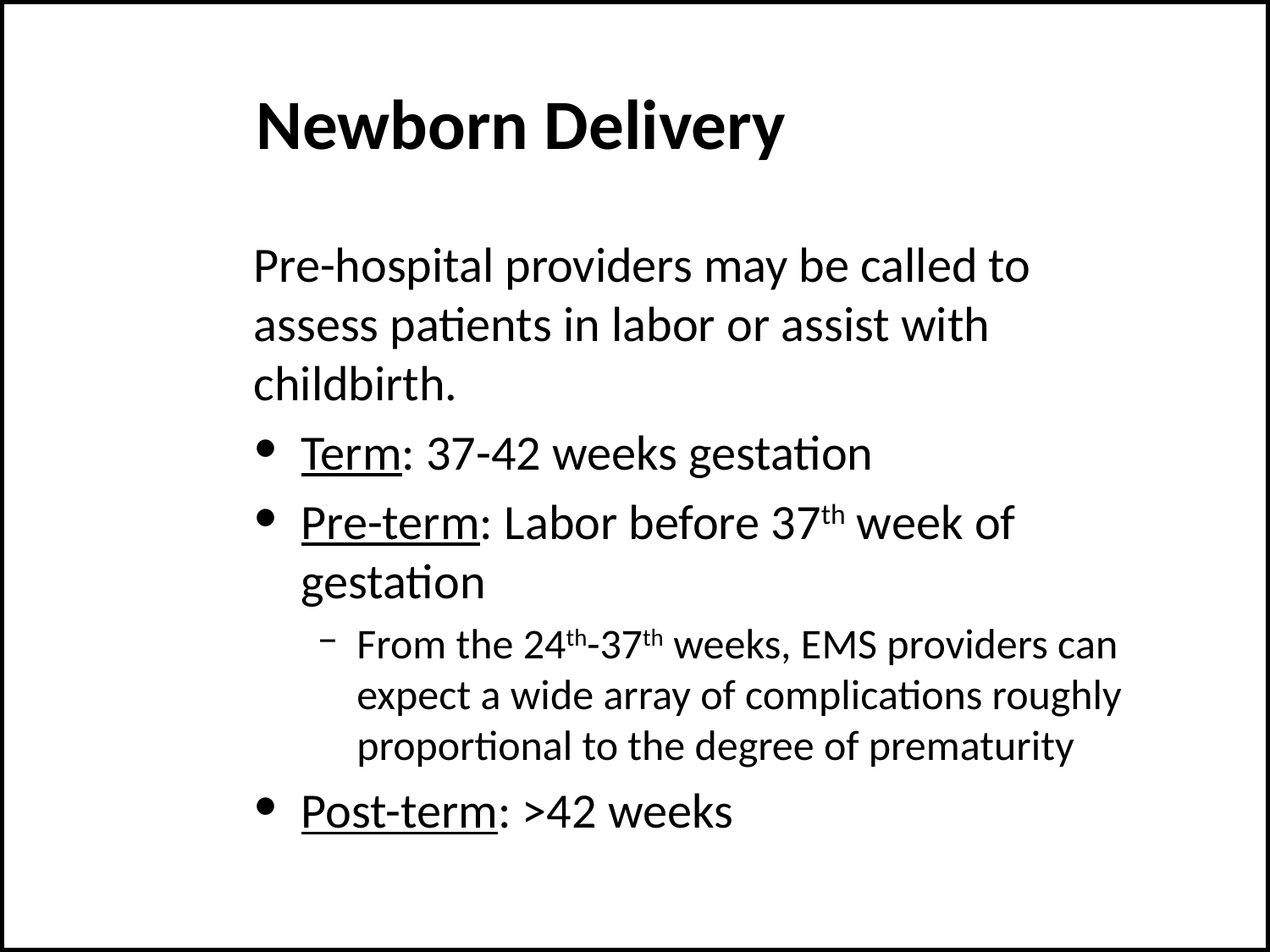

# Newborn Delivery
Pre-hospital providers may be called to assess patients in labor or assist with childbirth.
Term: 37-42 weeks gestation
Pre-term: Labor before 37th week of gestation
From the 24th-37th weeks, EMS providers can expect a wide array of complications roughly proportional to the degree of prematurity
Post-term: >42 weeks

## Slide 3
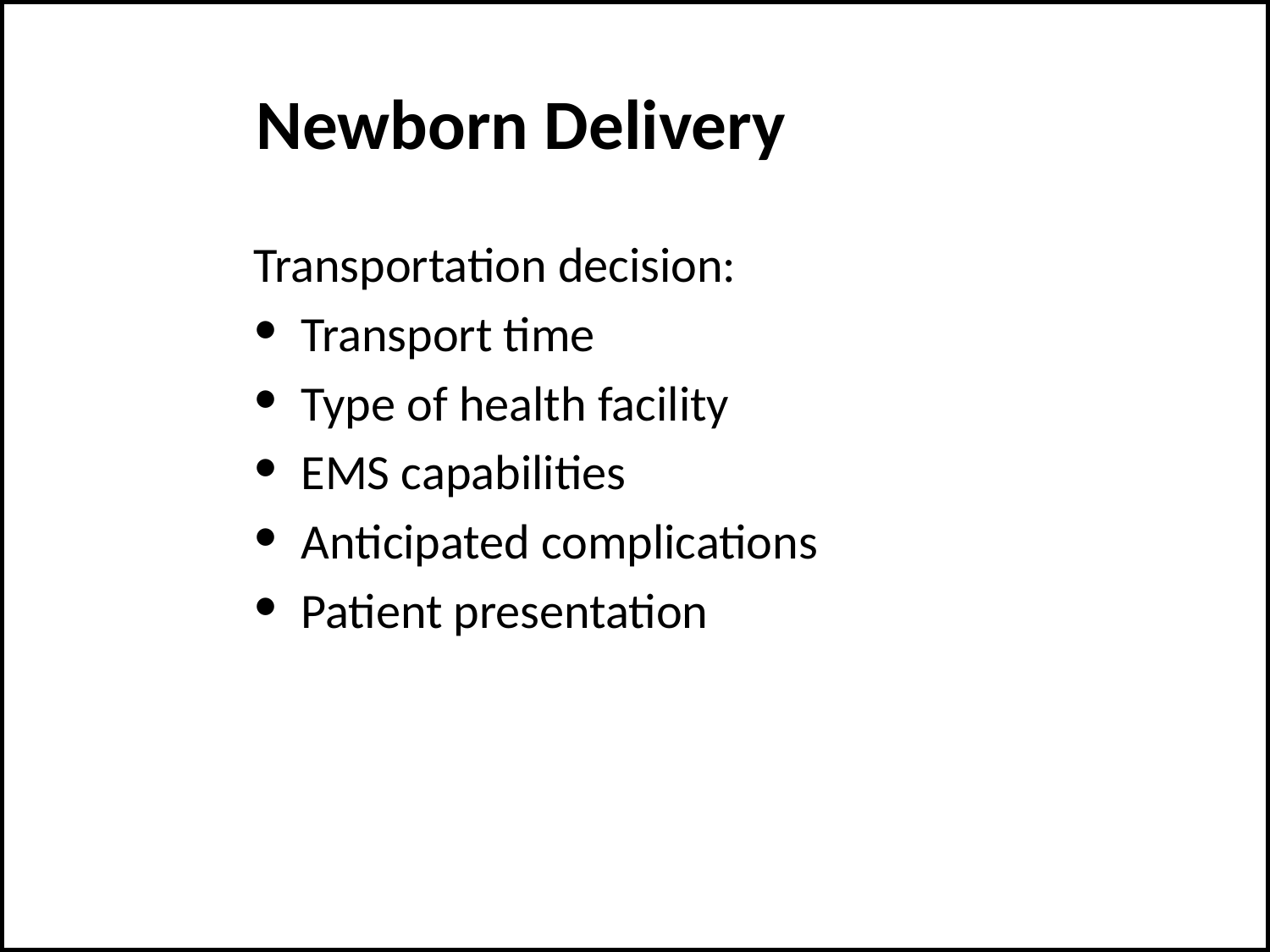

# Newborn Delivery
Transportation decision:
Transport time
Type of health facility
EMS capabilities
Anticipated complications
Patient presentation

## Slide 4
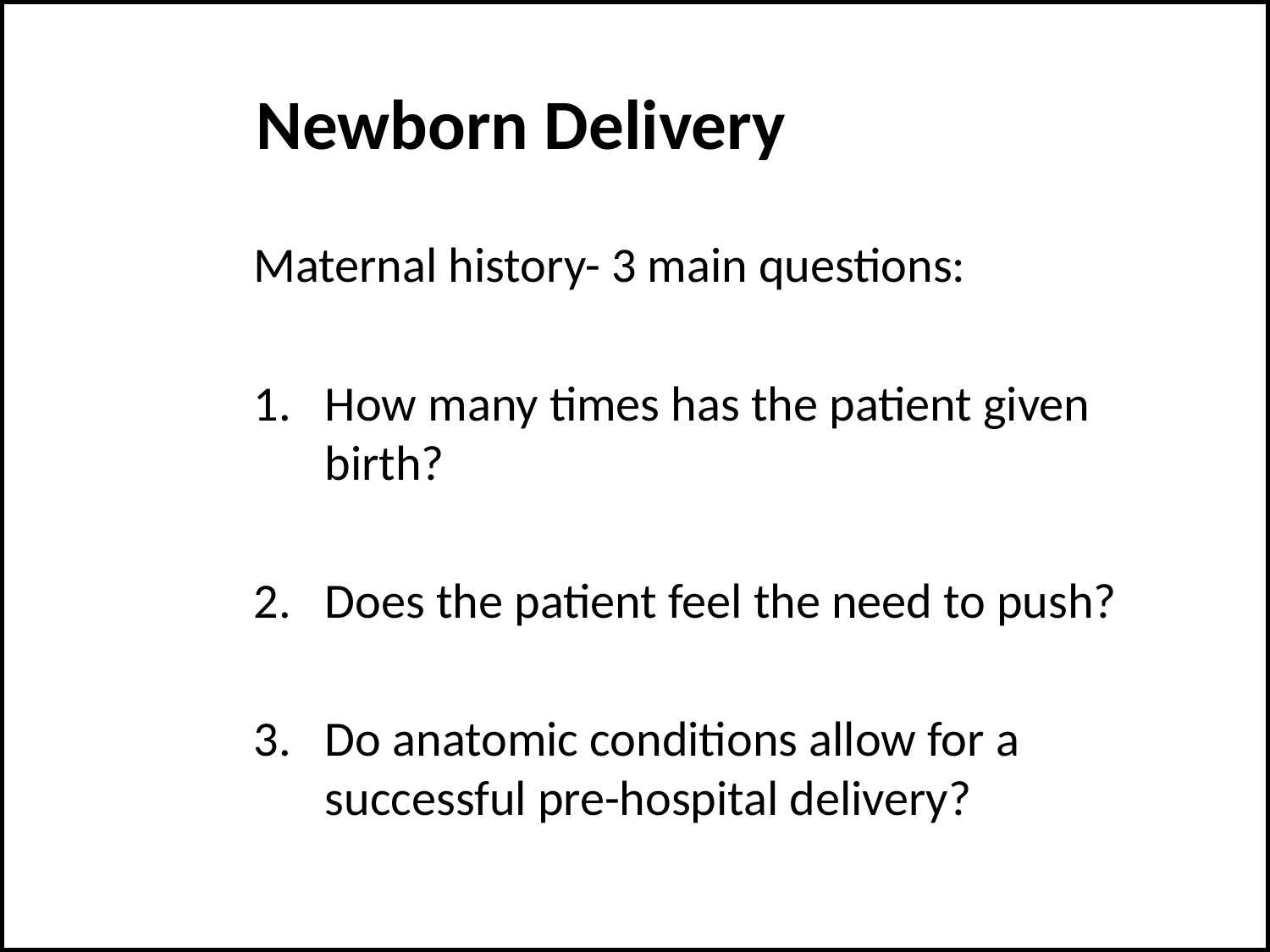

# Newborn Delivery
Maternal history- 3 main questions:
How many times has the patient given birth?
Does the patient feel the need to push?
Do anatomic conditions allow for a successful pre-hospital delivery?

## Slide 5
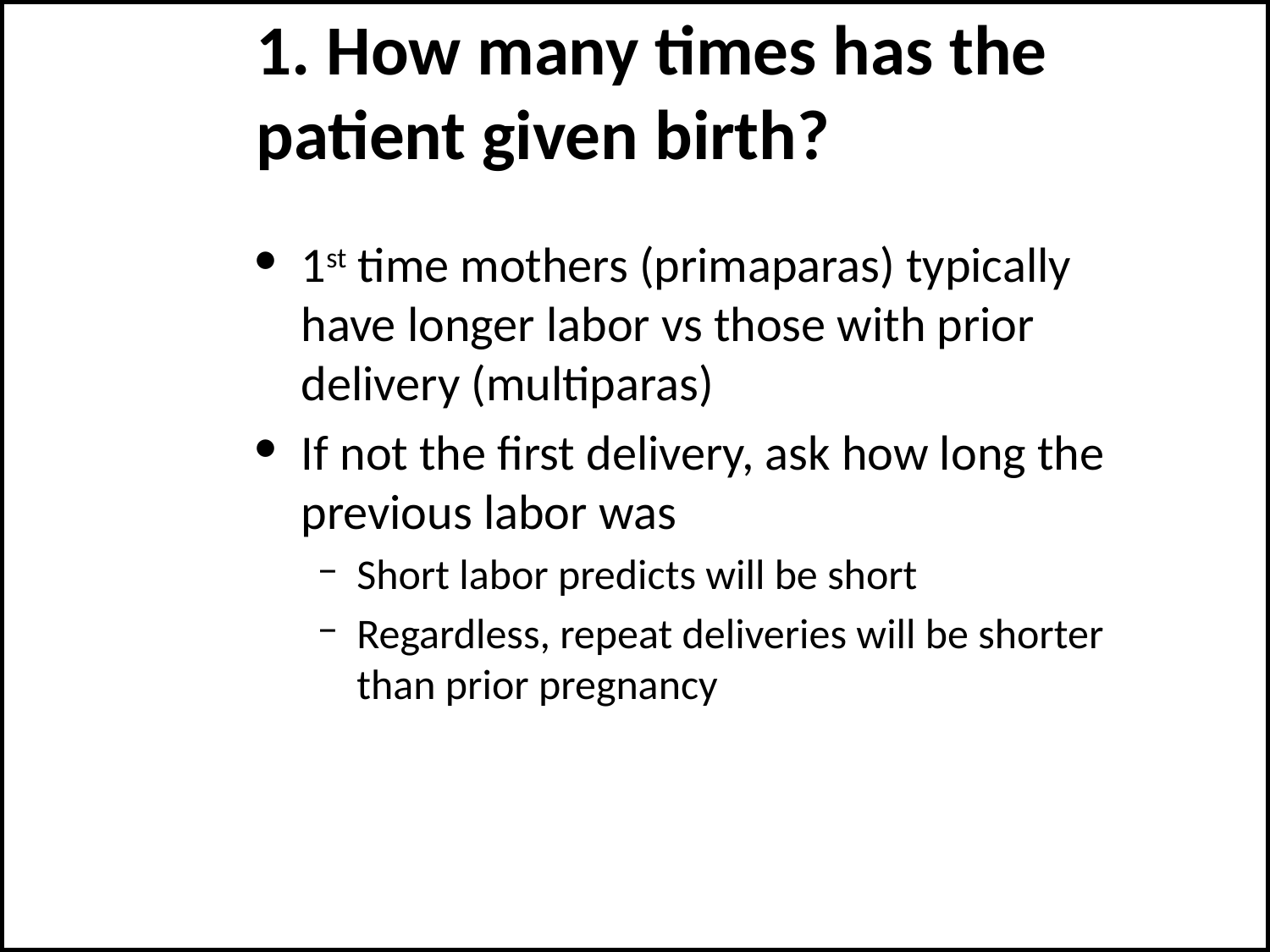

# 1. How many times has the patient given birth?
1st time mothers (primaparas) typically have longer labor vs those with prior delivery (multiparas)
If not the first delivery, ask how long the previous labor was
Short labor predicts will be short
Regardless, repeat deliveries will be shorter than prior pregnancy

## Slide 6
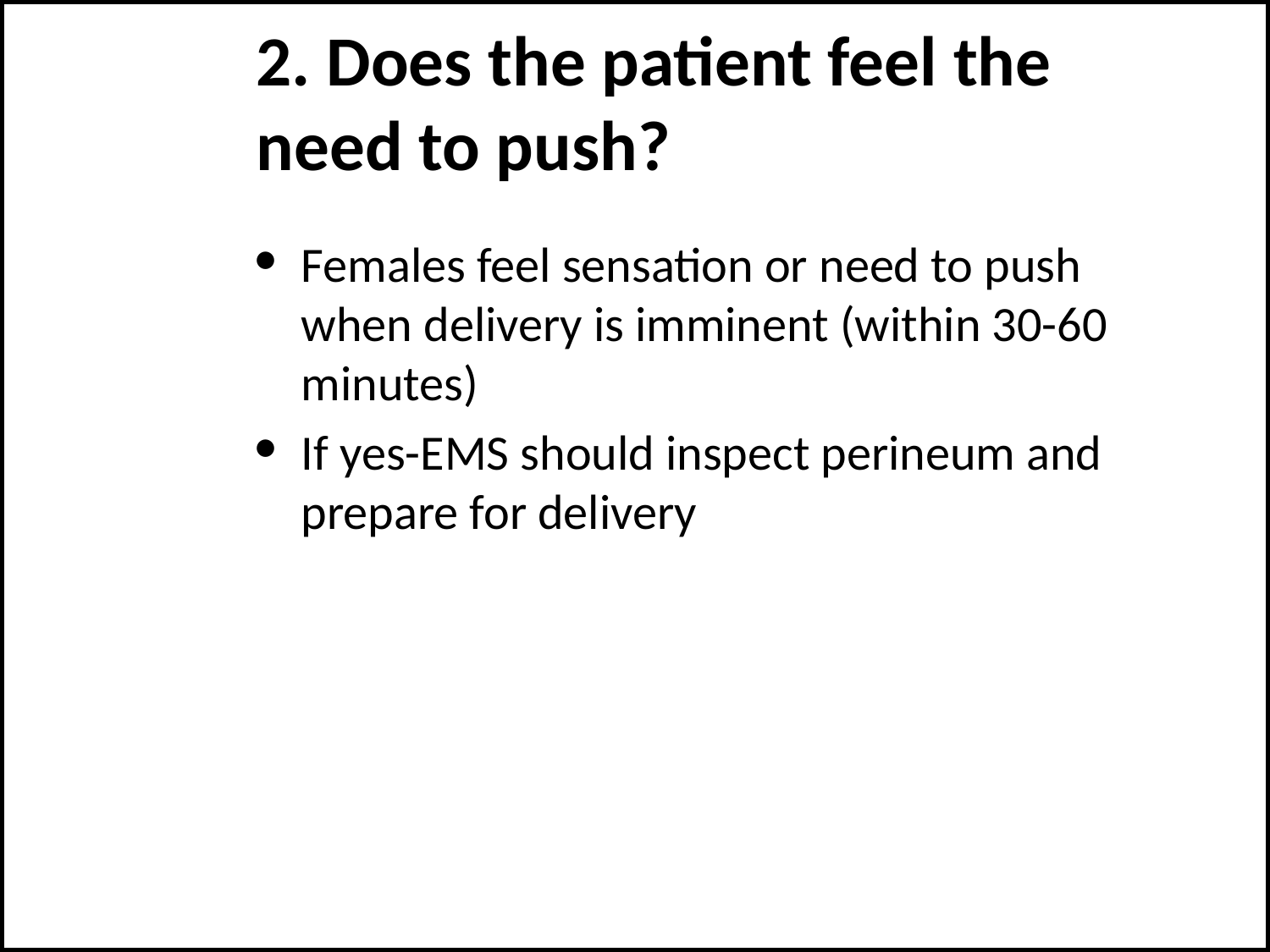

# 2. Does the patient feel the need to push?
Females feel sensation or need to push when delivery is imminent (within 30-60 minutes)
If yes-EMS should inspect perineum and prepare for delivery

## Slide 7
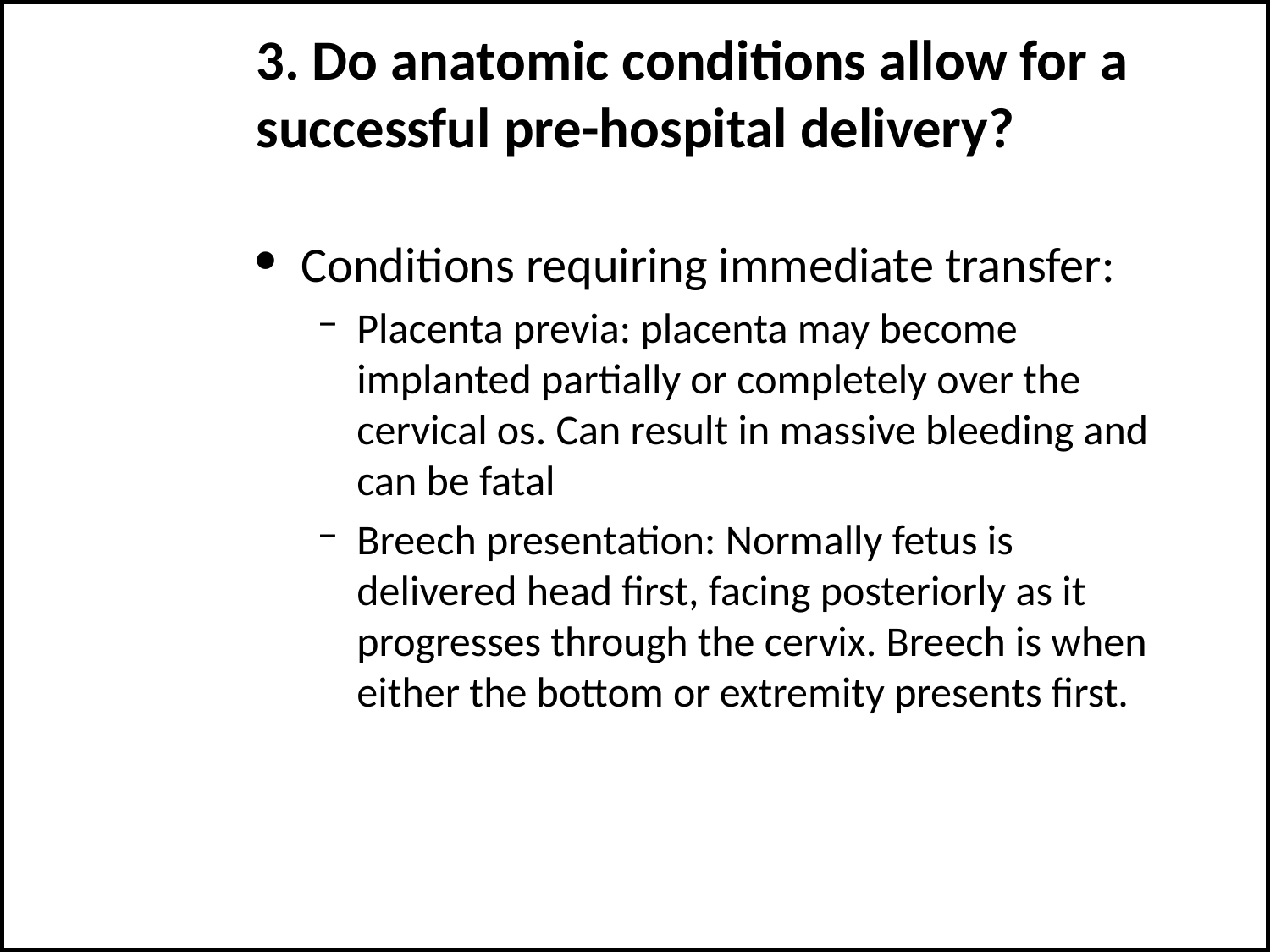

# 3. Do anatomic conditions allow for a successful pre-hospital delivery?
Conditions requiring immediate transfer:
Placenta previa: placenta may become implanted partially or completely over the cervical os. Can result in massive bleeding and can be fatal
Breech presentation: Normally fetus is delivered head first, facing posteriorly as it progresses through the cervix. Breech is when either the bottom or extremity presents first.

## Slide 8
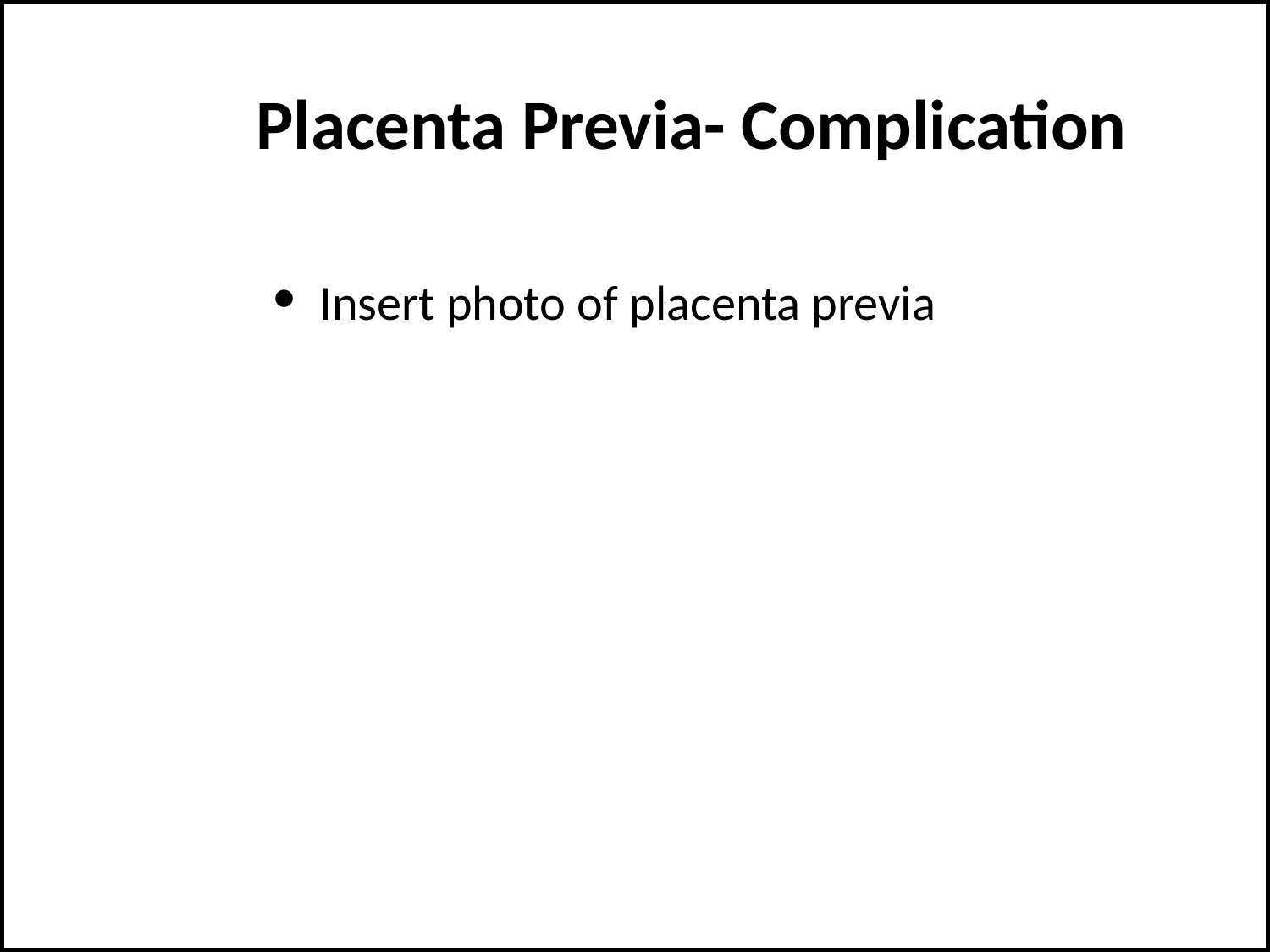

# Placenta Previa- Complication
Insert photo of placenta previa

## Slide 9
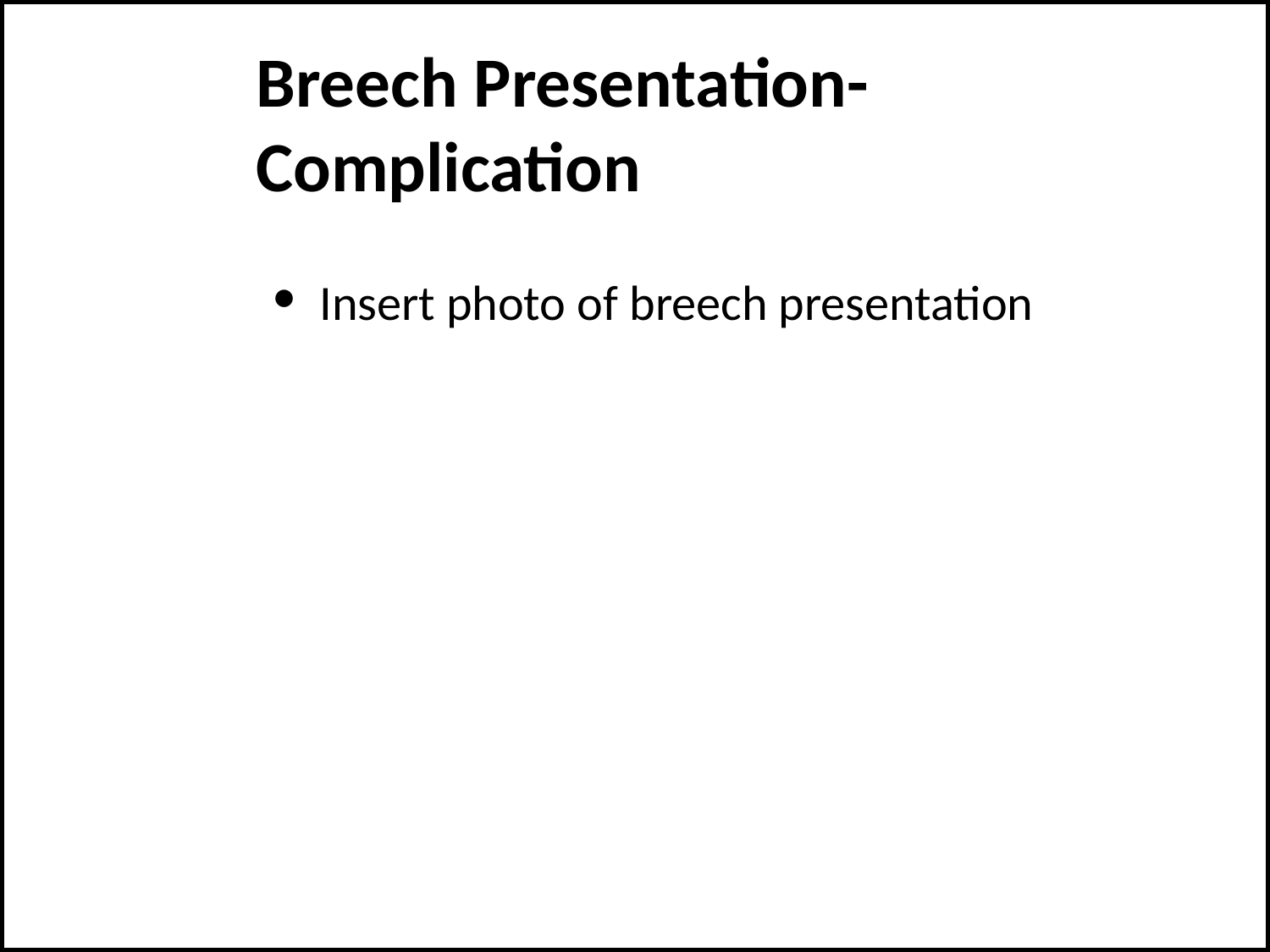

# Breech Presentation- Complication
Insert photo of breech presentation

## Slide 10
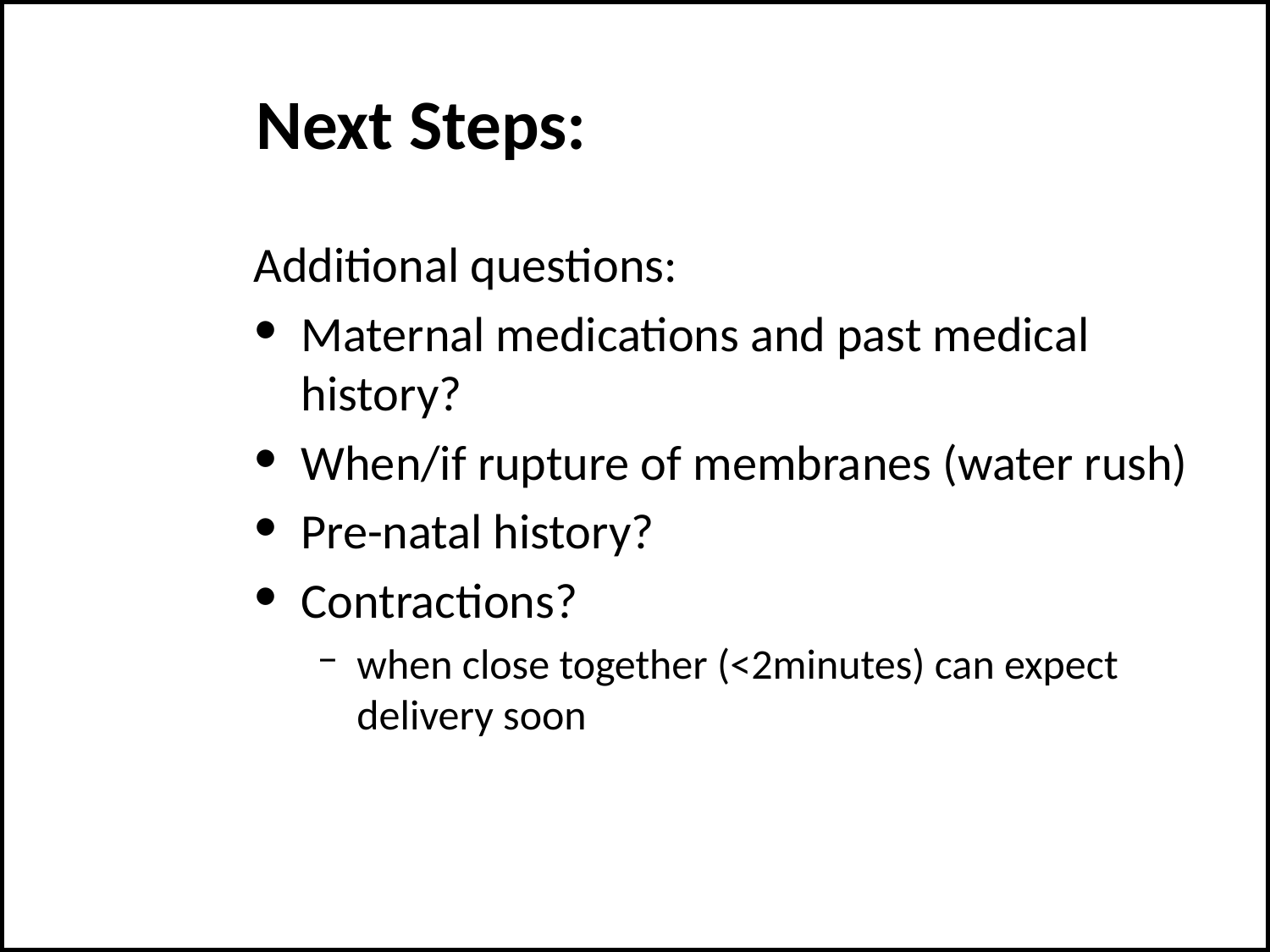

# Next Steps:
Additional questions:
Maternal medications and past medical history?
When/if rupture of membranes (water rush)
Pre-natal history?
Contractions?
when close together (<2minutes) can expect delivery soon

## Slide 11
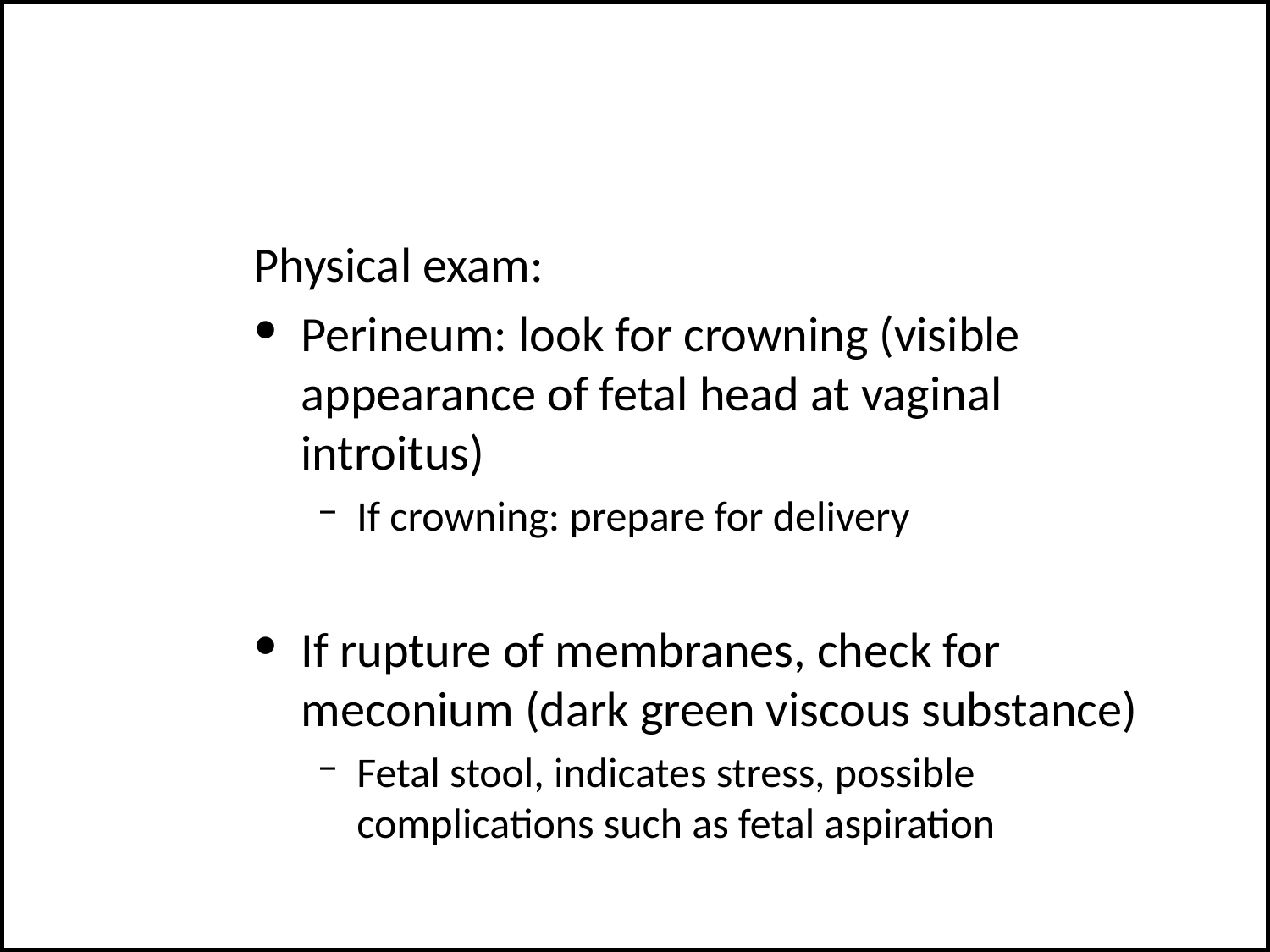

Physical exam:
Perineum: look for crowning (visible appearance of fetal head at vaginal introitus)
If crowning: prepare for delivery
If rupture of membranes, check for meconium (dark green viscous substance)
Fetal stool, indicates stress, possible complications such as fetal aspiration

## Slide 12
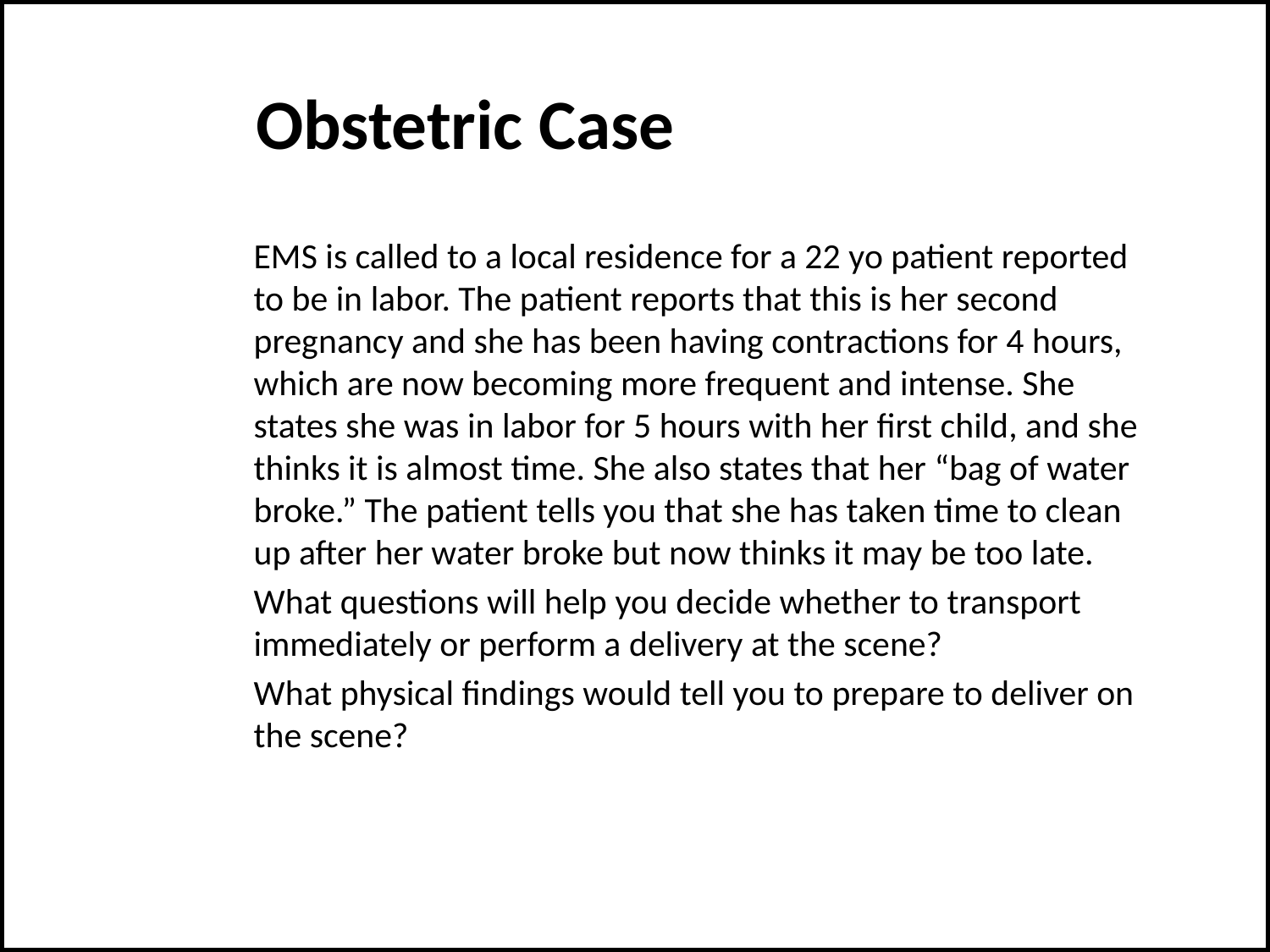

# Obstetric Case
EMS is called to a local residence for a 22 yo patient reported to be in labor. The patient reports that this is her second pregnancy and she has been having contractions for 4 hours, which are now becoming more frequent and intense. She states she was in labor for 5 hours with her first child, and she thinks it is almost time. She also states that her “bag of water broke.” The patient tells you that she has taken time to clean up after her water broke but now thinks it may be too late.
What questions will help you decide whether to transport immediately or perform a delivery at the scene?
What physical findings would tell you to prepare to deliver on the scene?

## Slide 13
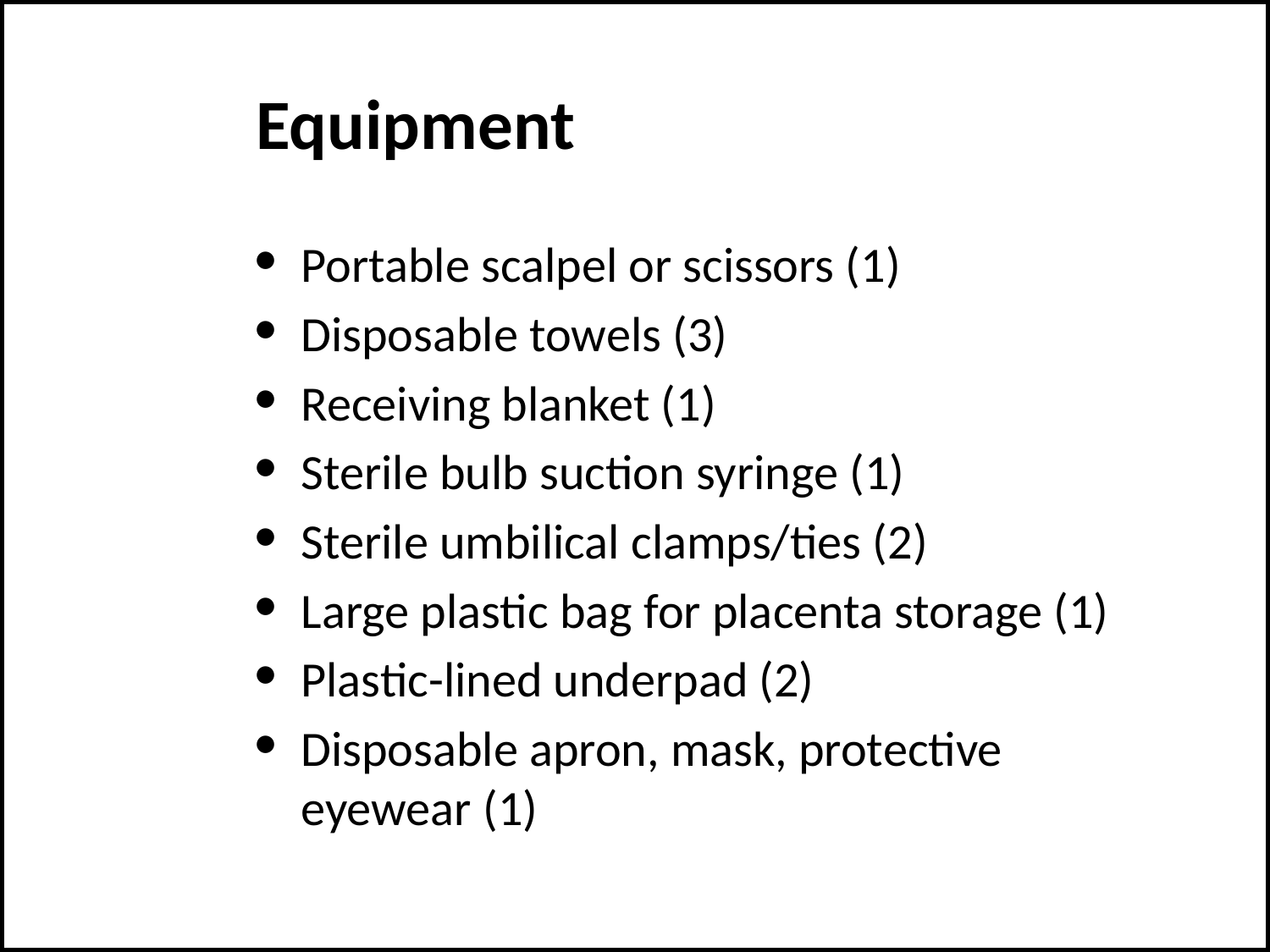

# Equipment
Portable scalpel or scissors (1)
Disposable towels (3)
Receiving blanket (1)
Sterile bulb suction syringe (1)
Sterile umbilical clamps/ties (2)
Large plastic bag for placenta storage (1)
Plastic-lined underpad (2)
Disposable apron, mask, protective eyewear (1)

## Slide 14
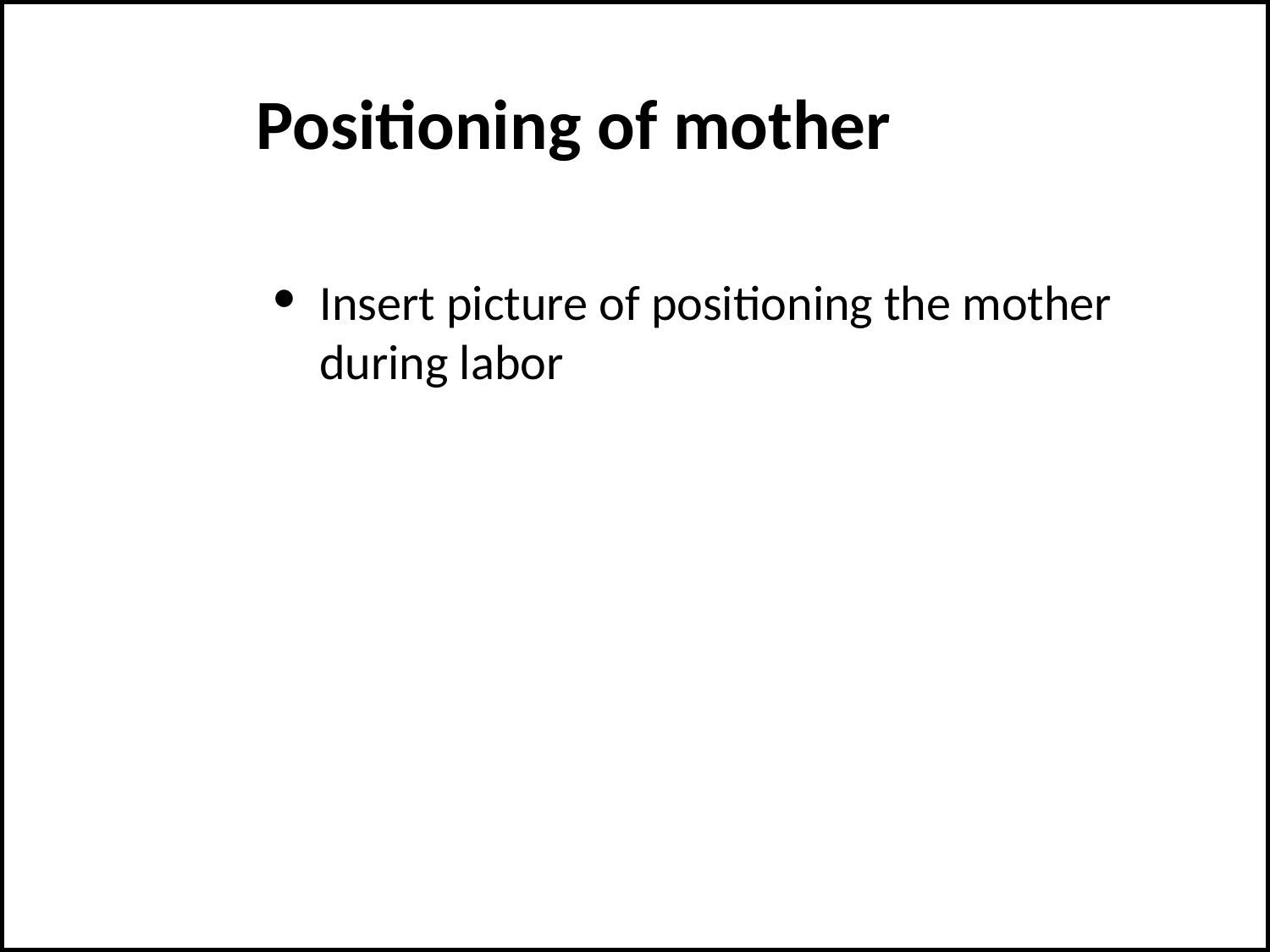

# Positioning of mother
Insert picture of positioning the mother during labor

## Slide 15
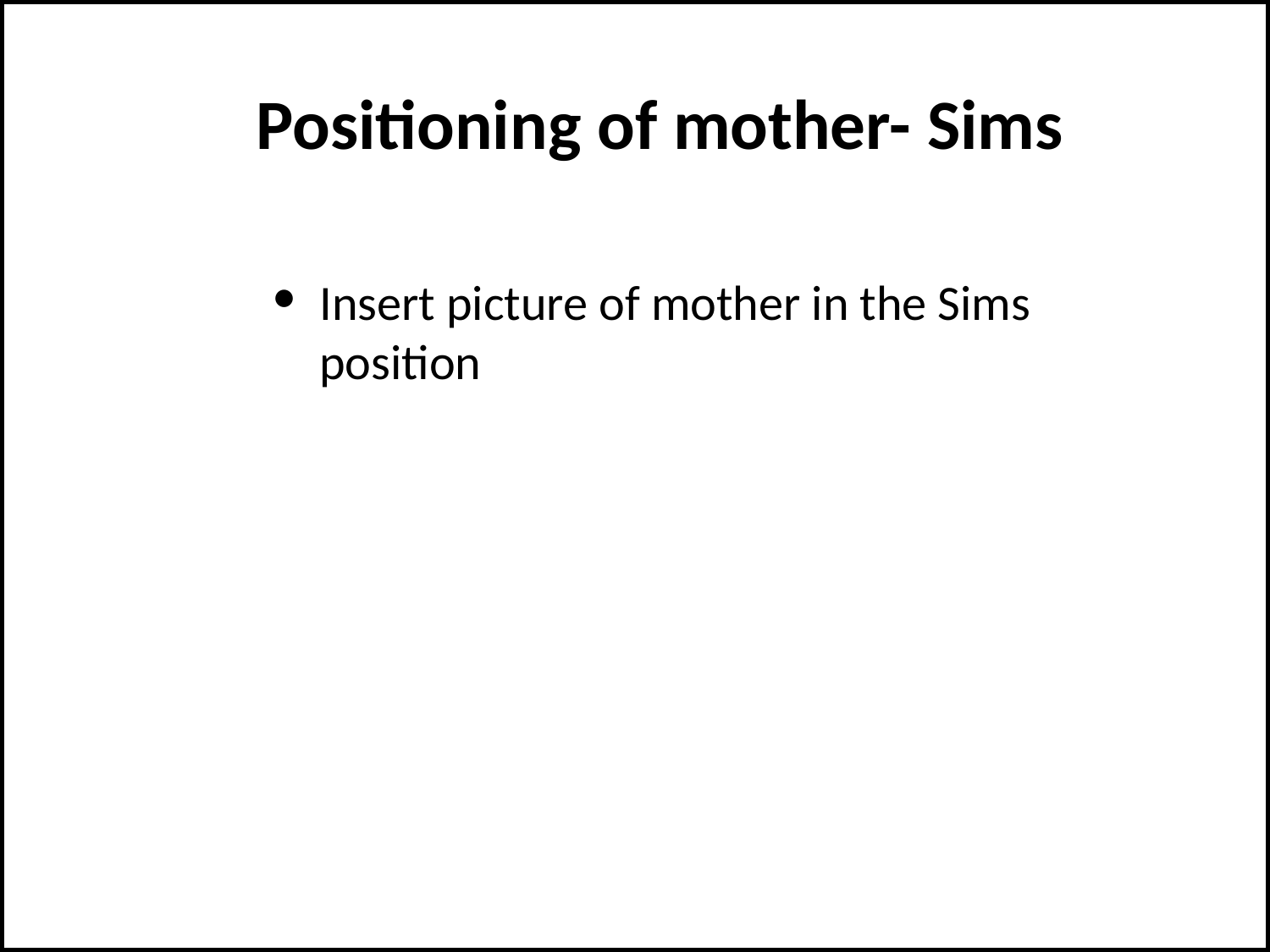

# Positioning of mother- Sims
Insert picture of mother in the Sims position

## Slide 16
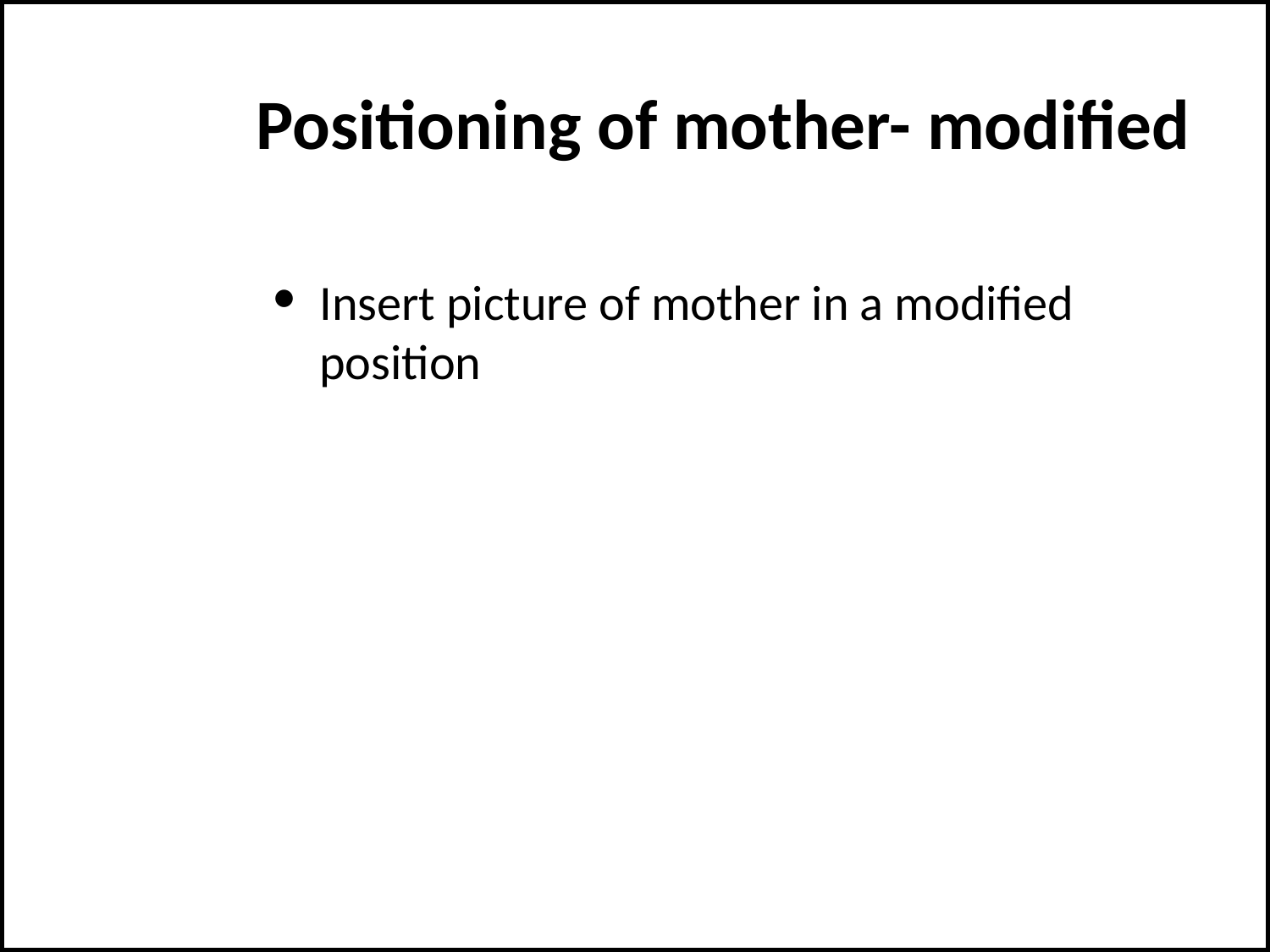

# Positioning of mother- modified
Insert picture of mother in a modified position

## Slide 17
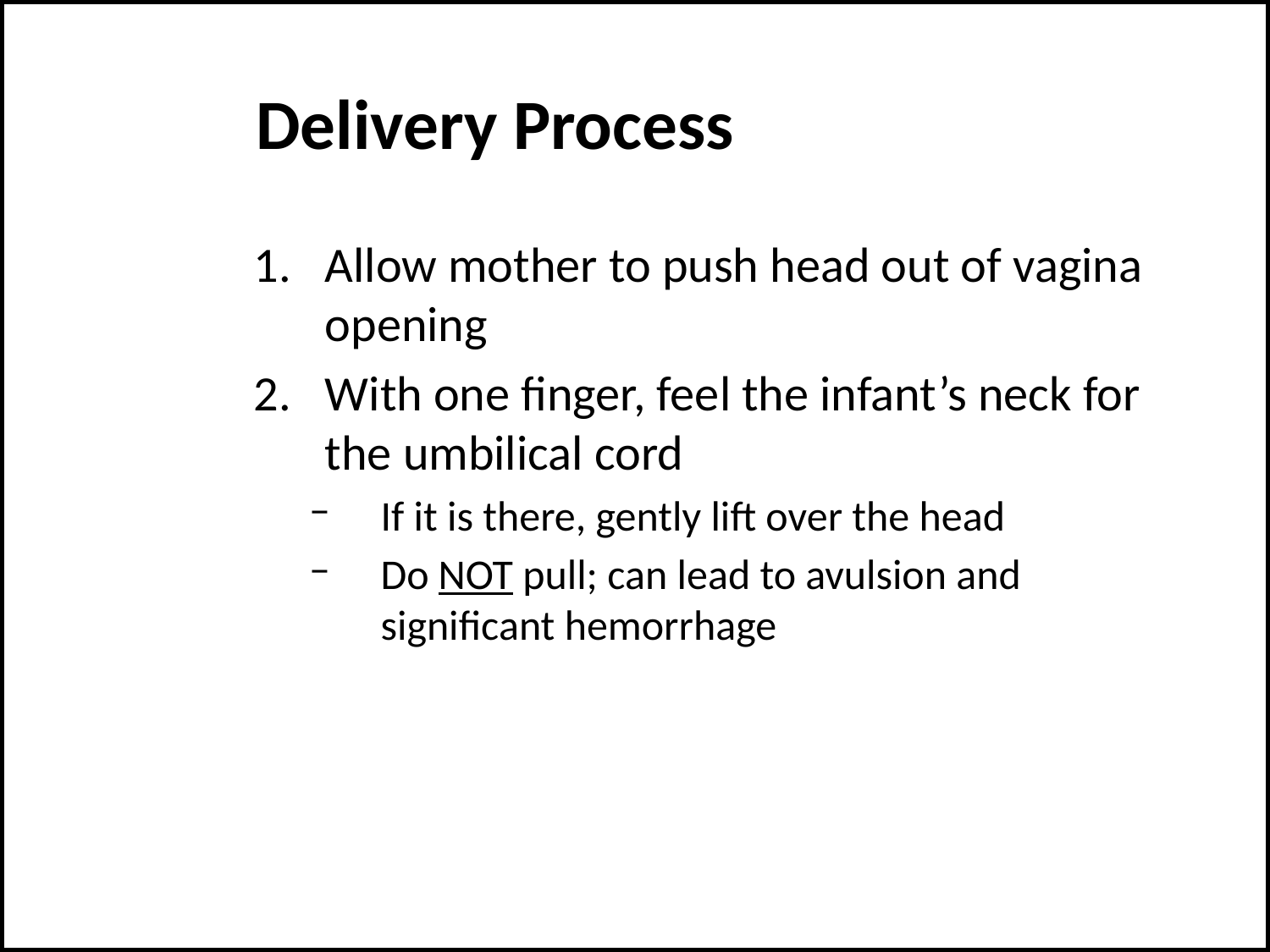

# Delivery Process
Allow mother to push head out of vagina opening
With one finger, feel the infant’s neck for the umbilical cord
If it is there, gently lift over the head
Do NOT pull; can lead to avulsion and significant hemorrhage

## Slide 18
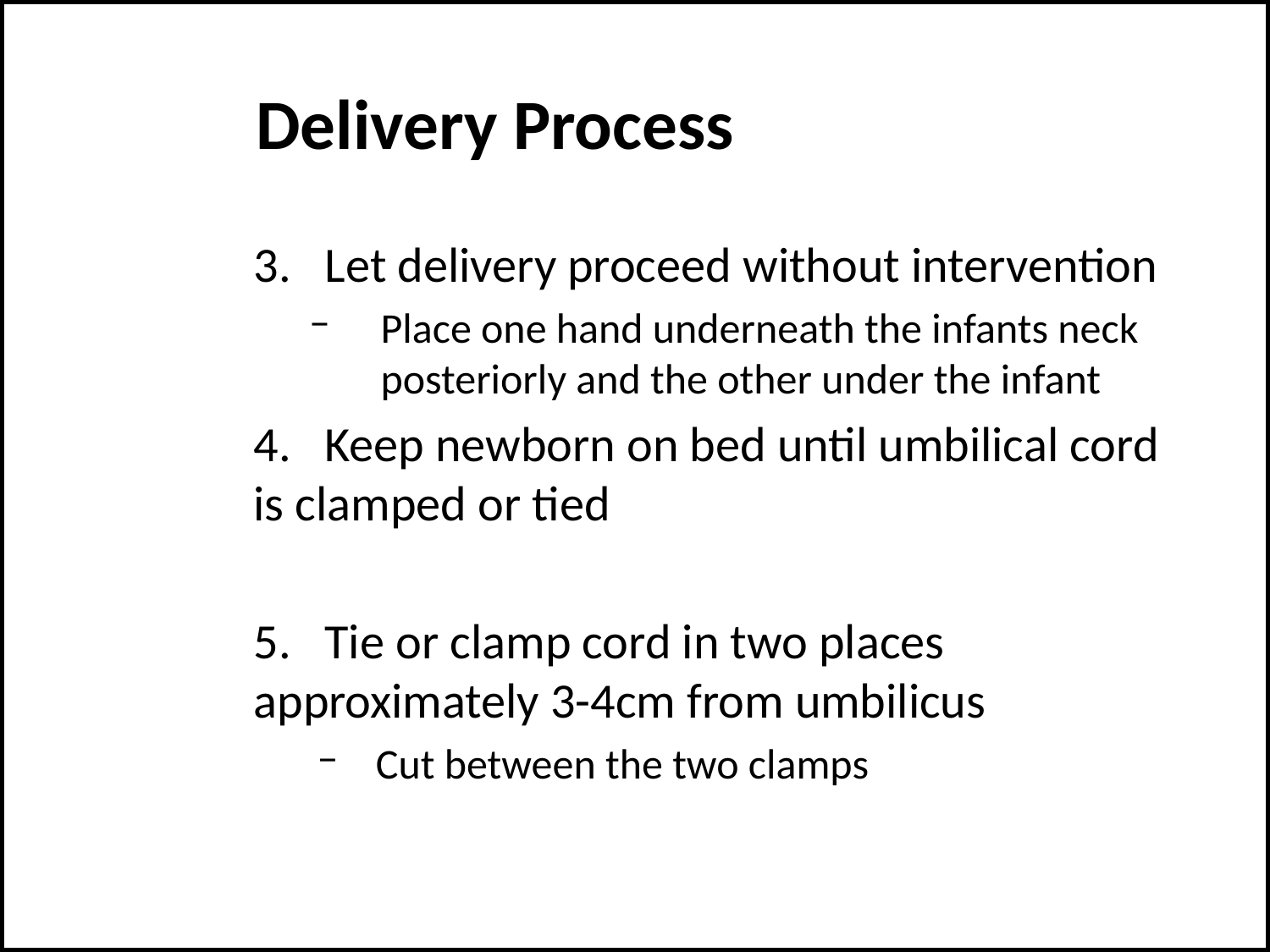

# Delivery Process
Let delivery proceed without intervention
Place one hand underneath the infants neck posteriorly and the other under the infant
4. Keep newborn on bed until umbilical cord is clamped or tied
5. Tie or clamp cord in two places approximately 3-4cm from umbilicus
 Cut between the two clamps

## Slide 19
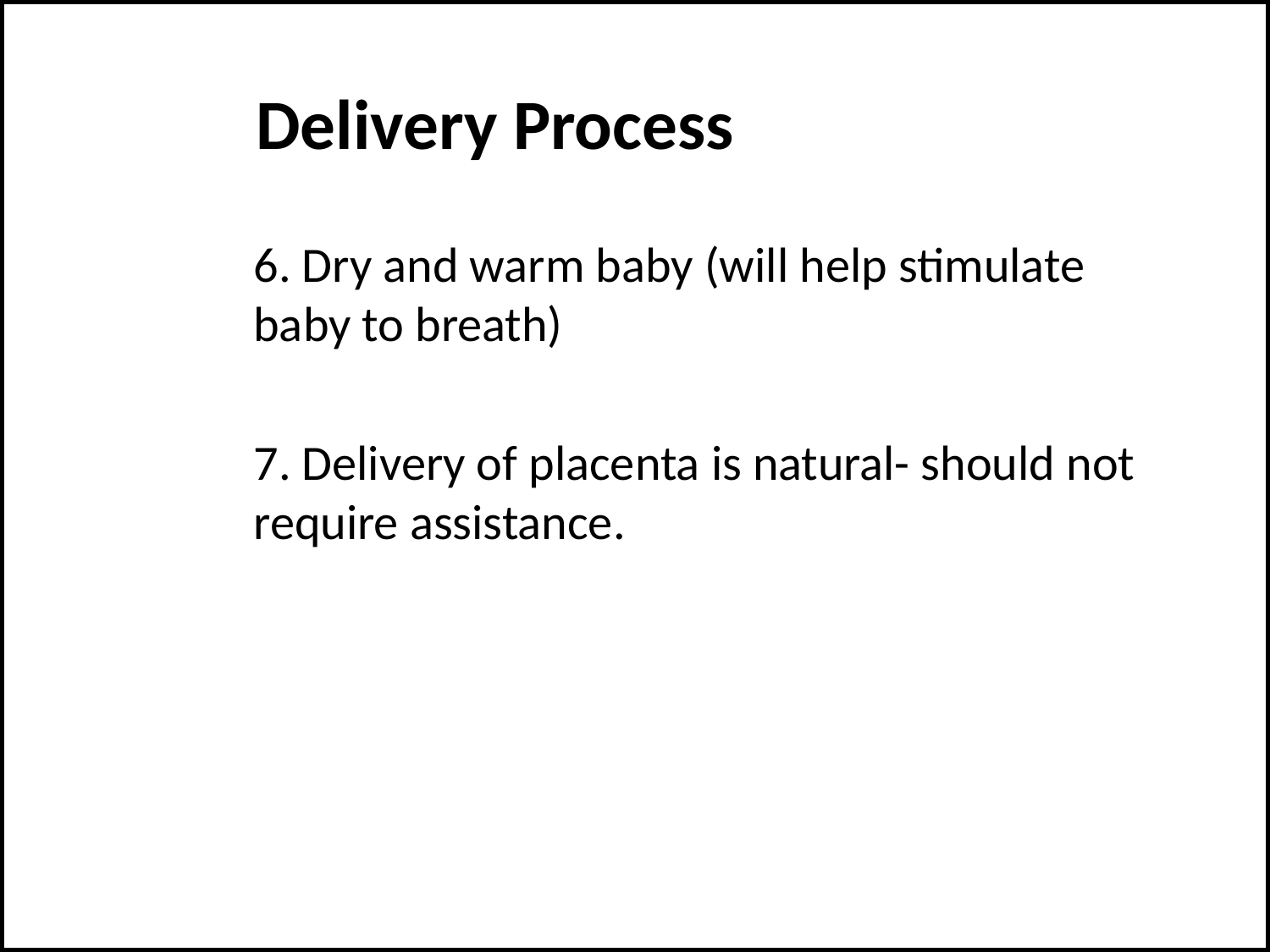

# Delivery Process
6. Dry and warm baby (will help stimulate baby to breath)
7. Delivery of placenta is natural- should not require assistance.

## Slide 20
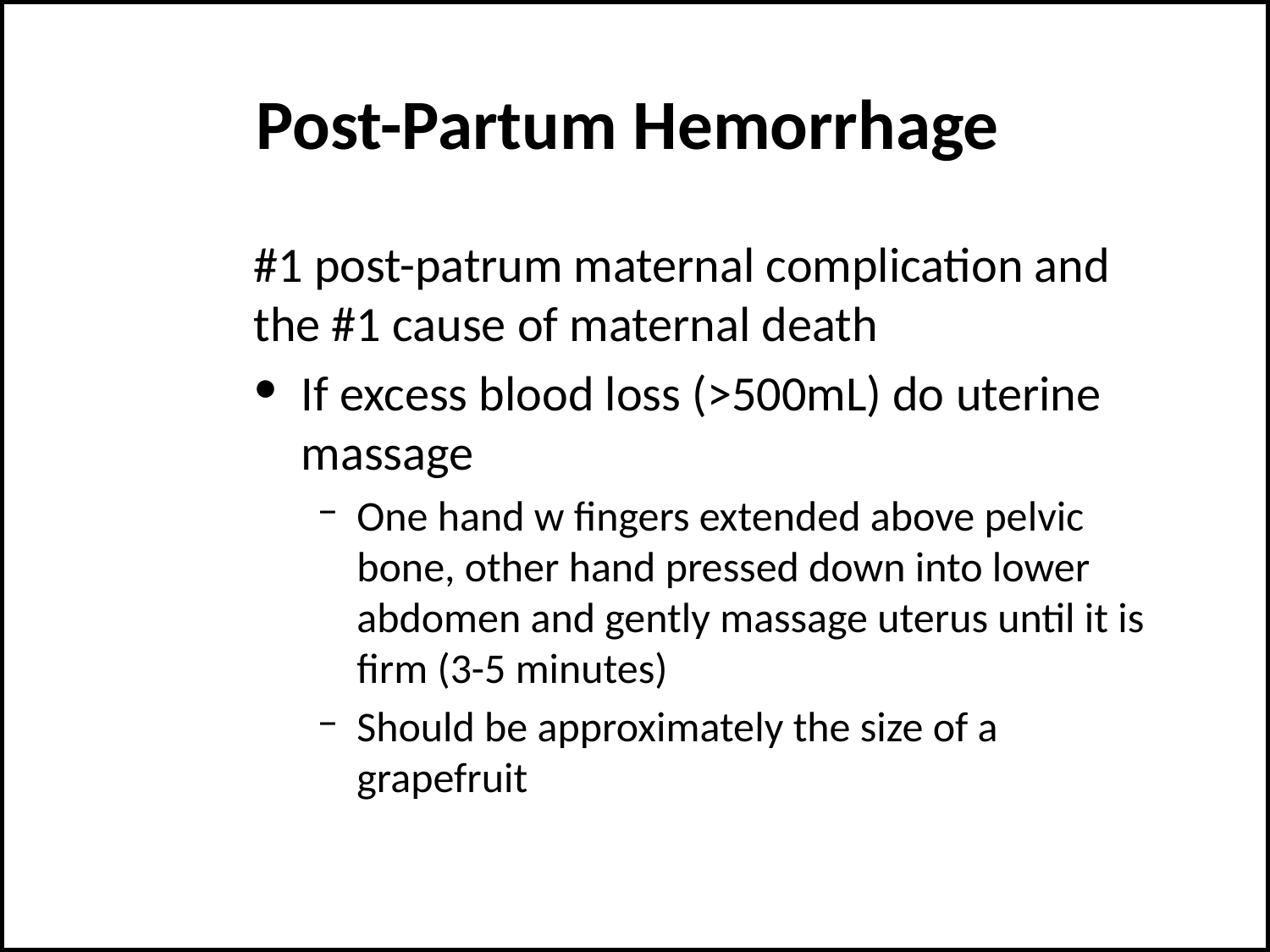

# Post-Partum Hemorrhage
#1 post-patrum maternal complication and the #1 cause of maternal death
If excess blood loss (>500mL) do uterine massage
One hand w fingers extended above pelvic bone, other hand pressed down into lower abdomen and gently massage uterus until it is firm (3-5 minutes)
Should be approximately the size of a grapefruit

## Slide 21
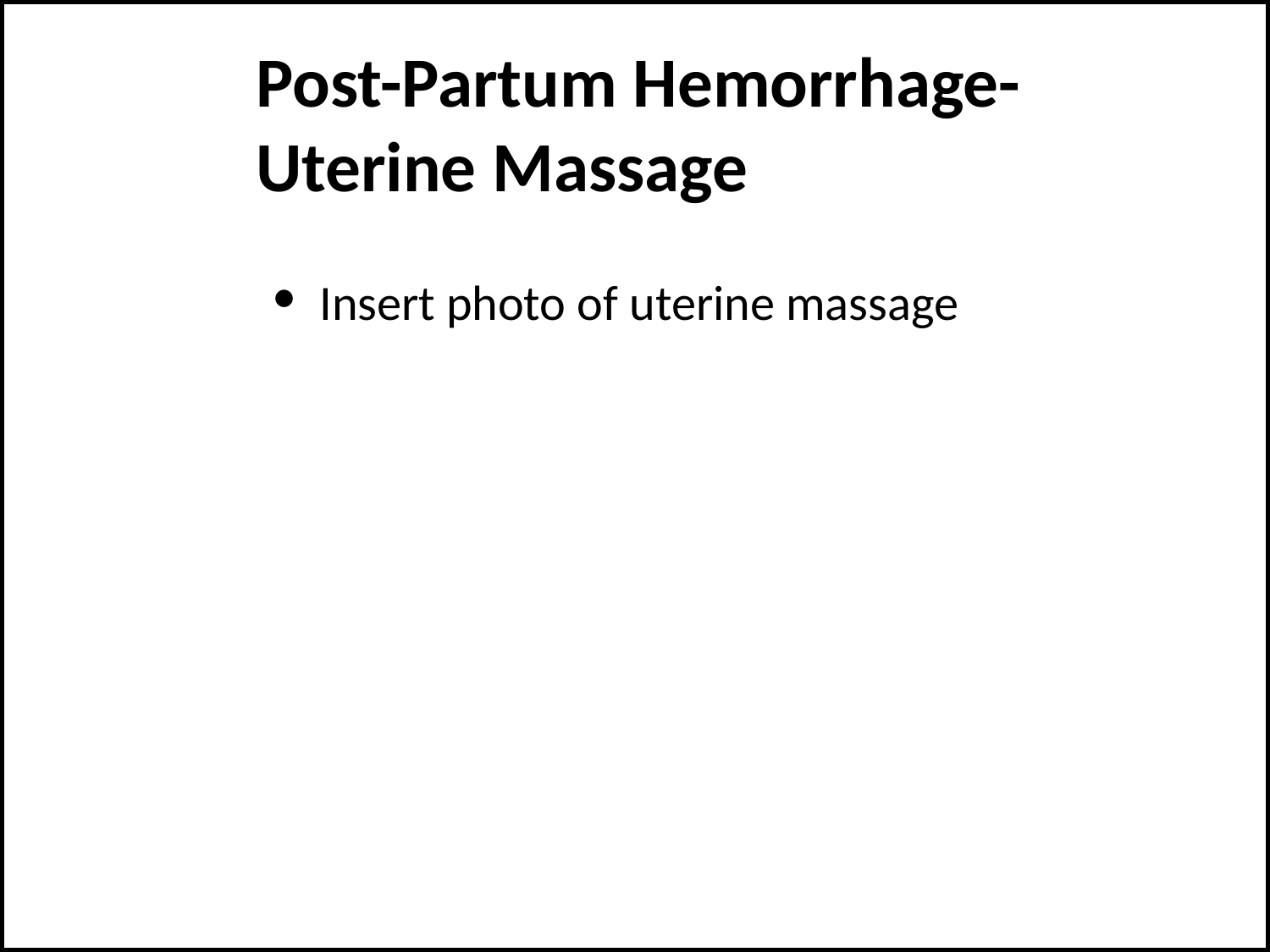

# Post-Partum Hemorrhage- Uterine Massage
Insert photo of uterine massage

## Slide 22
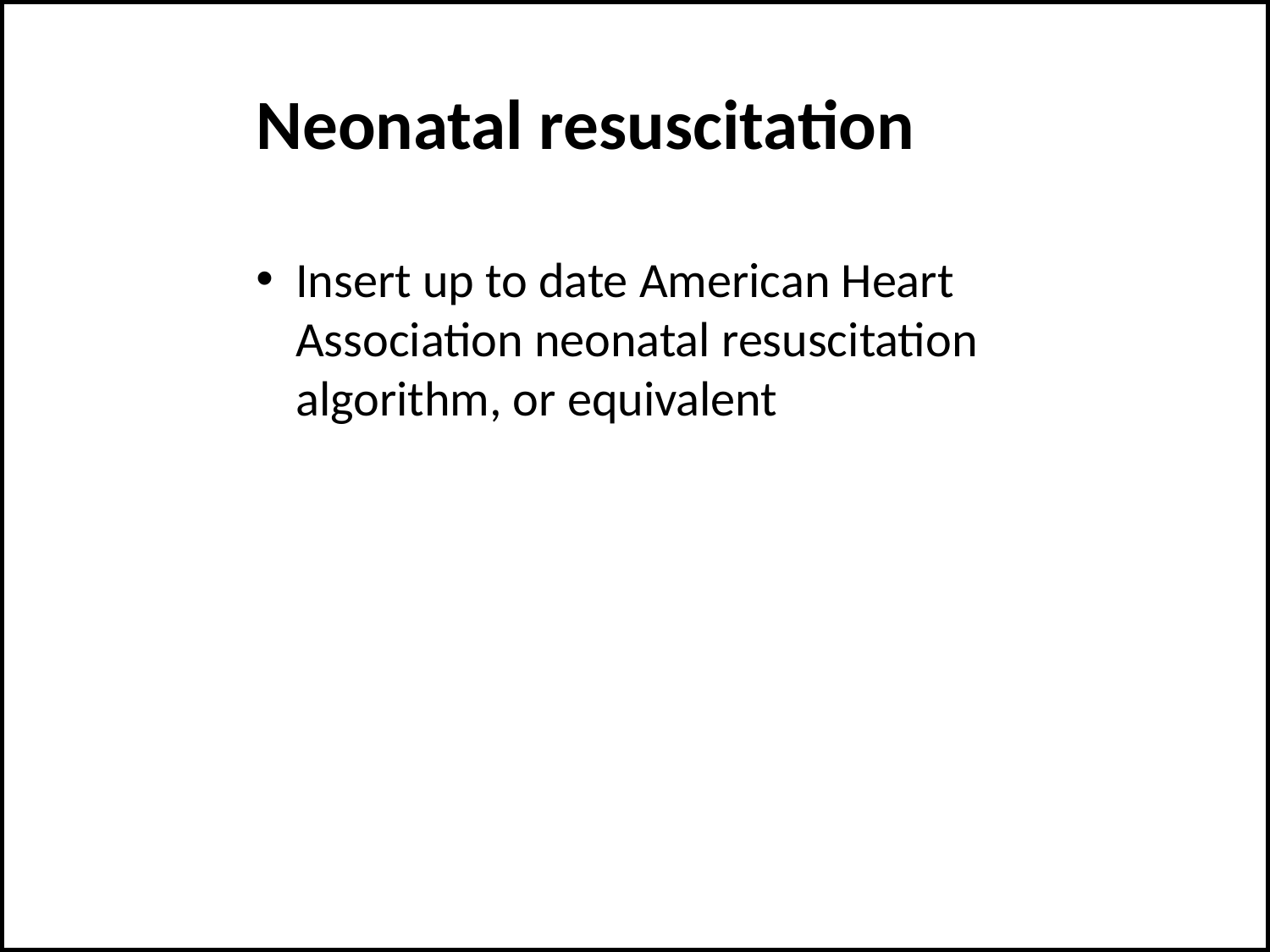

# Neonatal resuscitation
Insert up to date American Heart Association neonatal resuscitation algorithm, or equivalent
