# Supplementary material for: Pediatric Simulation-Based Prehospital Training Course in Botswana
Source: J Educ Teach Emerg Med. 2021 Jul 15;6(3):C64–C189. doi: 10.21980/J8306S (PMC10332686; doi:10.21980/J8306S)
Supplement: Supplementary file 4 — Please see associated PowerPoint file [file jetem-6-3-c64-appendixT.pptx]

## Slide 1
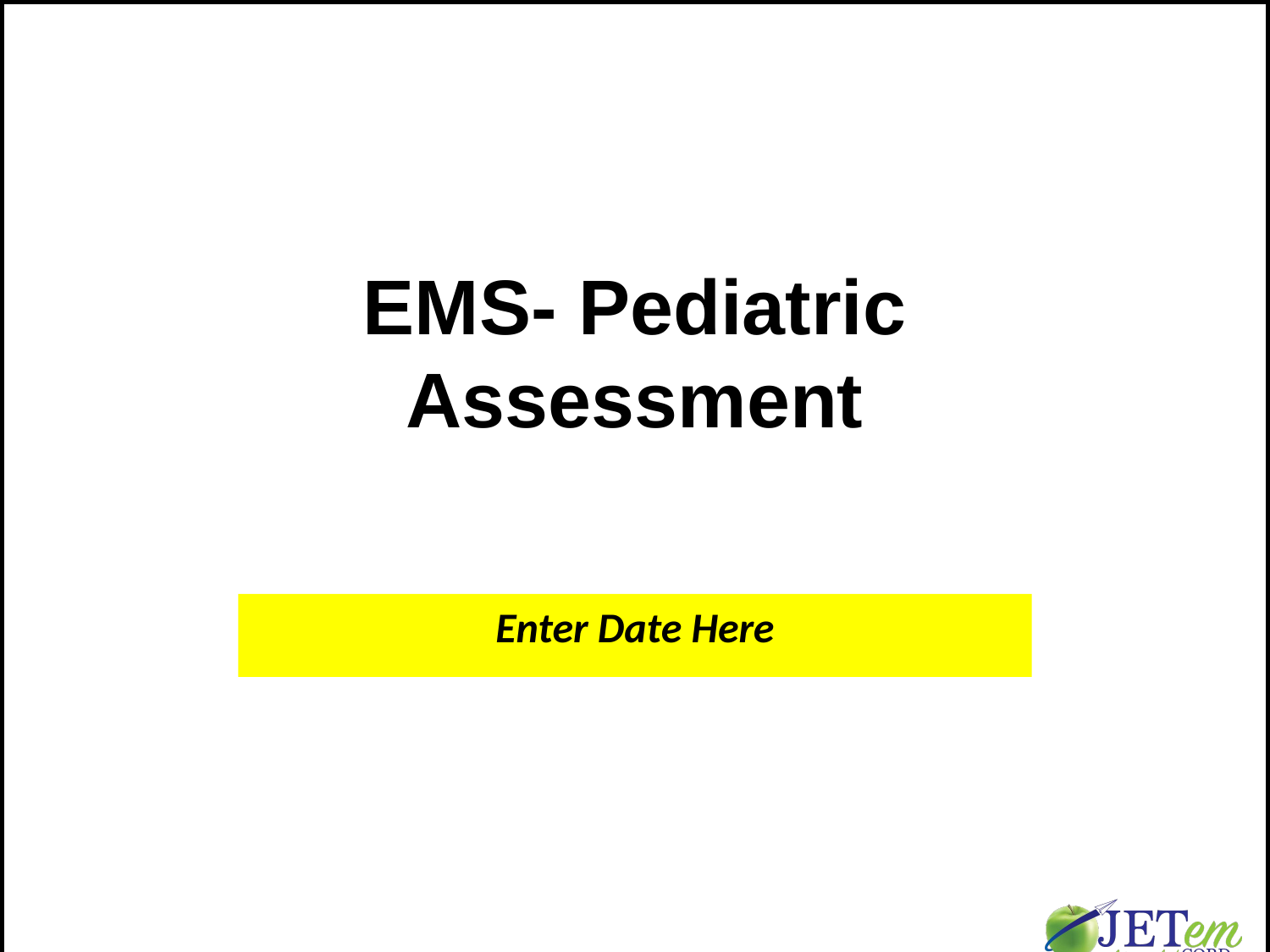

# EMS- Pediatric Assessment
Enter Date Here

## Slide 2
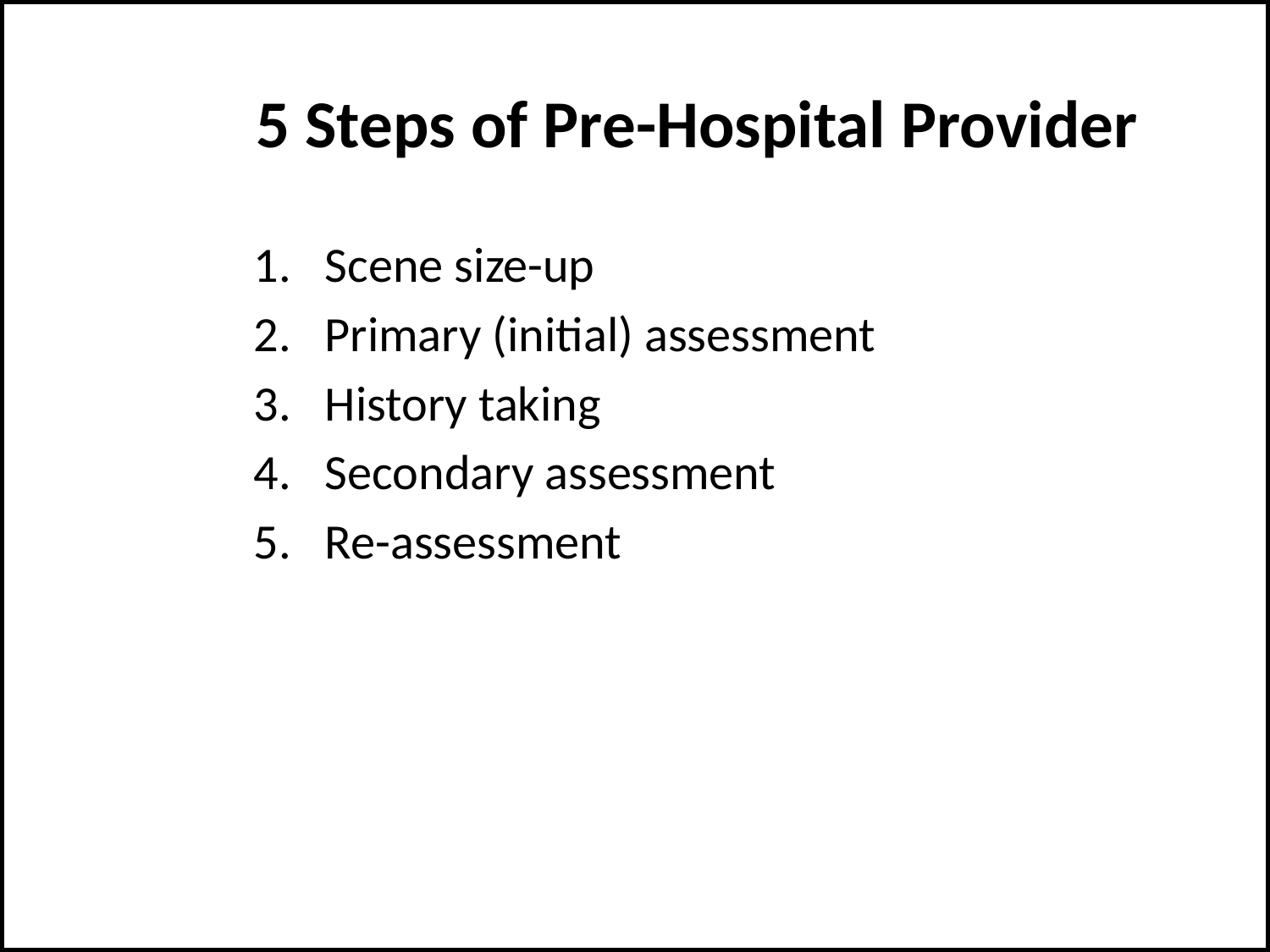

# 5 Steps of Pre-Hospital Provider
Scene size-up
Primary (initial) assessment
History taking
Secondary assessment
Re-assessment

## Slide 3
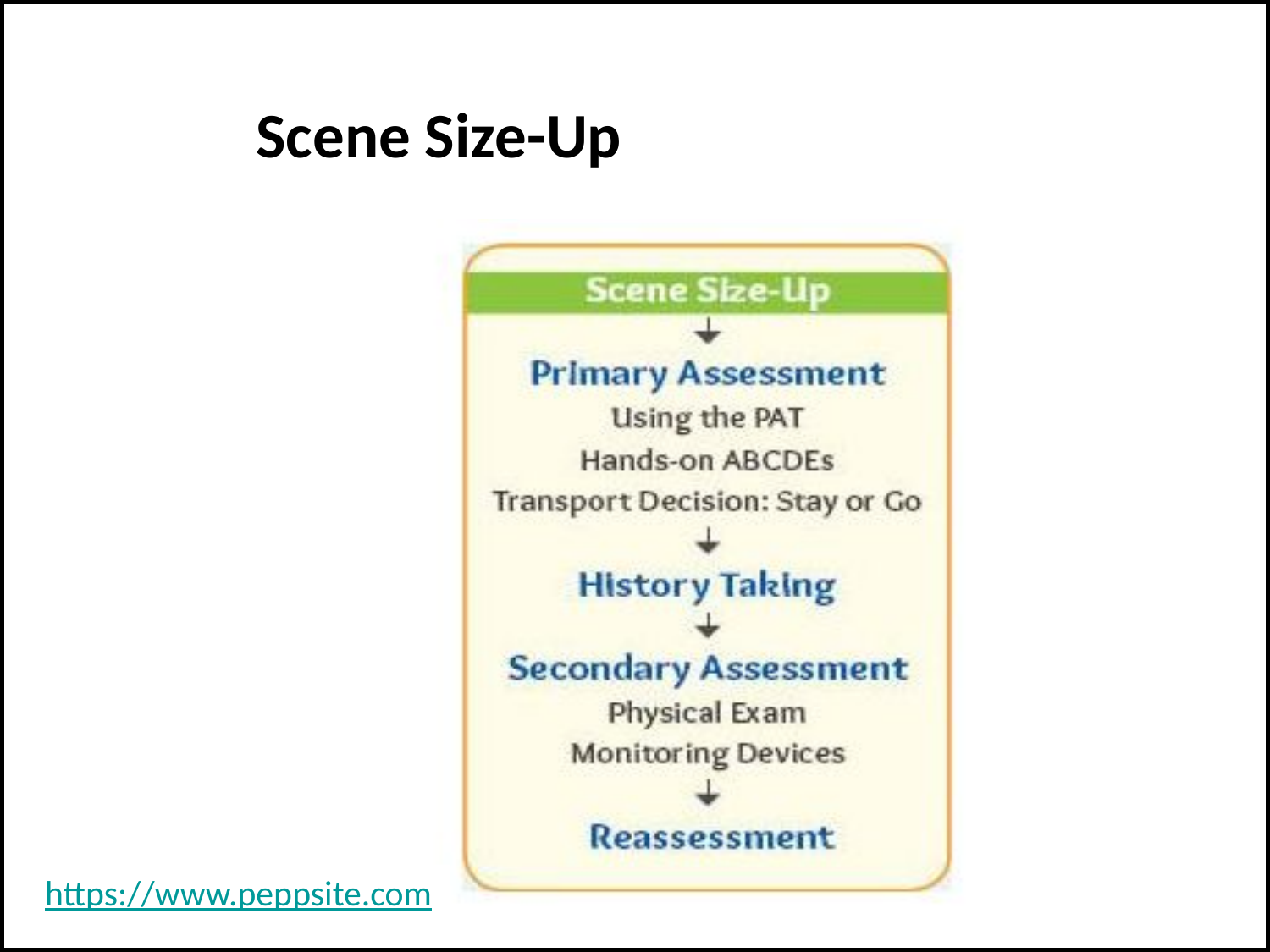

# Scene Size-Up
https://www.peppsite.com

## Slide 4
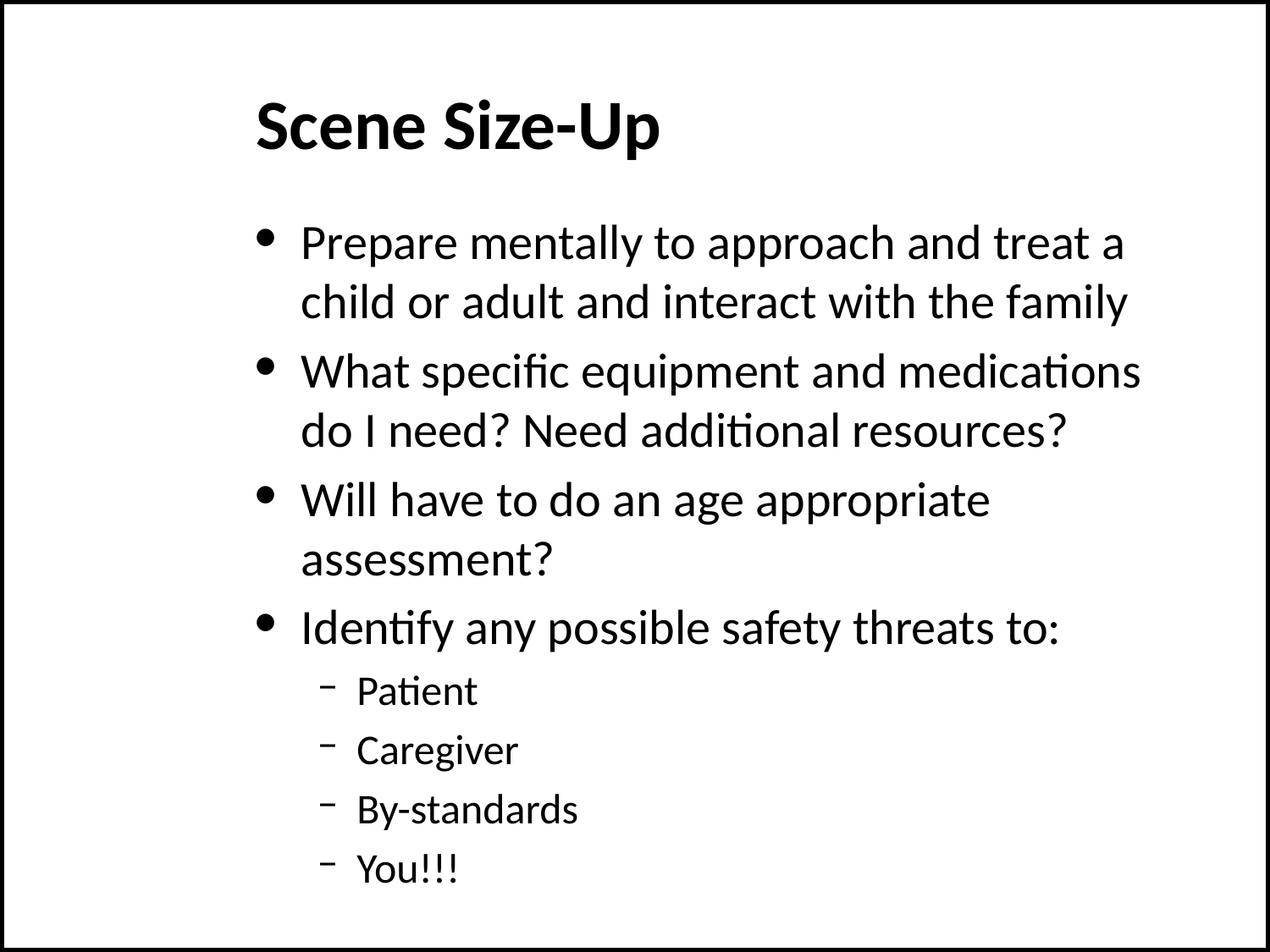

# Scene Size-Up
Prepare mentally to approach and treat a child or adult and interact with the family
What specific equipment and medications do I need? Need additional resources?
Will have to do an age appropriate assessment?
Identify any possible safety threats to:
Patient
Caregiver
By-standards
You!!!

## Slide 5
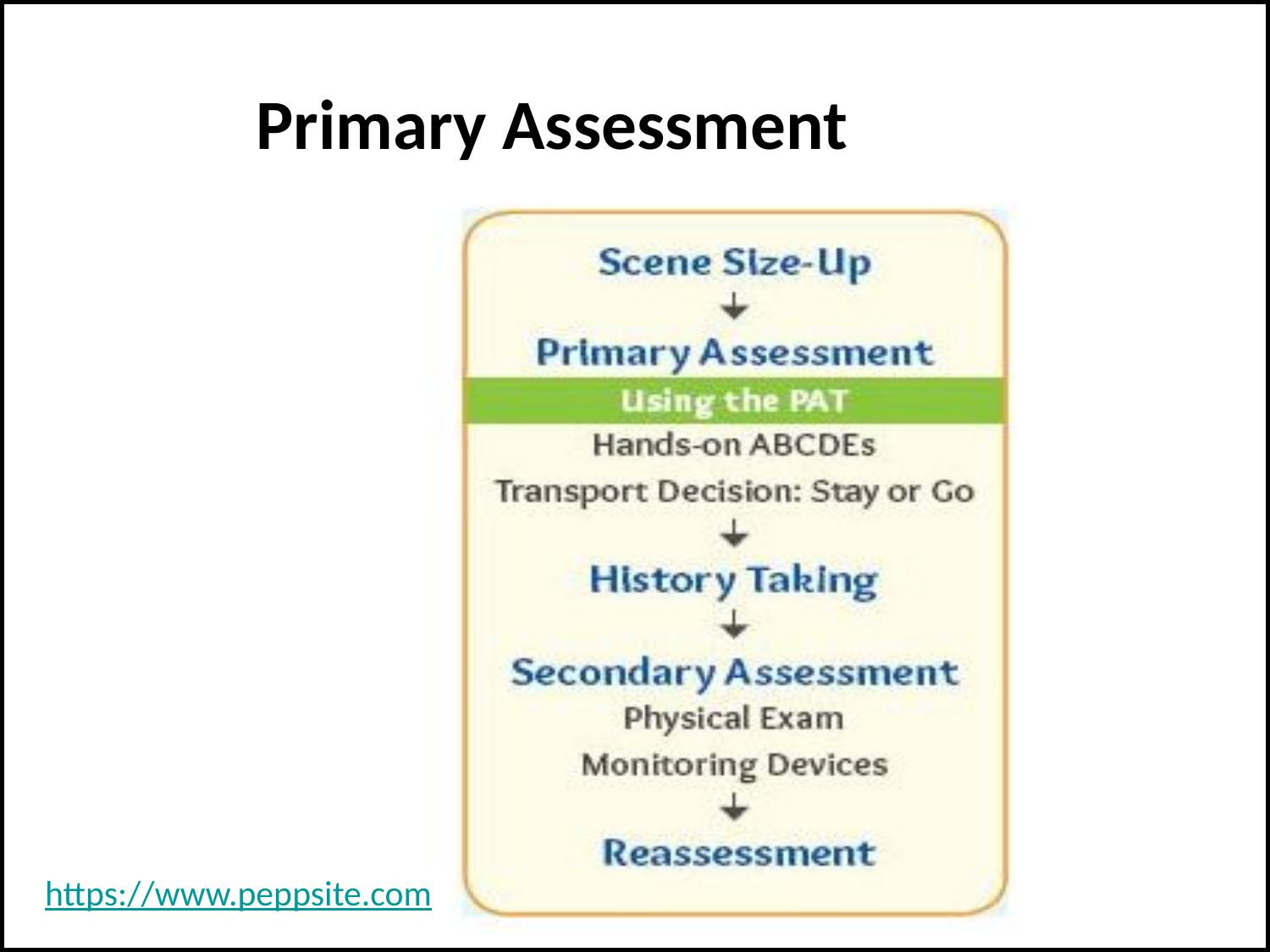

# Primary Assessment
https://www.peppsite.com

## Slide 6
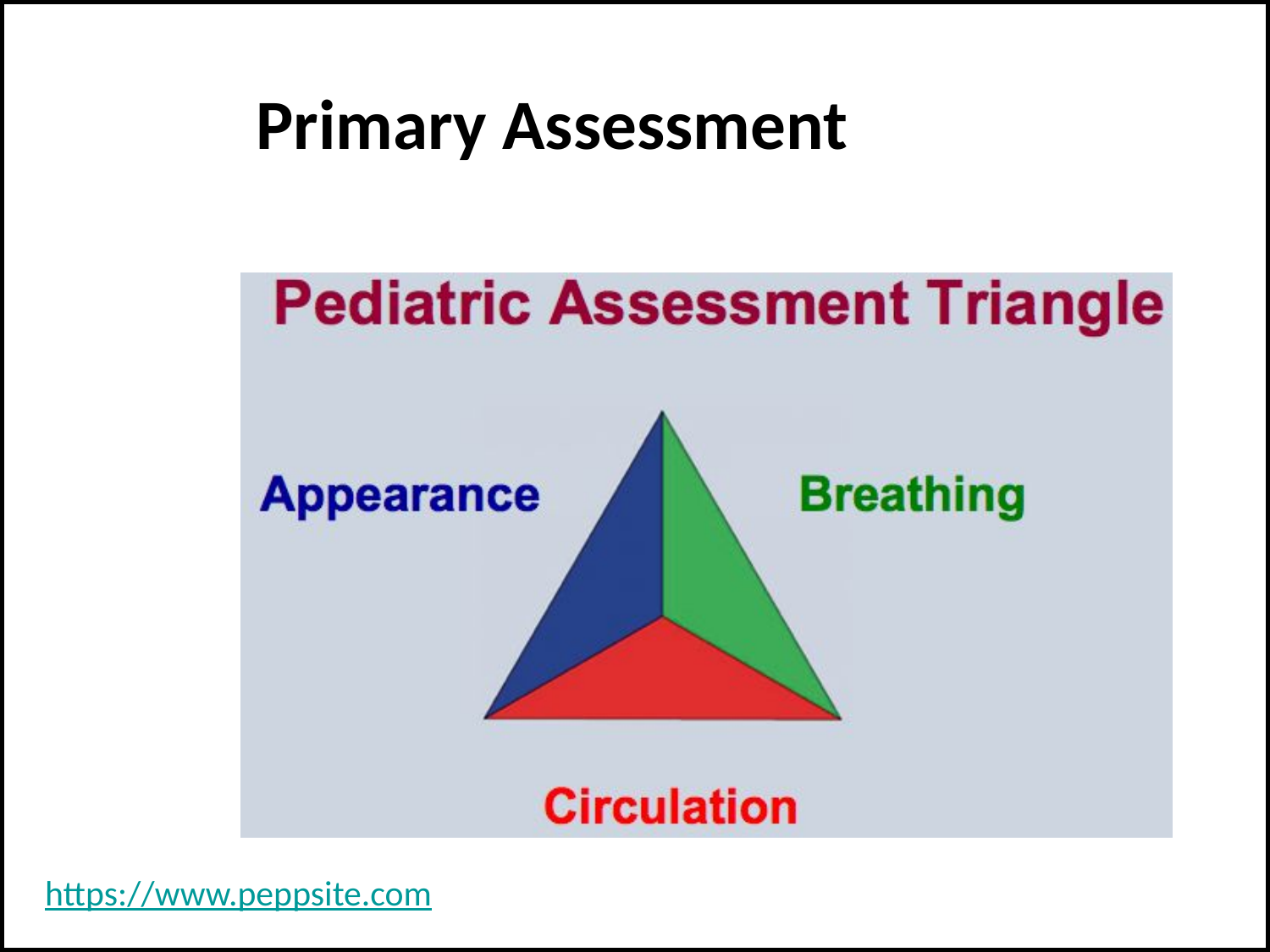

# Primary Assessment
https://www.peppsite.com

## Slide 7
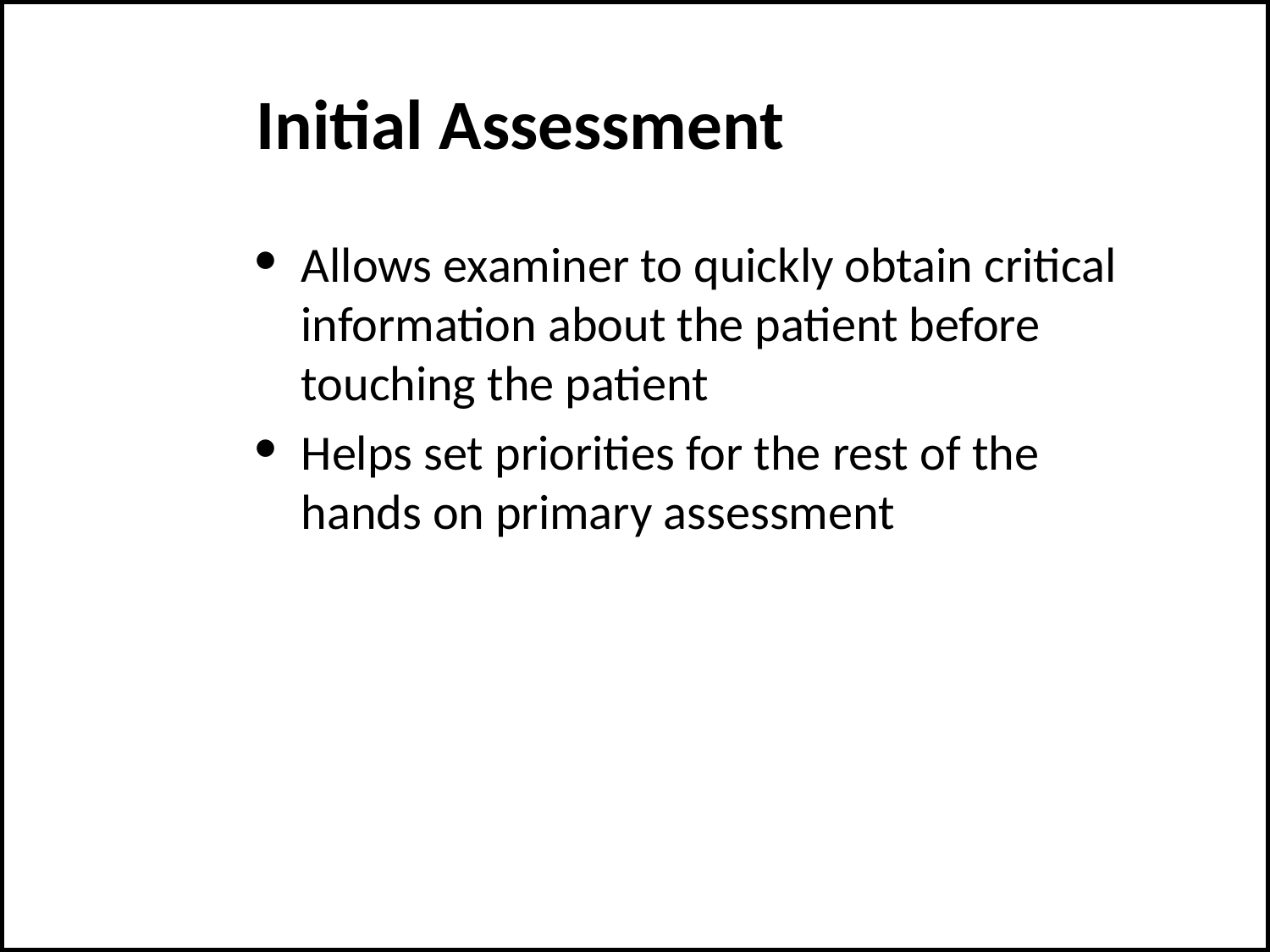

# Initial Assessment
Allows examiner to quickly obtain critical information about the patient before touching the patient
Helps set priorities for the rest of the hands on primary assessment

## Slide 8
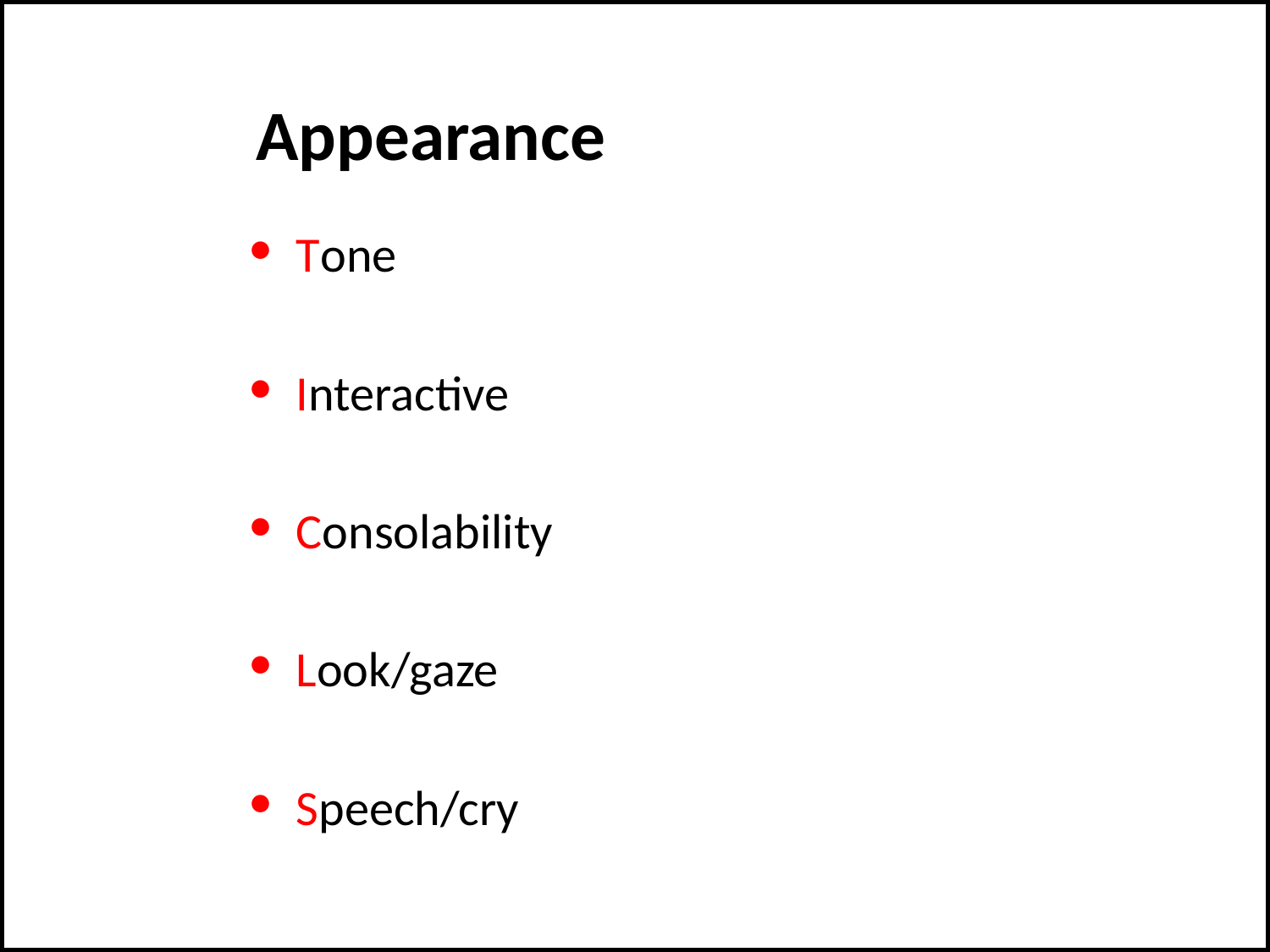

# Appearance
Tone
Interactive
Consolability
Look/gaze
Speech/cry

## Slide 9
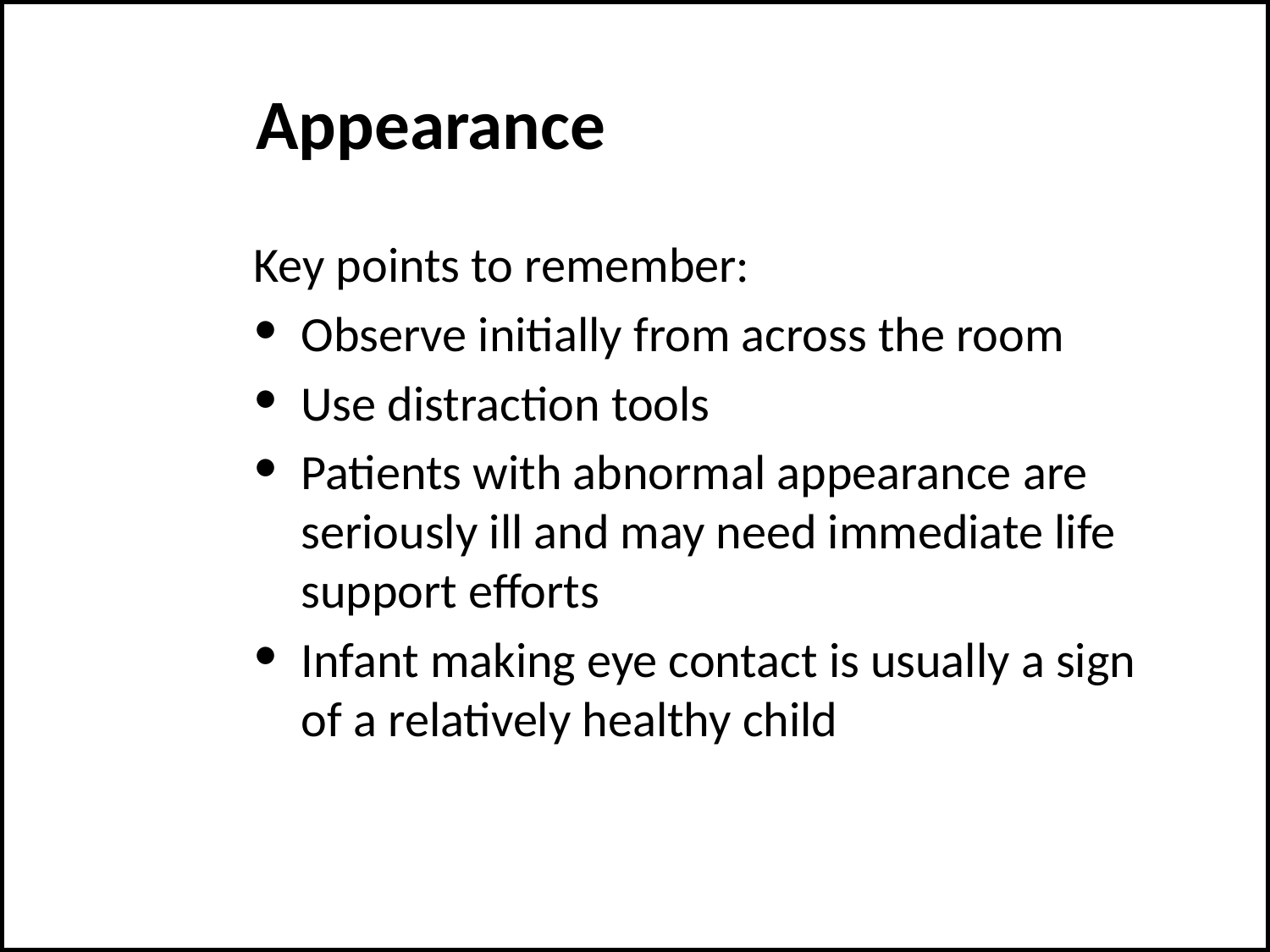

# Appearance
Key points to remember:
Observe initially from across the room
Use distraction tools
Patients with abnormal appearance are seriously ill and may need immediate life support efforts
Infant making eye contact is usually a sign of a relatively healthy child

## Slide 10
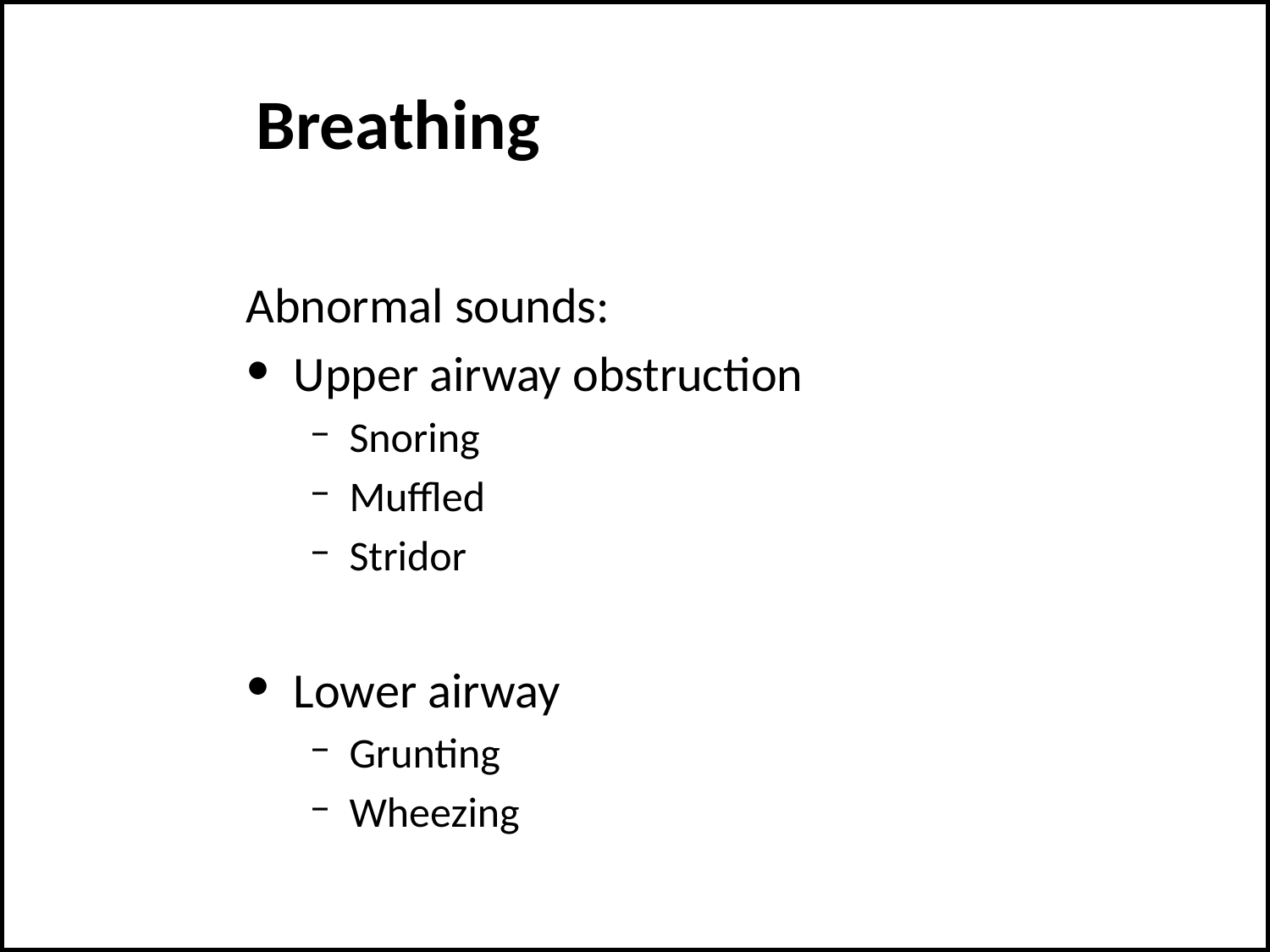

# Breathing
Abnormal sounds:
Upper airway obstruction
Snoring
Muffled
Stridor
Lower airway
Grunting
Wheezing

## Slide 11
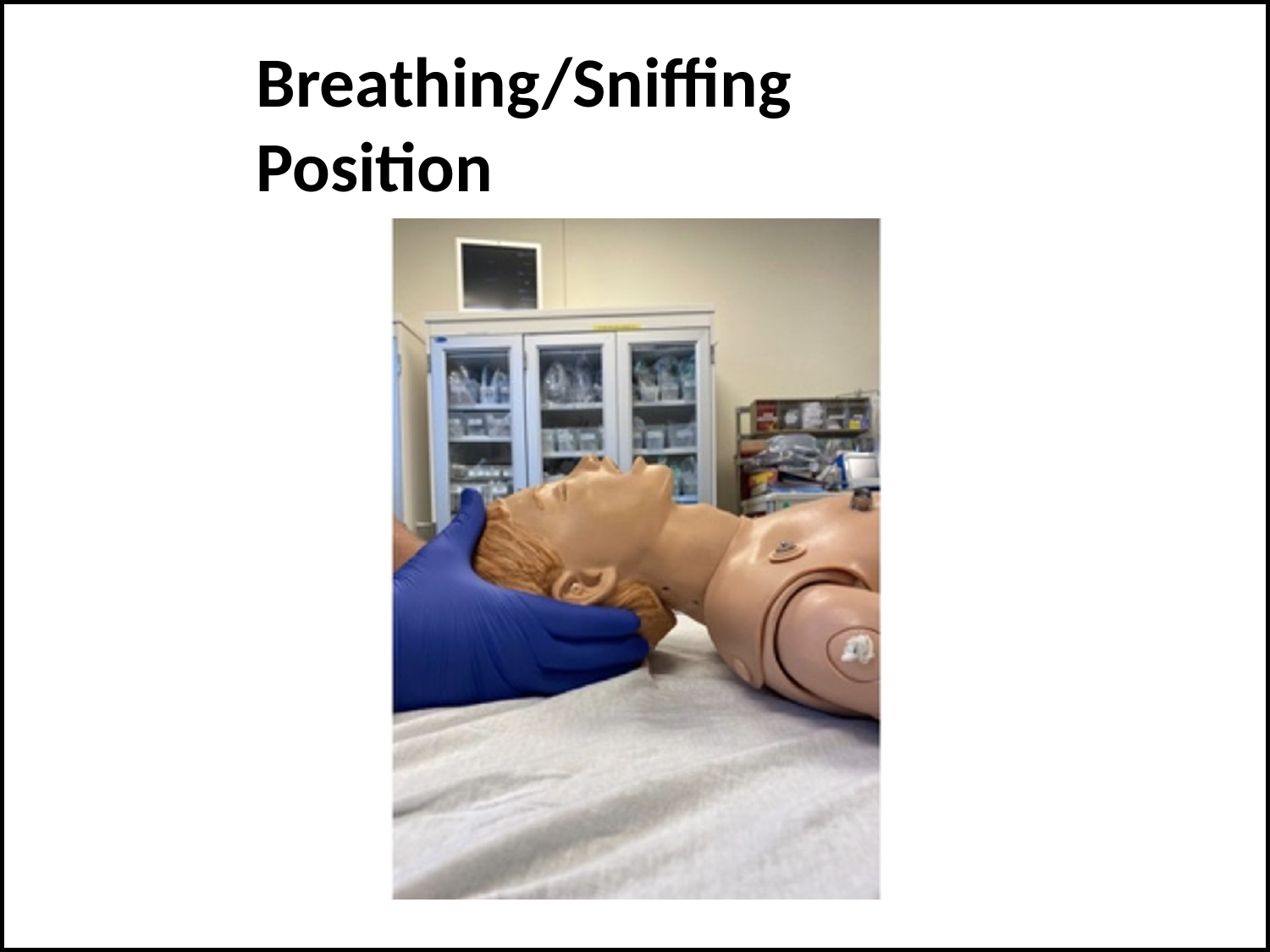

# Breathing/Sniffing Position

## Slide 12
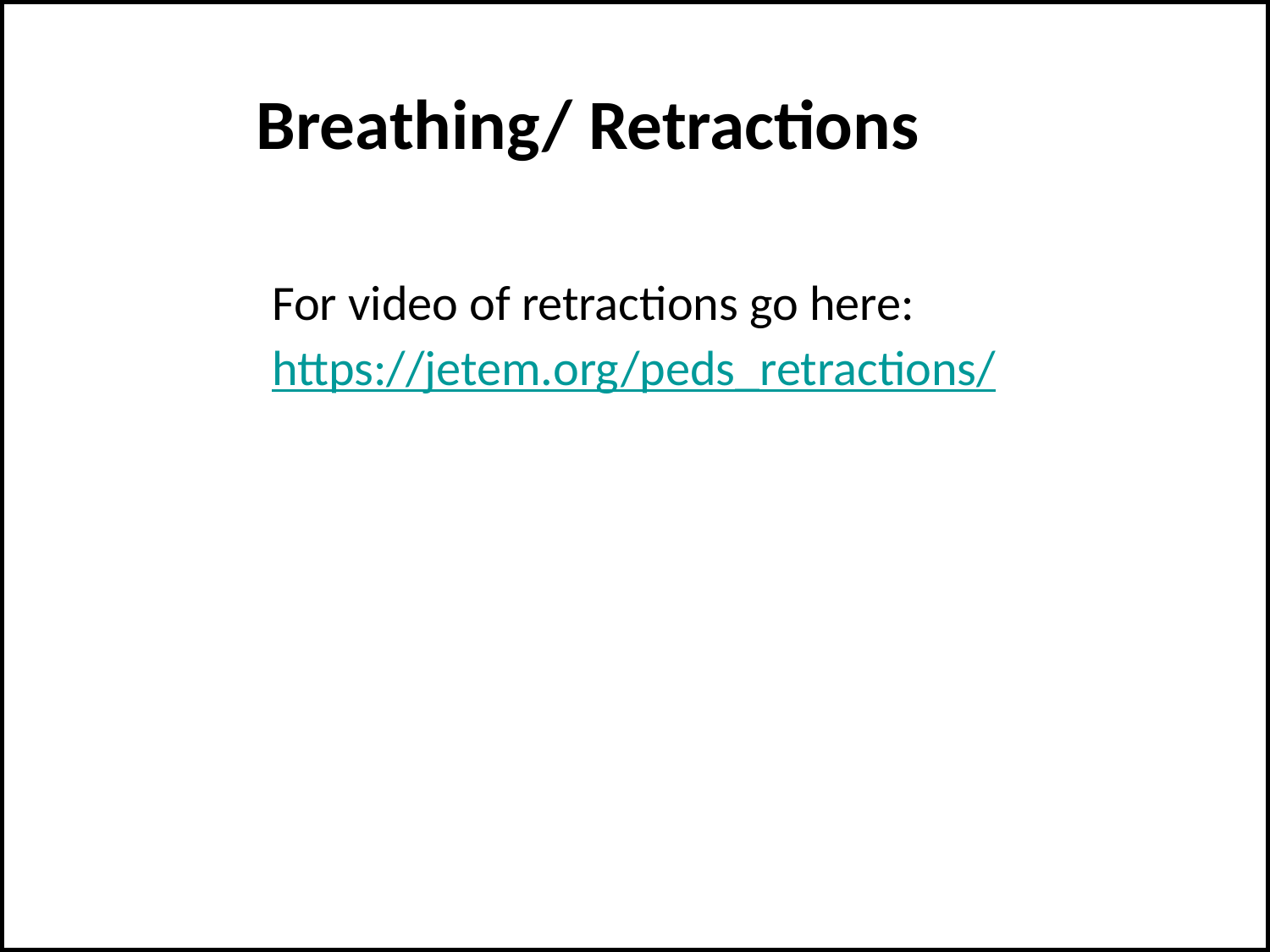

# Breathing/ Retractions
For video of retractions go here:
https://jetem.org/peds_retractions/

## Slide 13
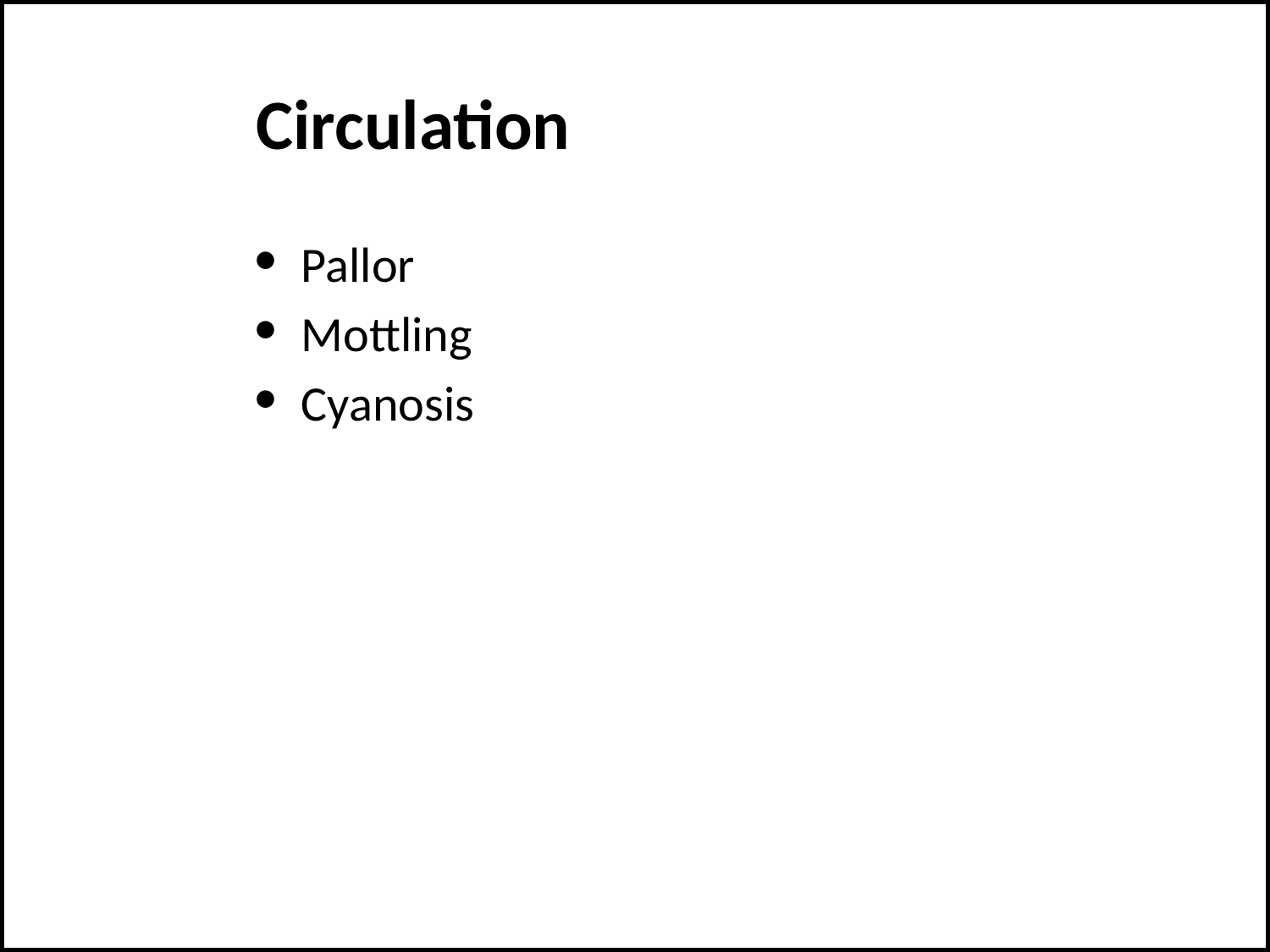

# Circulation
Pallor
Mottling
Cyanosis

## Slide 14
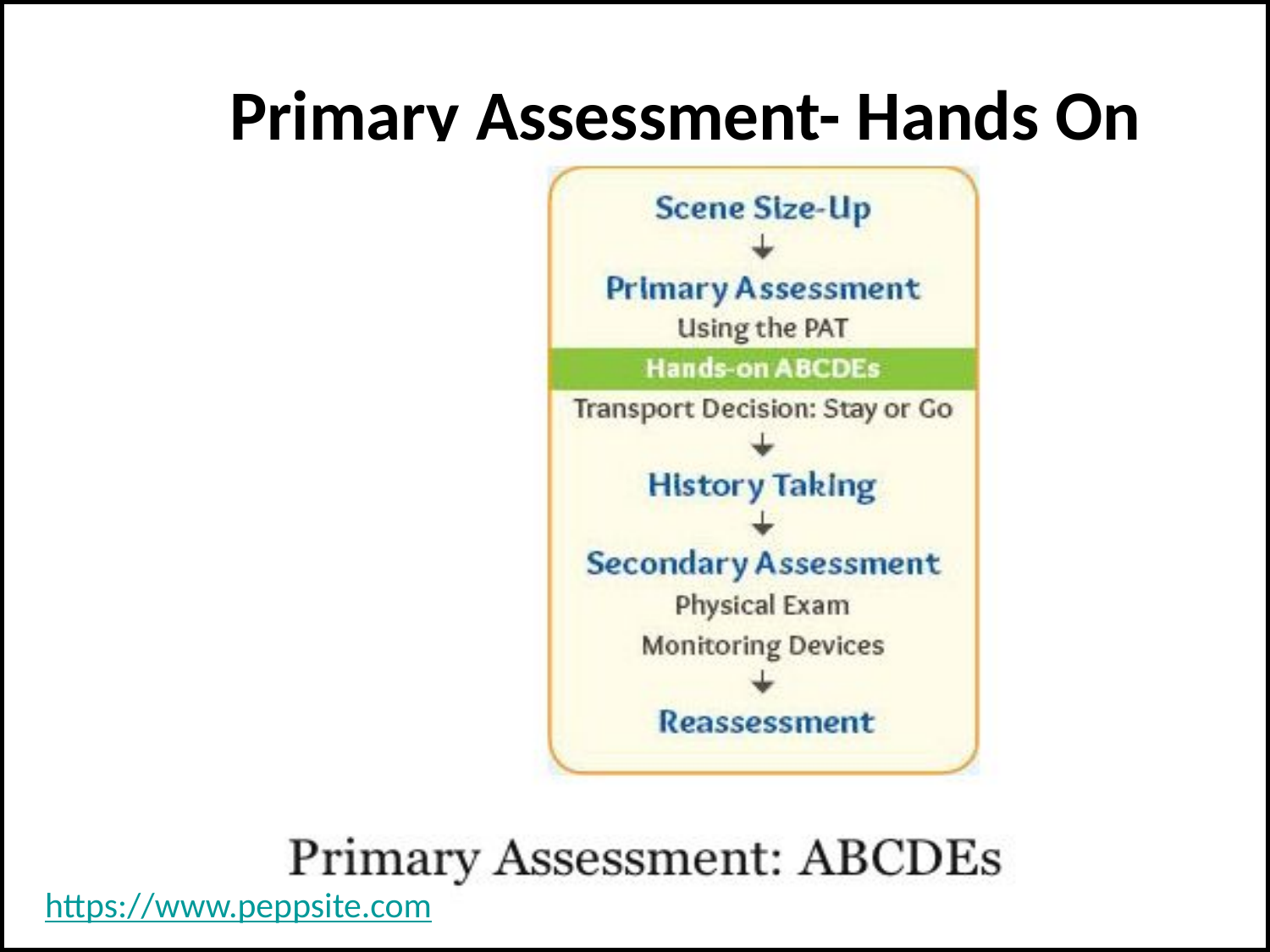

# Primary Assessment- Hands On
https://www.peppsite.com

## Slide 15
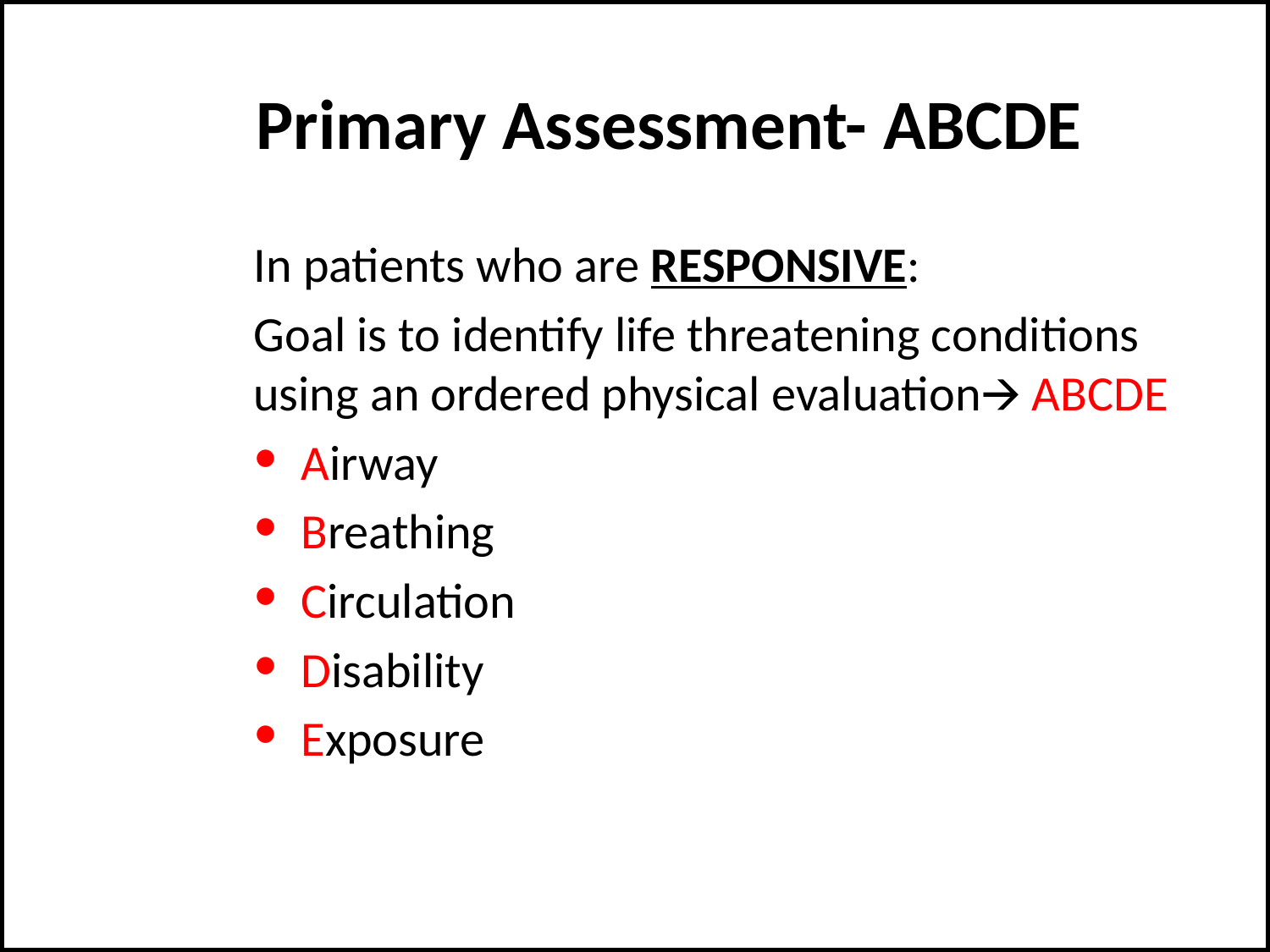

# Primary Assessment- ABCDE
In patients who are RESPONSIVE:
Goal is to identify life threatening conditions using an ordered physical evaluation🡪 ABCDE
Airway
Breathing
Circulation
Disability
Exposure

## Slide 16
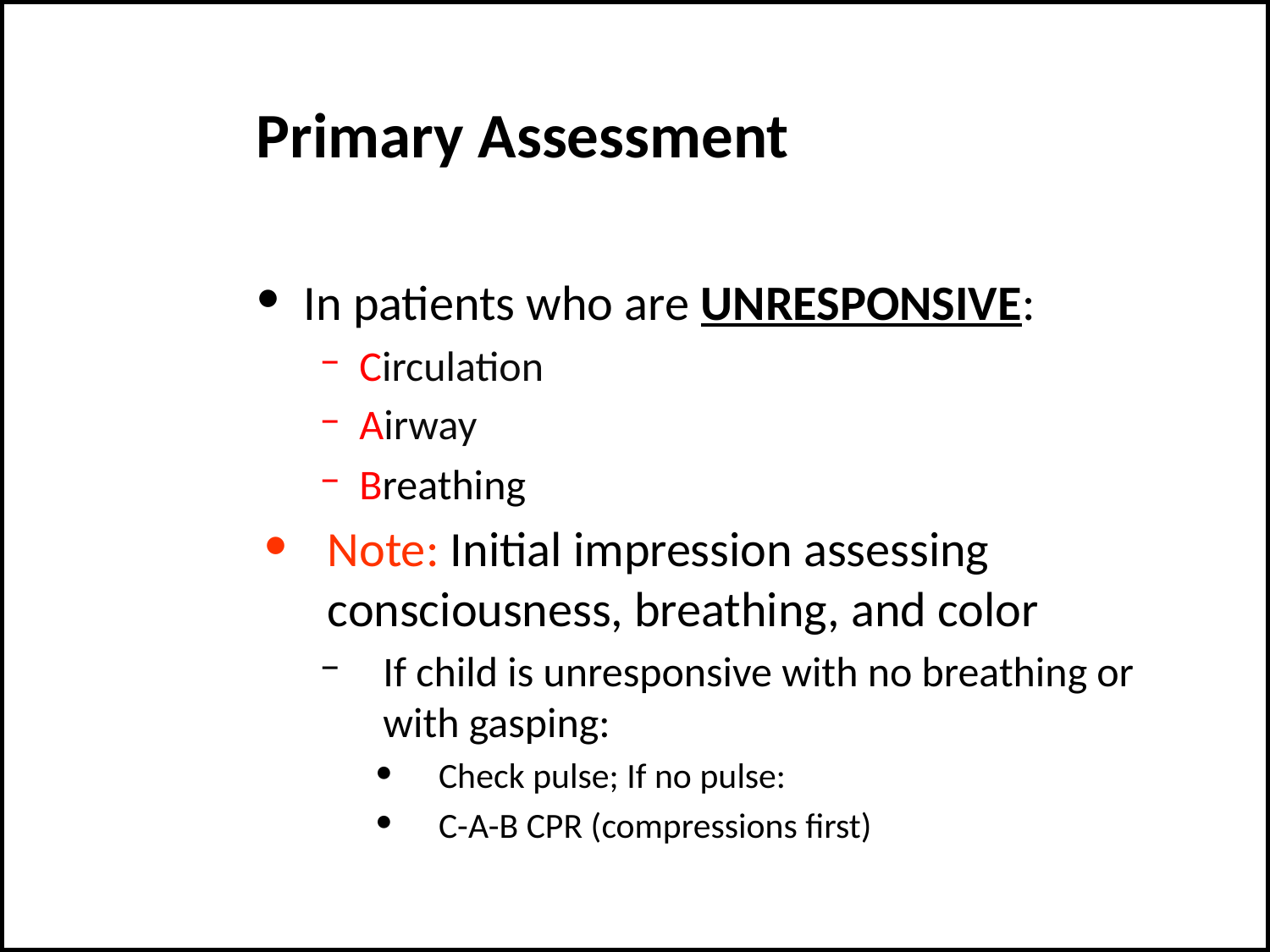

# Primary Assessment
In patients who are UNRESPONSIVE:
Circulation
Airway
Breathing
Note: Initial impression assessing consciousness, breathing, and color
If child is unresponsive with no breathing or with gasping:
Check pulse; If no pulse:
C-A-B CPR (compressions first)

## Slide 17
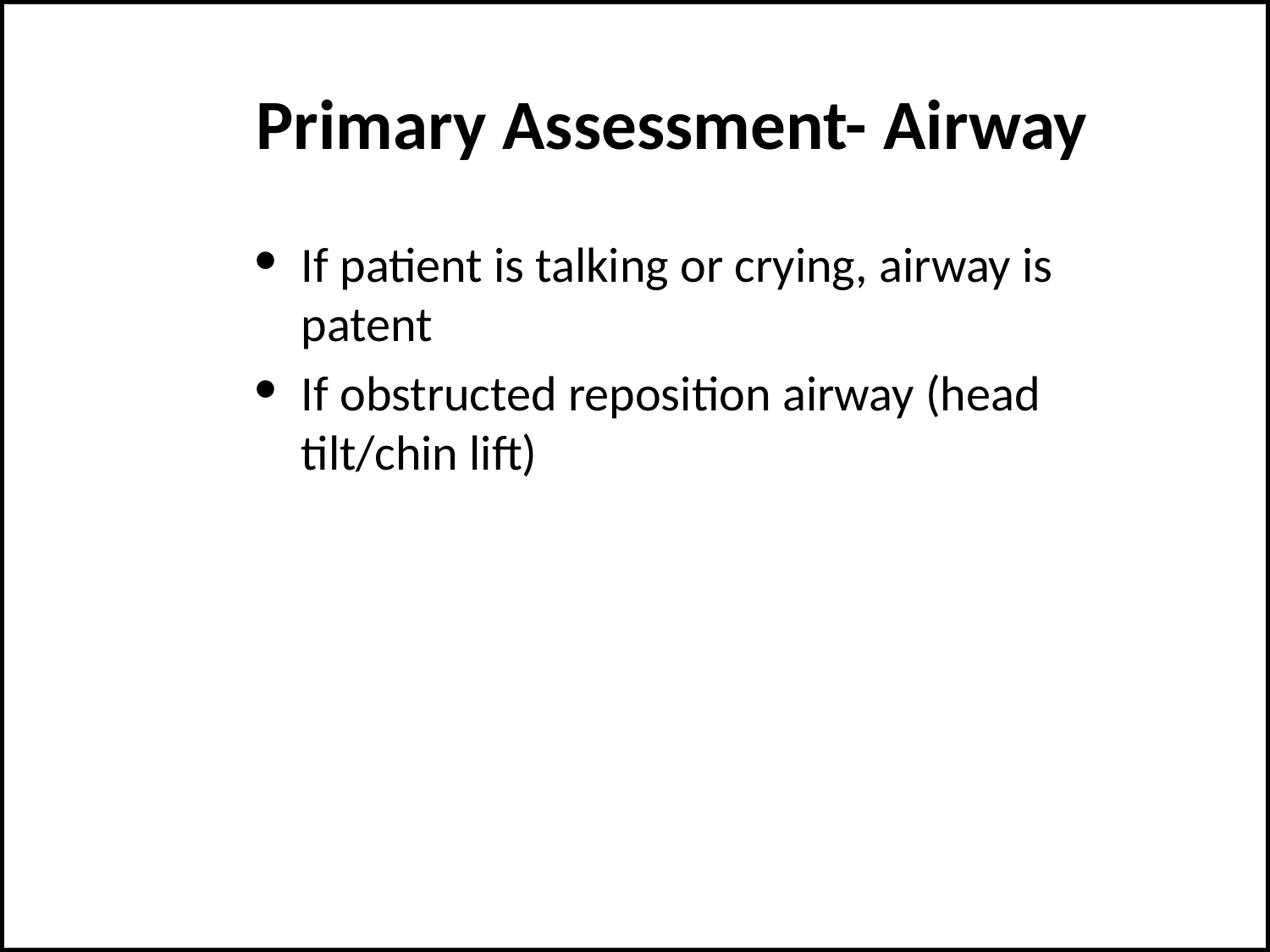

# Primary Assessment- Airway
If patient is talking or crying, airway is patent
If obstructed reposition airway (head tilt/chin lift)

## Slide 18
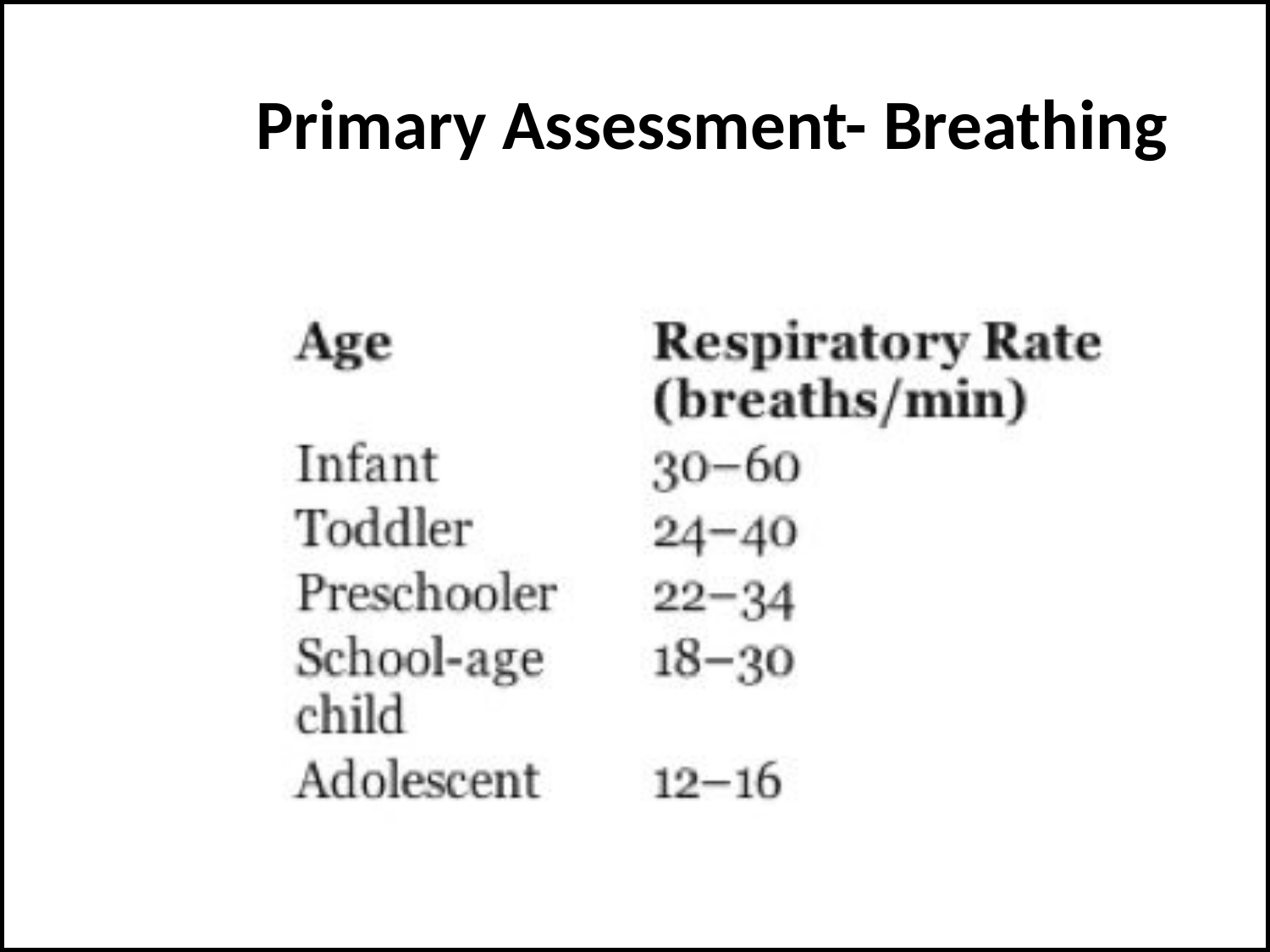

# Primary Assessment- Breathing

## Slide 19
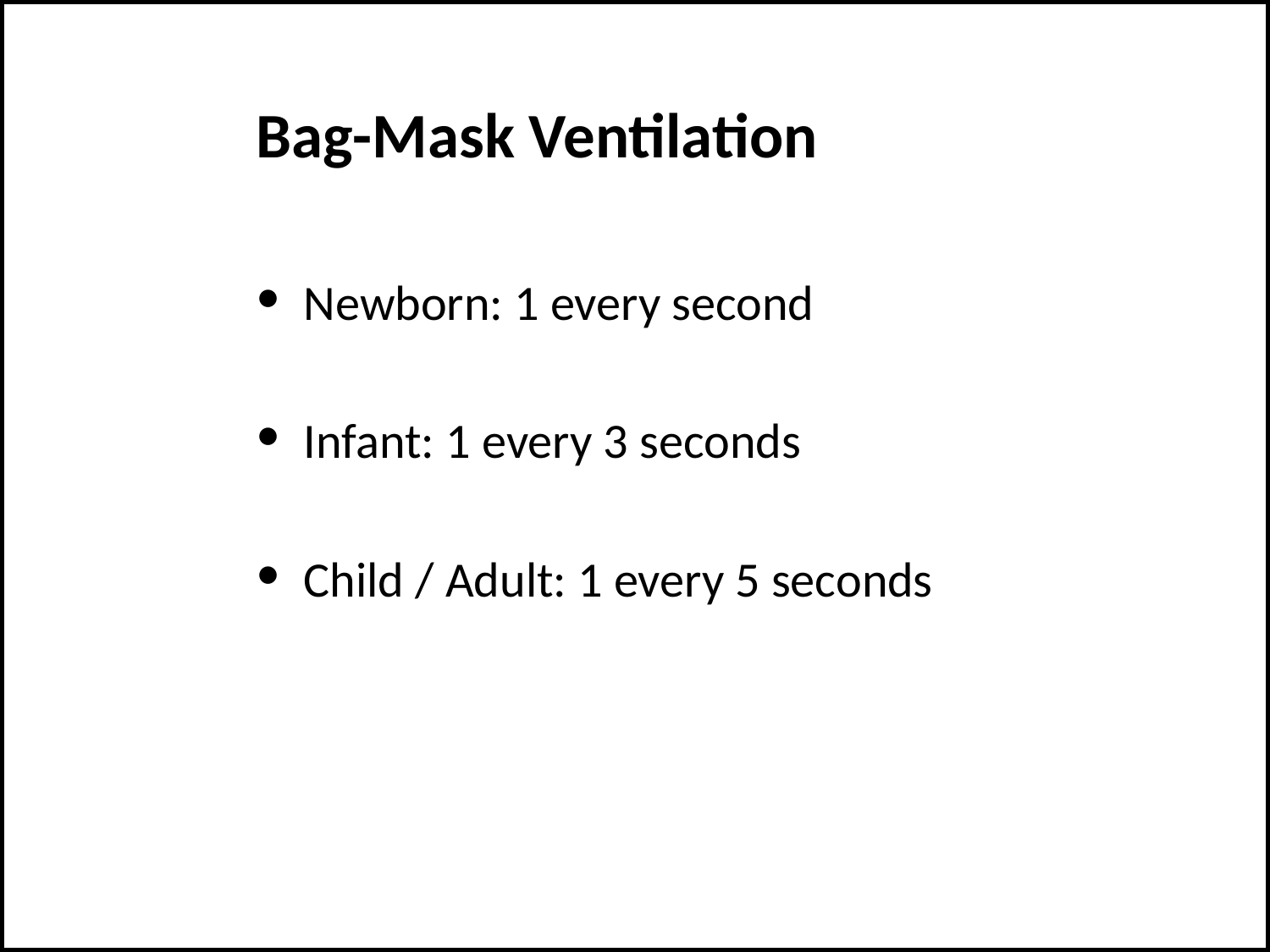

# Bag-Mask Ventilation
Newborn: 1 every second
Infant: 1 every 3 seconds
Child / Adult: 1 every 5 seconds

## Slide 20
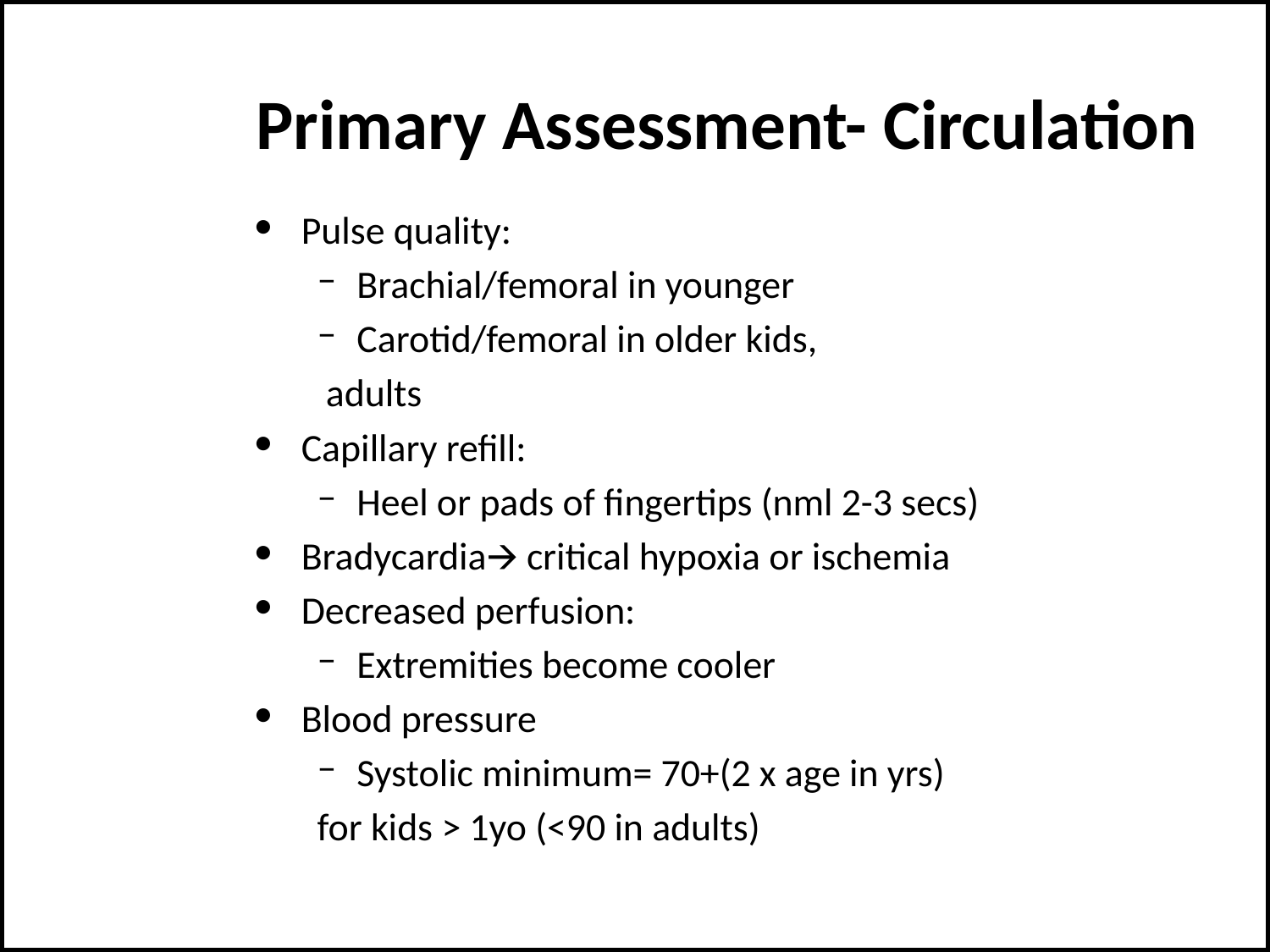

# Primary Assessment- Circulation
Pulse quality:
Brachial/femoral in younger
Carotid/femoral in older kids,
 adults
Capillary refill:
Heel or pads of fingertips (nml 2-3 secs)
Bradycardia🡪 critical hypoxia or ischemia
Decreased perfusion:
Extremities become cooler
Blood pressure
Systolic minimum= 70+(2 x age in yrs)
for kids > 1yo (<90 in adults)

## Slide 21
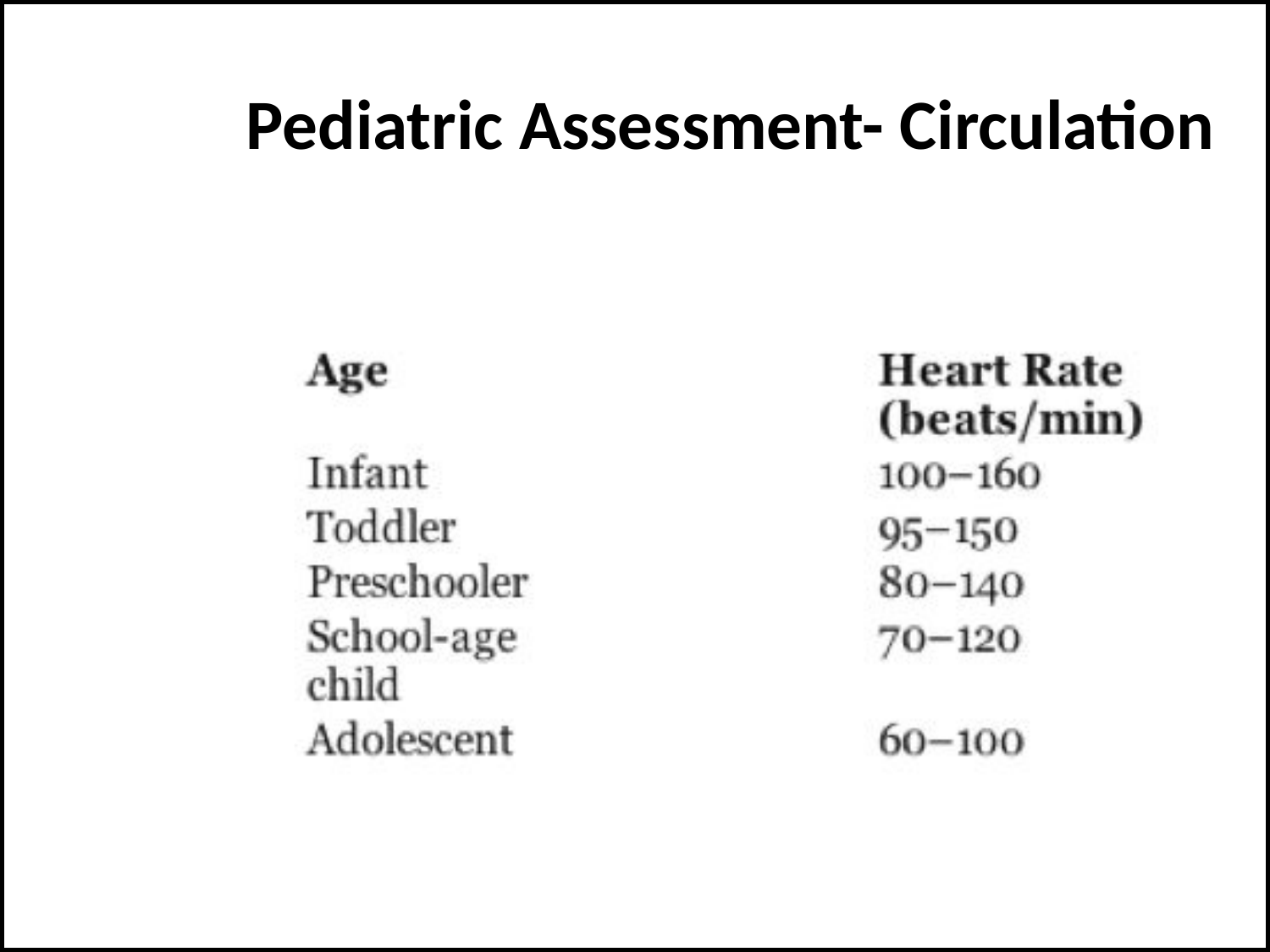

# Pediatric Assessment- Circulation

## Slide 22
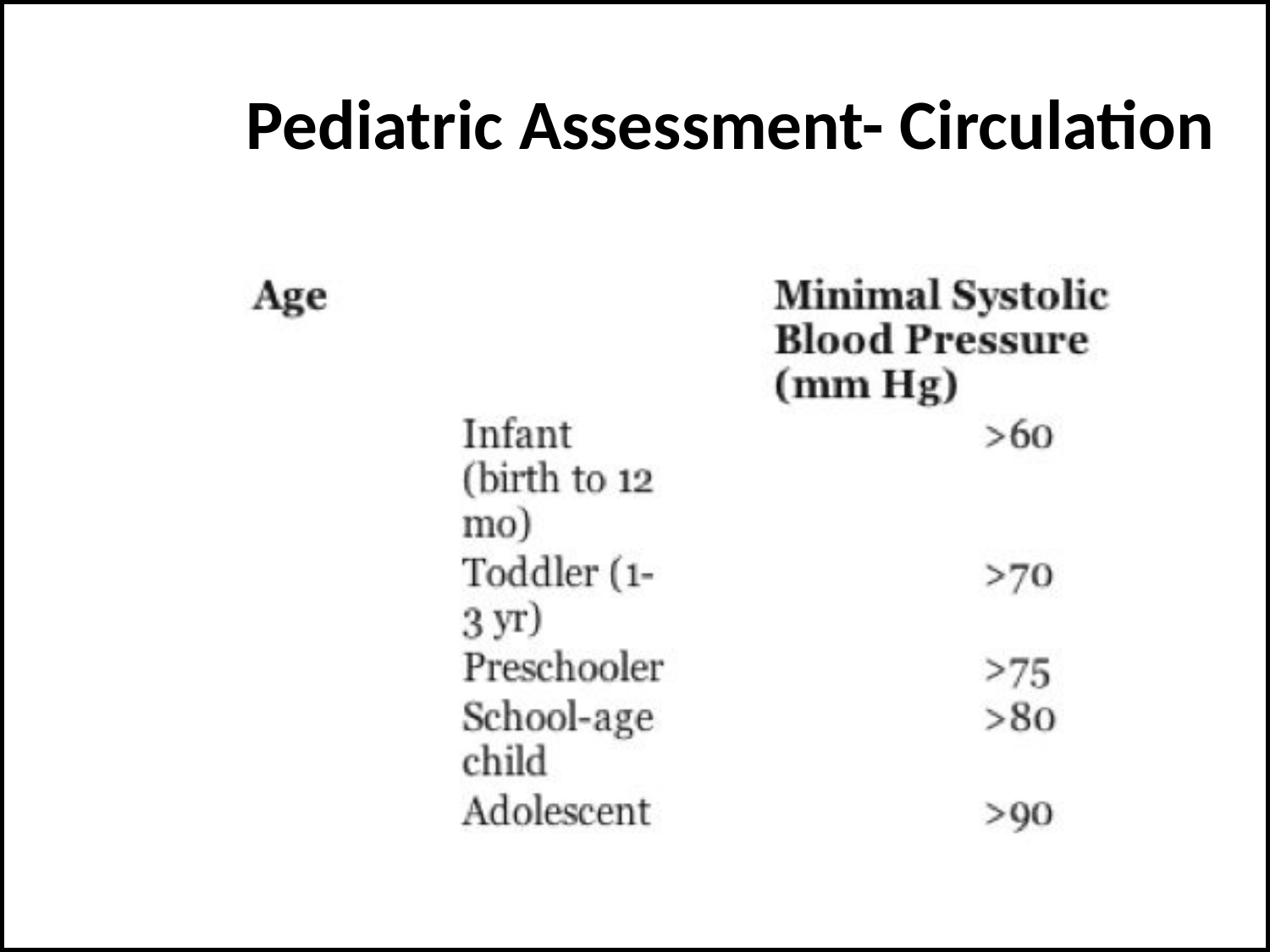

# Pediatric Assessment- Circulation

## Slide 23
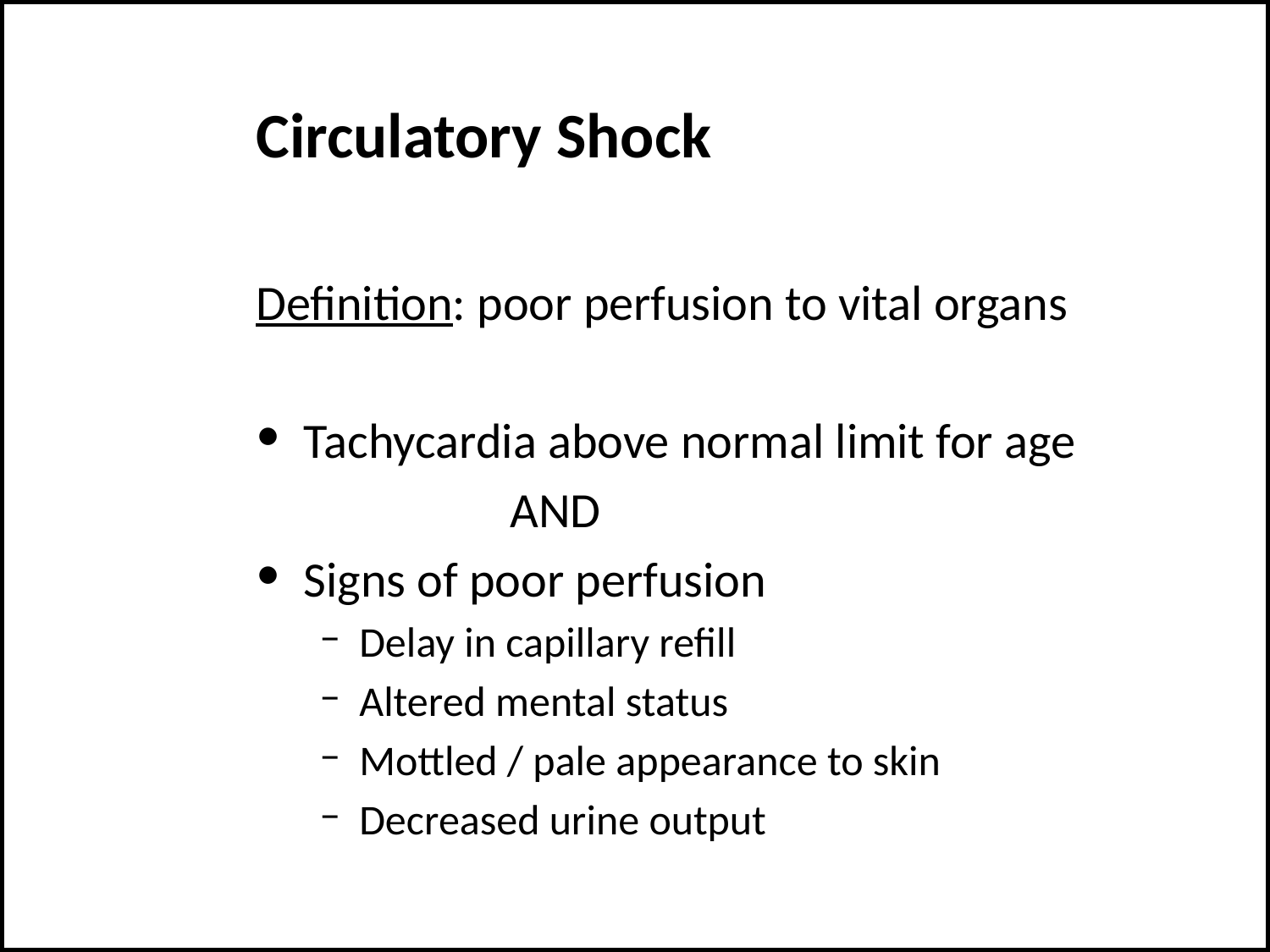

# Circulatory Shock
Definition: poor perfusion to vital organs
Tachycardia above normal limit for age
		AND
Signs of poor perfusion
Delay in capillary refill
Altered mental status
Mottled / pale appearance to skin
Decreased urine output

## Slide 24
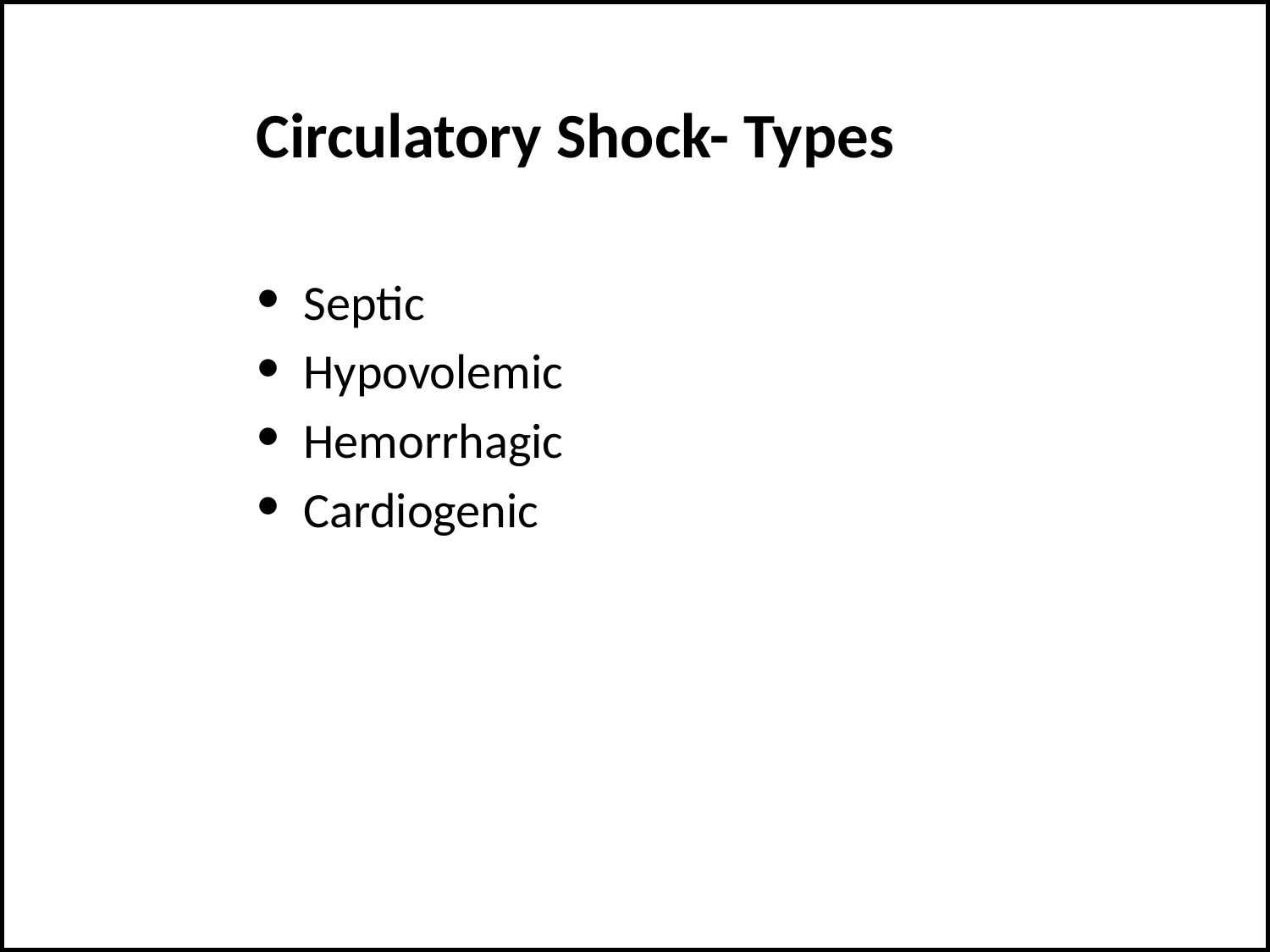

# Circulatory Shock- Types
Septic
Hypovolemic
Hemorrhagic
Cardiogenic

## Slide 25
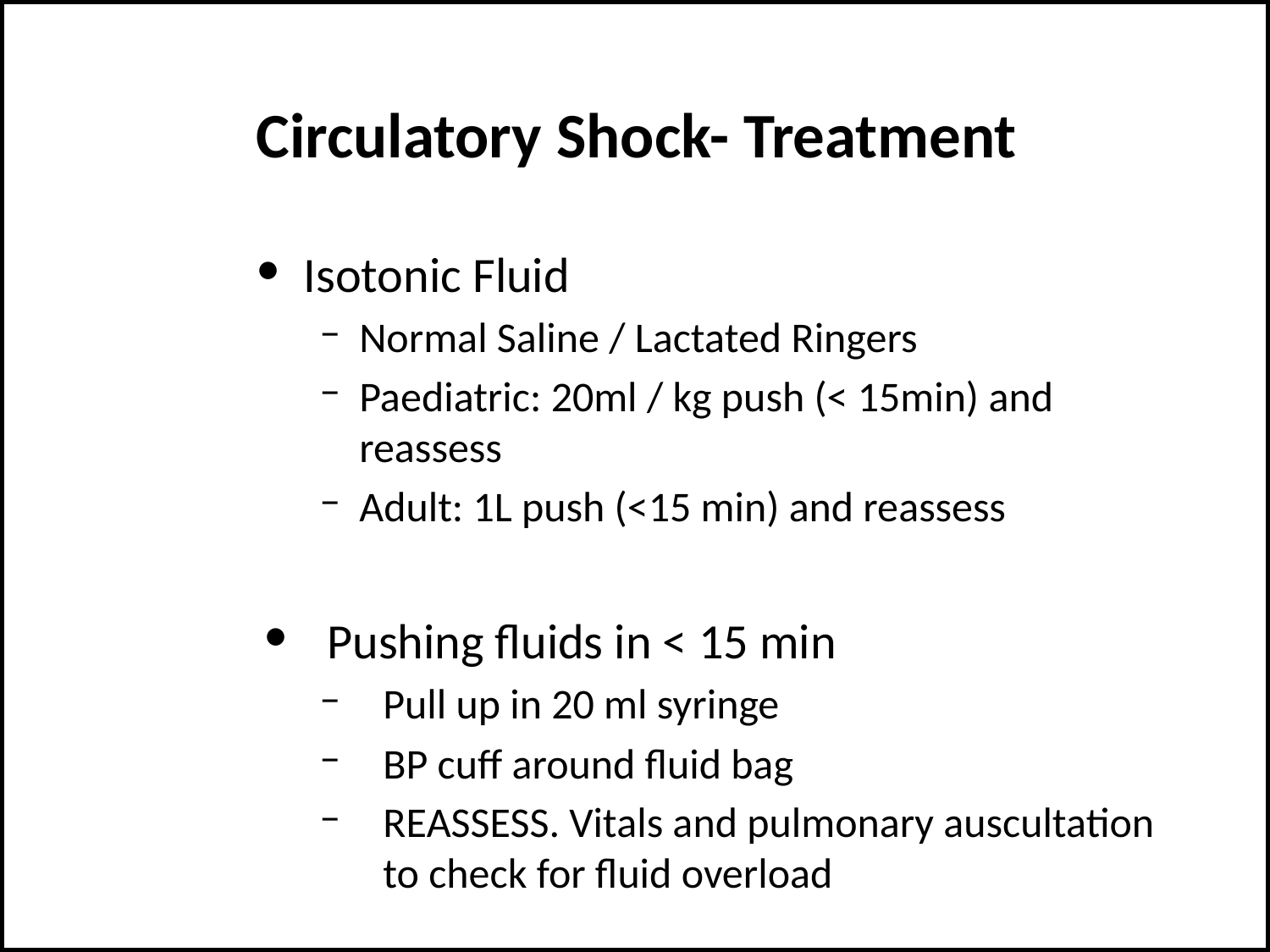

# Circulatory Shock- Treatment
Isotonic Fluid
Normal Saline / Lactated Ringers
Paediatric: 20ml / kg push (< 15min) and reassess
Adult: 1L push (<15 min) and reassess
Pushing fluids in < 15 min
Pull up in 20 ml syringe
BP cuff around fluid bag
REASSESS. Vitals and pulmonary auscultation to check for fluid overload

## Slide 26
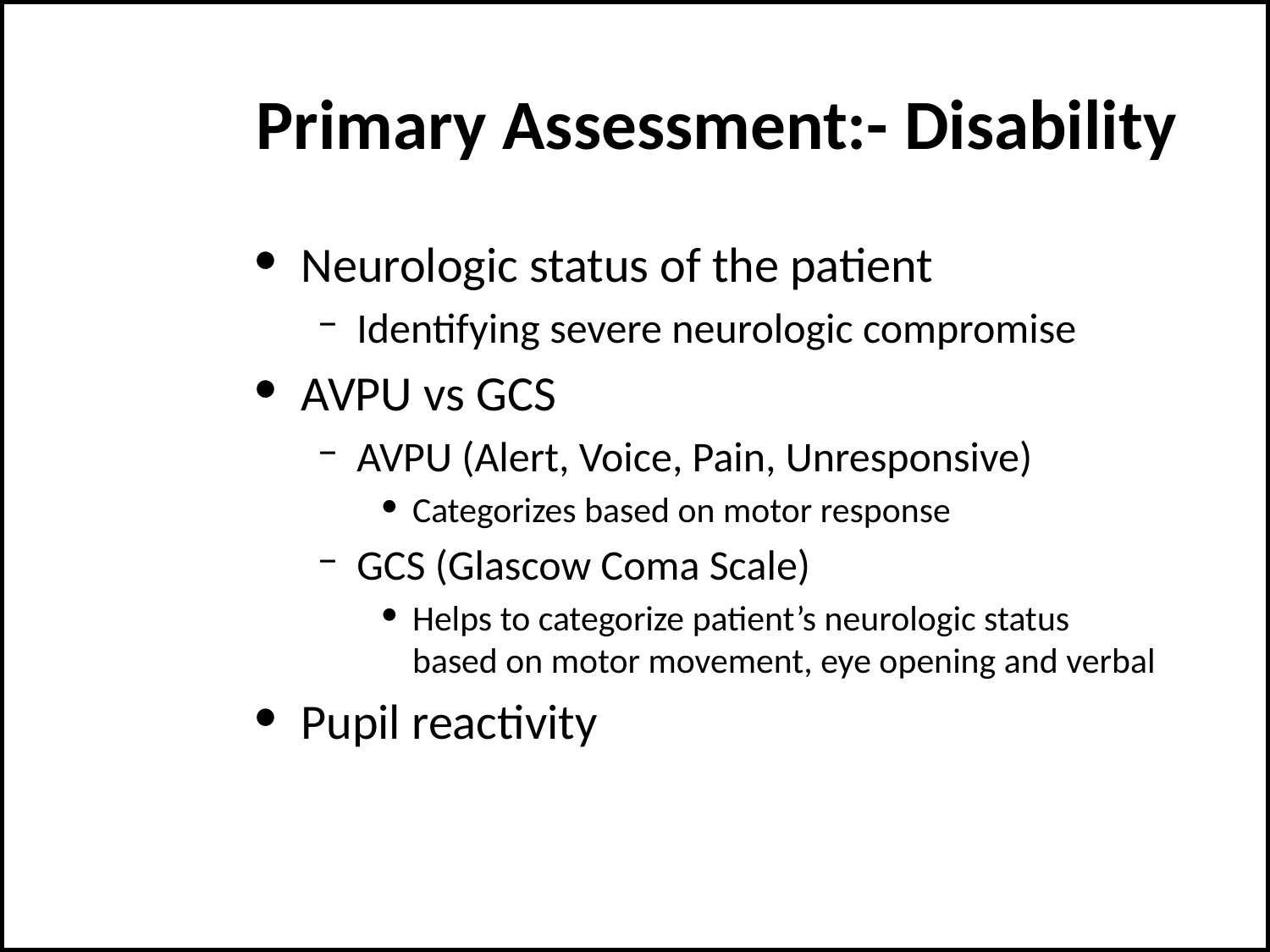

# Primary Assessment:- Disability
Neurologic status of the patient
Identifying severe neurologic compromise
AVPU vs GCS
AVPU (Alert, Voice, Pain, Unresponsive)
Categorizes based on motor response
GCS (Glascow Coma Scale)
Helps to categorize patient’s neurologic status based on motor movement, eye opening and verbal
Pupil reactivity

## Slide 27
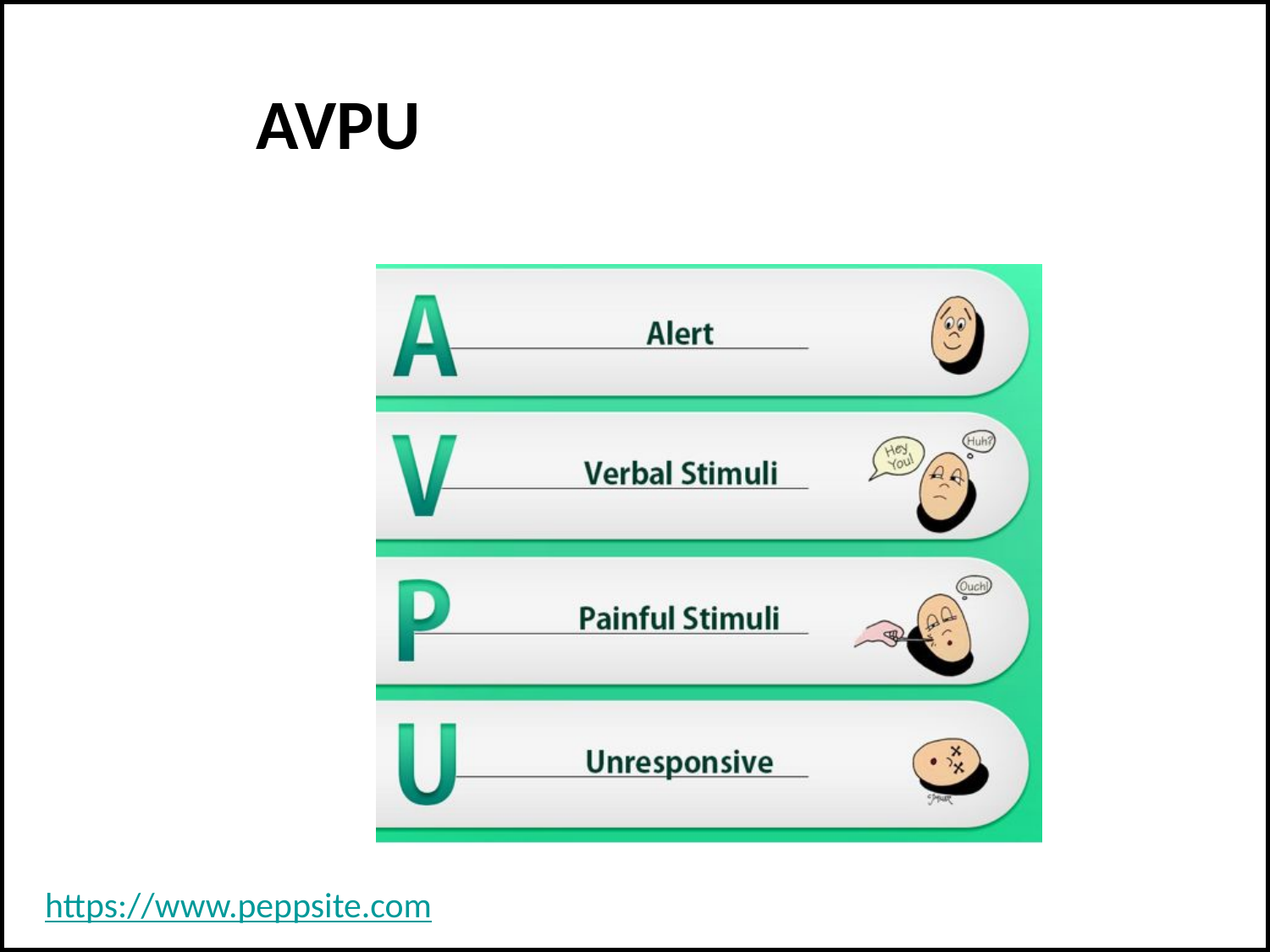

# AVPU
https://www.peppsite.com

## Slide 28
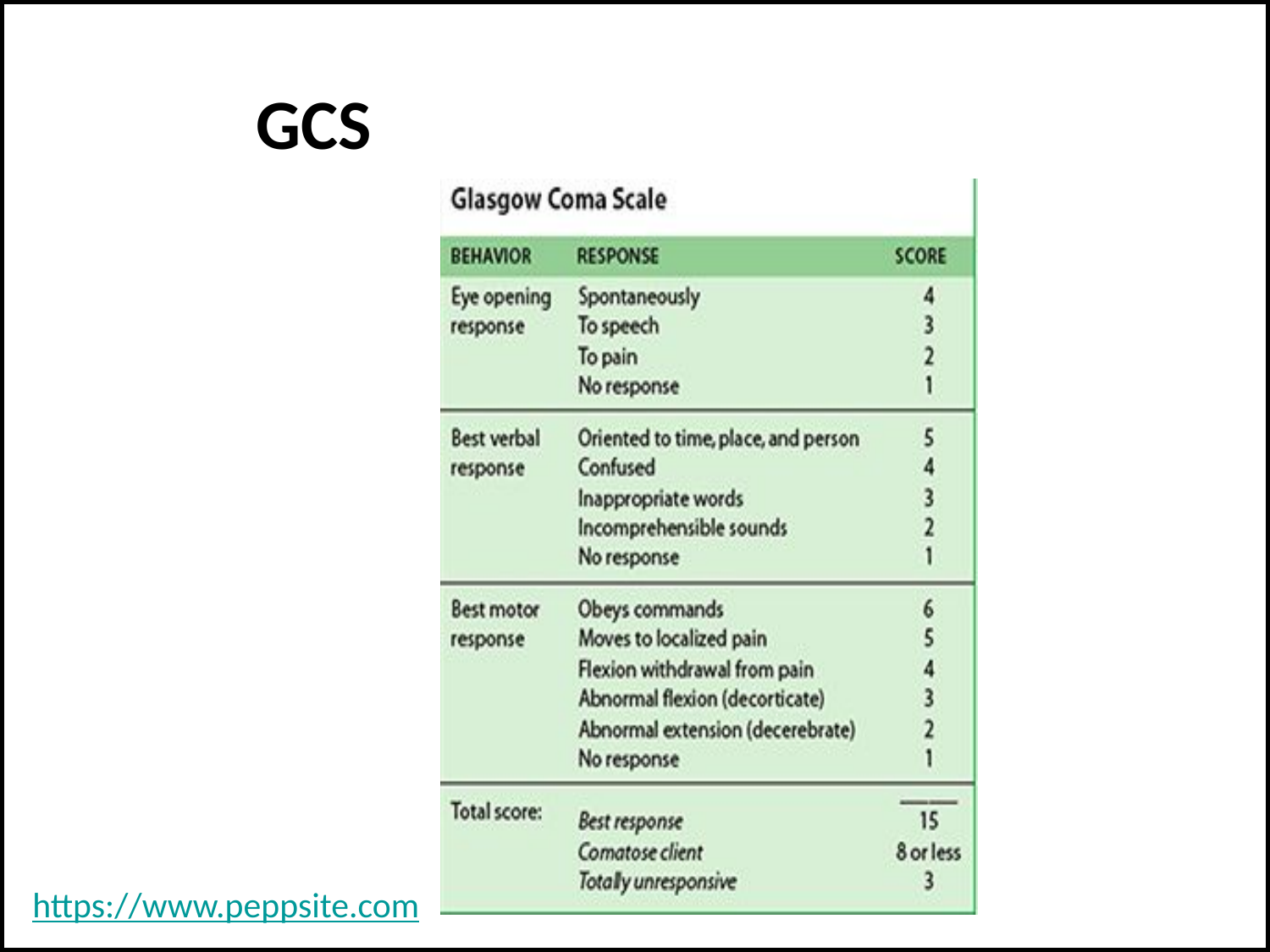

# GCS
https://www.peppsite.com

## Slide 29
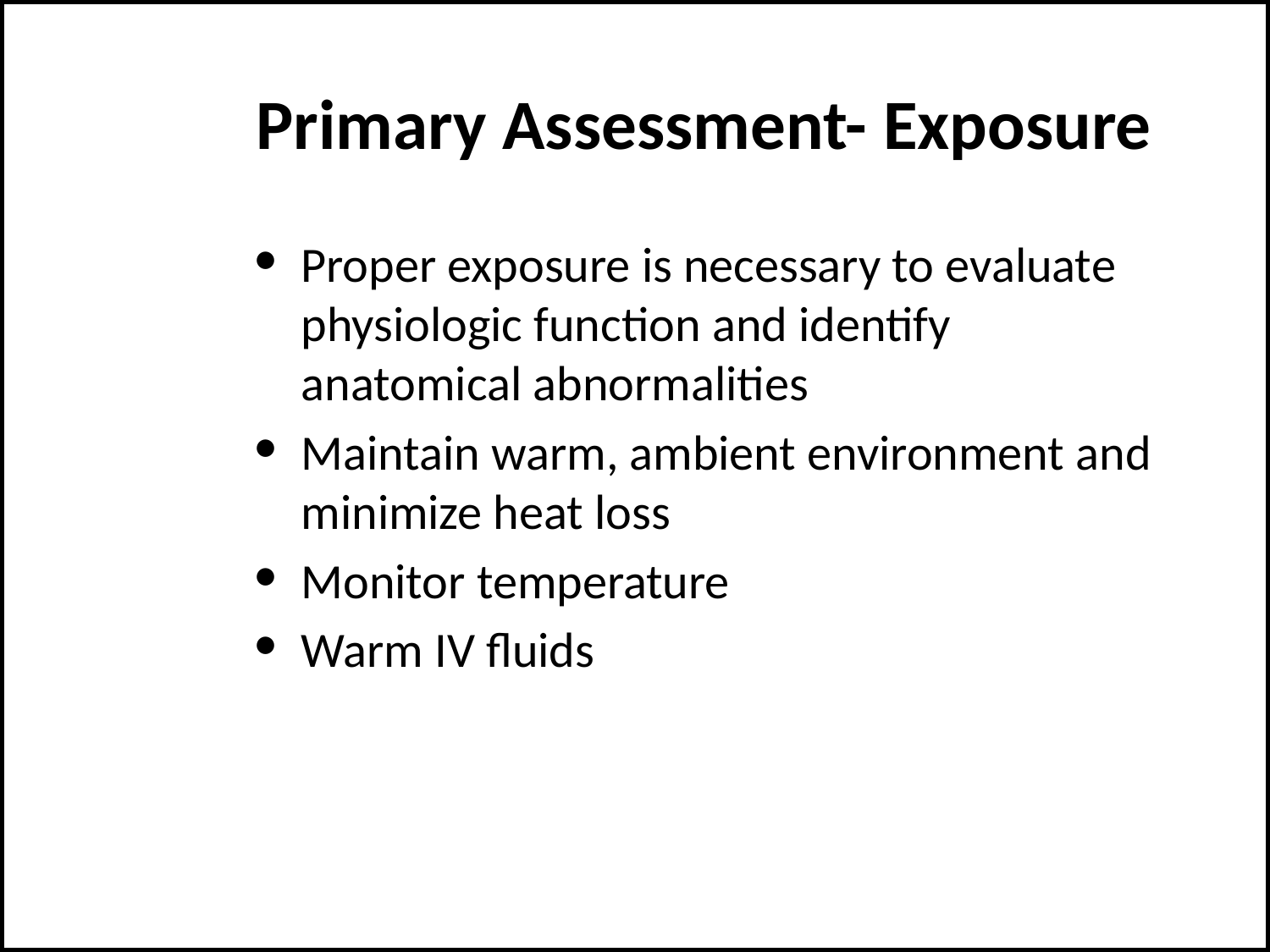

# Primary Assessment- Exposure
Proper exposure is necessary to evaluate physiologic function and identify anatomical abnormalities
Maintain warm, ambient environment and minimize heat loss
Monitor temperature
Warm IV fluids

## Slide 30
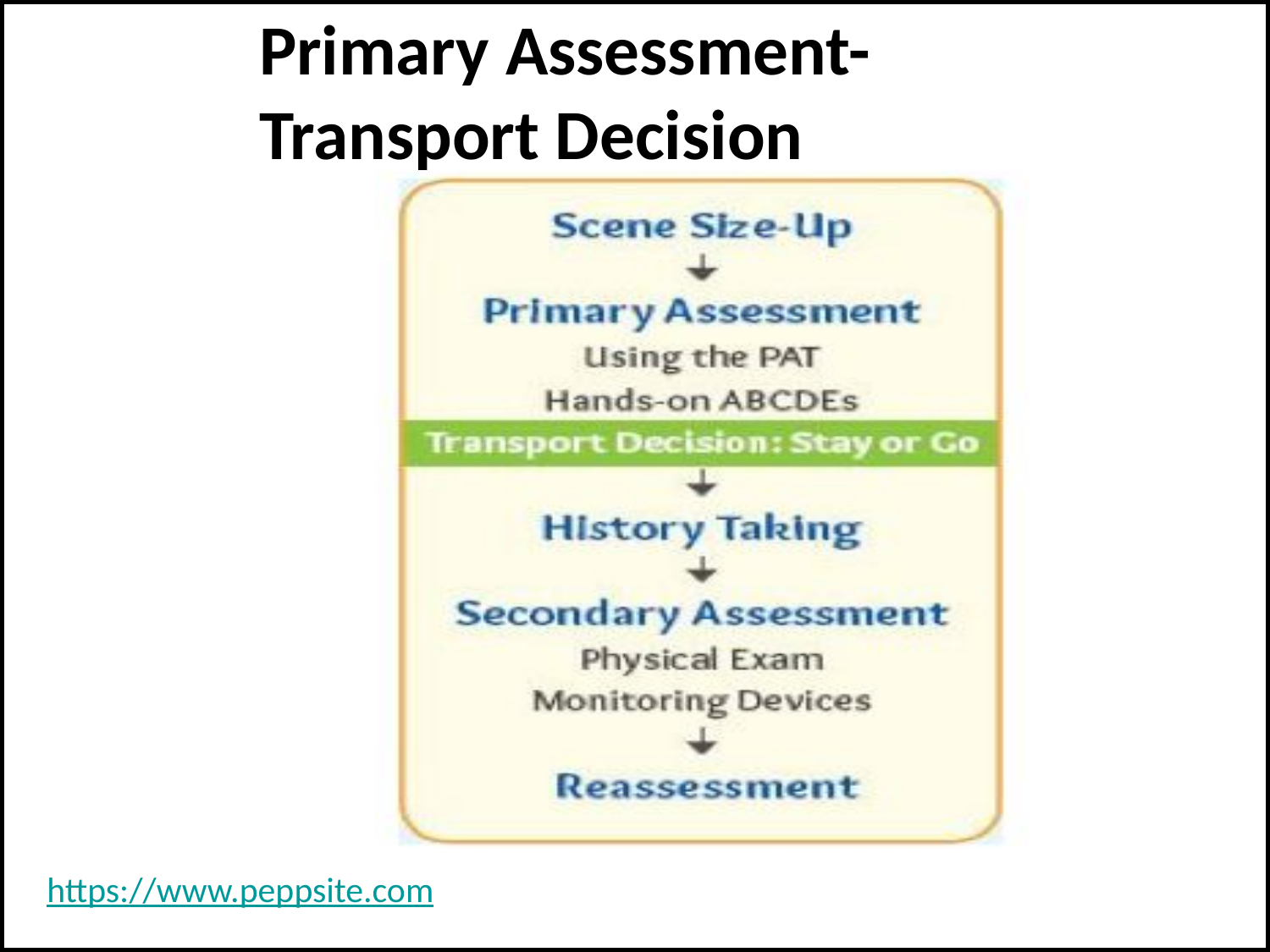

# Primary Assessment- Transport Decision
https://www.peppsite.com

## Slide 31
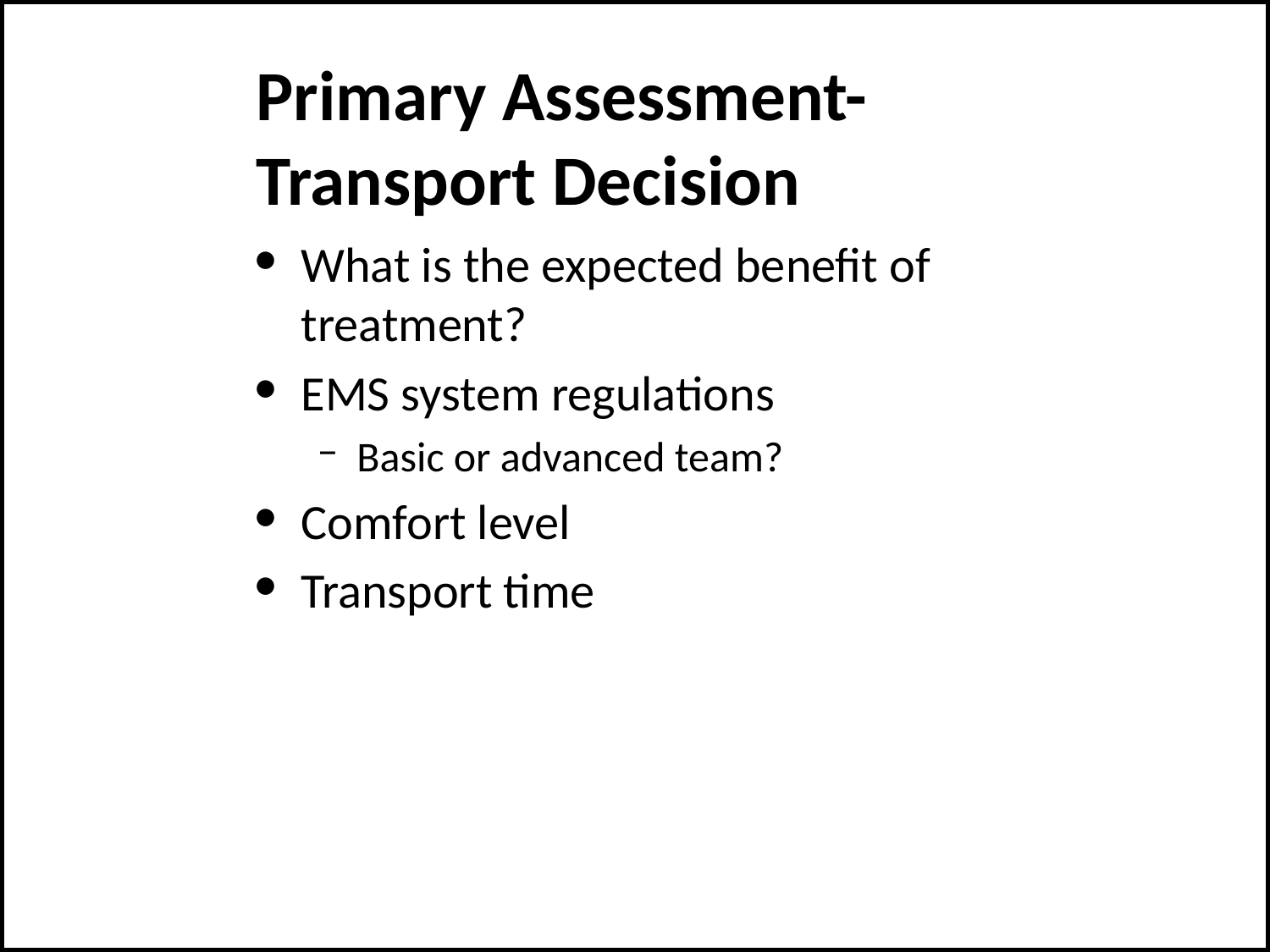

# Primary Assessment- Transport Decision
What is the expected benefit of treatment?
EMS system regulations
Basic or advanced team?
Comfort level
Transport time

## Slide 32
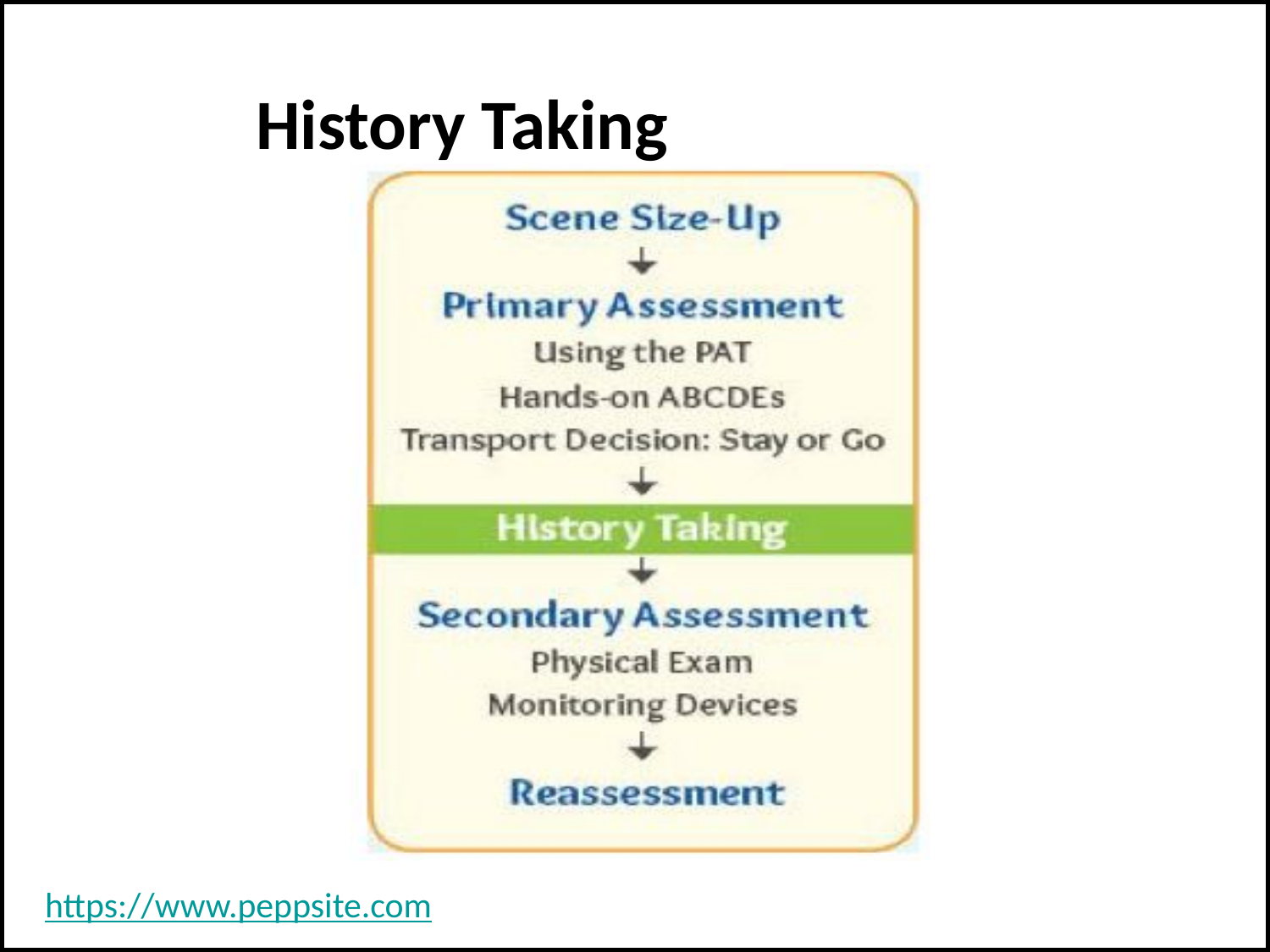

# History Taking
https://www.peppsite.com

## Slide 33
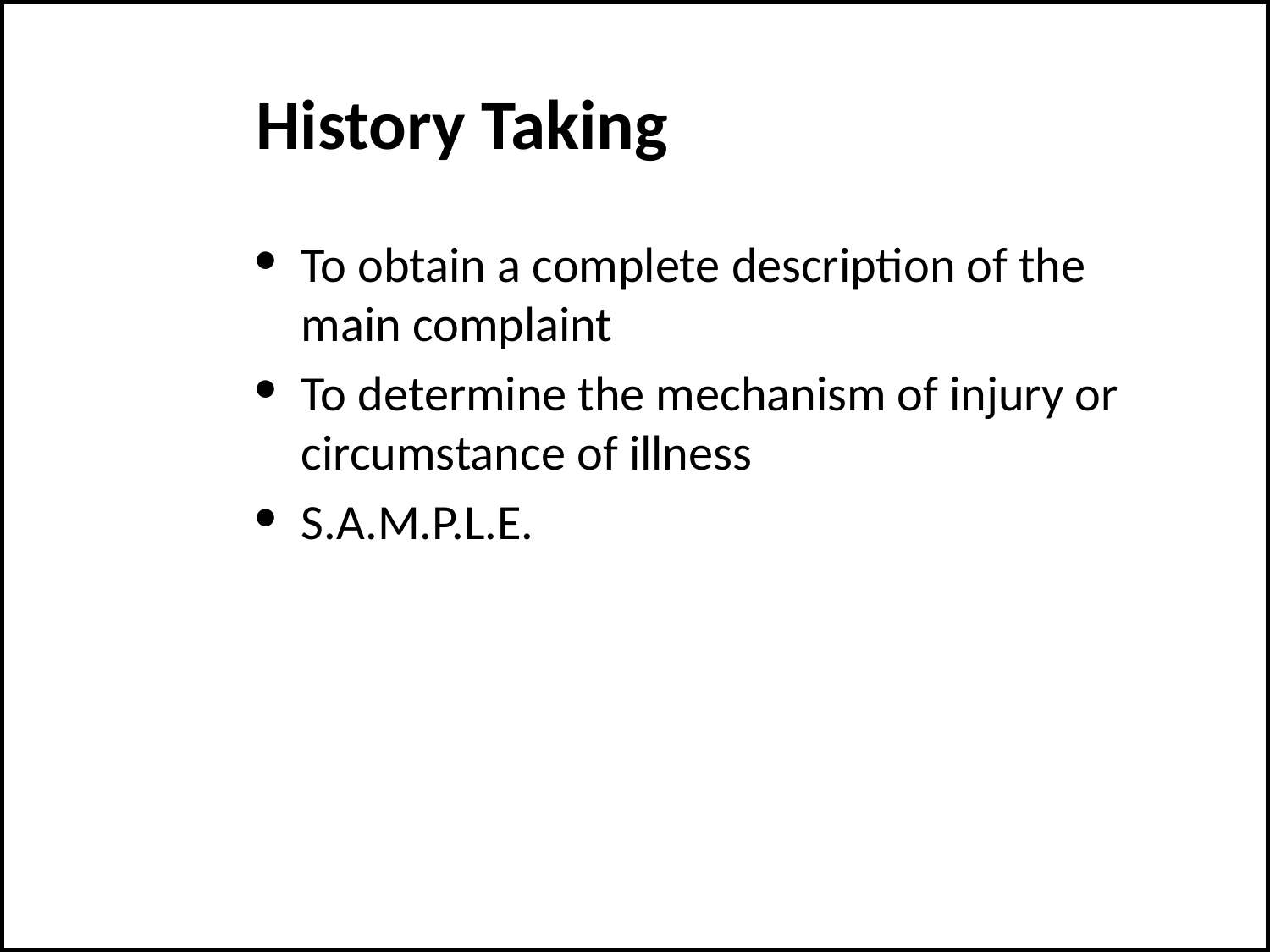

# History Taking
To obtain a complete description of the main complaint
To determine the mechanism of injury or circumstance of illness
S.A.M.P.L.E.

## Slide 34
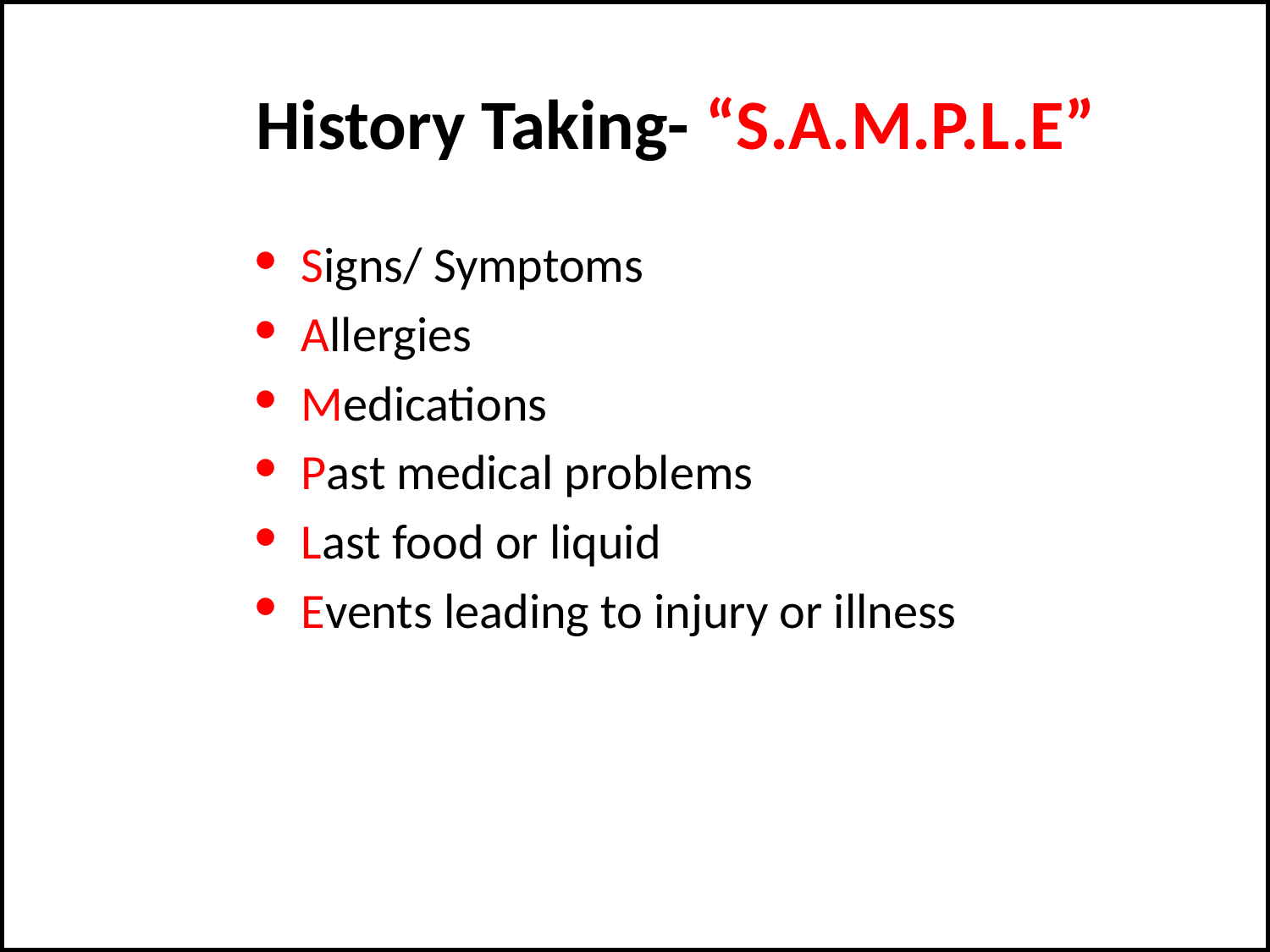

# History Taking- “S.A.M.P.L.E”
Signs/ Symptoms
Allergies
Medications
Past medical problems
Last food or liquid
Events leading to injury or illness

## Slide 35
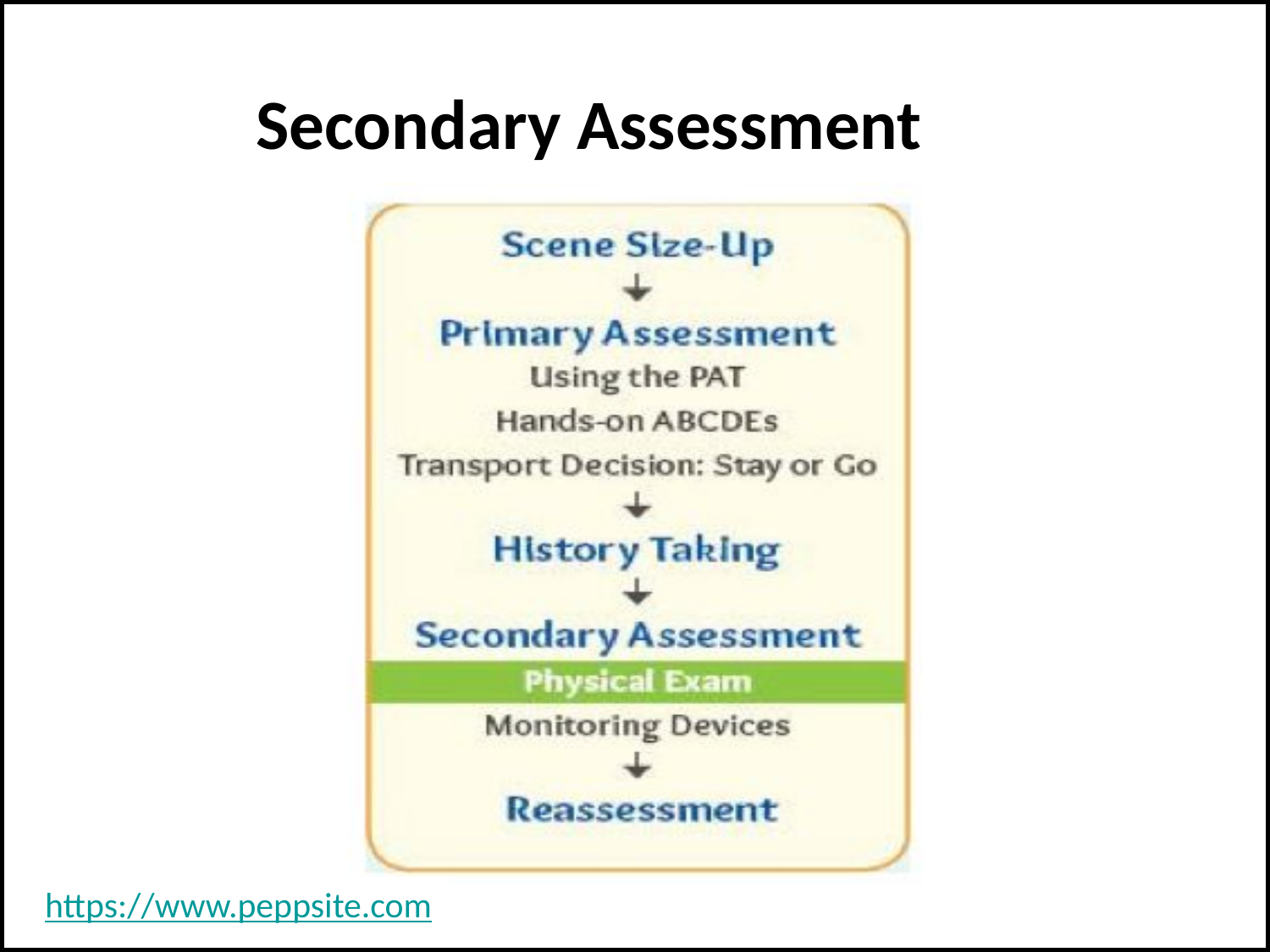

# Secondary Assessment
https://www.peppsite.com

## Slide 36
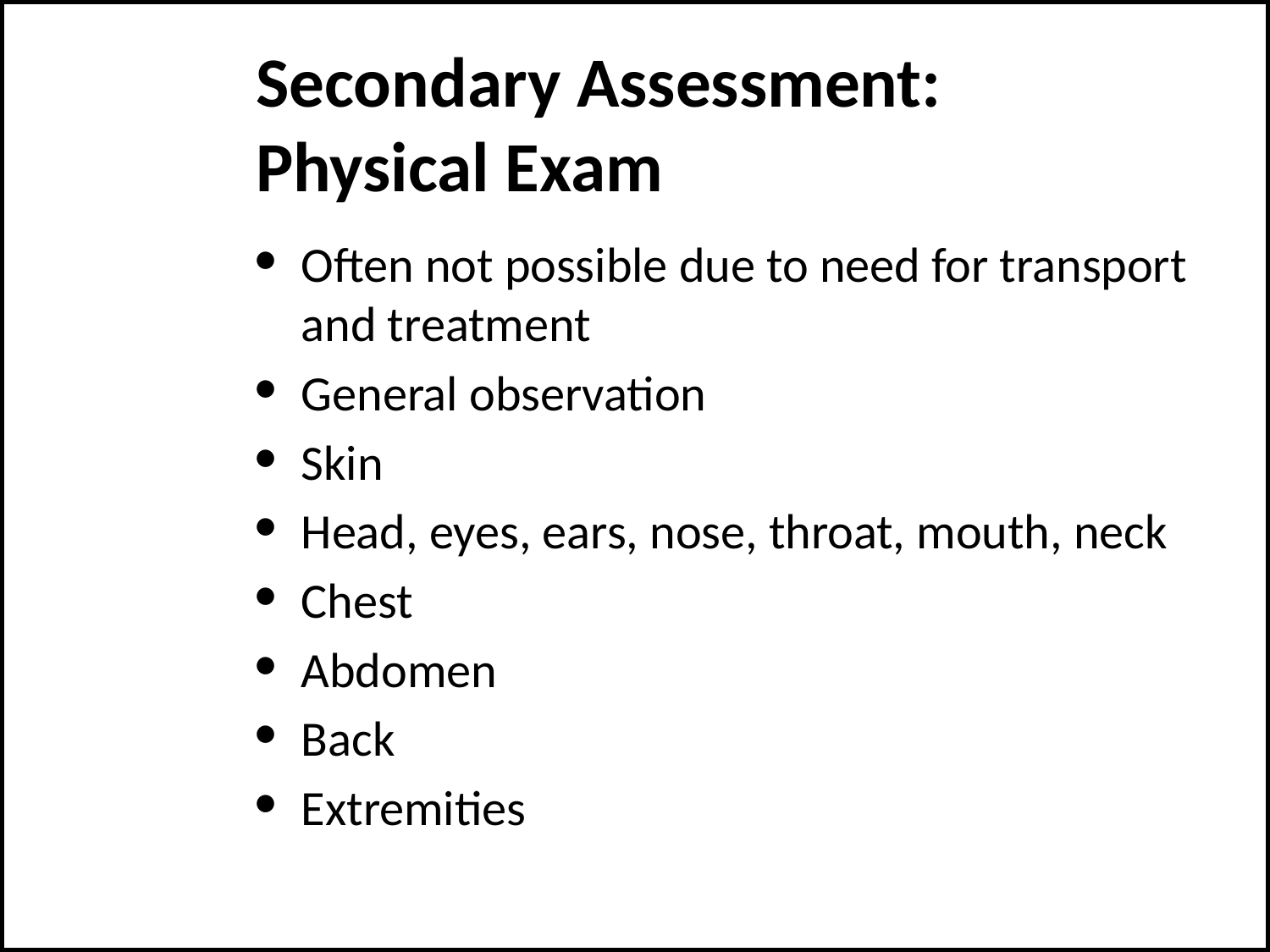

# Secondary Assessment: Physical Exam
Often not possible due to need for transport and treatment
General observation
Skin
Head, eyes, ears, nose, throat, mouth, neck
Chest
Abdomen
Back
Extremities

## Slide 37
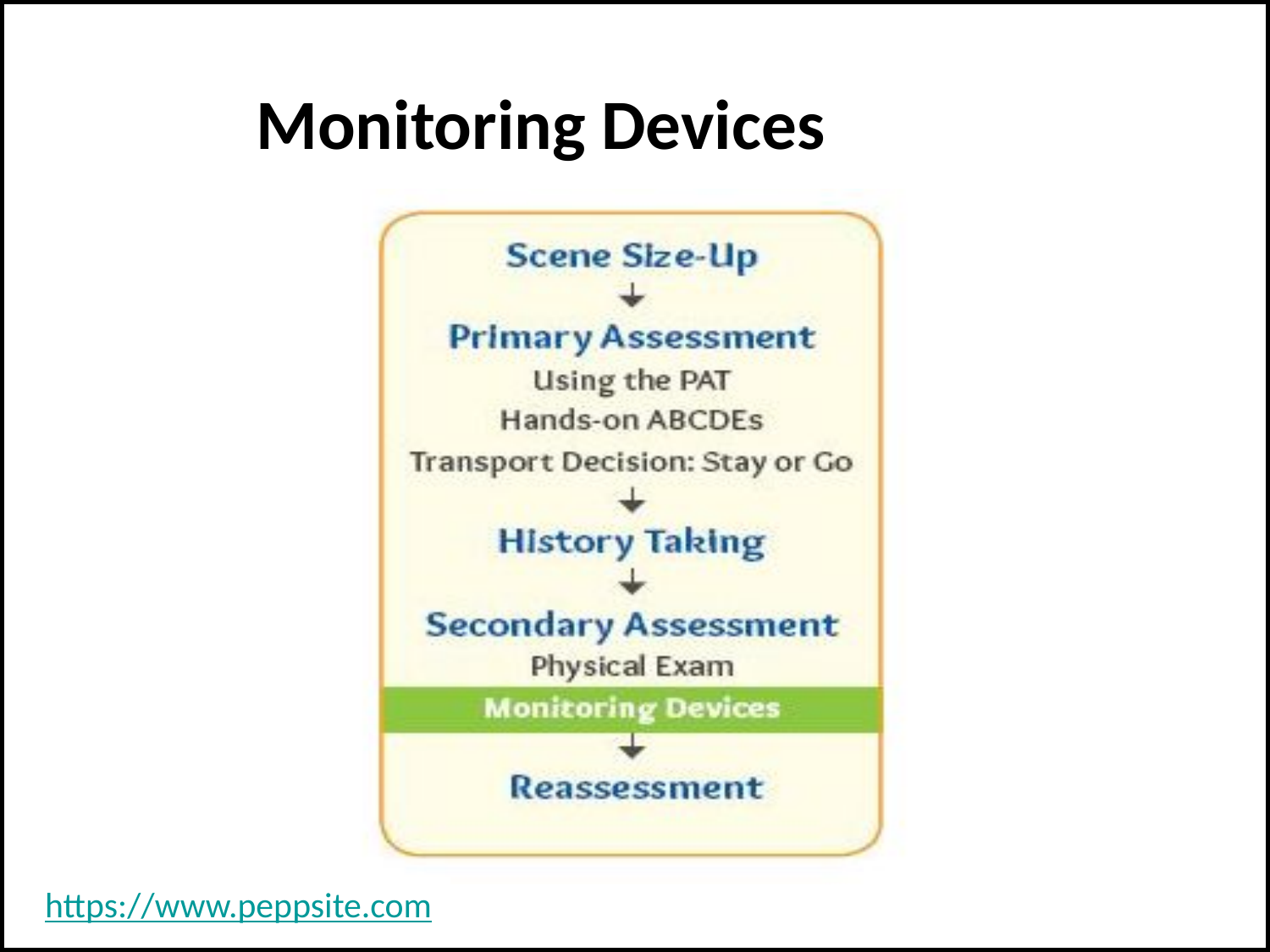

# Monitoring Devices
https://www.peppsite.com

## Slide 38
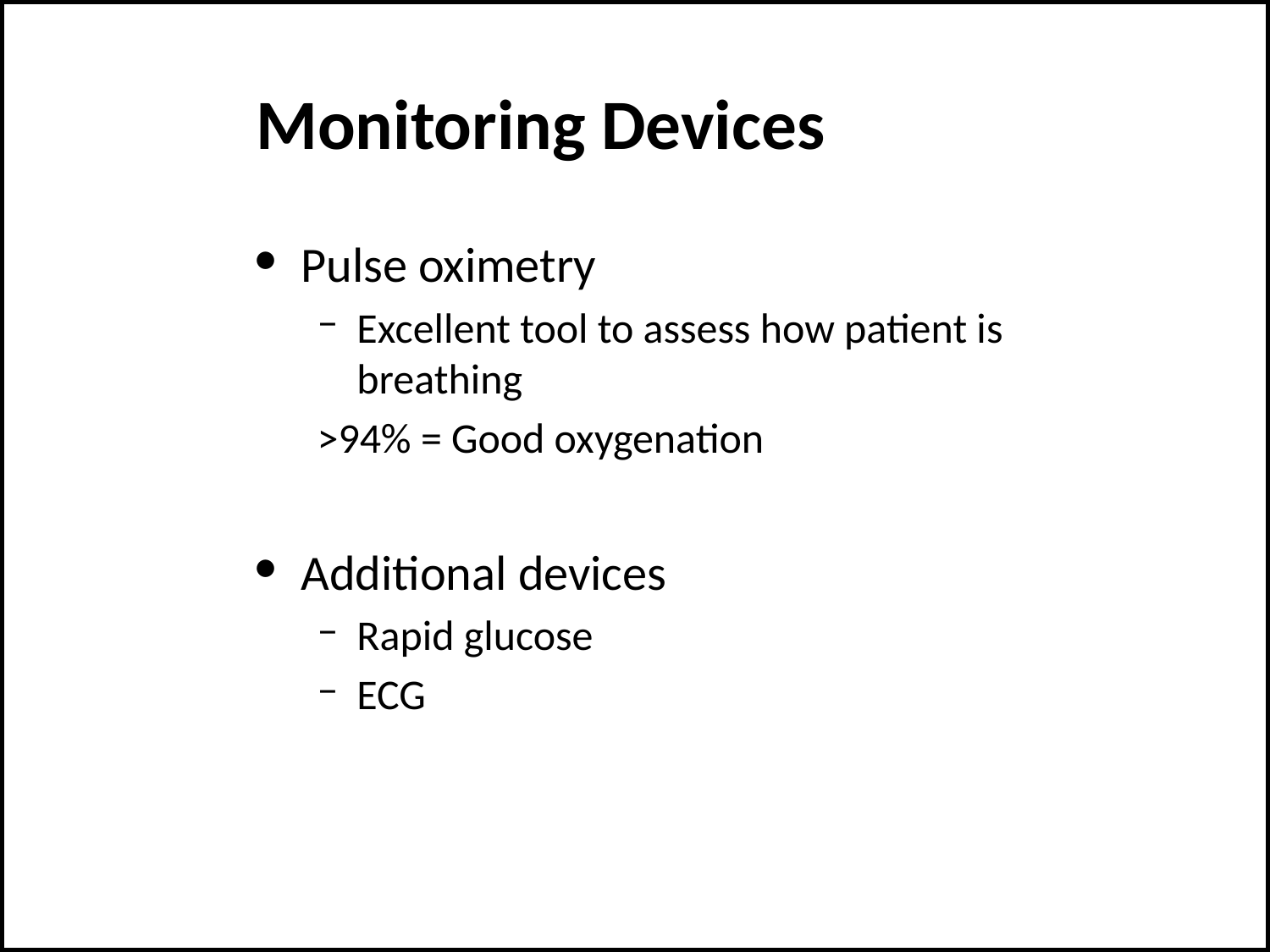

# Monitoring Devices
Pulse oximetry
Excellent tool to assess how patient is breathing
>94% = Good oxygenation
Additional devices
Rapid glucose
ECG

## Slide 39
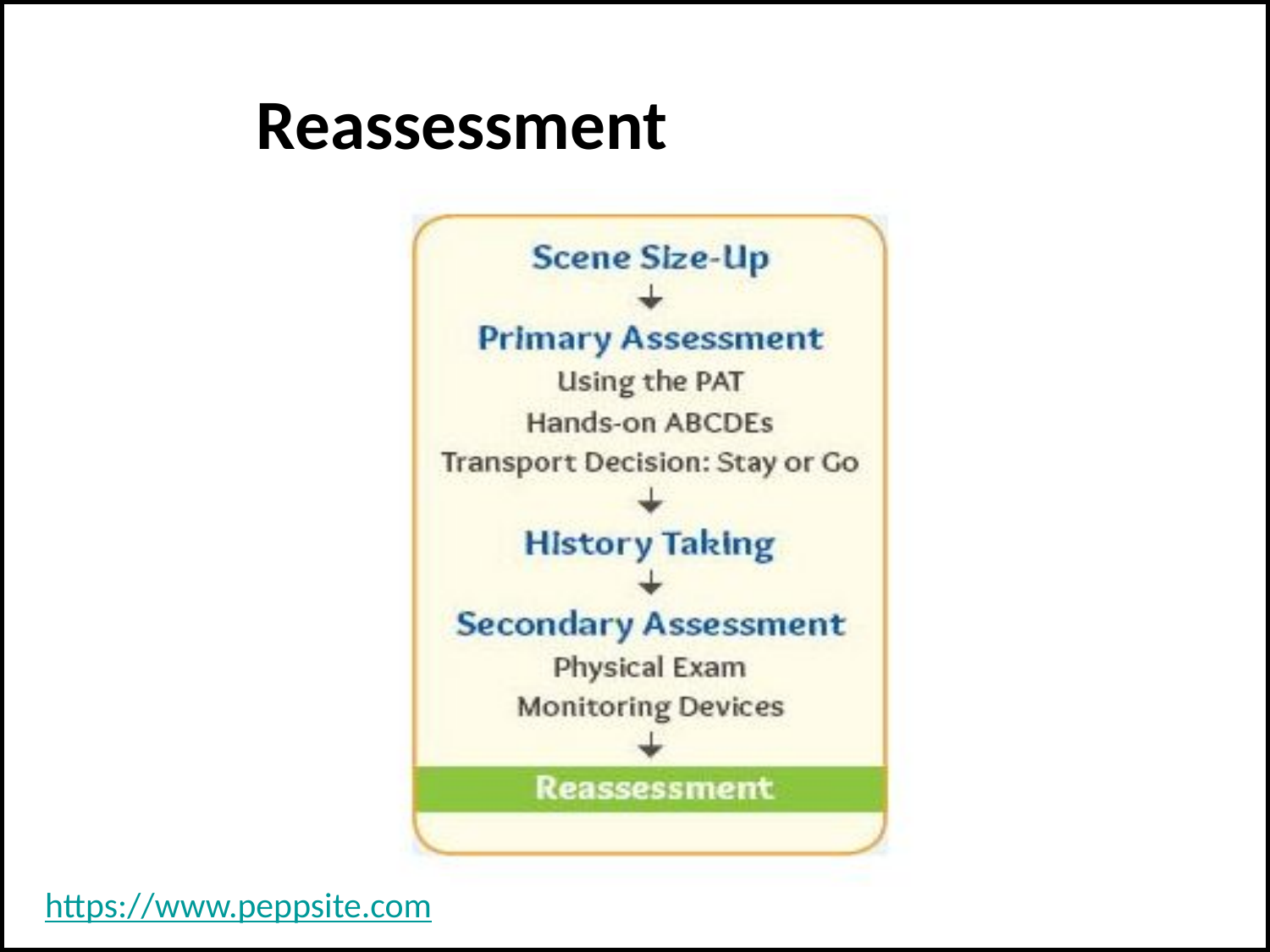

# Reassessment
https://www.peppsite.com

## Slide 40
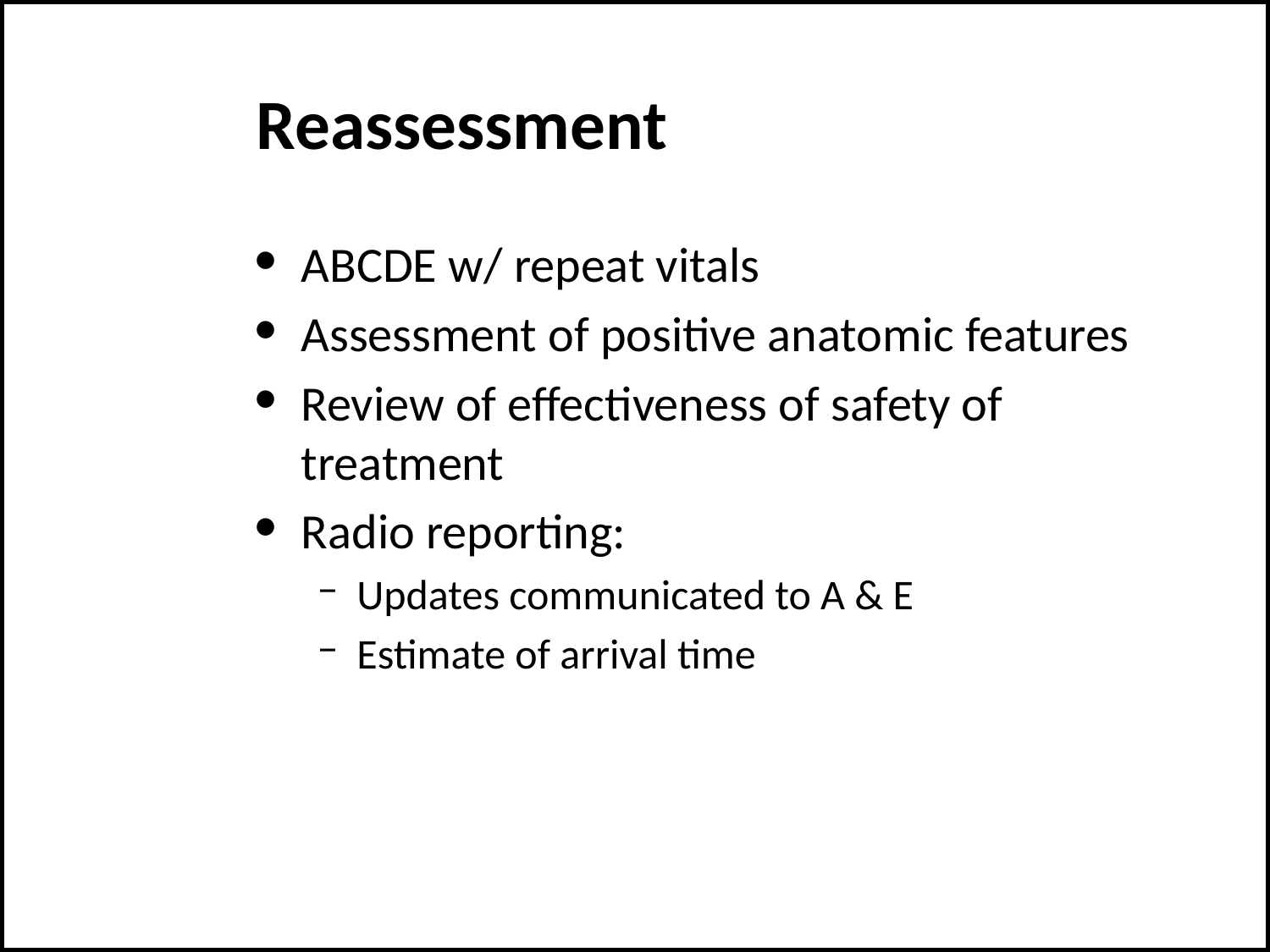

# Reassessment
ABCDE w/ repeat vitals
Assessment of positive anatomic features
Review of effectiveness of safety of treatment
Radio reporting:
Updates communicated to A & E
Estimate of arrival time
